# UBE4B interacts with the ITCH E3 ubiquitin ligase to induce Ku70 and c-FLIPL polyubiquitination and enhanced neuroblastoma apoptosis

**DOI:** 10.1038/s41419-023-06252-7

**Published:** 2023-11-13

**Authors:** Christophe Le Clorennec, Divya Subramonian, Yuchen Huo, Peter E. Zage

**Affiliations:** 1 Department of Pediatrics, Division of Hematology-Oncology, University of California San Diego, La Jolla, CA USA; 2 Peckham Center for Cancer and Blood Disorders, Rady Children’s Hospital, San Diego, CA USA

**Keywords:** Paediatric cancer, Ubiquitylation, Ubiquitylation, Paediatric cancer, Preclinical research

## Abstract

Expression of the UBE4B ubiquitin ligase is strongly associated with neuroblastoma patient outcomes, but the functional roles of UBE4B in neuroblastoma pathogenesis are not known. We evaluated interactions of UBE4B with the E3 ubiquitin ligase ITCH/AIP4 and the effects of UBE4B expression on Ku70 and c-FLIPL ubiquitination and proteasomal degradation by co-immunoprecipitation and Western blots. We also evaluated the role of UBE4B in apoptosis induced by histone deacetylase (HDAC) inhibition using Western blots. UBE4B binding to ITCH was mediated by WW domains in the ITCH protein. ITCH activation led to ITCH-UBE4B complex formation and recruitment of Ku70 and c-FLIPL via ITCH WW domains, followed by Ku70 and c-FLIPL Lys48/Lys63 branched polyubiquitination and proteasomal degradation. HDAC inhibition induced Ku70 acetylation, leading to release of c-FLIPL and Bax from Ku70, increased Ku70 and c-FLIPL Lys48/Lys63 branched polyubiquitination via the ITCH-UBE4B complex, and induction of apoptosis. UBE4B depletion led to reduced polyubiquitination and increased levels of Ku70 and c-FLIPL and to reduced apoptosis induced by HDAC inhibition via stabilization of c-FLIPL and Ku70 and inhibition of caspase 8 activation. Our results have identified novel interactions and novel targets for UBE4B ubiquitin ligase activity and a direct role for the ITCH-UBE4B complex in responses of neuroblastoma cells to HDAC inhibition, suggesting that the ITCH-UBE4B complex plays a critical role in responses of neuroblastoma to therapy and identifying a potential mechanism underlying the association of UBE4B expression with neuroblastoma patient outcomes.

## Introduction

Neuroblastoma is the most common extracranial solid tumor of childhood, and children with high-risk neuroblastoma currently have long-term survival rates under 40% despite intensive, multimodal treatment regimens [1–3]. Furthermore, children with high-risk neuroblastoma frequently suffer from treatment-resistant tumors and disease relapse, and children with recurrent or refractory neuroblastoma respond poorly to additional therapy [4, 5]. A better understanding of the mechanisms of neuroblastoma tumorigenesis will likely provide improved treatment options for these children to reduce relapse rates and improve survival.

The ubiquitin–proteasome system (UPS) manages protein levels in cells via regulation of protein degradation, thereby controlling fundamental cellular processes such as growth, migration, and cell division. Ubiquitination is an essential post-translational modification that covalently links the 76-amino acid ubiquitin polypeptide to target proteins and regulates a wide variety of cellular processes, including both tumor suppressing and promoting pathways. Aberrant ubiquitination has been associated with the etiology of human malignancies [6], and mutations and genetic alterations in genes involved in ubiquitination are found in a wide variety of tumor types and significantly impact patient outcomes [7–11]. Ubiquitination of proteins such as MYCN and p53 has been shown to be important in neuroblastoma pathogenesis [12–14], and the pathways regulating ubiquitination have been identified as candidate therapeutic targets in neuroblastoma cells and tumors [15–20].

The process of protein ubiquitination involves the sequential action of four classes of enzymes, including E1 enzymes that load ubiquitin polypeptides onto E2 enzymes, followed by the transfer of the ubiquitin polypeptides to substrate proteins by substrate-specific E3 ubiquitin ligases. Transfer of additional ubiquitin polypeptides to generate polyubiquitin chains is mediated by E4 ubiquitin ligases, which frequently function in complexes with partner E3 ligases [21–23]. The ubiquitin polypeptide contains seven lysine residues (Lys6, Lys11, Lys27, Lys29, Lys33, Lys48, and Lys63) that can serve as sites for linkage to target proteins, leading to ubiquitin chains of different lengths and configurations [24]. After a single ubiquitin molecule is conjugated to a protein substrate (monoubiquitination), further ubiquitin molecules can be added to yield a polyubiquitin chain (polyubiquitination) or additional ubiquitin molecules can be added to other sites (multi-monoubiquitination). Polyubiquitination of target proteins is often a signal for proteasomal degradation of cytosolic proteins, while mono- and multi-monoubiquitination of proteins can serve as signals for other intracellular protein sorting events [25]. However, the types of linkages between ubiquitin monomers also lead to distinct signaling functions and cellular processes. In general, polyubiquitination reactions are formed on the Lys48 residue, and proteins tagged with Lys48-linked ubiquitin chains are destined for proteasomal degradation [26, 27], whereas modifications involving Lys63-linked chains are more typically involved in regulation of intracellular trafficking and of the localization and activity of multiple kinases and signaling pathways [28–30].

UBE4B has been shown to function as both an E3 and E4 ubiquitin ligase [31, 32], and the *UBE4B* gene is located at chromosome 1p36.22 [33]. Deletions in chromosome 1p36 have been detected in approximately one-third of neuroblastoma tumors and are associated with high-risk tumor features and a poor prognosis [34–36], suggesting a potential role for *UBE4B* as a tumor suppressor gene. *UBE4B* gene expression is strongly associated with neuroblastoma patient outcomes [37, 38], and UBE4B ubiquitin ligase activity on the endosomal membrane surface is required for appropriate EGFR trafficking and lysosomal degradation in neuroblastoma cells [39]. UBE4B has also been shown to ubiquitinate cytosolic proteins, leading to proteasomal degradation [40–44]. The full scope of the targets of UBE4B ubiquitin ligase activity, however, has not been delineated, and while UBE4B is likely involved in regulating the activity of multiple signaling pathways and networks critical for neuroblastoma cell behavior and tumorigenesis, the mechanistic links between UBE4B ubiquitin ligase activity and neuroblastoma pathogenesis that impact patient outcomes are poorly understood.

Ku70 (Lupus Ku autoantigen protein p70; *XRCC6*) is a DNA-binding component of the non-homologous end joining (NHEJ) double strand break (DSB) repair complex, and Ku70 acetylation has previously been shown to mediate neuroblastoma cell apoptosis [45, 46]. Ku70 has also been shown to bind to the anti-apoptotic protein c-FLIPL (cellular Fas-associated death domain-like interleukin-1β-converting enzyme (FLICE)-inhibitory protein, long) [47, 48] and the proapoptotic protein Bax [49]. Ku70 acetylation leads to release of c-FLIPL and Bax, allowing for c-FLIPL degradation [47] and Bax activation, leading to cell death [50, 51]. We hypothesized that UBE4B would interact with members of DNA damage and repair and apoptotic pathways, potentially mediating the observed associations between *UBE4B* expression and patient outcomes, and we therefore hypothesized that regulation of the levels of the Ku70 and c-FLIPL proteins by UBE4B-mediated ubiquitination would impact responses of neuroblastoma tumors to therapy and contribute to the observed associations between *UBE4B* expression and patient outcomes.

## Materials and methods

### Cells and culture conditions

The human neuroblastoma cell lines (SK-N-AS; SK-N-BE;(2) SK-N-SH; SH-SY5Y; IMR-32; SH-EP) used in this study have been previously utilized by our laboratory [52–54] and were purchased from American Type Culture Collection (ATCC, www.atcc.org) or were generously provided by Susan Cohn (The University of Chicago Children’s Hospital, Chicago, IL) or John Maris (Children’s Hospital of Philadelphia, Philadelphia, PA). HEK 293T cells were purchased from ATCC. Cell lines were grown at 37 °C in 5% CO_2_ in RPMI media (Corning Life Science, Corning, NY) supplemented with 10% heat-inactivated fetal bovine serum (FBS) (Life Technologies, Grand Island, NY), 1% L-glutamine, 1% sodium pyruvate, and 1% non-essential amino acids (Sigma-Aldrich, St. Louis, MO). HEK 293T cells were grown in DMEM (Corning Life Science) supplemented with 10% heat-inactivated FBS (Life Technologies) and 1% L-glutamine at 37 °C in 5% CO_2_. All cell lines were passaged to maintain 20–80% confluence and were authenticated by DNA profiling prior to use.

SK-N-AS neuroblastoma cells with UBE4B depletion generated by lentiviral depletion of UBE4B and control SK-N-AS cells expressing a scrambled shRNA were generated using specific *UBE4B* shRNA sequences as well as scrambled control shRNA (Supplemental Table 1) as previously described [55]. SK-N-AS cells with UBE4B overexpression were generated as previously published [39], and SK-N-AS cells with overexpressed and depleted UBE4B were obtained from Dr. Andrew Bean (Rush University, Chicago, IL). SK-N-BE(2), SK-N-SH, SH-SY5Y, and SH-EP cells with UBE4B knockdown were generated using CRISPR/Cas9 techniques as detailed below. HEK 293T cells were transiently transduced with plasmid constructs as detailed below. Protein expression in all transfected and transduced cell lines was validated by Western blot immediately prior to use.

### Expression plasmids and constructs

Plasmid constructs with Myc-tagged ITCH were a gift from Professor Gerry Melino, (MRC Toxicology Unit, Cambridge, UK); Flag-tagged Ku70, c-FLIP(L), and c-FLIP(S) expression constructs were generated in the pCMV-3Tag-6 vector, and Myc-tagged Ku70 was generated in the pCMV-3Tag-7 vector (Agilent Technologies, Santa Clara, CA). Flag-tagged ITCH deleted WW mutants and Flag-tagged ITCH phosphomimic mutants were gifts from Dr. Venuprasad K. Poojary (UT Southwestern, Dallas, TX).

2 × 10^6^ HEK 293T or SK-N-AS cells were plated in 10 cm dishes with complete medium for transient transfections. When they reached 50–70% confluence, cells were transfected using Jet-Optimus DNA transfection reagent (VWR; Visalia, CA) with 2 µg/plate of each vector (Myc-ITCH, Flag-Ku70; Myc-Ku70; Flag-ITCH deleted WW mutants; Flag-ITCH phosphomimic mutants; Flag-c-FLIPL or V5-c-FLIPL) for 24–36 h before use.

### Generation of stable UBE4B knockout cells by CRISPR

SK-N-SH, SH-SY5Y, SH-EP, and SK-N-BE(2) neuroblastoma cell lines with depleted UBE4B were generated using lentiviral particles produced in HEK 293T cells using the Third Generation Packaging Mix and UBE4B sgRNA CRISPR/Cas9 All-in-One Lentivector set (Human) containing plasmid pLenti-U6-sgRNA-SFFV-Cas9-2A-Puro (Applied Biological Materials; Richmond, BC; Canada) (UBE4B sgRNA sequences listed in Supplemental Table 2). Lentivirus-containing culture media was collected from 293T cells; centrifuged at 300 × *g* for 10 min to remove cells; filtered through a 0.45 µM sterile filter and added to target cells with 6 µg/mL Polybrene. Target cells were incubated at 37 °C with 5% CO_2_ overnight; culture media was replaced with complete media (RPMI supplemented with 10% fetal bovine serum, 2 mM L-glutamine, 1× Antibiotic–Antimycotic solution (Corning Life Science)) and incubated at 37 °C with 5% CO_2_. Infected cells were selected for stable expression with 1 µg/mL puromycin and selected cells lines were maintained by adding 1 µg/mL puromycin in the culture media.

### Therapeutic agents and antibodies

MG132 was purchased from Millipore (Burlington, MA), and Vorinostat (Suberoylanilide Hydroxamic Acid, SAHA) was purchased from Selleck Chemicals (Houston, TX). All compound preparations were stored at −20 °C, with dilutions maintained at 4 °C for experimental use.

All antibodies employed are detailed in Supplemental Table 3. Rabbit polyclonal antibodies to UBE4B and Bax, as well as monoclonal antibodies to Ku70, c-FLIP, c-Myc and Flag were used for immunoblotting as well as co-immunoprecipitation experiments. Mouse monoclonal control IgG1 and rabbit monoclonal IgG1 antibodies were used for co-immunoprecipitation experiments. β-actin mouse antibodies were used for immunoblotting as a protein loading control. All antibodies were diluted in blocking buffer (5% BSA in TBST (TBS + 0.1% Tween 20)) to achieve the specified concentrations/dilutions (Supplemental Table 3).

### Cell lysis and immunoprecipitation

All cell lysis and co-immunoprecipitation experiments were performed in 3-((3-cholamidopropyl) dimethylammonio)-1-propanesulfonate (CHAPS) Buffer (CHAPS 1%, 10 mM HEPES, 150 mM NaCl; Sigma-Aldrich). Transfected cells or treated cells grown in 10 cm dishes were lysed in CHAPS buffer containing the protease inhibitor cocktail V (Calbiochem, Billerica, MA) and the phosphatase inhibitor cocktail II (Sigma-Aldrich). After protein quantification with a Bicinchoninic Acid (BCA) assay kit (Thermo Fisher Scientific, San Diego, CA), 1–2 mg of total protein cell lysate was pre-cleared by overnight addition of 40 μL of protein A/G Plus agarose beads (Santa Cruz Biotechnology, Dallas, TX). The supernatant was then incubated with 2 μg of the antibody of interest at 4 °C for 6 h before overnight incubation with 40 μL of protein A/G Plus agarose beads at 4 °C under agitation. Samples were washed three times with 400 μL CHAPS buffer, re-suspended in 100 μL of 2× SDS Laemmli buffer (1 mM TRIS pH 6.8, 1% SDS, 40% Glycerol, 0.1% Bromophenol blue, 20% β-Mercaptoethanol) and heated at 95 °C for 10 min before electrophoresis.

### Western blotting

HEK 293T cells and neuroblastoma cells were plated in 6-well plates or 10 cm tissue culture dishes in media with 10% FBS and were cultured at 37 °C to reach 80–90% confluence. Media was removed and replaced by 10 mL of new warm complete media with or without added SAHA at doses between 0.5 and 5 μM, with or without pretreatment with 5 μM MG132. After the specified treatment duration, cells were collected and lysed in CHAPS buffer. At each specified time point, the supernatant was removed and centrifuged at 1300 RPM, and the pellet was washed one time in 1× PBS. Adherent cells were washed with PBS, and 1 mL of CHAPS lysis buffer was added to the plate. Plates were incubated on a rotating platform for 30 min and then scraped into 1.5 mL Eppendorf tubes. The pellet from the supernatant was added with the lysis buffer containing the cells from the plate and rotated overnight at 4 °C. Protein lysates were then centrifuged at 1400RPM for 10 min, and the cell lysate supernatant was transferred to another 1.5 mL tube.

Protein concentrations in each cell lysate were measured using the BCA kit as above. 1 mg protein lysate was directly mixed with Laemmli buffer and heated at 95 °C for 5 min. Equal amounts of protein (30 μg) were loaded and separated by SDS-PAGE using reducing conditions. Proteins were transferred to PVDF membranes (Thermo Fisher Scientific) by liquid transfer using a mini trans-blot electrophoretic transfer cell (Biorad). Membranes were blocked in 5% BSA in TBST (TBS + 0.1% Tween 20) for 1 h at room temperature and then incubated overnight at 4 °C with the relevant primary antibodies in 5% BSA in TBST and then washed 2 or 3 times in TBST. Membranes were incubated for 1–2 h at room temperature or overnight at 4 °C with HRP-conjugated Goat anti-rabbit IgG (H + L) or Goat anti-mouse IgG (H + L) secondary antibodies (Supplemental Table 3). Signal was visualized using Amersham ECL Prime Luminol Enhancer and Peroxide Solution (GE Healthcare, Piscataway, NJ), and membranes were developed using SuperSignal™ West Pico Plus Chemiluminescent Substrate (Thermo Fisher Scientific). Membranes were exposed to film using Amersham Biosciences Hypercassettes and Denville Scientific HyBlot CL Films (Thomas Scientific, Swedesboro, NJ), and film was developed in an ECOMAX™ x-ray film processor (Protec, Oberstenfeld, Germany). Band intensity levels were quantified using ImageJ software (v. 1.53t; NIH) and were reported as either fold changes in band intensity or as arbitrary units of intensity. Error bars corresponding to standard deviations between three independent replicates were generated using GraphPad Prism (v10.0.1) for each experiment. Statistical comparisons were made using ANOVA with Dunnett’s multiple comparison test.

### Protein–protein docking analyses

Protein–protein docking structures were obtained by using the Ku70 structure from PDB (ID: 1jeq, chain A) and the WW3-WW4 ITCH structure without TXNIP (5cq2, chain A) in the protein–protein docking website clusPro 2.0 (https://cluspro.bu.edu/login.php), and the Ku70-WW3 ITCH docking structure was modeled with PyMOL2 (Shrödinger, LLC).

## Results

### Identification of Ku70 and c-FLIPL as targets of an ITCH-UBE4B E3-E4 ubiquitin ligase complex

**Fig. 1:**
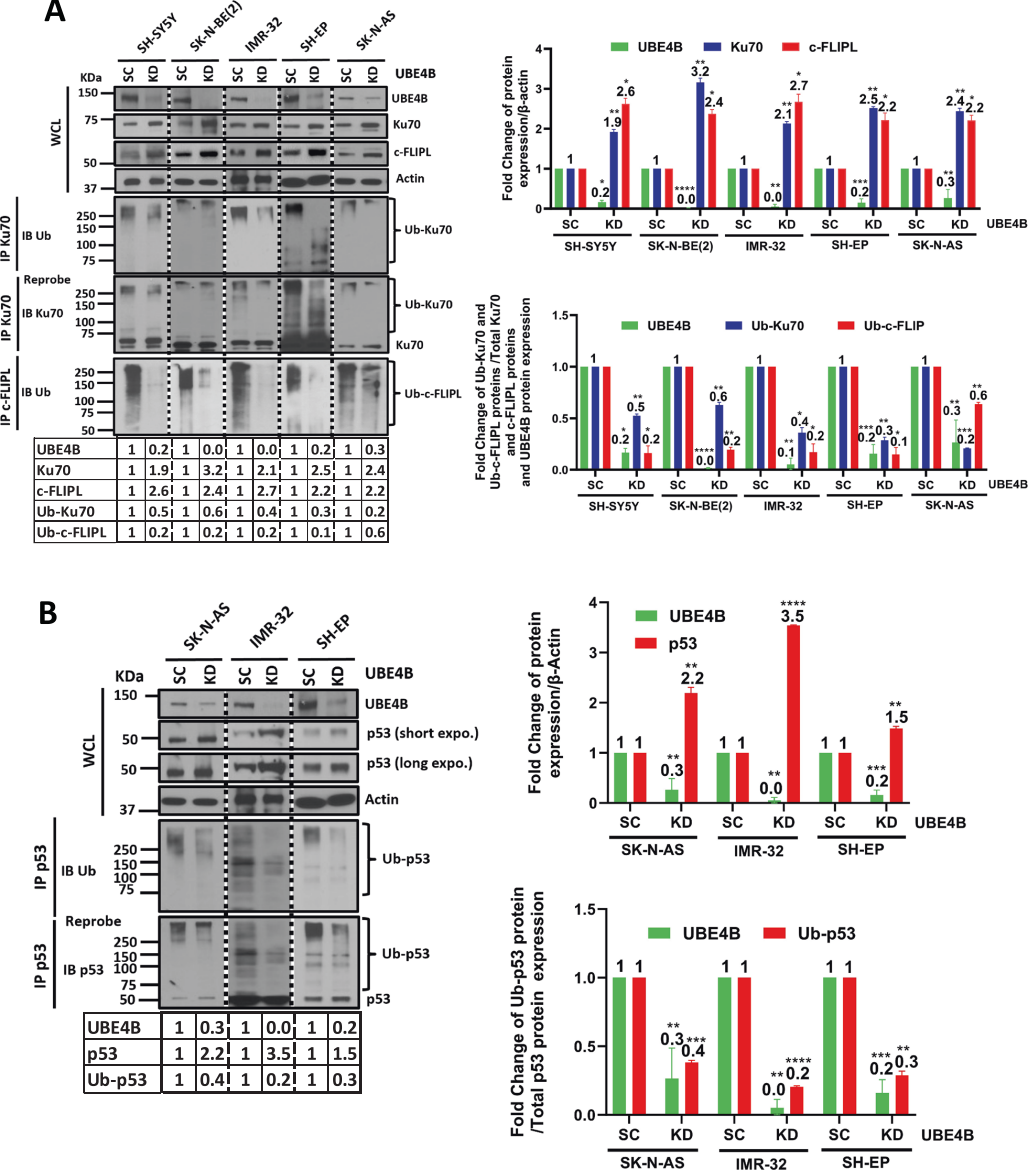
UBE4B depletion results in increased Ku70, c-FLIPL and p53 levels and decreased Ku70, c-FLIPL and p53 ubiquitination in neuroblastoma cells. **A** Neuroblastoma cell lines with depleted UBE4B (KD) and control cells with scrambled vectors (SC) were lysed and Western blots of whole cell lysates (WCL) were performed for UBE4B, Ku70, c-FLIPL, and actin (Top Left). Immunoprecipitated Ku70 was isolated by electrophoresis and Western blots were performed for ubiquitin and for Ku70 (Bottom Left). Band intensities of Ku70 (blue), c-FLIPL (red) and UBE4B (green) were normalized to β-actin expression and compared against control cells with scrambled vectors (SC) (Top Right). Band intensities for UBE4B (green) and for ubiquitinated c-FLIPL (red) and Ku70 (blue) (normalized to total c-FLIPL and Ku70 expression levels) were also compared as fold change between UBE4B KD and scrambled control conditions (Bottom Right). **B** Using the same WCLs of neuroblastoma cell lines with depleted UBE4B and control cells from above, Western blots for p53 were performed (Top Left). Images of UBE4B and actin Western blots from (**A**) above were duplicated for ease of comparison. Immunoprecipitated p53 was isolated by SDS-Page and Western blots and immunoblotting for ubiquitin and p53 were performed (Bottom Left). Band intensities of total p53 (red) and UBE4B (green) were compared between SC and UBE4B KD conditions (Top Right), and band intensities of total UBE4B (green) and ubiquitinated p53 (normalized to total p53 levels; red) were compared between SC and UBE4B KD conditions (Bottom Right) (*p < 0.05, **p < 0.01, ***p < 0.001, ****p < 0.0001).

The DNA damage repair protein Ku70 has previously been associated with induction of neuroblastoma cell death [45, 46] and has also previously been shown to interact with the anti-apoptotic protein c-FLIPL. To explore the potential roles of Ku70 and c-FLIPL as mediators of the established association between *UBE4B* expression and neuroblastoma patient outcomes, we hypothesized that Ku70 and c-FLIPL could be targets of UBE4B ubiquitin ligase activity. Neuroblastoma cell lines with depleted UBE4B were analyzed by Western blot for Ku70 and c-FLIPL protein levels, and increased Ku70 and c-FLIPL protein levels were seen in all cell lines with depleted UBE4B (Fig. 1A). UBE4B depletion was also associated with reduced Ku70 and c-FLIPL ubiquitination (Fig. 1A), suggesting that UBE4B depletion leads to reduced Ku70 and c-FLIPL ubiquitin-mediated proteasomal degradation. UBE4B depletion also led to reduced p53 ubiquitination and increased p53 expression in NB cells (Fig. 1B), confirming the previously published role of UBE4B in p53 regulation [40, 44, 56].

**Fig. 2:**
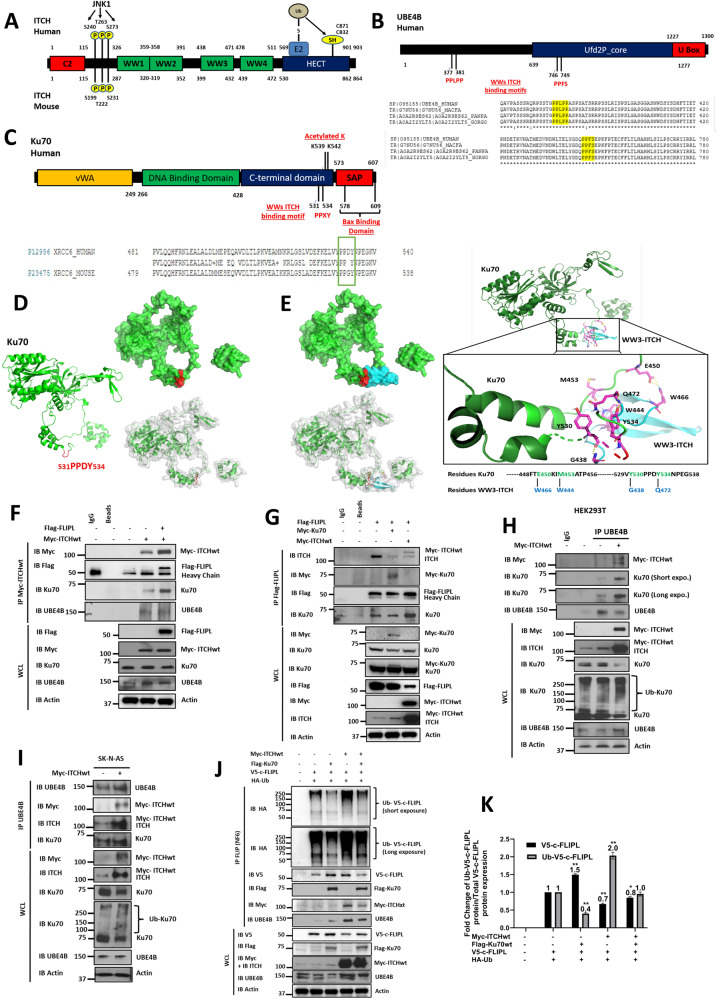
Identification of Ku70 and c-FLIPL as targets of an ITCH-UBE4B E3-E4 ubiquitin ligase complex. **A** Schematic Structure of ITCH, with an N-terminal Ca^2+^/lipid-binding (C2) domain (red box), a proline rich motif with three sites that undergo JNK-mediated phosphorylation, four WW domains (green boxes) for interactions with PPxY motifs in target proteins, and a C-terminal HECT domain (blue box) capable of interacting with E2 enzymes for ubiquitin loading. **B** Schematic structure of UBE4B with the Ufd2P core (blue box) and the U Box domain (red box), with candidate ITCH binding motifs in the UBE4B protein sequences shown. **C** Schematic structure of Ku70, with a vWA domain (yellow box), a DNA Binding domain (green box), a C-terminal domain (blue box) with two sites for acetylation, and a SAP domain (red box) capable of Bax binding, with candidate PPxY ITCH binding motifs in the mouse and human Ku70 protein sequences shown. **D** Structure of Ku70 with the PPxY motif shown in red (531PPDY534) in the C-terminal part of Ku70. Ku70 structure was obtained at PDB (ID: 1jeq, chain A corresponding to Ku70) and modeled using PyMOL2 (Shrödinger, LLC). **E** Modeling of Ku70-WW3 ITCH protein-protein interaction identified interacting residues between Ku70 and WW3-ITCH (E450-W466; M453-W444; Y530-G438; Y534-Q472). **F** Whole cell lysates (WCL) of HEK 293T cells expressing full length Myc-ITCH (Myc-ITCHwt) and Flag-c-FLIPL were evaluated by Western blot for Flag, Myc, Ku70, UBE4B, and actin (Bottom), and immunoprecipitated Myc-ITCH was isolated and Western blots were performed for Myc, Flag, Ku70, and UBE4B (Top). **G** WCLs of HEK 293T cells expressing Flag-c-FLIPL alone or with Myc-ITCH or Myc-Ku70 were evaluated by Western blot for Myc, Ku70, Flag, ITCH, and actin (Bottom), and immunoprecipitated Flag-c-FLIPL was isolated and Western blots were performed for ITCH, Myc, Flag, and Ku70 (Top). **H** WCLs of HEK 293T cells expressing Myc-ITCH were evaluated by Western blot for Myc, ITCH, Ku70, UBE4B, and actin (Bottom), and immunoprecipitated UBE4B was isolated and Western blots were performed for Myc, Ku70, and UBE4B (Top). **I** WCLs of SK-N-AS neuroblastoma cells expressing Myc-ITCH were evaluated by Western blot for Myc, ITCH, Ku70, UBE4B, and actin (Bottom), and immunoprecipitated UBE4B was isolated and Western blots were performed for UBE4B, Myc, ITCH, and Ku70 (Top). **J** WCLs of HEK 293T cells co-transfected with V5-c-FLIPL, HA-ubiquitin Myc-ITCH and/or Flag-Ku70 were evaluated by Western blot for V5, Flag, Myc, ITCH, UBE4B, and actin (Bottom), and immunoprecipitated V5-c-FLIPL was isolated and Western blots were performed for V5, HA, Flag, Myc, and UBE4B (Top). **K** Band intensities of total (black) and ubiquitinated V5-c-FLIPL (gray) in WCLs were compared between Myc-ITCHwt overexpression or/and Flag-Ku70 overexpression conditions and V5-c-FLIPL overexpression alone as a control condition (*p < 0.05, **p < 0.01).

We hypothesized that the effects of UBE4B depletion on Ku70 and c-FLIPL could be mediated by polyubiquitination induced by UBE4B E4 ubiquitin ligase activity in collaboration with other E3 ubiquitin ligases. Analogous to *UBE4B* [37], reduced expression of the gene for the HECT family E3 ubiquitin protein ligase ITCH (also called Atrophin-1-interacting protein 4 (AIP4); Fig. 2A) is associated with stage 4 disease, *MYCN* amplification, and poor neuroblastoma patient outcomes (Supplemental Fig. 1). By protein sequence analysis, we identified two specific ITCH binding motifs in the UBE4B protein sequence (Fig. 2B), suggesting that ITCH and UBE4B may serve as an E3-E4 ubiquitin ligase complex. We further identified a “PPxY” ITCH-binding motif in the C-terminal part of the Ku70 protein sequence (531PPDY534, Fig. 2C), and we analyzed the structure of Ku70 to determine the specific conformation of this binding motif in Ku70 (Fig. 2D). By protein-protein docking analysis, we identified potential interactions between four residues of Ku70 and the WW3 domain of ITCH (E450-W466; M453-W444; Y530-G438; Y534-Q472) immediately adjacent to the “PPxY” motif, suggesting direct binding of Ku70 with the WW3 domain of ITCH (Fig. 2E).

To evaluate potential physical interactions between Ku70, c-FLIPL, and UBE4B with ITCH, cells transfected with tagged ITCH, c-FLIPL, and Ku70 were evaluated by immunoprecipitation. Ku70 and UBE4B were co-immunoprecipitated with myc-ITCH (Fig. 2F). FLAG-c-FLIPL also interacted with myc-ITCH and endogenous Ku70 and ITCH (Fig. 2F, G), suggesting the existence of an ITCH-UBE4B complex that includes both Ku70 and c-FLIPL. We further confirmed associations between ITCH and UBE4B, with co-immunoprecipitation of myc-ITCH and Ku70 with UBE4B (Fig. 2H). Moreover, myc-ITCH overexpression resulted in reduced total levels of c-FLIPL and Ku70 in whole cell lysates (Fig. 2G–I) and led to increased Ku70 ubiquitination (Fig. 2H, I), suggesting that ITCH overexpression can induce its own activation and subsequent ubiquitination and degradation of Ku70. Myc-ITCH overexpression further led to increased UBE4B binding to ITCH and to increased binding of ITCH to Ku70 and c-FLIPL (Fig. 2I) with increased c-FLIPL ubiquitination that was mitigated by concurrent overexpression of Ku70 (Fig. 2J, K). Conversely, c-FLIPL ubiquitination was significantly decreased and total c-FLIPL levels increased after overexpression of Ku70 (Fig. 2J, K).

### UBE4B, Ku70, and c-FLIPL interact with distinct domains of ITCH

**Fig. 3:**
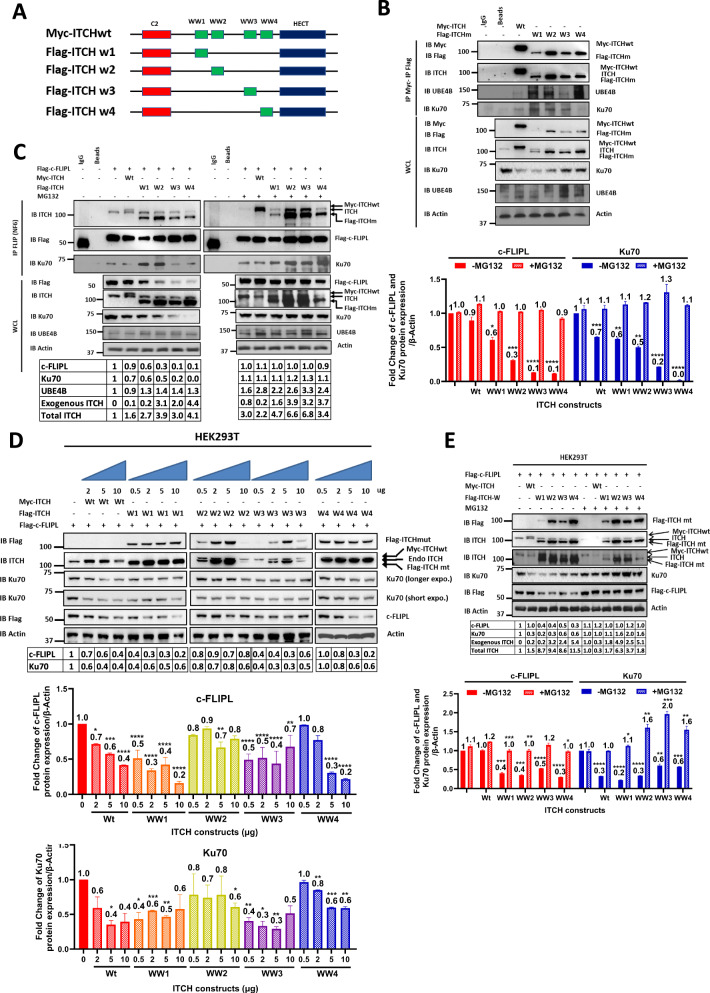
Determination of the ITCH domains interacting with UBE4B, Ku70, and c-FLIPL. **A** Schematic structures of full length Myc-ITCH (Myc-ITCHwt) and of Flag-ITCH mutants with deletions of WW domains (Flag-ITCHw1, Flag-ITCHw2, Flag-ITCHw3, Flag-ITCHw4). Each Flag-ITCH WW deleted mutant has an intact C2 domain and a functional HECT domain. **B** Whole cell lysates (WCL) of HEK 293T cells transfected with Myc-ITCHwt or individual Flag-ITCH WW deleted mutants were analyzed by Western blot for Myc, Flag, ITCH, Ku70, UBE4B, and actin (Bottom), and immunoprecipitated ITCH (via either Myc or Flag) was isolated and analyzed by Western blot for Myc, Flag, ITCH, UBE4B, and Ku70 (Top). **C** WCLs of HEK 293T cells co-transfected with Flag-c-FLIPL and either Myc-ITCHwt or individual Flag-ITCH WW deleted mutants with or without 8 h of treatment with 5 µM of MG132 24 h after transfection were evaluated by Western blot for Flag, ITCH, Ku70, UBE4B, and actin (Left, lower lanes), and immunoprecipitated c-FLIP (using a mouse monoclonal c-FLIP antibody (NF6)) from these same cell lines was isolated and evaluated by Western blot for ITCH, Flag, and Ku70 (Left, upper lanes). Band intensities relative to control lanes and normalized to β-actin expression are shown in the table. Normalized band intensities of c-FLIPL (red) and Ku70 (blue) from the WCL were compared with and without MG132 treatment (Right). **D** WCLs of HEK 293T cells expressing Flag-c-FLIPL and either Myc-ITCHwt or individual Flag-ITCH WW deleted mutants transfected with increasing doses of vector (0.5 μg; 2 μg; 5 μg; 10 μg) were evaluated by Western blots for Flag, ITCH, Ku70, and actin (Top). Band intensities relative to control lanes and normalized to β-actin expression are shown in the table. Normalized band intensities of c-FLIPL and Ku70 were compared to evaluate the effect of increasing doses of each Flag-ITCH WW deleted mutant protein on the expression levels of c-FLIPL and Ku70 (Bottom) (*p < 0.05, **p < 0.01, ***p < 0.001, ****p < 0.0001). **E** WCLs of HEK 293T cells expressing Flag-c-FLIPL and either Myc-ITCHwt or individual Flag-ITCH WW deleted mutants with or without 8 h of treatment with 5 µM of MG132 24 h after transfection were evaluated by Western blots for Flag, ITCH, Ku70, Flag, and β-actin. Band intensities relative to control lanes and normalized to β-actin expression are shown in the table. Normalized band intensities of c-FLIPL (red) and Ku70 (blue) were compared with and without MG132 treatment (*p < 0.05, **p < 0.01, ***p < 0.001, ****p < 0.0001).

The ITCH ubiquitin ligase binds to target proteins via its 4 WW domains, with the HECT domain mediating E2 ubiquitin ligase binding and transfer of ubiquitin polypeptides to target proteins [57]. To determine which domains of ITCH were required for binding to UBE4B, Ku70, and c-FLIPL, we employed ITCH constructs expressing single WW domains with conserved C2 and active HECT domains (Fig. 3A). Using co-immunoprecipitation experiments, UBE4B bound strongly to the WW4 domain, with less binding to the WW1 and WW2 domains (Fig. 3B). Ku70 bound most strongly to the WW1, WW2, and WW3 domains, suggesting that in the ITCH-UBE4B-Ku70 complex, UBE4B is likely bound to the WW4 domain, with Ku70 interacting with other WW domains. In contrast, c-FLIPL appeared to be able to interact with all 4 WW domains, with reduced total c-FLIPL protein levels seen in each case (Fig. 3C). Increased levels of full-length ITCH induced by transfection with increasing vector doses were also associated with reduced levels of both Ku70 and c-FLIPL (Fig. 3D), while increasing doses of individual mutant vectors resulted in the most significant reduction in total levels of Ku70 with the WW4 ITCH mutant and in c-FLIPL levels most significantly with the ITCH WW1 and WW4 constructs (Fig. 3D), suggesting that UBE4B–ITCH interaction via the WW4 domain is required for the observed decreased levels in c-FLIPL and Ku70. Pre-treatment with the proteasome inhibitor MG132 abrogated the reduction of both Ku70 and c-FLIPL levels induced by expression of ITCH WW constructs, further suggesting that the reduced levels observed with ITCH construct expression were due to proteasomal degradation (Fig. 3C, E).

### The ITCH-UBE4B complex induces Lys48/Lys63-branched polyubiquitin chains on Ku70 and c-FLIPL

**Fig. 4:**
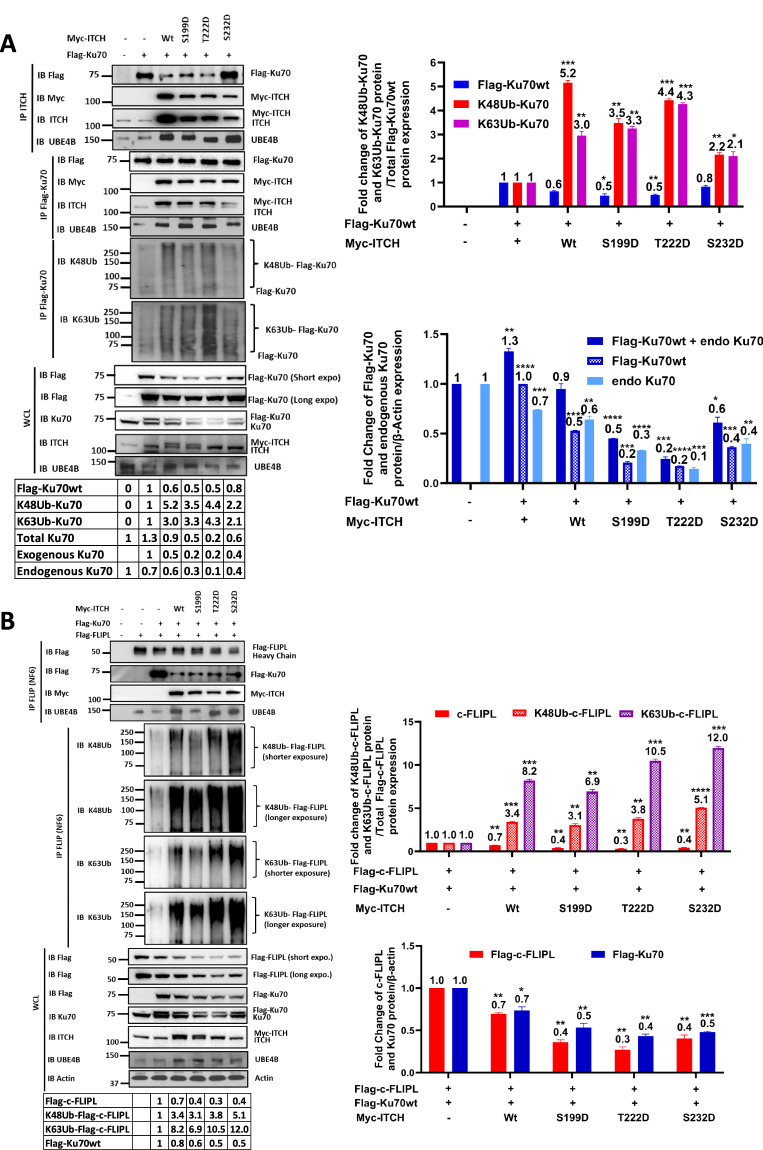
The ITCH-UBE4B complex induces Lys48/Lys63-branched polyubiquitination of Ku70 and c-FLIPL. **A** Whole cell lysates (WCL) of HEK 293T cells expressing Flag-Ku70 and either Myc-ITCHwt or individual Myc-ITCH phosphomimic mutants (S199D, T222D, or S232D) [58] were evaluated by Western blots for Flag, Ku70, ITCH, and UBE4B (Bottom), and immunoprecipitated ITCH and Ku70 were isolated and evaluated by Western blots for Flag, Myc, ITCH, UBE4B, Lys48-linked ubiquitin (K48Ub), and Lys63-linked ubiquitin (K63Ub) (Top). Band intensities relative to control lanes are shown in the table. Band intensities of total Flag-Ku70 (blue), Lys48-linked ubiquitinated (K48Ub) Ku70 (orange) and Lys63-linked ubiquitinated (K63Ub) Ku70 (red) from each cell line were compared (Top Right). Band intensities of total Ku70, Flag-Ku70, and endogenous Ku70 from each cell line were also compared (Bottom Right). **B** WCLs of HEK 293T cells expressing Flag-Ku70, Flag-c-FLIPL, and either Myc-ITCHwt or individual Myc-ITCH phosphomimic mutants were evaluated by Western blots for Flag, Ku70, ITCH, UBE4B, and β-actin (Bottom), and immunoprecipitated c-FLIPL was isolated and evaluated by Western blots for Flag, Myc, UBE4B, Lys48-linked ubiquitin (K48Ub), and Lys63-linked ubiquitin (K63Ub) (Top). Band intensities relative to control lanes are shown in the table. Band intensities of total c-FLIPL (red), Lys48-linked ubiquitinated (K48Ub) c-FLIPL (orange) and Lys63-linked ubiquitinated (K63Ub) c-FLIPL (purple) from each cell line were compared (Top Right). Band intensities of Flag-c-FLIPL (red) and Flag-Ku70 (blue) were also compared (Bottom Right) (*p < 0.05, **p < 0.01, ***p < 0.001, ****p < 0.0001).

To identify the types of ubiquitin chain linkages induced by the ITCH-UBE4B complex on Ku70, cells transduced to express tagged ITCH and Ku70 were evaluated by immunoblotting for Lys48 and Lys63 ubiquitin chains. At baseline, endogenous ITCH interacts with UBE4B and recruits Ku70, which is polyubiquitinated with both Lys48 and Lys63 linkages (Fig. 4A). With overexpression of tagged wild-type ITCH or constitutively active ITCH phosphomimic mutants (S199D, T222D, or S232D) [58], binding of Ku70 to the ITCH-UBE4B complex was observed, with increasing Lys48 and Lys63 polyubiquitination of Ku70 and with reduced total Ku70 levels in whole cell lysates from cells with wild-type ITCH and the S199D and T222D mutants, suggesting increased degradation (Fig. 4A).

Analogous experiments also demonstrated c-FLIPL binding to the ITCH-UBE4B complex with Ku70 (Fig. 4B). Increased Lys48 and Lys63 polyubiquitination of c-FLIPL was seen with overexpression of tagged wild-type ITCH and with constitutively active ITCH phosphomimic mutants and with associated reductions in both Ku70 and c-FLIPL total protein levels (Fig. 4B), suggesting that the Lys48 and Lys63 polyubiquitin chains contribute to both c-FLIPL and Ku70 proteasomal degradation.

### HDAC inhibition induces Ku70 and c-FLIPL acetylation and Ku70 and c-FLIPL Lys48/Lys63 branched polyubiquitination via the ITCH-UBE4B complex, leading to induction of apoptosis

**Fig. 5:**
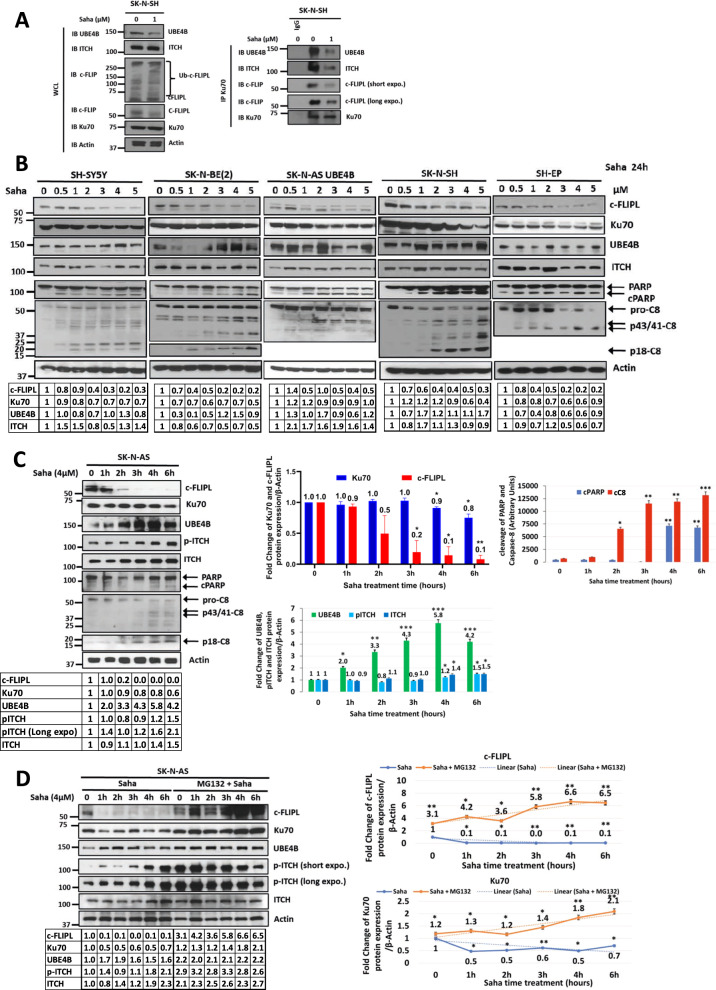

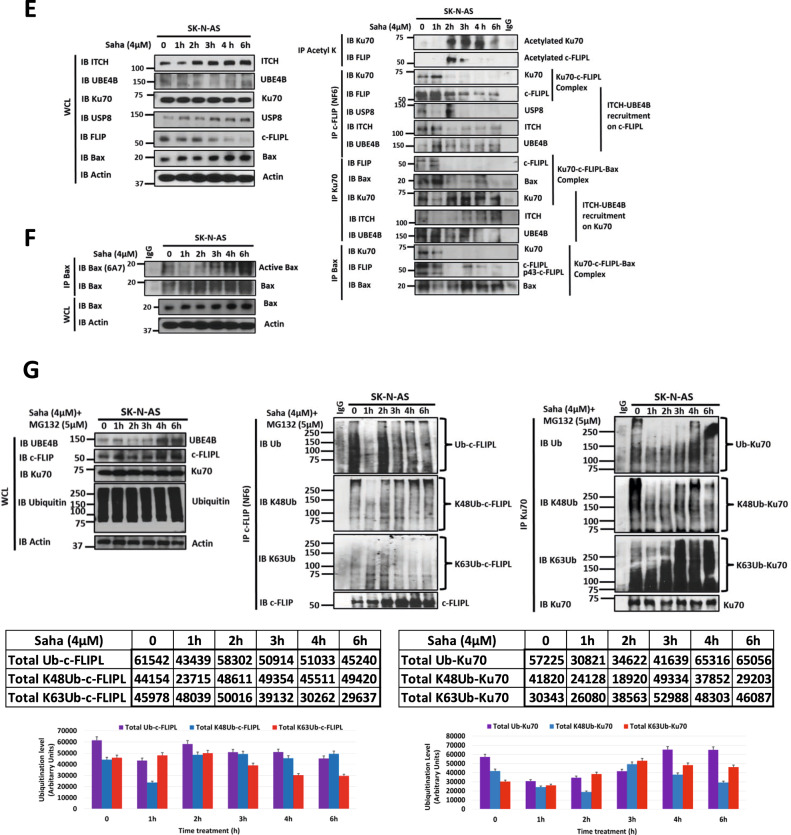
HDAC inhibition induces Ku70 and c-FLIPL acetylation and Ku70 and c-FLIPL Lys48/Lys63 branched polyubiquitination via the ITCH-UBE4B complex. **A** Whole cell lysates (WCL) of SK-N-SH neuroblastoma cells with or without treatment with 1μM vorinostat (Saha) for 24 h were evaluated by Western blots for UBE4B, ITCH, c-FLIPL, Ku70, and actin (Left), and immunoprecipitated Ku70 was isolated and evaluated by Western blots for UBE4B, ITCH, c-FLIPL, and Ku70 (Right). **B** WCLs of neuroblastoma cell lines treated with increasing doses of vorinostat (Saha) for 24 h prior to lysis were evaluated by Western blots for c-FLIPL, Ku70, UBE4B, ITCH, PARP and cleaved PARP (cPARP), caspase-8 and cleaved caspase-8 fragments p43/41 and p18, and β-actin. Band intensities for c-FLIPL, Ku70, UBE4B and ITCH relative to control lanes are shown in the tables. **C** WCLs of SK-N-AS neuroblastoma cells treated with 4μM vorinostat (Saha) for increasing time intervals were evaluated by Western blot for c-FLIPL, Ku70, UBE4B, phosphorylated ITCH (p-ITCH), total ITCH, PARP and cleaved PARP (cPARP), and caspase-8 and cleaved caspase-8 fragments p43/41 and p18, and β-actin (Left). Band intensities relative to control lanes are shown in the tables. Band intensities for c-FLIPL (red) and Ku70 (dark blue) relative to β-actin expression were compared over increasing time of Saha treatment (Top Middle). Band intensities of UBE4B (green), p-ITCH (light blue), and ITCH (dark blue) normalized to β-actin expression were compared at each timepoint (Bottom Middle), and band intensities of cleaved PARP (cPARP; blue) and cleaved caspase-8 fragments (cC8; red) normalized to β-actin expression were also compared at each time point (*p < 0.05, **p < 0.01, ***p < 0.001). **D** WCLs of SK-N-AS neuroblastoma cells treated with 4μM vorinostat (Saha) for increasing duration with or without added 5μM MG132 were evaluated by Western blots for c-FLIPL, Ku70, UBE4B, phosphorylated ITCH (p-ITCH), total ITCH, and β-actin. Band intensities relative to control lanes are shown in the tables. Band intensities for c-FLIPL and Ku70 normalized to β-actin expression were compared in independent graphs in cells treated with Saha without pre-treatment with MG132 (blue lines) and in cells treated with Saha with MG132 pre-treatment (orange lines) (*p < 0.05, **p < 0.01). **E** WCLs of SK-N-AS neuroblastoma cells treated with 4μM vorinostat (Saha) for increasing duration were evaluated by Western blots for ITCH, UBE4B, Ku70, USP8, c-FLIPL, Bax, and β-actin (Left). Immunoprecipitated acetyl-lysine was isolated and evaluated by Western blot for Ku70 and c-FLIPL (Top Right), and immunoprecipitated c-FLIPL, Ku70, and Bax were each isolated and evaluated by Western blots for Ku70, c-FLIPL, and Bax as well as for UBE4B, ITCH and USP8 (Bottom Right). **F** WCLs of SK-N-AS neuroblastoma cells treated with 4μM vorinostat (Saha) for increasing duration were evaluated by Western blots for Bax and β-actin (Bottom), and immunoprecipitated Bax was isolated and evaluated by Western blots for total Bax and activated Bax using an antibody (6A7) which recognizes the active conformation of Bax (Top). **G** WCLs of SK-N-AS neuroblastoma cells pre-treated with 5μM MG132 and treated with 4μM vorinostat (Saha) for increasing duration were evaluated by Western blots for UBE4B, c-FLIPL, Ku70, ubiquitin, and β-actin (Top Left). Immunoprecipitated c-FLIPL (Top Middle) and Ku70 (Top Right) were isolated and evaluated by Western blots for ubiquitin, Lys48-linked ubiquitin (K48Ub), Lys63-linked ubiquitin (K63Ub), c-FLIPL, and Ku70. Lane “0” corresponded to 8 h of MG132 treatment (2 h pretreatment, followed by 6 h of treatment with MG132 plus vehicle (DMSO) used for Saha), while subsequent lanes were from samples treated with MG132 for 2 h, followed by treatment with MG132 plus Saha in DMSO for designated times. Absolute band intensities of total ubiquitinated (violet), Lys48-linked ubiquitinated (K48Ub; blue), and Lys63-linked ubiquitinated (K63Ub; red)) c-FLIPL (Left) and Ku70 (Right) are shown in the tables and were compared over time (Bottom).

Ku70 and the proapoptotic protein Bax have previously been shown to interact in neuroblastoma cells, with Ku70 acetylation leading to Bax and c-FLIPL release, allowing for c-FLIPL degradation [47] and Bax activation, leading to induction of apoptosis [45, 46, 50, 51] and suggesting that Ku70 acetylation plays a key role in neuroblastoma cell death. Histone deacetylase (HDAC) inhibitors such as vorinostat (Suberoylanilide Hydroxamic Acid; SAHA) have been previously shown to promote Ku70 acetylation [59] and to inhibit cell growth and induce differentiation, apoptosis and cell cycle arrest in neuroblastoma cells and tumors [60–63]. To evaluate the effects of HDAC inhibition in neuroblastoma cells, we analyzed neuroblastoma cells treated with SAHA, which induced caspase 3/7 activation and apoptosis (Supplemental Fig. 2). To evaluate the effects of HDAC inhibition in neuroblastoma cells on the ITCH-UBE4B complex and on Ku70 and c-FLIPL, we evaluated SAHA-treated cells by Western blot. Treated cells demonstrated Ku70 and c-FLIPL binding to the ITCH-UBE4B complex, with increased c-FLIPL ubiquitination and reduced total c-FLIPL levels, consistent with degradation (Fig. 5A). SAHA-treated neuroblastoma cells demonstrated dose-dependent decreases in the levels of Ku70 and c-FLIPL, with induction of caspase 8 activation and PARP cleavage indicative of induction of apoptosis (Fig. 5B and Supplemental Fig. 3A). SAHA treatment furthermore induced time-dependent increases in ITCH and UBE4B expression as well as ITCH activation (as determined by ITCH T222 phosphorylation) in parallel with decreases in Ku70 and c-FLIPL protein levels with associated caspase-8 and PARP cleavage (Fig. 5C, D). Proteasomal inhibition resulted in increased levels of of c-FLIPL and Ku70 protein levels with increasing doses of SAHA (Supplemental Fig. 3B) and accumulation of c-FLIPL and Ku70 during extended SAHA treatment (Fig. 5D), further suggesting that HDAC inhibition induces c-FLIPL and Ku70 proteasomal degradation. Proteasomal inhibition also led to increased levels of UBE4B, ITCH, and p-ITCH with increasing SAHA doses (Supplemental Fig. 3B) and in stable levels of UBE4B, ITCH, and p-ITCH during prolonged SAHA treatment (Supplemental Fig. 3C).

To evaluate whether the induction of apoptosis by HDAC inhibition is mediated by the release of Bax and c-FLIPL from Ku70, neuroblastoma cells were evaluated for Ku70-c-FLIPL-Bax interactions and Ku70 and c-FLIPL acetylation. Ku70, c-FLIPL, and Bax were identified in a complex at baseline (Fig. 5E), and SAHA treatment resulted in increased acetylation of both Ku70 and c-FLIPL, leading to the release of Bax, which underwent a conformational change to the active form (Fig. 5E, F). HDAC inhibition also increased the binding of ITCH and UBE4B to acetylated-Ku70 and acetylated-c-FLIPL within one hour, followed by reduced binding as the total levels of Ku70 and c-FLIPL decreased (Fig. 5E).

To evaluate the patterns of polyubiquitination of Ku70 and c-FLIPL after HDAC inhibition, neuroblastoma cells were evaluated for Lys48 and Lys63 ubiquitin linkages. HDAC inhibition induced Lys48-linkages on both Ku70 and c-FLIPL that were more notable after proteasomal inhibition (Fig. 5G and Supplemental Figs. 3D and 4). Formation and elongation of polyubiquitin chains on c-FLIPL and Ku70 were observed with increased duration of HDAC inhibition (Fig. 5G, H and Supplemental Fig. 4), suggesting that HDAC inhibition resulted in increased formation and elongation of Lys48/Lys63 branched polyubiquitination of Ku70 and c-FLIPL, leading to the degradation of both c-FLIPL and Ku70.

### UBE4B depletion reduces apoptosis induced by HDAC inhibition via reduced Ku70 and c-FLIPL ubiquitination and proteasomal degradation

**Fig. 6:**
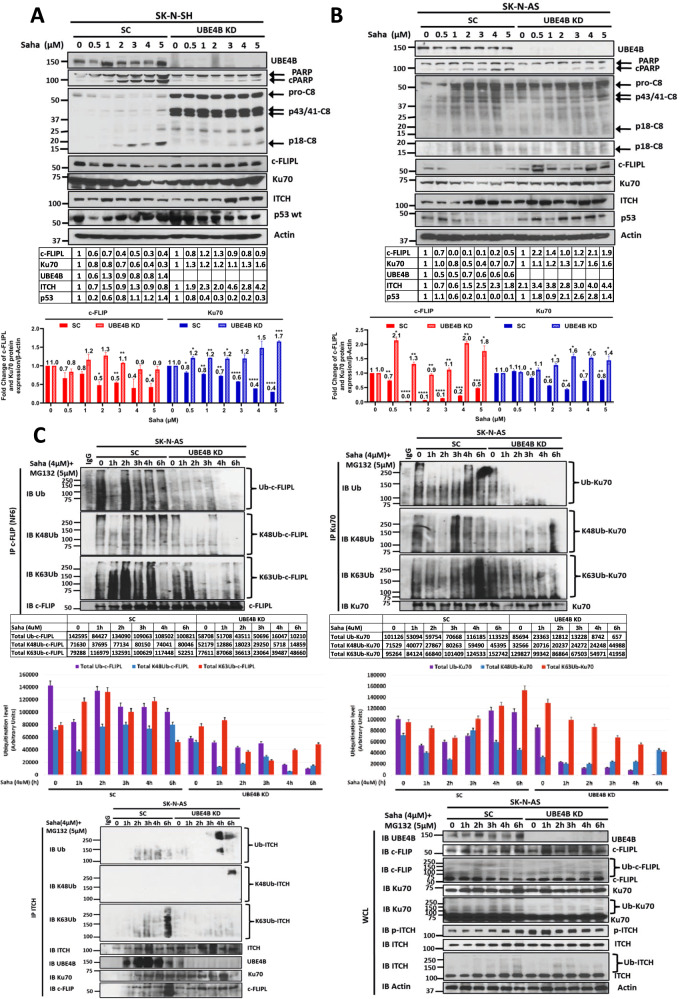

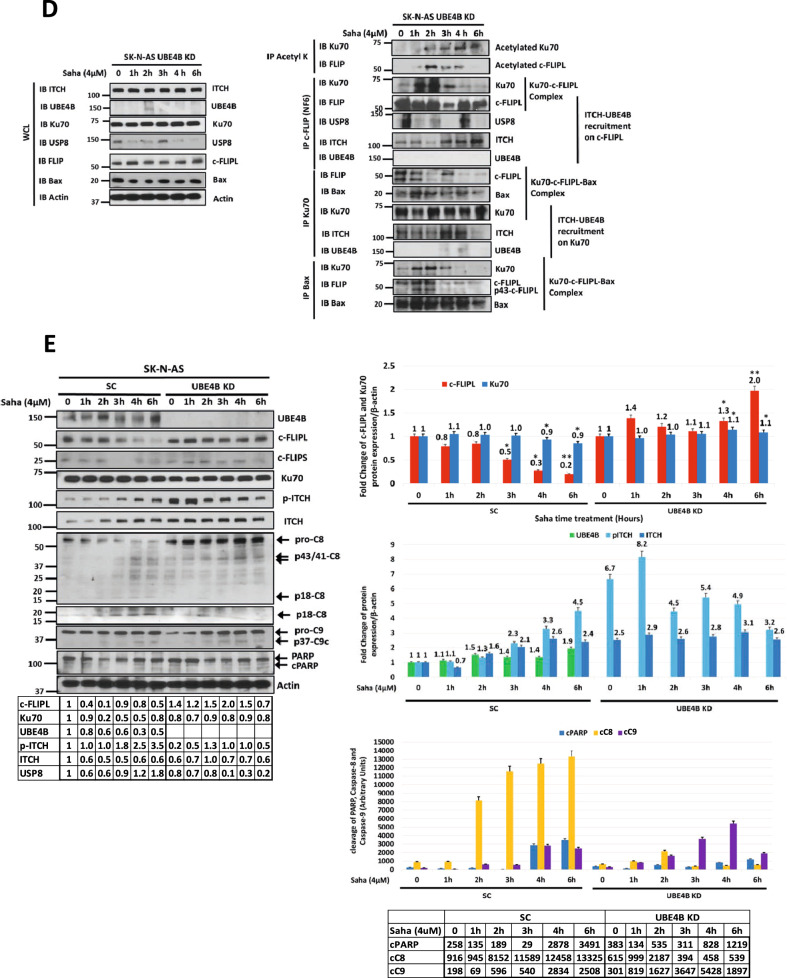
UBE4B depletion reduces apoptosis induced by HDAC inhibition via reduced Ku70 and c-FLIPL ubiquitination and proteasomal degradation. **A**, **B** Whole cell lysates (WCLs) of SK-N-SH (**A**) and SK-N-AS (**B**) neuroblastoma cells expressing scrambled control sgRNA (**A**) or shRNA (**B**) (SC) or *UBE4B* sgRNA (**A**) or shRNA (**B**) for UBE4B knockdown (UBE4B KD) treated for 24 h with increasing doses of vorinostat (Saha) were evaluated by Western blots for UBE4B, PARP and cleaved PARP (cPARP), caspase-8 and cleaved caspase-8 fragments p43/41 and p18, c-FLIPL, Ku70, ITCH, p53, and β-actin (Top). Band intensities for c-FLIPL (red) and Ku70 (dark blue), relative to control lanes and normalized to β-actin expression, were compared across Saha dose levels (Bottom). **C** WCLs of SK-N-AS neuroblastoma cells expressing scrambled control shRNA (SC) or *UBE4B* shRNA for UBE4B knockdown (UBE4B KD) treated with 4μM vorinostat (Saha) and 5μM MG132 for increasing times were evaluated by Western blots for UBE4B, c-FLIPL, Ku70, p-ITCH, ITCH and β-actin (Bottom Right). Immunoprecipitated c-FLIPL (Top Left), Ku70 (Top Right) and ITCH (Bottom Left) were isolated and evaluated by Western blots for ubiquitin, Lys48-linked ubiquitin (K48Ub), Lys63-linked ubiquitin (K63Ub), c-FLIPL, and Ku70 as well as UBE4B and ITCH for immunoprecipitated-ITCH. Lane “0” corresponded to 8 h of MG132 treatment (2 h pretreatment, followed by 6 h of treatment with MG132 plus vehicle (DMSO) used for Saha), while subsequent lanes were from samples treated with MG132 for 2 h, followed by treatment with MG132 plus Saha in DMSO for designated times. Absolute band intensities for total ubiquitinated (violet), K48-ubiquitinated (blue) and K63-ubiquitinated (red) ubiquitin c-FLIPL and Ku70 are shown in the tables and were compared in each cell line at each time point. **D** WCLs of SK-N-AS neuroblastoma cells with depleted UBE4B (UBE4B KD) treated with 4μM vorinostat (Saha) for increasing duration were evaluated by Western blots for ITCH, UBE4B, Ku70, USP8, c-FLIPL, Bax, and β-actin (Left). Immunoprecipitated acetyl-lysine was isolated and evaluated by Western blot for Ku70 and c-FLIPL (Top Right), and immunoprecipitated c-FLIPL, Ku70, and Bax were each isolated and evaluated by Western blots for ITCH, UBE4B, Ku70, USP8, c-FLIPL, Bax and β-actin (Right). **E** WCLs of SK-N-AS neuroblastoma cells expressing scrambled control shRNA (SC) or UBE4B shRNA for UBE4B knockdown (UBE4B KD) treated with 4μM vorinostat (Saha) for increasing times were evaluated by Western blots for UBE4B, c-FLIPL, c-FLIPs, Ku70, phosphorylated ITCH (p-ITCH), total ITCH, caspase-8 and cleaved caspase-8 fragments p43/41 and p18, caspase-9 and cleaved caspase-9 fragment p37, PARP and cleaved PARP (cPARP), and β-actin. Band intensities relative to control lanes are shown in the tables. Band intensities for c-FLIPL (red) and Ku70 (dark blue) relative to β-actin expression were compared over increasing time of Saha treatment (Top Right). Band intensities of UBE4B (green), p-ITCH (light blue), and total ITCH (dark blue) relative to β-actin expression were also compared over increasing time of Saha treatment (Middle Right). Absolute band intensities of cleaved PARP (cPARP) (blue), cleaved caspase-8 fragments (cC8) (yellow) and caspase-9 fragments (cC9) (violet) are shown in the table and were compared in each cell line at each time point (Bottom Right).

To further evaluate the role of UBE4B in the proteasomal degradation of Ku70 and c-FLIPL induced by HDAC inhibition, neuroblastoma cells with depleted UBE4B were analyzed after SAHA treatment. UBE4B depletion abrogated the expected decrease in Ku70, c-FLIPL, and p53 protein levels and dramatically reduced the induction of caspase-8 and PARP cleavage (Fig. 6A, B and Supplemental Fig. 5A) and caspase 3/7 activation (Supplemental Fig. 2). UBE4B depletion resulted in reduced total ubiquitination and Lys48-linked polyubiquitination of Ku70 and c-FLIPL at baseline and after HDAC inhibition (Fig. 6C and Supplemental Figs. 5B and 6), with less reduction in Lys63-linked polyubiquitination of c-FLIPL and Ku70 noted in SK-N-SH cells (Supplemental Figs. 5B and 6), suggesting a crucial role for UBE4B ubiquitin ligase activity in the formation and the elongation of Lys48/Lys63 branched polyubiquitin chains on the c-FLIPL and Ku70 proteins, particularly for creating Lys48-polyubiquitin linkages on existing Lys63-linked ubiquitin residues and for allowing Ku70 and c-FLIP proteasomal degradation. UBE4B depletion also led to reduced ITCH Lys63 ubiquitination (Fig. 6C and Supplemental Fig. 5B), suggesting that the presence of UBE4B in the ITCH-UBE4B complex may be required for ITCH loading with ubiquitin residues on its HECT domain by the E2 enzyme prior to ITCH transferring these residues to Ku70 and c-FLIPL.

While UBE4B depletion did not inhibit acetylation of Ku70 and c-FLIPL induced by HDAC inhibition (Fig. 6D), Ku70 and c-FLIPL remained in a complex with ITCH and Bax for an extended time in UBE4B-depleted cells (Fig. 6D compared to Fig. 5E). The increase in apoptosis induced by HDAC inhibition was also reduced with UBE4B depletion (Fig. 6E). HDAC inhibition induced the release of c-FLIPL from acetylated Ku70 (Fig. 6D) as well as the binding of Ku70 and c-FLIPL to ITCH in the setting of UBE4B depletion, but the degradation of Ku70 and c-FLIPL was significantly reduced (Fig. 6E), suggesting that ITCH is an intermediary binding partner between UBE4B and Ku70 and c-FLIPL and that ITCH E3 ubiquitin ligase activity initiates the process of polyubiquitination of Ku70 and c-FLIPL.

## Discussion

**Fig. 7:**
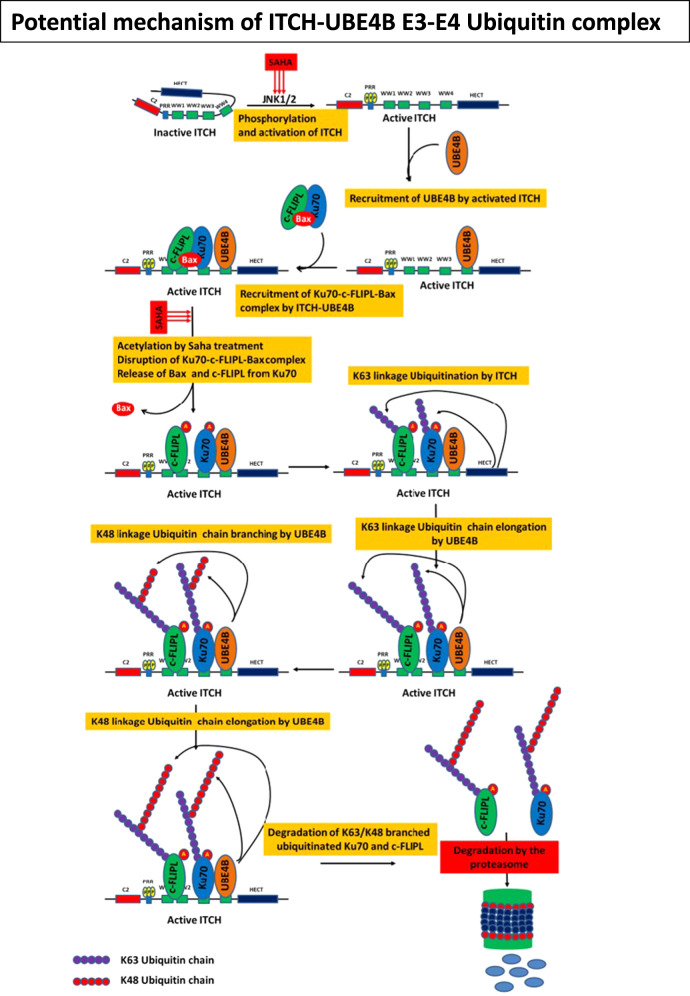
Proposed model of the ITCH-UBE4B E3-E4 ubiquitin ligase complex inducing c-FLIPL and Ku70 Lys48/Lys63 polyubiquitination and proteasomal degradation. We propose that HDAC inhibition induces ITCH activation mediated by JNK phosphorylation, followed by recruitment of UBE4B binding to the WW4 domain of ITCH. The ITCH-UBE4B complex then recruits Ku70 and c-FLIPL binding via other ITCH WW domains. Acetylation of Ku70 disrupts the interaction between Ku70 and Bax, leading to the release of Bax. ITCH then ubiquitinates Ku70 and c-FLIPL, and UBE4B subsequently induces elongation of Lys63 ubiquitin chains, branching via Lys48 ubiquitin linkages, and elongation of Lys48 ubiquitin chains. The polyubiquitinated Ku70 and c-FLIPL are then directed to the proteasome for degradation, leading to activation of caspase 8 and induction of apoptosis.

Children with high-risk neuroblastoma have poor outcomes, and additional understanding of the pathways involved in neuroblastoma pathogenesis will assist in the development of improved therapies. UBE4B is an E3/E4 ubiquitin ligase that catalyzes the transfer of ubiquitin molecules to target proteins, leading to target protein trafficking and degradation. We have previously shown that *UBE4B* gene expression is strongly associated with neuroblastoma patient outcomes, prognostic features, and tumor differentiation [37, 38], and we hypothesized that UBE4B would interact with members of DNA damage and repair and apoptotic pathways, potentially mediating the observed associations between *UBE4B* expression and patient outcomes. Our results have shown for the first time that UBE4B binds to the E3 ubiquitin ligase ITCH via specific WW domains in the activated ITCH protein and that the ITCH-UBE4B E3/E4 ubiquitin ligase complex induced polyubiquitination and proteasomal degradation of both Ku70, a DNA repair subunit protein, and the anti-apoptotic c-FLIPL protein, both novel and previously unknown targets of UBE4B activity. HDAC inhibition induced Ku70 acetylation, leading to release of c-FLIPL and Bax, increased Ku70 and c-FLIPL Lys48/Lys63 polyubiquitination via the ITCH-UBE4B complex, and induction of apoptosis. We have further demonstrated that UBE4B depletion led to reduced polyubiquitination and increased levels of Ku70, c-FLIPL, and p53 and to reduced apoptosis induced by HDAC inhibition via stabilization of c-FLIPL and inhibition of caspase 8 activation. We propose a model where the ITCH-UBE4B E3-E4 ubiquitin complex is induced by HDAC inhibition, leading to c-FLIPL and Ku70 Lys48/Lys63 branched polyubiquitination and proteasomal degradation with subsequent caspase 8-mediated apoptosis (Fig. 7). Our results imply a direct role of the ITCH-UBE4B complex in responses of neuroblastoma cells to HDAC inhibition, suggesting that the ITCH-UBE4B complex plays a critical role in responses of neuroblastoma to therapy and suggesting a potential mechanism underlying the significant association of *UBE4B* expression with neuroblastoma patient outcomes.

We previously identified a direct physical interaction between UBE4B and the hepatocyte growth factor-regulated tyrosine kinase substrate (Hrs) protein [39], a key regulator of growth factor receptor (GFR) endosomal trafficking [64, 65]. Recruitment of UBE4B to endosomal membranes is dependent on binding with Hrs, and we demonstrated that UBE4B ubiquitin ligase activity is required for appropriate EGFR trafficking and lysosomal degradation in neuroblastoma cells [39]. UBE4B has also been shown to ubiquitinate cytosolic proteins, leading to proteasomal degradation, and previously identified direct cytosolic UBE4B targets include p53, FEZ1, ataxin-3, TTK, PRMT1, MAPK3, OTUB1, and PGAM5 [40–44]. UBE4B has also been shown to interact with other proteins, including VCP and Hsp90 [31, 66, 67]. These links between UBE4B ubiquitin ligase activity and GFR trafficking and additional signaling pathways therefore suggest potential alternative mechanisms that could also mediate the observed associations between *UBE4B* gene expression and neuroblastoma patient outcomes.

Polyubiquitination of target proteins has been shown to direct proteins for proteasomal degradation, while mono- and multi-monoubiquitination of proteins can serve as signals for other intracellular protein sorting events [25]. Polyubiquitination reactions are formed on the Lys48 residue, and Lys48-linked ubiquitin chains are the most prevalent proteasome-targeting signal, although other ubiquitin modifications such as Lys11- or Lys29-linked chains as well as multi-monoubiquitination also function as proteasomal signals [22, 68]. Modifications involving Lys63-linked chains are more typically involved in regulation of inflammatory signal transduction, DNA repair, endocytosis, and selective autophagy [69, 70]. However, recent studies have shown that Lys63 ubiquitination triggers proteasome-mediated degradation by serving as a “seed” for Lys48/Lys63 branched ubiquitin chains [69–73], demonstrating that a non-degradative ubiquitin chain can be converted to a signal to induce proteasomal degradation through induction of branched ubiquitin chains. In *S. cerevisiae*, Ufd2p (i.e., UBE4B) catalyzes Lys48-linked polyubiquitination on Lys29-linked ubiquitin chains assembled by the ubiquitin ligase Ufd4p, a HECT-domain containing E3 ubiquitin ligase, resulting in substrate proteasomal degradation [74]. Prior reports have suggested that ITCH-mediated Lys63 ubiquitination plays a role in proteasome-mediated substrate degradation by serving as a “seed” for Lys48/Lys63 branched ubiquitin chains, with ITCH-dependent Lys63 ubiquitination of the proapoptotic regulator TXNIP inducing subsequent generation of Lys48/Lys63 branched chains via UBR5, leading to TXNIP degradation [73]. Based on these prior reports and our results, we speculate that ITCH induces the branching of the first short Lys63-linked ubiquitin chain on Ku70 and c-FLIPL, and UBE4B then acts as an E4 ubiquitin ligase to induce the polymerization of longer Lys48- and Ly63-branched polyubiquitin chains on Ku70, c-FLIPL, p53, and likely other UBE4B targets and acts also as a specific E3 ubiquitin ligase to induce Lys48 polyubiquitin branching on the elongated Lys63 polyubiquitin chain initiated by ITCH on Ku70, c-FLIPL and p53.

Our results demonstrate for the first time that HDAC inhibition induces Lys48/Lys63 branched polyubiquitination on Ku70 and c-FLIPL via the ITCH-UBE4B complex with induction of neuroblastoma cell apoptosis. ITCH is a NEDD4 family ubiquitin ligase with a C-terminal HECT domain for E3 ligase function and WW domains for substrate binding. ITCH has previously been shown to regulate several signaling pathways through polyubiquitination, with Lys48, Lys63, Lys27, Lys29, and Lys33 linkages to its target proteins [75], but the roles of many of these linkages remain poorly understood. ITCH is involved in the control of inflammatory signaling pathways and also has a multifaceted role in human cancers, acting in context-dependent fashion as either an oncogene or tumor suppressor. ITCH has been shown to ubiquitinate over 50 target proteins [76–85], and ITCH depletion leads to increased p73 levels and increased sensitivity to radiation therapy in neuroblastoma cells [76]. ITCH has also been shown to participate in endocytic sorting of membrane proteins by promoting endocytic degradation of endophilin [86] as well as CXCR4 [87, 88] and other membrane proteins. Similarly, ITCH mediates ubiquitination of the Hrs protein [87], suggesting further interactions between UBE4B and ITCH may occur on the endosomal membrane surface and contribute to the associations of *UBE4B* expression with neuroblastoma patient outcomes.

Our results have identified and confirmed Ku70 and c-FLIPL as new targets of UBE4B ubiquitin ligase activity through the ITCH-UBE4B complex. Ku70 is a single-stranded DNA-dependent ATP-dependent helicase that is involved in DNA non-homologous end joining (NHEJ) required for double-strand break repair and V(D)J recombination. Ku70 acetylation has been reported to lead to the release of c-FLIPL and Bax, allowing for c-FLIPL degradation and Bax activation. Ku70 and Bax have previously been shown to interact in neuroblastoma cells, with Ku70 acetylation regulated by the CBP acetyl transferase and HDAC6 leading to Bax release and induction of apoptosis [45, 46]. Ku70 also sequesters and stabilizes Bax via Ku70-mediated deubiquitination and maintains Bax in an inactive conformation [45, 46, 49–51, 89]. However, the molecular mechanisms underlying these interactions have not previously been described.

HDACs play an important role in regulating gene expression through effects on chromatin structure. Dysregulation of HDAC activity has been found to promote tumor development and progression in many tumor types, and HDAC inhibitors induce apoptosis in many types of cancer cells [90–92]. HDAC expression has been shown to be associated with neuroblastoma treatment resistance [93–95], and HDAC inhibitors have been previously shown to inhibit neuroblastoma cell growth and to induce differentiation, apoptosis and cell cycle arrest in neuroblastoma tumors [60]. Treatment with the HDAC inhibitor vorinostat (SAHA) also induced neuroblastoma cell apoptosis and increased chemotherapy sensitivity [50, 51] and demonstrated synergistic efficacy with both radiation and retinoic acid [50, 96, 97], demonstrating the clinical potential of vorinostat for the treatment of neuroblastoma. However, the mechanisms by which HDAC inhibition induces apoptosis in neuroblastoma cells are poorly understood and likely involve restoring expression of epigenetically repressed tumor suppressor genes as well as restoring the acetylation of other non-histone proteins. Our results demonstrating effects of HDAC inhibitors on Ku70, leading to Ku70 and c-FLIPL polyubiquitination and degradation and neuroblastoma cell apoptosis, are consistent with these prior findings.

Apoptosis is mediated by extrinsic and intrinsic pathways, and HDAC inhibitors can induce both extrinsic and intrinsic apoptotic pathways in cancer cells. The extrinsic pathway is initiated by the binding of death receptors to specific ligands such as Fas ligand (FasL) and TNF-related apoptosis-inducing ligand (TRAIL). The adapter protein Fas-associated death domain protein (FADD) and caspase-8/−10 are recruited and activated to trigger apoptosis [98]. The FADD-like interleukin 1-converting enzyme (FLICE) inhibitory protein c-FLIPL, which competitively binds to procaspase-8 and inhibits caspase-8 activity, is a negative regulator of this extrinsic apoptosis pathway [99]. The intrinsic pathway is controlled mainly by the pro- and anti-apoptotic Bcl-2 family proteins and the effector proteins Bak and Bax, which can oligomerize with truncated Bid and form pores in the outer mitochondrial membrane, releasing cytochrome c and Smac/Diablo into the cytoplasm, leading to the activation of caspase-9 and caspase-3 and induction of apoptosis [100]. Our results therefore demonstrate novel linkages of the ITCH-UBE4B complex to both the intrinsic and extrinsic apoptotic pathways, with Ku70 having a role in triggering both the intrinsic pathway via Bax activation and the extrinsic pathway via c-FLIPL degradation.

We believe that neuroblastoma tumorigenesis is promoted by a coordinated network of intracellular signaling pathways whose activities are altered by reduced *UBE4B* expression and loss of UBE4B ubiquitin ligase activity in high-risk neuroblastoma tumors and that these UBE4B-mediated pathways directly impact neuroblastoma patient outcomes. Our results have identified novel interactions and novel targets for UBE4B ubiquitin ligase activity and a direct role of the ITCH-UBE4B E3-E4 ubiquitin ligase complex in neuroblastoma cell responses to HDAC inhibition. Our results also suggest that HDAC inhibition induces ITCH activation and formation of ITCH-UBE4B ubiquitin ligase complex that recruits Ku70 and c-FLIPL for polyubiquitination and degradation, leading to induction of apoptosis and suggesting that the association of UBE4B expression with neuroblastoma patient outcomes may be mediated by effects on responses of patient tumors to therapy via the ITCH-UBE4B complex. The combined associations of UBE4B expression and UBE4B-mediated intracellular signaling pathway activity with patient outcomes suggests that these pathways play critical roles in neuroblastoma pathogenesis and represent potential therapeutic targets. Increased understanding of UBE4B targets and downstream signaling pathways will help identify the functional links between *UBE4B* expression and patient outcomes and identify novel targets for the development of new treatment combinations for children with neuroblastoma.

### Reporting summary

Further information on research design is available in the Nature Portfolio Reporting Summary linked to this article.

### Supplementary information

Author Reporting Summary checklist

Supplemental Tables 1, 2, and 3

Supplemental Figure 1

Supplemental Figure 2

Supplemental Figure 3

Supplemental Figure 4

Supplemental Figure 5

Supplemental Figure 6

Supplemental methods and legends

Uncropped Western blots

## Data Availability

Data sharing is not applicable to this article as no datasets were generated during the current study. All data generated during this study are included in this published article and are available from the lead contact upon request.
