# Supplementary material for: UBE4B interacts with the ITCH E3 ubiquitin ligase to induce Ku70 and c-FLIPL polyubiquitination and enhanced neuroblastoma apoptosis
Source: Cell Death Dis. 2023 Nov 13;14(11):739. doi: 10.1038/s41419-023-06252-7 (PMC10643674; doi:10.1038/s41419-023-06252-7)
Supplement: Supplementary file 2 — Supplemental Tables 1, 2, and 3 [file 41419_2023_6252_MOESM2_ESM.pdf]

Supplemental Table 1 – UBE4B shRNA Sequences

Name (Sigma ID #)	shRNA UBE4B DNA sequence
**TRCN0000338295**	CCGGGCCTAGTTGCCGTCGCTATATCTCGAGATATAGCGACGGCAACTAGGCTTTTTG
**TRCN0000007548**	CCGGGCAGGGATCAAATCCACAATACTCGAGTATTGTGGATTTGATCCCTGCTTTTT
**TRCN0000350907**	CCGGGCAGGGATCAAATCCACAATACTCGAGTATTGTGGATTTGATCCCTGCTTTTTG
**TRCN0000338354**	CCGGGAAGTGTTCAAGCAGATATTTCTCGAGAAATATCTGCTTGAACACTTCTTTTTG

Supplemental Table 2 - UBE4B and Scrambled Control sgDNA sequences

Name (Abcam ID #)	sgRNA UBE4B sequences
**UBE4B sgRNA CRISPR/Cas9 All-in-One Lentivector set (Human)<br><br>(K257610 5)**	37 CGCCTTGCACGACTTGCTGG<br><br>836 AGAGCTCCAGAAACTGGGAG<br><br>950 GCTTCCCGCAGCAGTTCCAG
**Name (Abcam ID #)**	sgRNA Scramble Control sequence
**Scrambled sgRNA CRISPR/Cas9 All-in-One Lentivector<br><br>(K010)**	GCACTCACATCGCTACATCA

Supplemental Table 3 - Antibodies used in this study

Name	Antibody Type	Manufacturer	Reference	Clone	Dilution used
UBE4B	Rabbit Polyclonal	Bethyl Laboratories	A301-123A		1/10000 (1µg)
Ku70	Rabbit Polyclonal	Novus Biological	NBP2-66920		1/10000 (1µg)
Ku70	Mouse Monoclonal	Novus Biological	NB100-1915		1/10000 (1µg)
c-FLIPL	Mouse Monoclonal	ThermoFisher	50-168-7227	NF6	1/1000
ITCH/AIP4	Mouse Monoclonal	BD Biosciences	611199		1/5000
p-ITCH (Thr222)	Rabbit Polyclonal	Milipore/Sigma-Aldrich	AB10050MI		1/10000
USP8	Rabbit Polyclonal	Bethyl Laboratories	A302-929A		1/5000
PARP	Rabbit Polyclonal	Cell signaling Technology	9542S		1/1000
Caspase 8	Mouse Monoclonal	Cell signaling Technology	9746S	1C12	1/1000
Cleaved Caspase-8	Mouse Monoclonal	Cell signaling Technology	9496S	18C8	1/1000
Caspase-9	Rabbit Polyclonal	Cell signaling Technology	9502S		1/1000
Caspase-3	Rabbit Polyclonal	Cell signaling Technology	9262S	8G10	1/1000
Cleaved Caspase-3	Rabbit Polyclonal	Cell signaling Technology	9661S		1/1000
XIAP	Rabbit Polyclonal	Cell signaling Technology	14334S	D2Z8W	1/1000
c-IAP2	Rabbit Polyclonal	Cell signaling Technology	3130S	58C7	1/1000
FAS/CD95	Rabbit Polyclonal	Cell signaling Technology	4233S	C18C12	1/1000
DR4	Rabbit Polyclonal	Cell signaling Technology	42533S	D9S1R	1/1000
DR5	Rabbit Polyclonal	Cell signaling Technology	8074S	D4E9	1/1000
BIM	Rabbit Polyclonal	Cell signaling Technology	2933S	C34C5	1/1000
BID	Rabbit Polyclonal	Cell signaling Technology	2002S		1/1000
USP9X	Rabbit Polyclonal	Cell signaling Technology	14898S	D4Y7W	1/1000
p53	Rabbit Polyclonal	Cell signaling Technology	2527S	7F5	1/1000
p53	Mouse Monoclonal	Cell signaling Technology	1832S	DO1	1/1000
Ubiquitin	Rabbit Polyclonal	Cell signaling Technology	3933S		1/1000
K48-linkage polyubiquitin	Rabbit Polyclonal	Cell signaling Technology	8081S	D9D5	1/1000
K63-linkage polyubiquitin	Rabbit Polyclonal	Cell signaling Technology	5621S	D7A11	1/1000
Bax	Rabbit Polyclonal	Milipore/Sigma-Aldrich	ABC11		1/1000
Bax	Mouse Monoclonal	Santa Cruz Biotechnoloy	sc-23959	6A7	1/1000
c-Myc	Mouse Monoclonal	Santa Cruz Biotechnoloy	sc-40		1/1000
Flag	Mouse Monoclonal	Sigma-Aldrich	F1804		1/1000
control IgG1	Mouse Monoclonal	Cell signaling Technology	5415S	G3A1	
control IgG1	Rabbit Polyclonal	Cell signaling Technology	3900S	DA1E	
β-Actin	Mouse Monoclonal	Sigma-Aldrich	AS316		1/20000
HRP-conjugated Goat anti-rabbit IgG (H+L)	Secondary antibody	Biorad	170-6515		1/5000
HRP-conjugated Goat anti-mouse IgG (H+L)	Secondary antibody	Biorad	170-6516		1/5000
