# Supplementary material for: UBE4B interacts with the ITCH E3 ubiquitin ligase to induce Ku70 and c-FLIPL polyubiquitination and enhanced neuroblastoma apoptosis
Source: Cell Death Dis. 2023 Nov 13;14(11):739. doi: 10.1038/s41419-023-06252-7 (PMC10643674; doi:10.1038/s41419-023-06252-7)
Supplement: Supplementary file 4 — Supplemental Figure 2 [file 41419_2023_6252_MOESM4_ESM.pdf]

Supplemental Figure 2 - UBE4B depletion reduces HDAC inhibitor-induced neuroblastoma cell caspase 3/7 activation

A

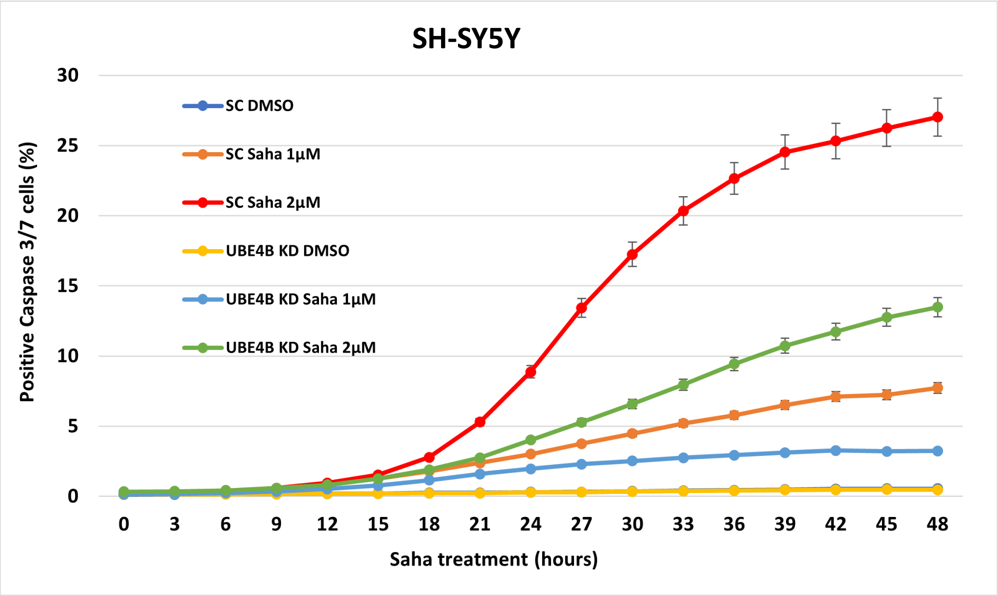

B

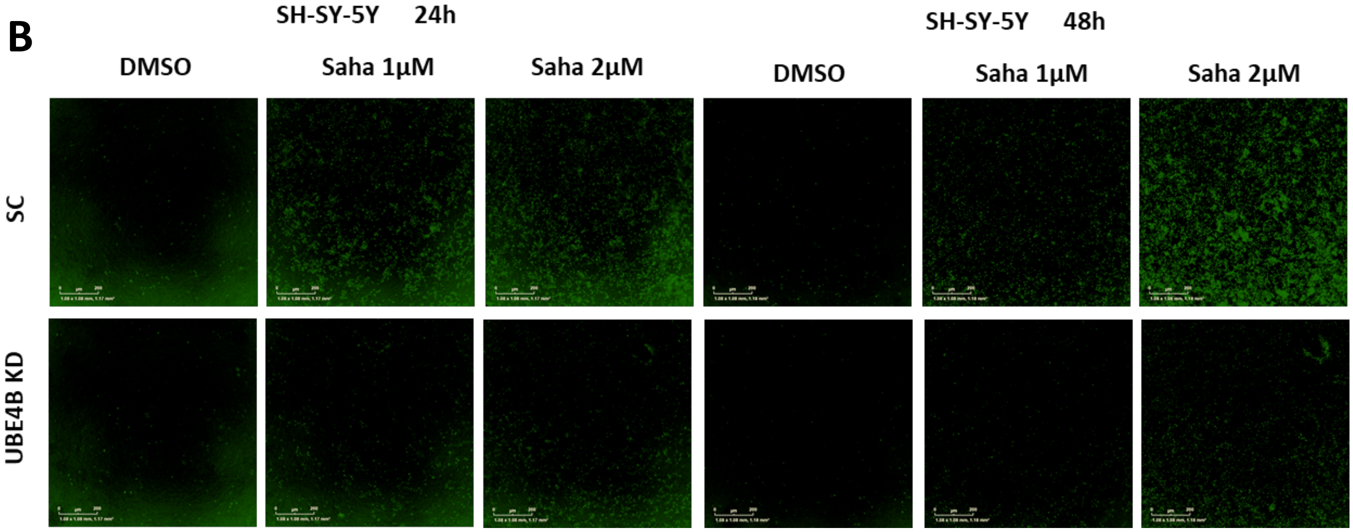

C

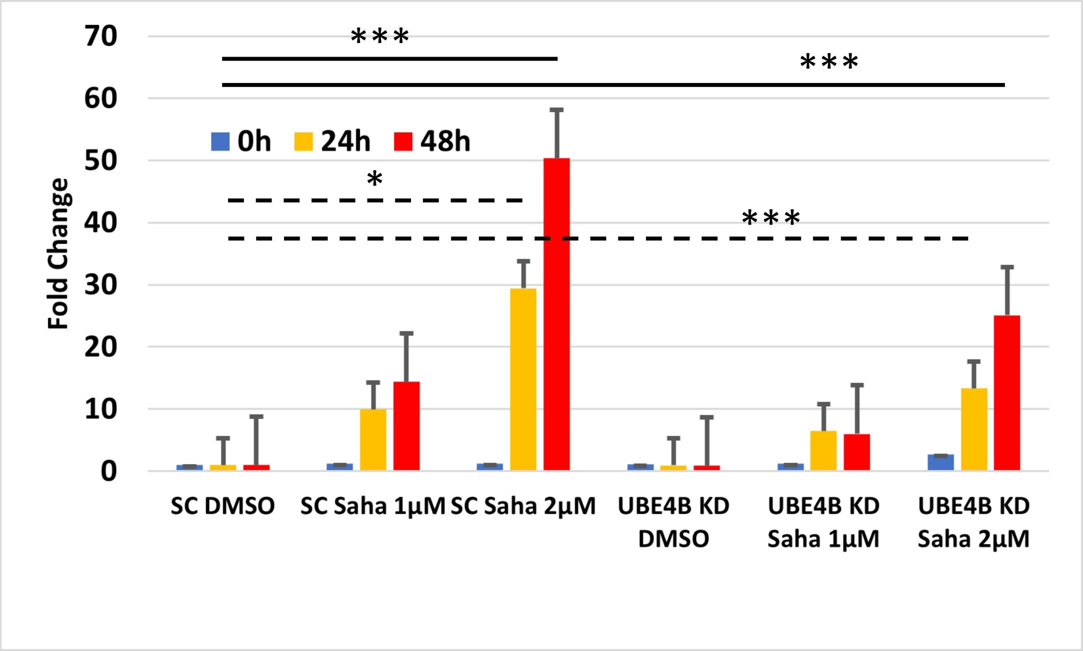

D

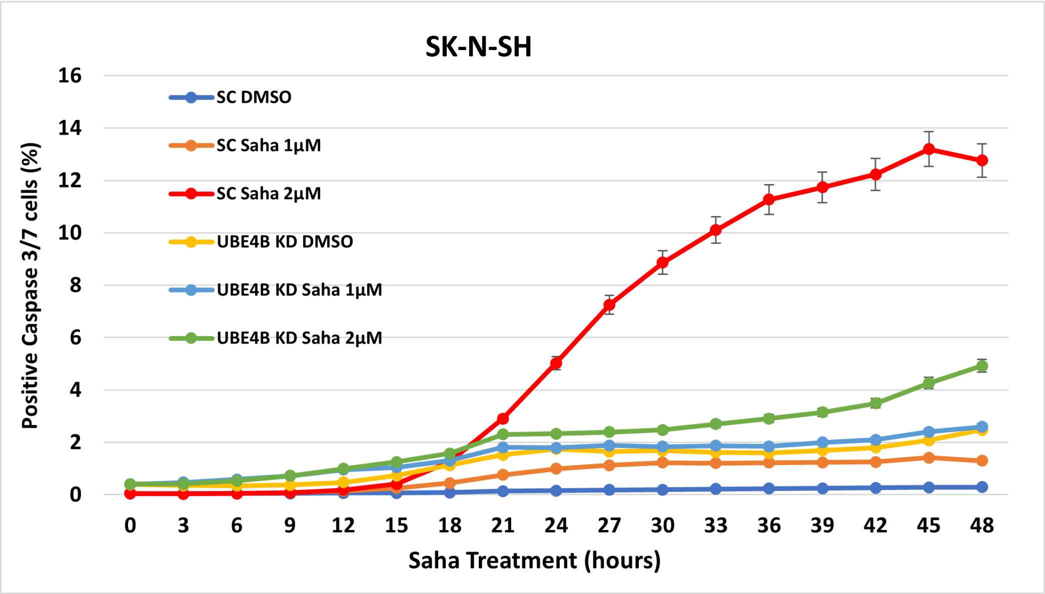
SK-N-SH
Positive Caspase 3/7 cells (%)
16
14
12
10
8
6
4
2
0
SC DMSO
SC Saha 1μM
SC Saha 2μM
UBE4B KD DMSO
UBE4B KD Saha 1μM
UBE4B KD Saha 2μM
0
3
6
9
12
15
18
21
24
27
30
33
36
39
42
45
48
Saha Treatment (hours)

E

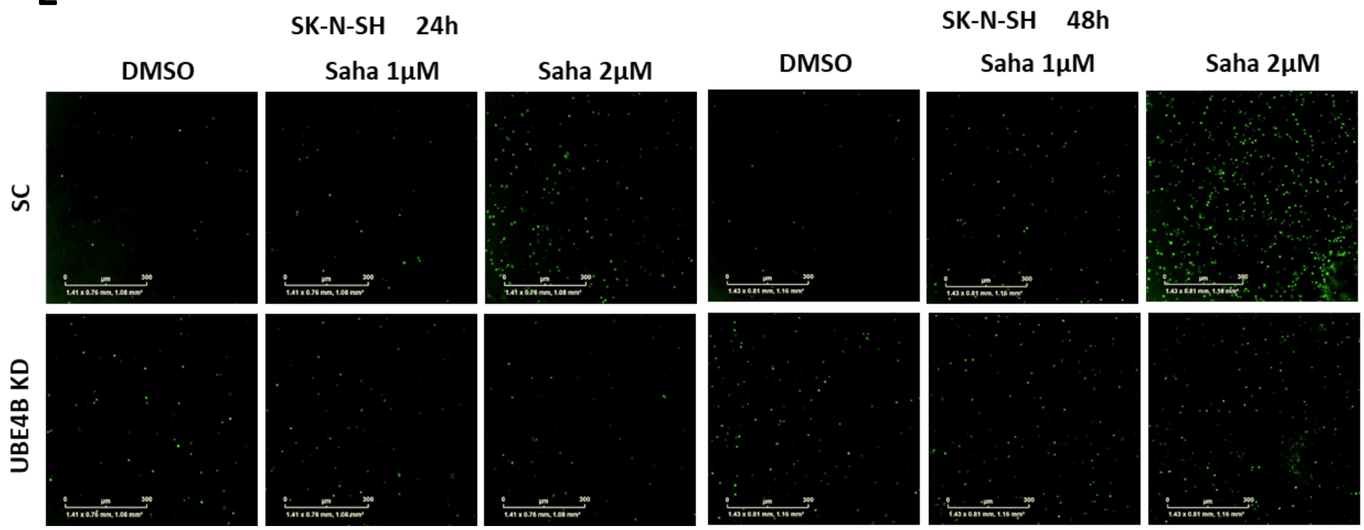
SK-N-SH 24h
SK-N-SH 48h
DMSO
Saha 1μM
Saha 2μM
DMSO
Saha 1μM
Saha 2μM
SC
UBE4B KD

F

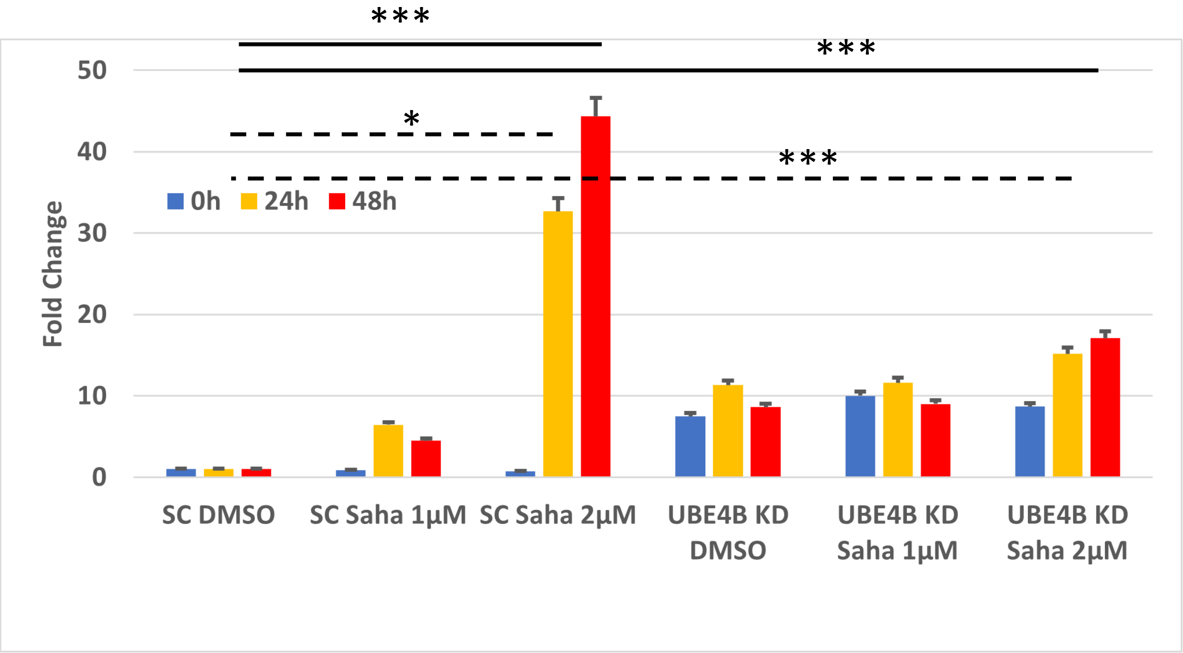
***
***
*
***
50
40
30
20
10
0
Fold Change
0h
24h
48h
SC DMSO
SC Saha 1μM
SC Saha 2μM
UBE4B KD DMSO
UBE4B KD Saha 1μM
UBE4B KD Saha 2μM

G

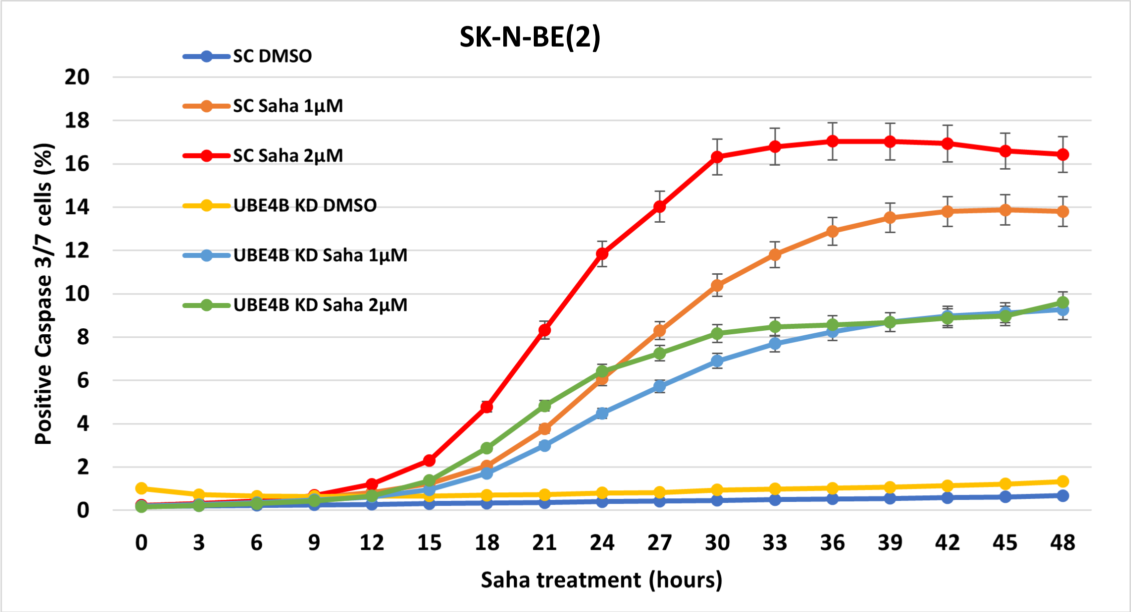
SK-N-BE(2)
SC DMSO
SC Saha 1µM
SC Saha 2µM
UBE4B KD DMSO
UBE4B KD Saha 1µM
UBE4B KD Saha 2µM
Positive Caspase 3/7 cells (%)
0
2
4
6
8
10
12
14
16
18
20
0 3 6 9 12 15 18 21 24 27 30 33 36 39 42 45 48
Saha treatment (hours)

H

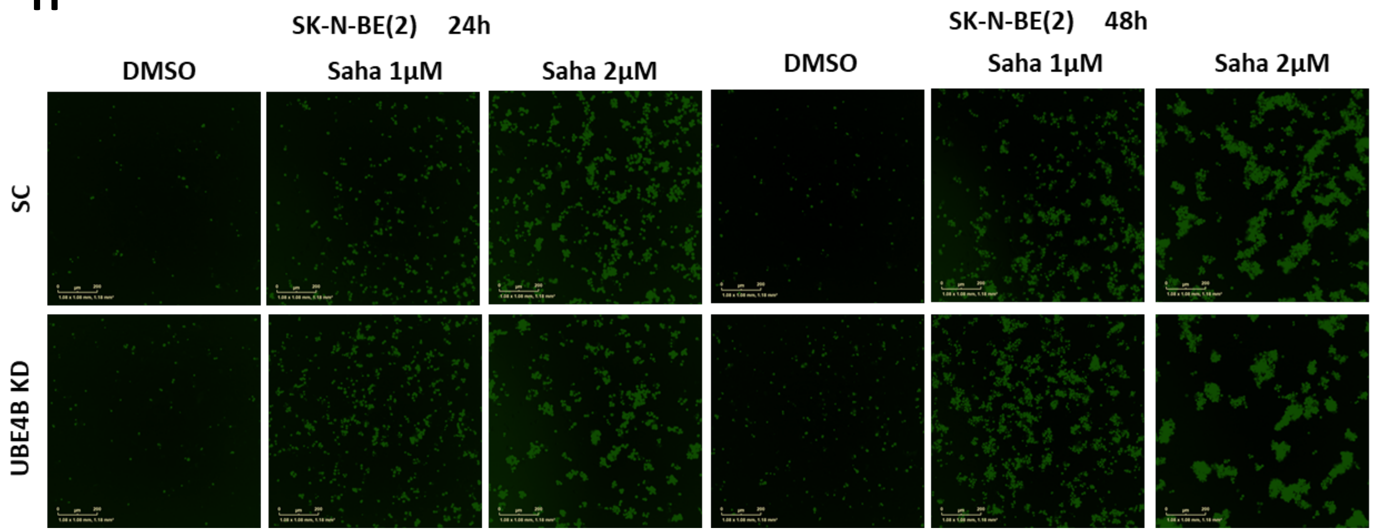
SK-N-BE(2) 24h
SK-N-BE(2) 48h
DMSO
Saha 1µM
Saha 2µM
DMSO
Saha 1µM
Saha 2µM
SC
UBE4B KD

I

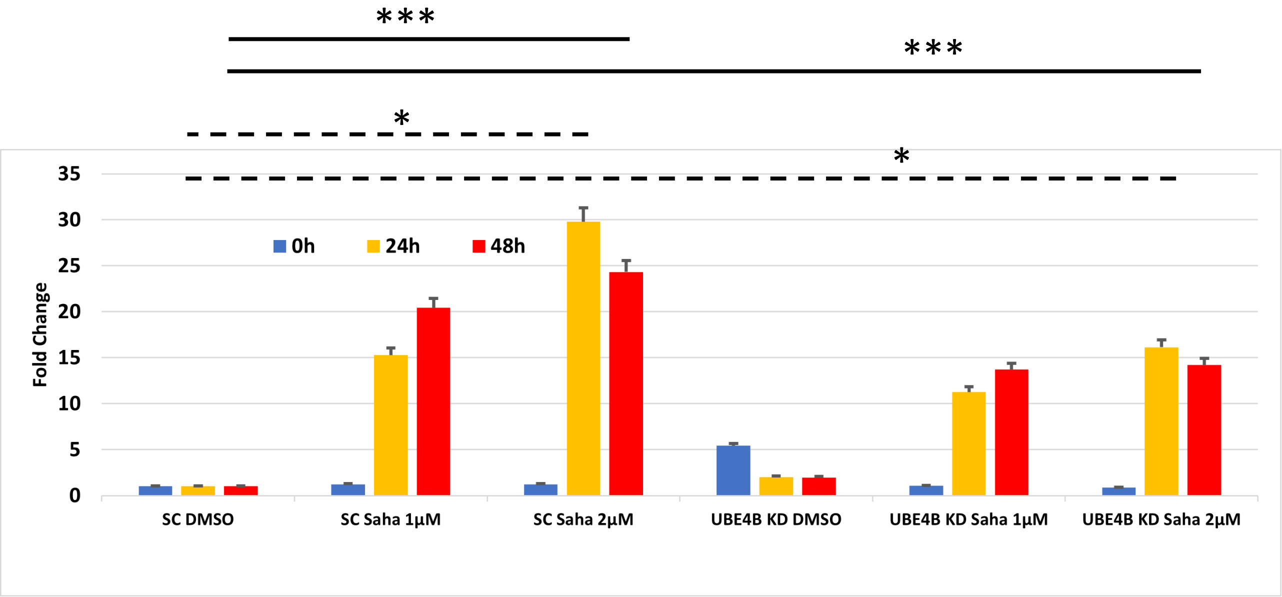
***
***
*
*
0h
24h
48h
Fold Change
0
5
10
15
20
25
30
35
SC DMSO
SC Saha 1µM
SC Saha 2µM
UBE4B KD DMSO
UBE4B KD Saha 1µM
UBE4B KD Saha 2µM

J

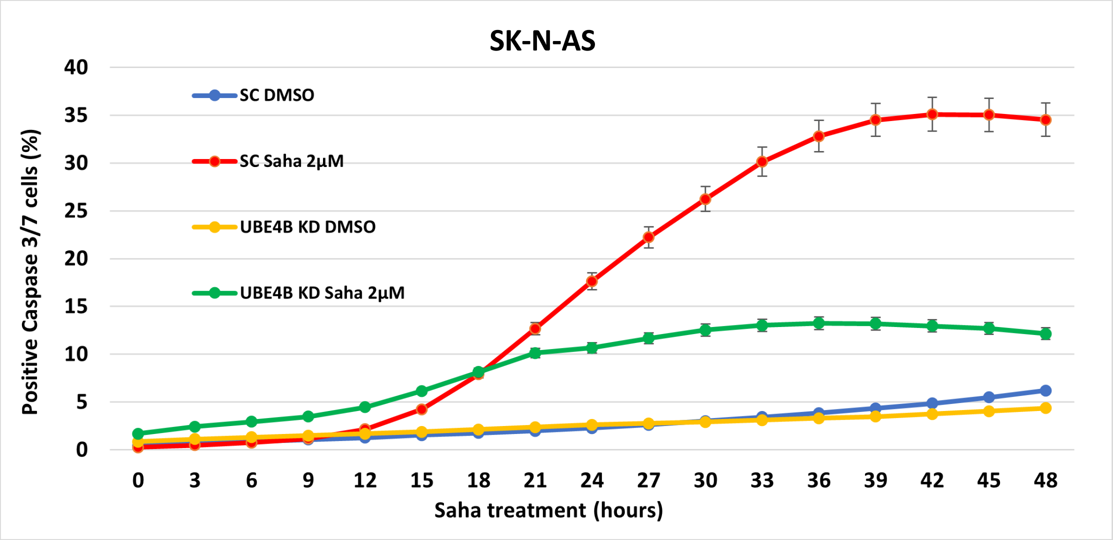

SK-N-AS
SC DMSO
SC Saha 2µM
UBE4B KD DMSO
UBE4B KD Saha 2µM
Positive Caspase 3/7 cells (%)
40
35
30
25
20
15
10
5
0
0 3 6 9 12 15 18 21 24 27 30 33 36 39 42 45 48
Saha treatment (hours)

K

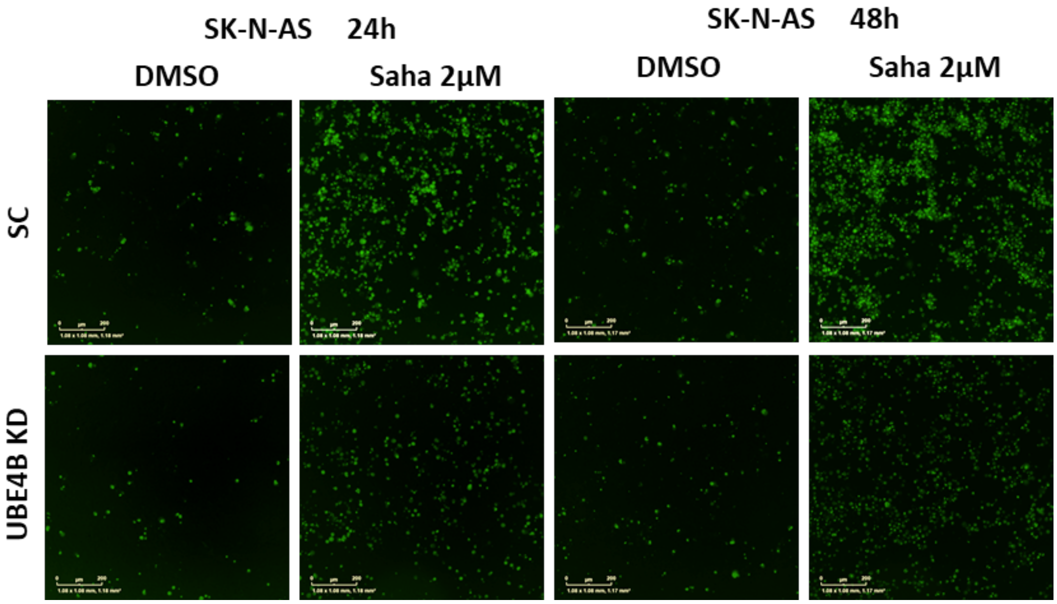

SK-N-AS 24h
SK-N-AS 48h
DMSO
Saha 2µM
DMSO
Saha 2µM
SC
UBE4B KD

L

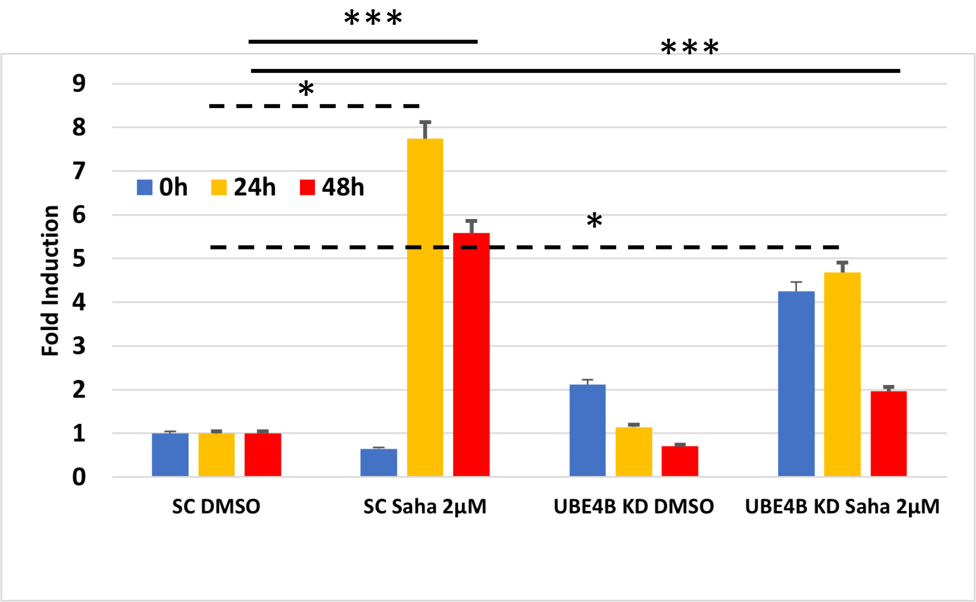

***
***
*
*
0h
24h
48h
Fold Induction
9
8
7
6
5
4
3
2
1
0
SC DMSO
SC Saha 2µM
UBE4B KD DMSO
UBE4B KD Saha 2µM
