# Supplementary material for: UBE4B interacts with the ITCH E3 ubiquitin ligase to induce Ku70 and c-FLIPL polyubiquitination and enhanced neuroblastoma apoptosis
Source: Cell Death Dis. 2023 Nov 13;14(11):739. doi: 10.1038/s41419-023-06252-7 (PMC10643674; doi:10.1038/s41419-023-06252-7)

Supplemental Figure 3 – HDAC inhibition induces Ku70 and c-FLIPL acetylation and Lys48/Lys63 branched polyubiquitination via the ITCH-UBE4B complex

A

SH-SY5Y

Saha (µM)	0	0.5	1	2	3	4	5
cPARP	670	20842	14619	12683	17387	22433	23306
cC8	1404	6134	14375	16743	13876	14638	28280

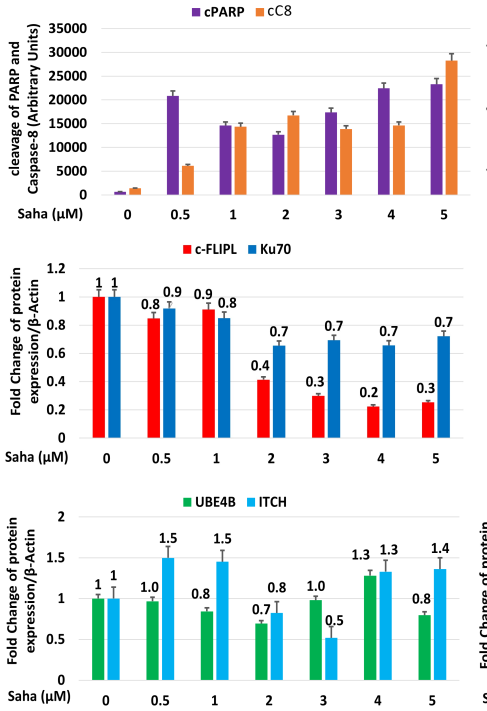

SK-N-BE(2)

Saha (µM)	0	0.5	1	2	3	4	5
cPARP	1416	5561	7321	13199	14852	20757	31839
cC8	696	1970	3858	14618	19086	23192	38316

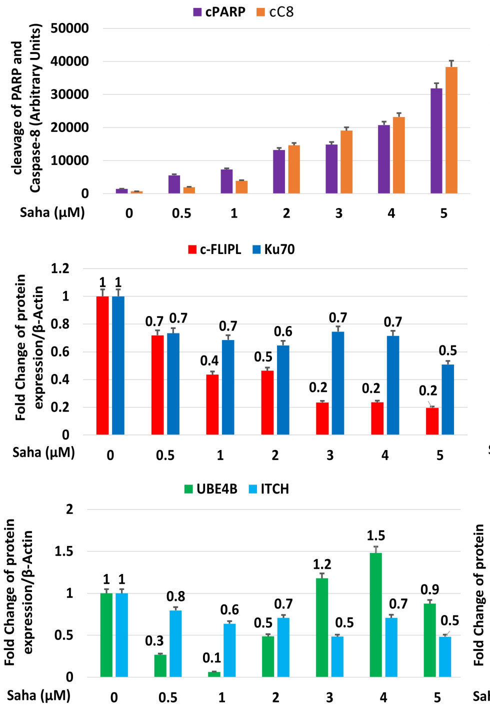

SK-N-AS UBE4B

Saha (µM)	0	0.5	1	2	3	4	5
cPARP	7053	6446	31671	41139	35118	34834	32694
cC8	1102	3024	7431	28102	19824	22355	22723

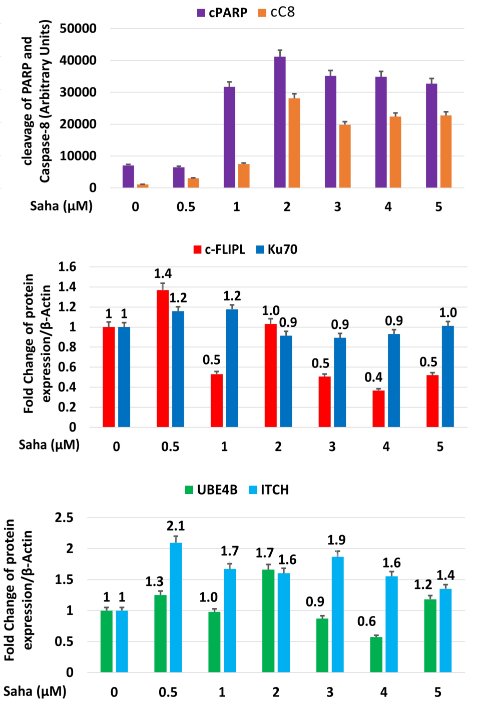

SK-N-SH

Saha (µM)	0	0.5	1	2	3	4	5
cPARP	105	506	6752	22085	35197	29024	40197
cC8	243	602	5455	26424	38269	36884	53867

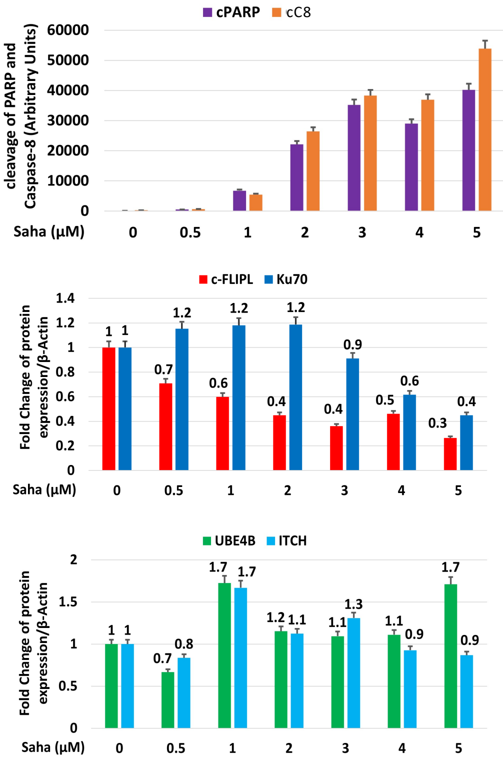

SH-EP

Saha (µM)	0	0.5	1	2	3	4	5
cPARP	4284	18822	18246	19145	11667	26054	11759
cC8	3846	19298	27928	21272	14167	31668	24922

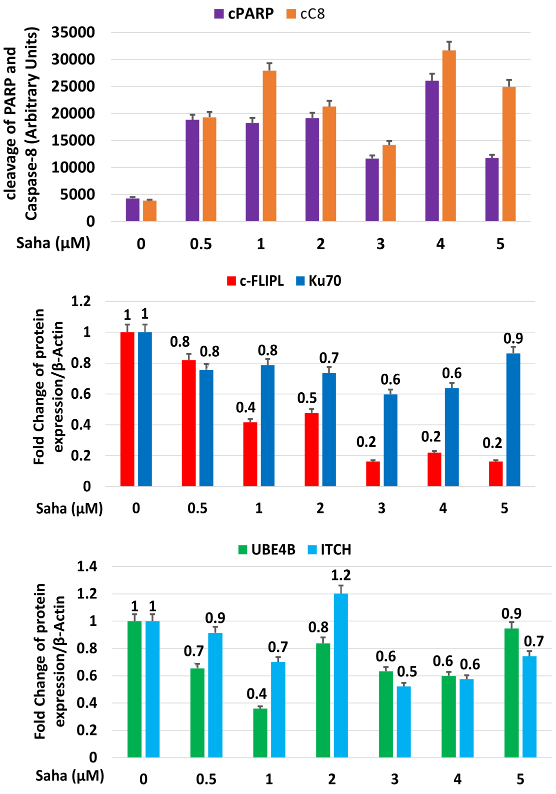

B

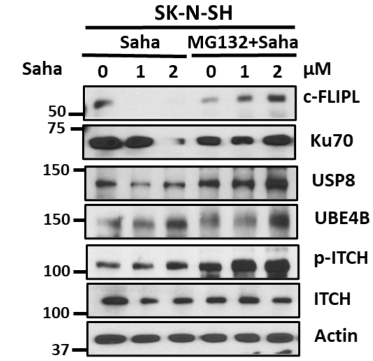

c-FLIPL	1	0.0	0.0	0.5	0.9	1.1
Ku70	1	0.9	0.2	0.7	0.6	0.9
USP8	1	0.7	1.6	2.9	3.8	4.4
UBE4B	1	1.5	2.3	2.0	2.0	2.7
p-ITCH	1	1.2	1.7	2.7	3.9	3.5
ITCH	1	0.6	0.7	0.6	0.8	0.6

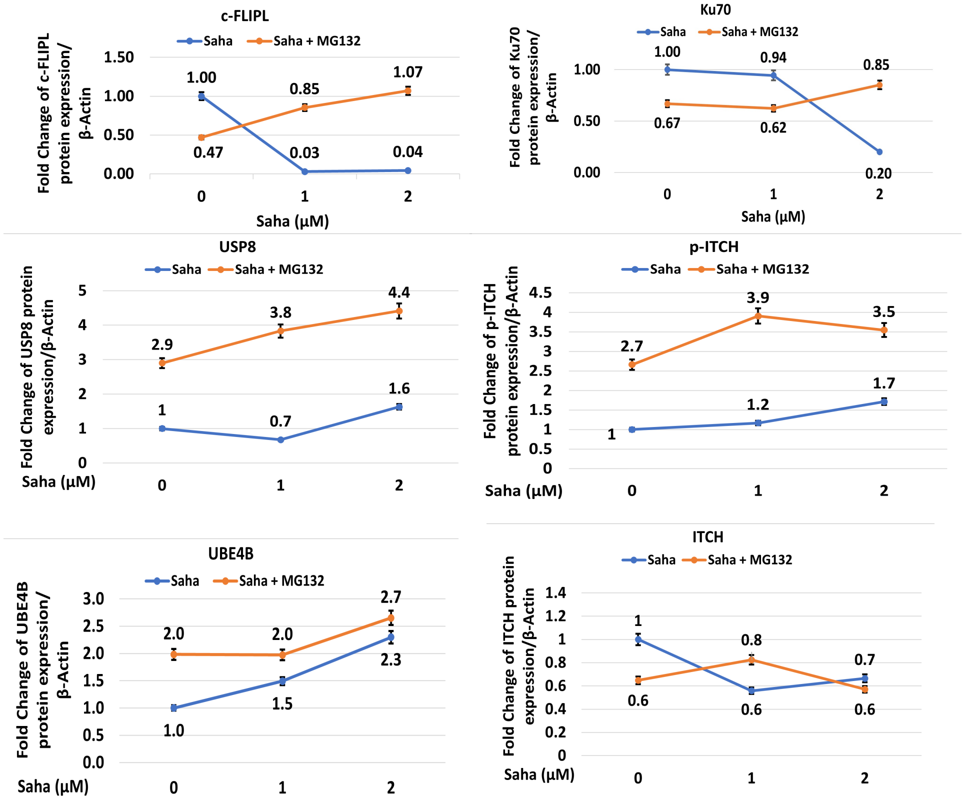

C

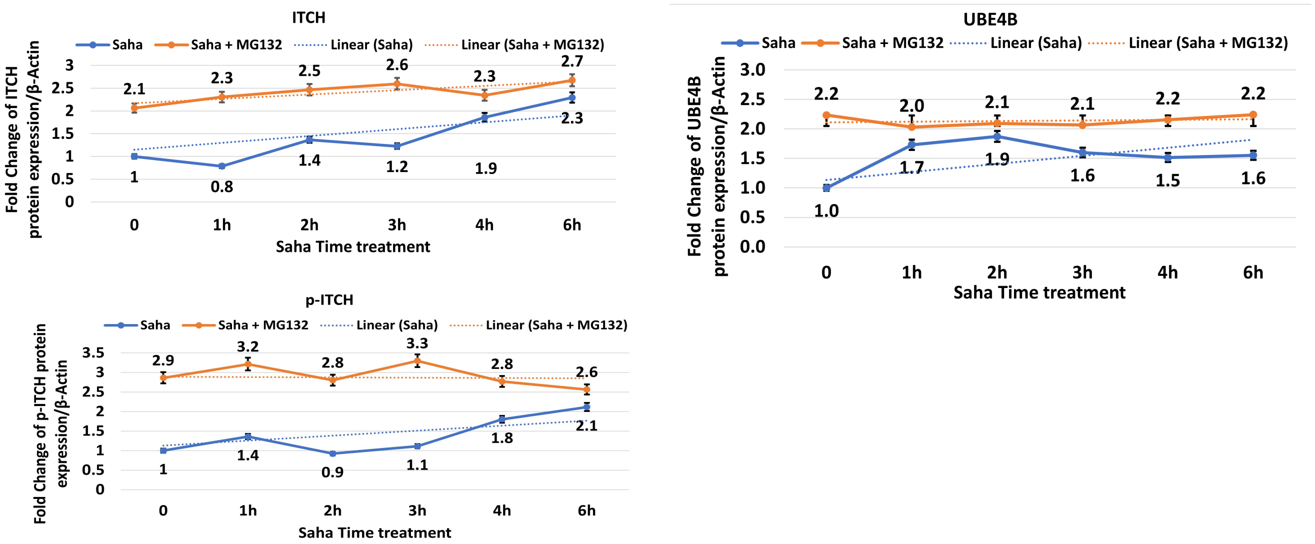

D

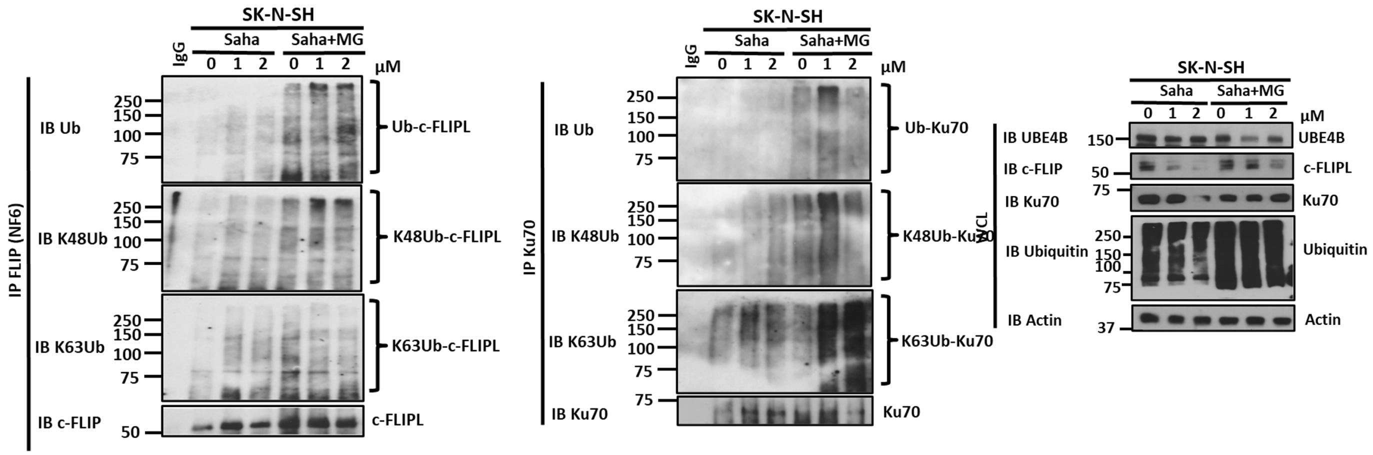

				+MG132		
Saha (μM)	0	1	2	0	1	2
Total Ub-c-FLIPL	10053	14720	15879	34188	39616	47208
Total K48Ub-c-FLIPL	18569	29700	26700	37730	40513	41413
Total K63Ub-c-FLIPL	4898	13008	13661	22311	16477	15374

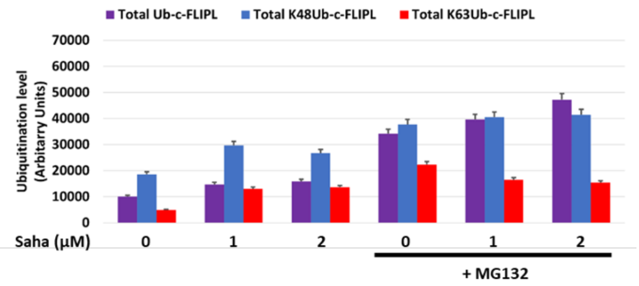

				+MG132		
Saha (μM)	0	1	2	0	1	2
Total Ub-Ku70	18591	19475	23594	38288	48196	40735
Total K48Ub-Ku70	23160	36869	51510	64959	76777	52779
Total K63Ub-Ku70	24310	35130	33946	32368	57847	76162

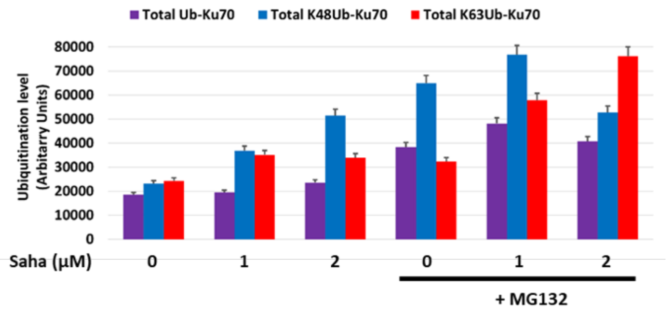

Supplement: Supplementary file 5 — Supplemental Figure 3 [file 41419_2023_6252_MOESM5_ESM.pdf]
