# Supplementary material for: UBE4B interacts with the ITCH E3 ubiquitin ligase to induce Ku70 and c-FLIPL polyubiquitination and enhanced neuroblastoma apoptosis
Source: Cell Death Dis. 2023 Nov 13;14(11):739. doi: 10.1038/s41419-023-06252-7 (PMC10643674; doi:10.1038/s41419-023-06252-7)
Supplement: Supplementary file 7 — Supplemental Figure 5 [file 41419_2023_6252_MOESM7_ESM.pdf]

Supplemental Figure 5 – UBE4B depletion reduces apoptosis induced by HDAC inhibition via reduced Ku70 and c-FLIPL ubiquitination and proteasomal degradation

A

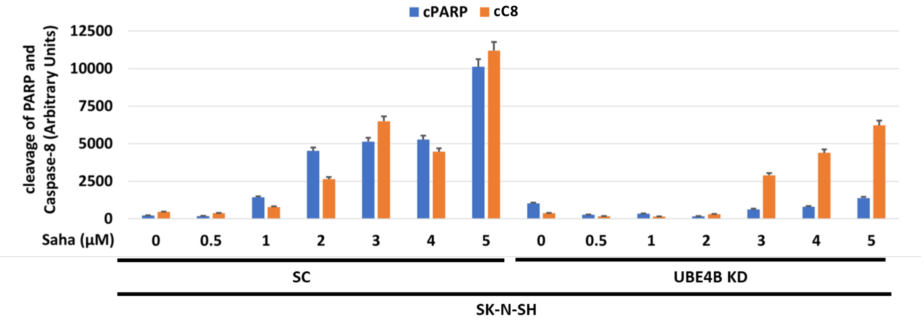

	SC							UBE4B KD						
Saha (µM)	0	0.5	1	2	3	4	5	0	0.5	1	2	3	4	5
cPARP	227	182	1432	4524	5143	5272	10117	1024	280	337	175	632	804	1386
cC8	460	380	788	2644	6497	4464	11201	375	169	154	300	2898	4398	6227

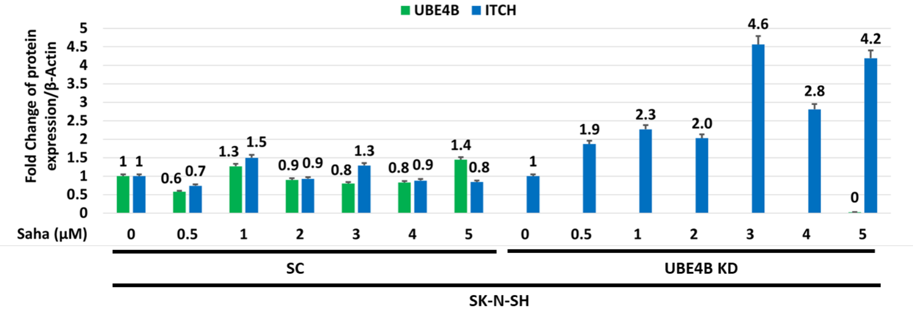

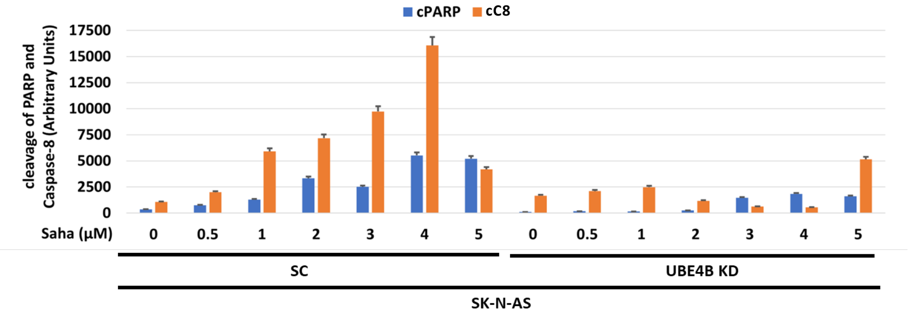

	SC							UBE4B KD						
Saha (µM)	0	0.5	1	2	3	4	5	0	0.5	1	2	3	4	5
cPARP	367	763	1288	3337	2509	5528	5210	109	178	152	247	1458	1839	1607
p18-cC8	1069	1997	5911	7179	9735	16063	4186	1667	2103	2478	1170	625	555	5141
p43/45-cC8	4011	12191	21308	21967	23827	26518	12485	10703	7350	10386	15443	15161	12873	16050

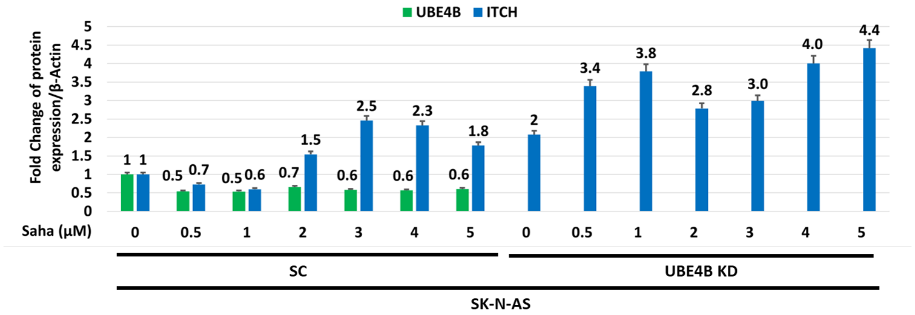

B

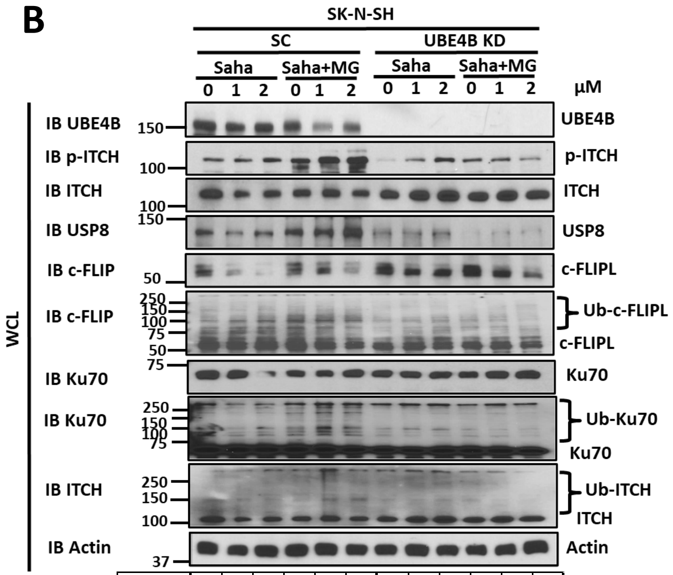

c-FLIPL	1	0.4	0.1	0.9	0.8	0.5	1.4	1.2	1.5	2.0	1.5	0.7
Ku70	1	0.9	0.2	0.5	0.5	0.8	0.8	0.7	0.9	0.8	0.9	0.8
UBE4B	1	0.8	0.6	0.6	0.3	0.5						
p-ITCH	1	1.0	1.0	1.8	2.5	3.5	0.2	0.5	1.3	1.0	1.0	0.5
ITCH	1	0.6	0.5	0.5	0.6	0.6	0.6	0.7	1.0	0.7	0.7	0.6
USP8	1	0.6	0.6	0.9	1.2	1.8	0.8	0.7	0.8	0.1	0.3	0.2

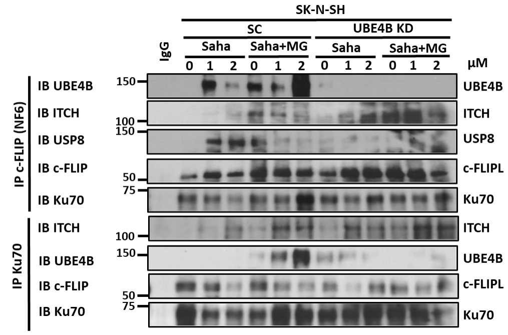

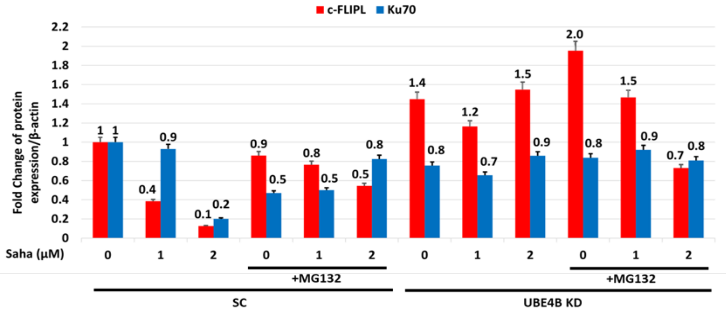

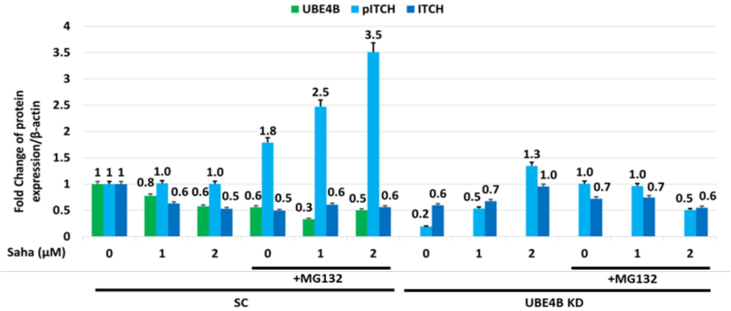

C

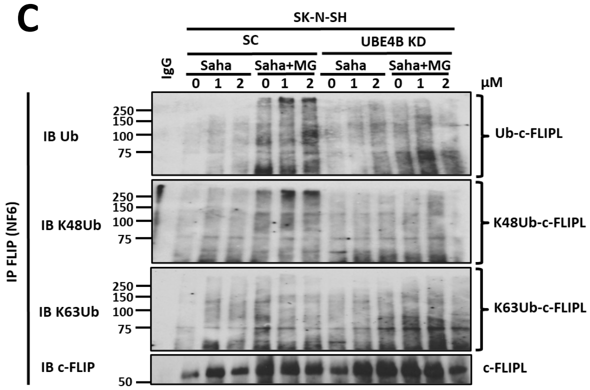

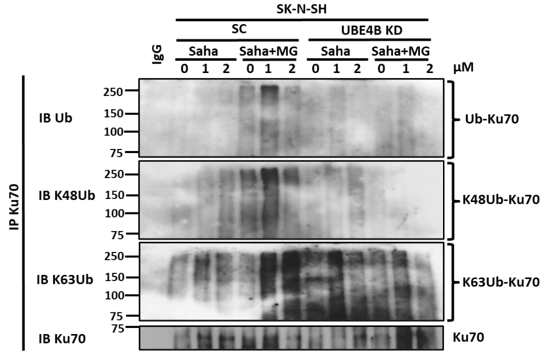

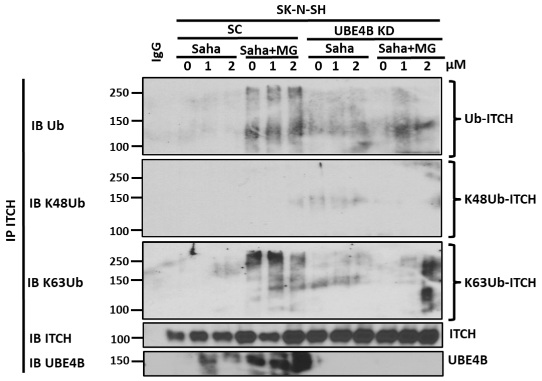

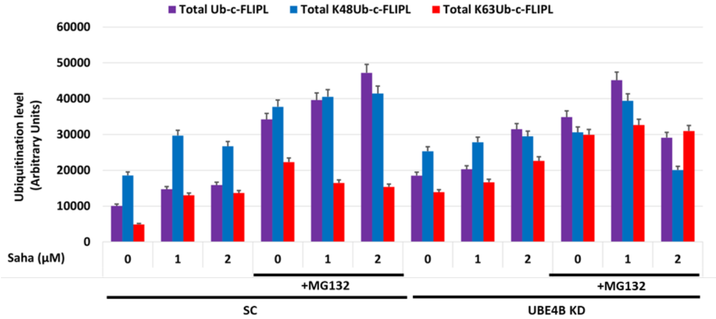

	SC						UBE4B KD					
				MG132						MG132		
Saha (μM)	0	1	2	0	1	2	0	1	2	0	1	2
Total Ub-c-FLIPL	10053	14720	15879	34188	39616	47208	18516	20278	31468	34827	45135	29105
Total K48Ub-c-FLIPL	18569	29700	26700	37730	40513	41413	25328	27841	29487	30586	39397	20087
Total K63Ub-c-FLIPL	4898	13008	13661	22311	16477	15374	13902	16646	22623	29921	32624	30970

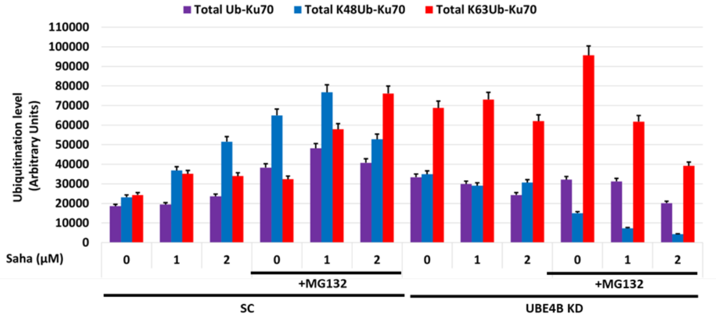

	SC						UBE4B KD					
				MG132						MG132		
Saha (μM)	0	1	2	0	1	2	0	1	2	0	1	2
Total Ub-Ku70	18591	19475	23594	38288	48196	40735	33404	29919	24330	32179	31265	20163
Total K48Ub-Ku70	23160	36869	51510	64959	76777	52779	34905	29087	30640	15029	7297	4318
Total K63Ub-Ku70	24310	35130	33946	32368	57847	76162	68776	73078	62069	95665	61768	39195
