# Supplementary figures and images for: UBE4B interacts with the ITCH E3 ubiquitin ligase to induce Ku70 and c-FLIPL polyubiquitination and enhanced neuroblastoma apoptosis
Source: Cell Death Dis. 2023 Nov 13;14(11):739. doi: 10.1038/s41419-023-06252-7 (PMC10643674; doi:10.1038/s41419-023-06252-7)

# Supplemental Figure 6 – UBE4B depletion reduces Ku70 and c-FLIPL polyubiquitination.

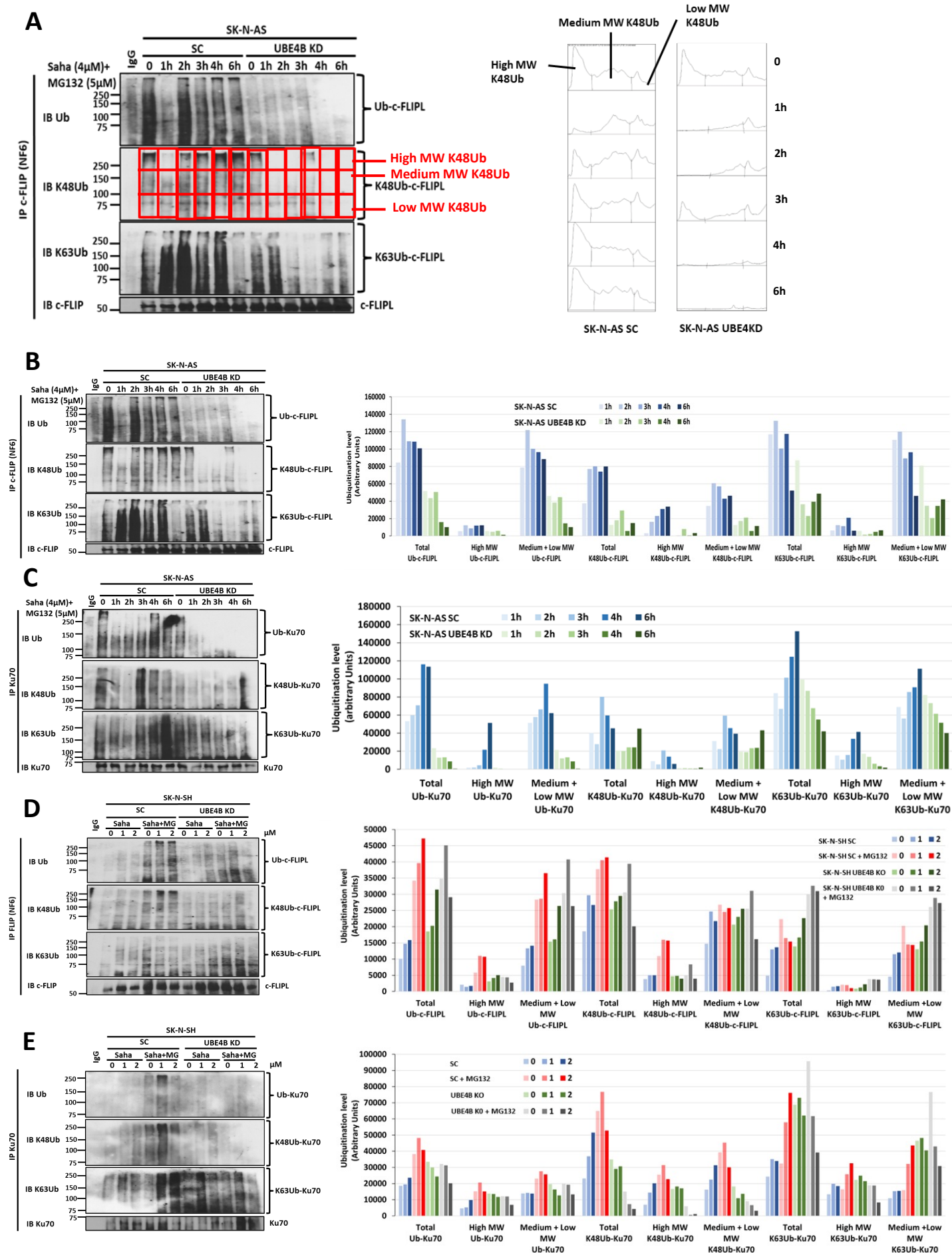

Supplement: Supplementary file 8 — Supplemental Figure 6 [file 41419_2023_6252_MOESM8_ESM.pdf]
