# Supplementary material for: UBE4B interacts with the ITCH E3 ubiquitin ligase to induce Ku70 and c-FLIPL polyubiquitination and enhanced neuroblastoma apoptosis
Source: Cell Death Dis. 2023 Nov 13;14(11):739. doi: 10.1038/s41419-023-06252-7 (PMC10643674; doi:10.1038/s41419-023-06252-7)
Supplement: Supplementary file 9 — Supplemental methods and legends [file 41419_2023_6252_MOESM9_ESM.doc]

**Supplemental Methods**

**Continuous Live Cell Imaging Assays**: Continuous live cell imaging was performed as previously described (52,53). Briefly, neuroblastoma cells were plated in 96-well plates at seeding densities between 10,000-25,000 cells/well and plates were placed into the IncuCyte® Zoom^TM^ continuous live cell imaging system (Essen Bioscience, Ann Arbor, MI). Phase contrast images were taken every 6 hours at 10X magnification for 72 hours. Cell confluence was calculated using IncuCyte® analysis software. Replicates of at least three wells were used for each experimental condition, and the assay was performed at least three independent times. Cell growth curves were generated from the calculated percent cell confluence.

Additional neuroblastoma cells were plated at 500,000 cells/well in 6-well plates and, after 2 days of growth (approximately 80-90% confluence), media was removed and replaced with warm complete new media with 5μM IncuCyte® Caspase 3/7 Green Reagent (Essen Bioscience) with 1μM or 2μM SAHA or DMSO. Plates were evaluated by continuous live-cell imaging in the IncuCyte® Zoom^TM^, and phase contrast and fluorescence images were taken every 4 hours, with sixteen non-overlapping images taken per well at 10X magnification for 48 hours. Average green object counts (each representing individual cells with activated caspase 3/7) per field were generated and normalized to control. Replicates of at least three plates were used for each experimental condition. Error bars corresponding to standard deviations between 3 independent replicates were generated for each experiment, and calculated fold changes in green object counts were compared using 2-sided Student’s t-tests.

**Supplemental Figure Legends**

***Supplemental Figure 1 - ITCH expression is associated with neuroblastoma patient outcomes and prognostic features.*** (A) Using the neuroblastoma SEQC patient data-set in the R2 Genomics Analysis and Visualization Platform (http://r2.amc.nl), patients were divided into high (blue) and low (red) *ITCH* gene expression groups by median-centered Log2 ratios and survival curves were generated. Overall (left) and Event-free (right) survival (top left) curves are shown with patient numbers in parentheses. (B) Relative *ITCH* expression levels were plotted in patients with and without *MYCN* amplification (left) from the SEQC patient data-set, with patient numbers shown in parentheses. Relative *ITCH* expression levels from the SEQC patient data-set were plotted in patients with stage 1, 2, 3, 4, and 4S disease, respectively (middle), with patient numbers shown in parentheses. Relative *ITCH* expression levels were plotted in patients alive and in those who died from disease from the SEQC patient data-set (right), with patient numbers shown in parentheses.

***Supplemental Figure 2 - UBE4B depletion reduces HDAC inhibitor-induced neuroblastoma cell caspase 3/7 activation.*** SH-SY5Y (A-C), SK-N-SH (D-F), SK-N-BE(2) (G-I), and SK-N-AS (J-L) cells expressing scrambled control shRNA (SC) or UBE4B shRNA for UBE4B knockdown (UBE4B KD) were treated with DMSO or with 1μM or 2μM SAHA for 24 or 48 hours and evaluated for caspase 3/7 cleavage using the IncuCyte Zoom^TM^, with individual green dots representing cells with active caspase 3/7. (A,D,G,J) The percentage of green cells representing active caspase 3/7 compared to total cell number determined by phase-contrast imaging were quantified over 48 hours of SAHA treatment. (B,E,H,K) Images of untreated and treated cells. (C,F,I,L) Fold change of green dot percentage representing caspase 3/7 activation compared between treated and untreated cells at each dose and time point. Final results were compared using Student's t-tests. (***p<0.001 and *p<0.005)

***Supplemental Figure 3 - HDAC inhibition induces Ku70 and c-FLIPL acetylation and Lys48/Lys63 branched polyubiquitination via the ITCH-UBE4B complex.*** (A) Band intensities for cleaved PARP (cPARP) as well as cleaved caspase-8 (cC8) fragments from Western blots in Figure 5B were calculated from 5 neuroblastoma cell lines using arbitrary units and are shown in the tables and in the graphs (cPARP - violet; cC8 - orange)(Top graphs). Band intensities for c-FLIPL (red), Ku70 (dark blue), UBE4B (green) and ITCH (light blue) from the Western blots in Figure 5B were normalized to β-actin expression and also compared to intensities in untreated cells (Bottom graphs). (B) SK-N-SH neuroblastoma cells were treated for 3 hours with either 5μM MG132 or vehicle alone, followed by either 1 or 2μM vorinostat (Saha) for 18 hours. Whole cell lysates were then evaluated by Western blots for c-FLIPL, Ku70, USP8, UBE4B, phosphorylated ITCH (on threonine-222, p-ITCH), total ITCH, and β-actin (Top Left). Band intensities relative to control lanes are shown in the table (Bottom Left). Band intensities for c-FLIPL (Top Middle), Ku70 (Top Right), USP8 (Middle), p-ITCH (Middle Right), UBE4B (Bottom Middle) and ITCH (Bottom Right) normalized to β-actin expression were compared in independent graphs in cells treated with Saha without pre-treatment with MG132 (blue lines) and with cells treated with Saha with MG132 pre-treatment (orange lines). (C) Band intensities for UBE4B, ITCH and pITCH from Western blots in Figure 5D were compared in independent graphs in cells treated with Saha without pre-treatment with MG132 (blue lines) and with cells treated with Saha with MG132 pre-treatment (orange lines). (D) SK-N-SH neuroblastoma cells were treated for 3 hours with either 5μM MG132 or vehicle alone, followed by either 1 or 2μM vorinostat (Saha) for 18 hours. Whole cell lysates (WCL) were then evaluated by Western blots for UBE4B, c-FLIPL, Ku70, ubiquitin, and β-actin (Top Right). Immunoprecipitated c-FLIPL (Top Left) and Ku70 (Top Middle) were isolated and evaluated by Western blots for ubiquitin, Lys48-linked ubiquitin (K48Ub), Lys63-linked ubiquitin (K63Ub), c-FLIPL, and Ku70. (Bottom) Absolute band intensities of total ubiquitinated (violet), Lys48-linked ubiquitinated (K48Ub; blue), and Lys63-linked ubiquitinated (K63Ub; red)) c-FLIPL and Ku70 are shown in the tables and were compared in each condition (Bottom).

***Supplemental Figure 4 - HDAC inhibitor-mediated induction of Ku70 and c-FLIPL Lys48/Lys63 polyubiquitination.*** Immunoblots from Figure 5G and Supplemental Figure 3D (duplicated here for direct comparison) were analyzed for relative levels of ubiquitination. **(A)** Images of ubiquitin, K48Ub and K63Ub immunoprecipitates were quantified using ImageJ software and were decomposed into 3 regions (High Molecular Weight (MW), Medium MW, and Low MW) (shown on the right) corresponding to the length of the polyubiquitin chains and quantified using arbitrary units of intensity. Images of immunoprecipitates of c-FLIPL **(B)** and Ku70 **(C)** from SK-N-AS neuroblastoma cells that were immunoblotted for ubiquitin, K48Ub, K63Ub from cells pre-treated for 2 hours with 5μM MG132, followed by addition of 4μM vorinostat (Saha) for the indicated times underwent quantification of the total, high MW, and medium MW ubiquitin; of the total, high MW, and medium MW K48Ub; and of the total, high MW, and medium MW K63. Images of immunoprecipitates of c-FLIPL **(D)** and Ku70 **(E)** from SK-N-SH neuroblastoma cells that were immunoblotted for ubiquitin, K48Ub, K63Ub from cells pre-treated for 3 hours with either 5μM MG132 or vehicle alone, followed by either 1 or 2μM vorinostat (Saha) for 18 hours underwent quantification of the total, high MW, and medium plus low MW ubiquitin; of the total, high MW, and medium plus low MW K48Ub; and of the total, high MW, and medium plus low MW K63.

***Supplemental Figure 5 - UBE4B depletion reduces apoptosis induced by HDAC inhibition via reduced Ku70 and c-FLIPL ubiquitination and proteasomal degradation.*** (A) Band intensities for cleaved PARP (cPARP) as well as cleaved caspase-8 (cC8) fragments from Western blots in Figures 6A and 6B were calculated using arbitrary units and are shown in the tables for SK-N-SH (Top) and SK-N-AS (Bottom) cells. Band intensities of cPARP (blue) and cC8 (orange) were compared compared in cell lines treated with increasing doses of vorinostat (Saha) between SC vs UBE4B KD cells (Top graphs). Band intensities for UBE4B (green) and ITCH (blue) normalized to β-actin were compared in cell lines treated with increasing dose of vorinostat (Saha) between SC vs UBE4B KD cells (Bottom graphs). (B) SK-N-SH neuroblastoma cells expressing scrambled control sgRNA (SC) or *UBE4B* sgRNA for UBE4B knockdown (UBE4B KD) were treated for 3 hours with either 5µM MG132 or vehicle alone, followed by either 1 or 2μM vorinostat (Saha) for 18 hours. Whole cell lysates (WCL) were then evaluated by Western blots for UBE4B, p-ITCH, total ITCH, USP8, c-FLIPL, Ku70, and β-actin (Top Left). Immunoprecipitated c-FLIPL and Ku70, were each isolated and evaluated by Western blots for UBE4B, ITCH, USP8, c-FLIPL, and Ku70 (Top Right). Band intensities for c-FLIPL (red), Ku70 (dark blue), UBE4B (green), p-ITCH (light blue), and ITCH (dark blue) were normalized to β-actin and compared in each cell line at each dose (Bottom Right). (C) Immunoprecipitated c-FLIPL (Top Left), Ku70 (Middle Left) and ITCH (Bottom Left) were isolated and evaluated by Western blots for ubiquitin, Lys48-linked ubiquitin (K48Ub), Lys63-linked ubiquitin (K63Ub), c-FLIPL, Ku70, ITCH, and UBE4B. Absolute band intensities of total ubiquitinated (violet), Lys48-linked ubiquitinated (K48Ub; blue), and Lys63-linked ubiquitinated (K63Ub; red)) c-FLIPL and Ku70 are shown in the tables and were compared in each cell line at each dose (Right).

***Supplemental Figure 6 - UBE4B depletion reduces Ku70 and c-FLIPL polyubiquitination.*** Immunoblots from Figures 6C and 6D (duplicated here for direct comparison) were analyzed for relative levels of ubiquitination. **(A)** Images of ubiquitin, K48Ub and K63Ub immunoprecipitates were quantified using ImageJ software and were decomposed into 3 regions (High Molecular Weight (MW), Medium MW, and Low MW) (shown on the right) corresponding to the length of the polyubiquitin chains and quantified using arbitrary units of intensity. Images of immunoprecipitates of c-FLIPL **(B)** and Ku70 **(C)** from SK-N-AS SC and SK-N-AS UBE4B KD neuroblastoma cells that were immunoblotted for ubiquitin, K48Ub, K63Ub from cells pre-treated for 2 hours with 5μM MG132, followed by addition of 4μM vorinostat (Saha) for the indicated times underwent quantification of the total, high MW, and medium plus low MW ubiquitin; of the total, high MW, and medium plus low MW K48Ub; and of the total, high MW, and medium plus low MW K63. Images of immunoprecipitates of c-FLIPL **(D)** and Ku70 **(E)** from SK-N-SH SC and SK-N-SH UBE4B KD neuroblastoma cells that were immunoblotted for ubiquitin, K48Ub, K63Ub from cells pre-treated for 3 hours with either 5μM MG132 or vehicle alone, followed by either 1 or 2μM vorinostat (Saha) for 18 hours underwent quantification of the total, high MW, and medium plus low MW ubiquitin; of the total, high MW, and medium plus low MW K48Ub; and of the total, high MW, and medium plus low MW K63.
