# Supplementary material for: UBE4B interacts with the ITCH E3 ubiquitin ligase to induce Ku70 and c-FLIPL polyubiquitination and enhanced neuroblastoma apoptosis
Source: Cell Death Dis. 2023 Nov 13;14(11):739. doi: 10.1038/s41419-023-06252-7 (PMC10643674; doi:10.1038/s41419-023-06252-7)

NB SC vs UBE4B KD

Fig 1A

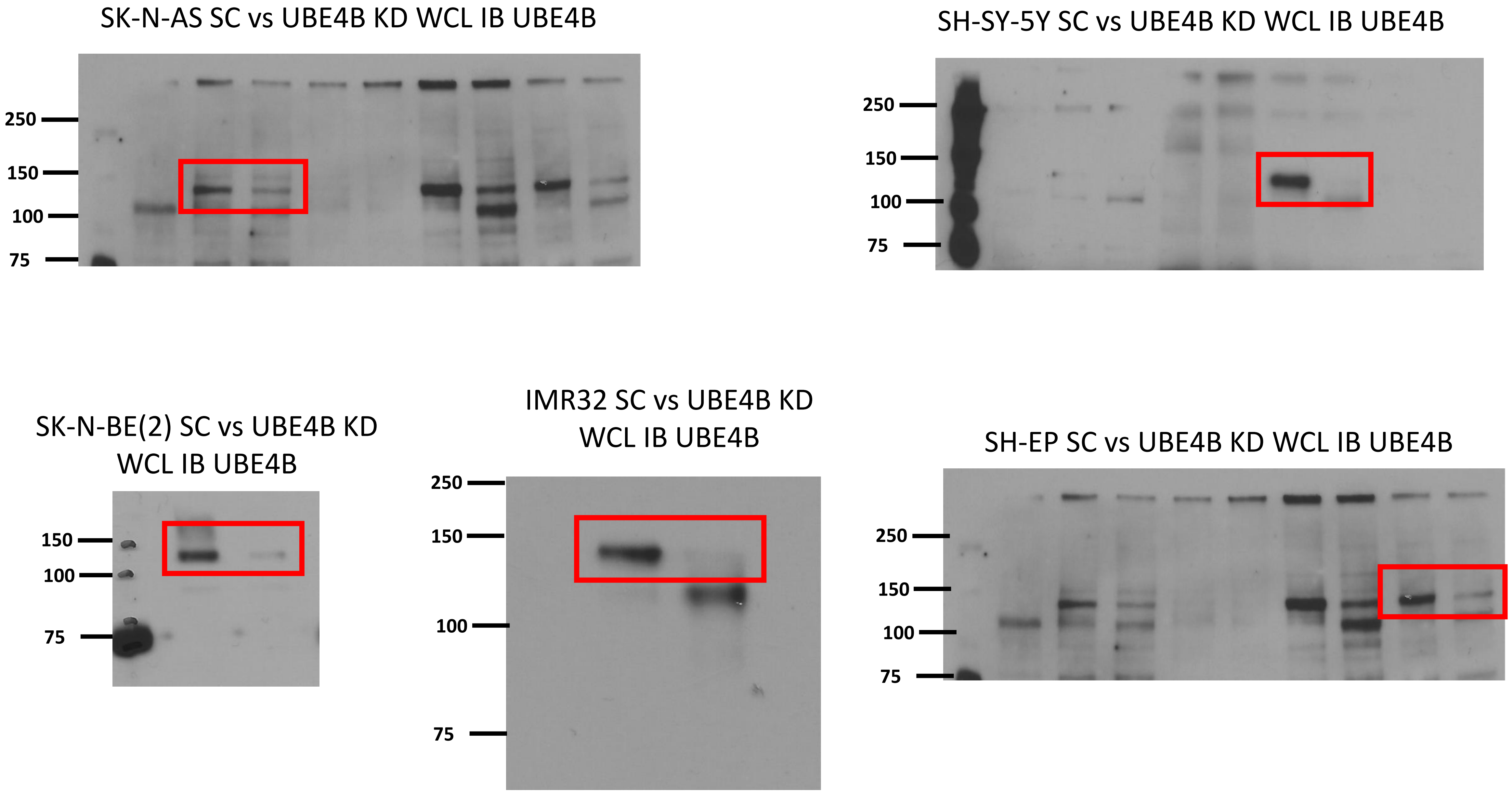

NB SC vs UBE4B KD

Fig 1A

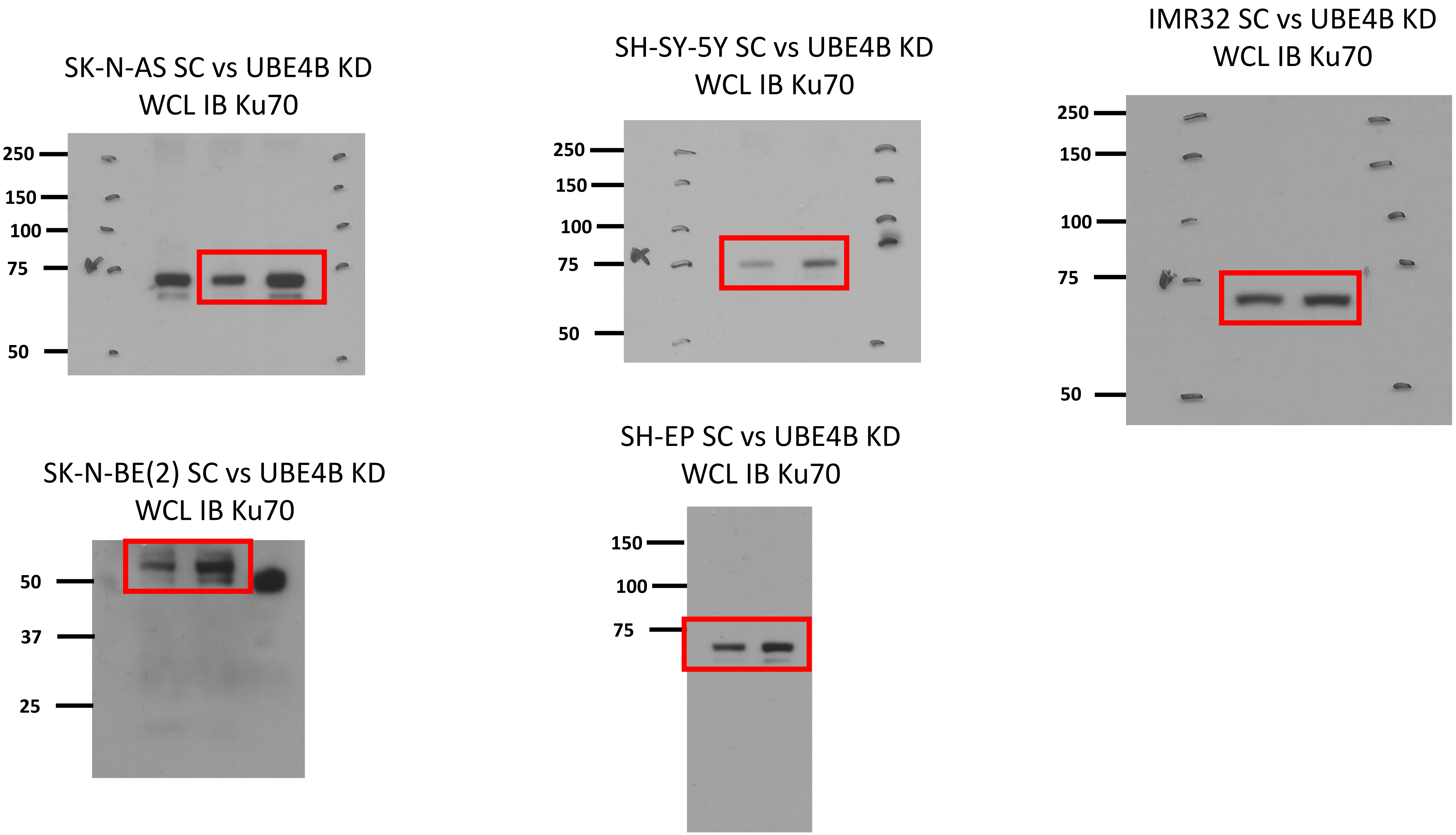

NB SC vs UBE4B KD

Fig 1A

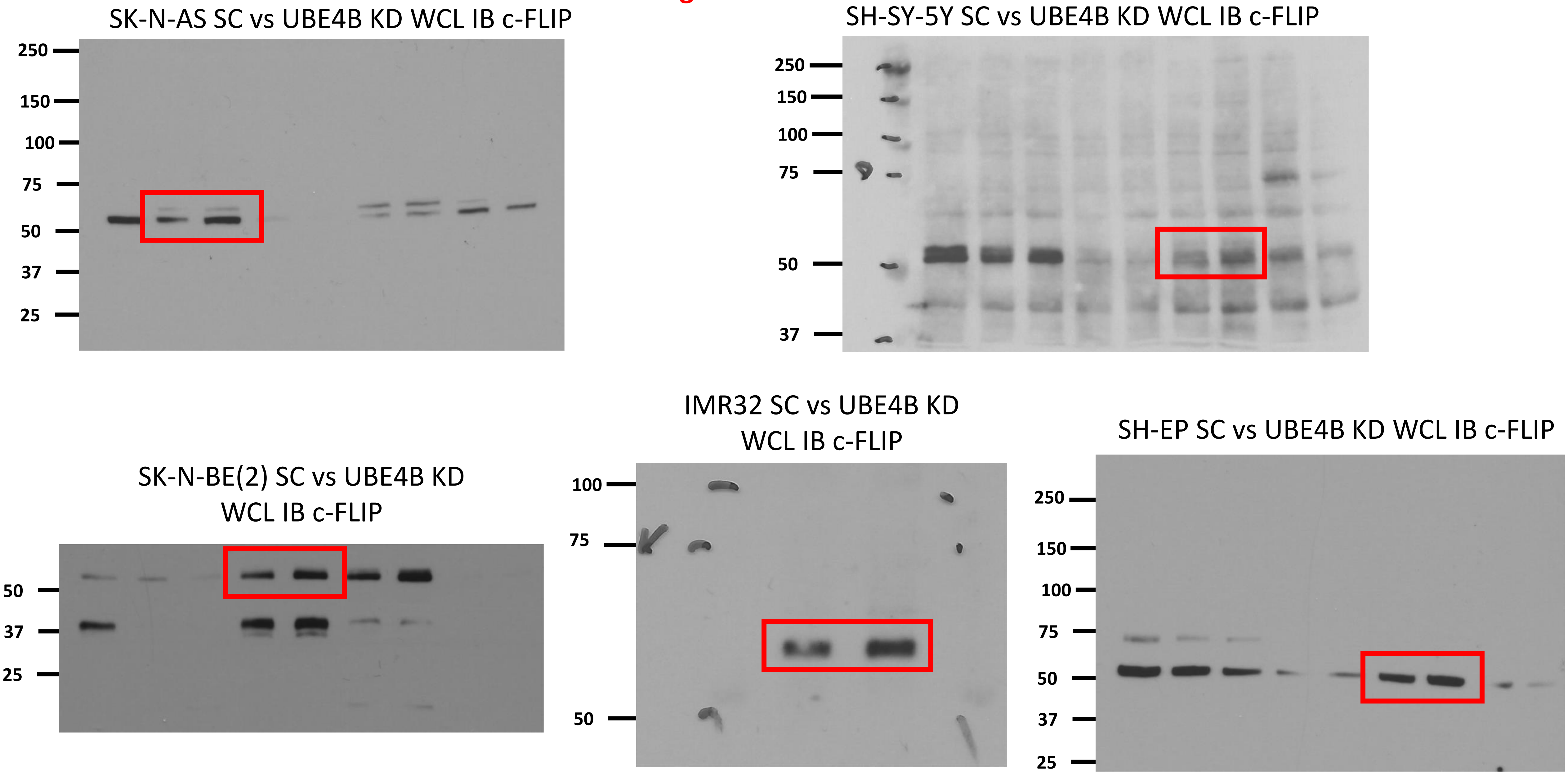

NB SC vs UBE4B KD

Fig 1A

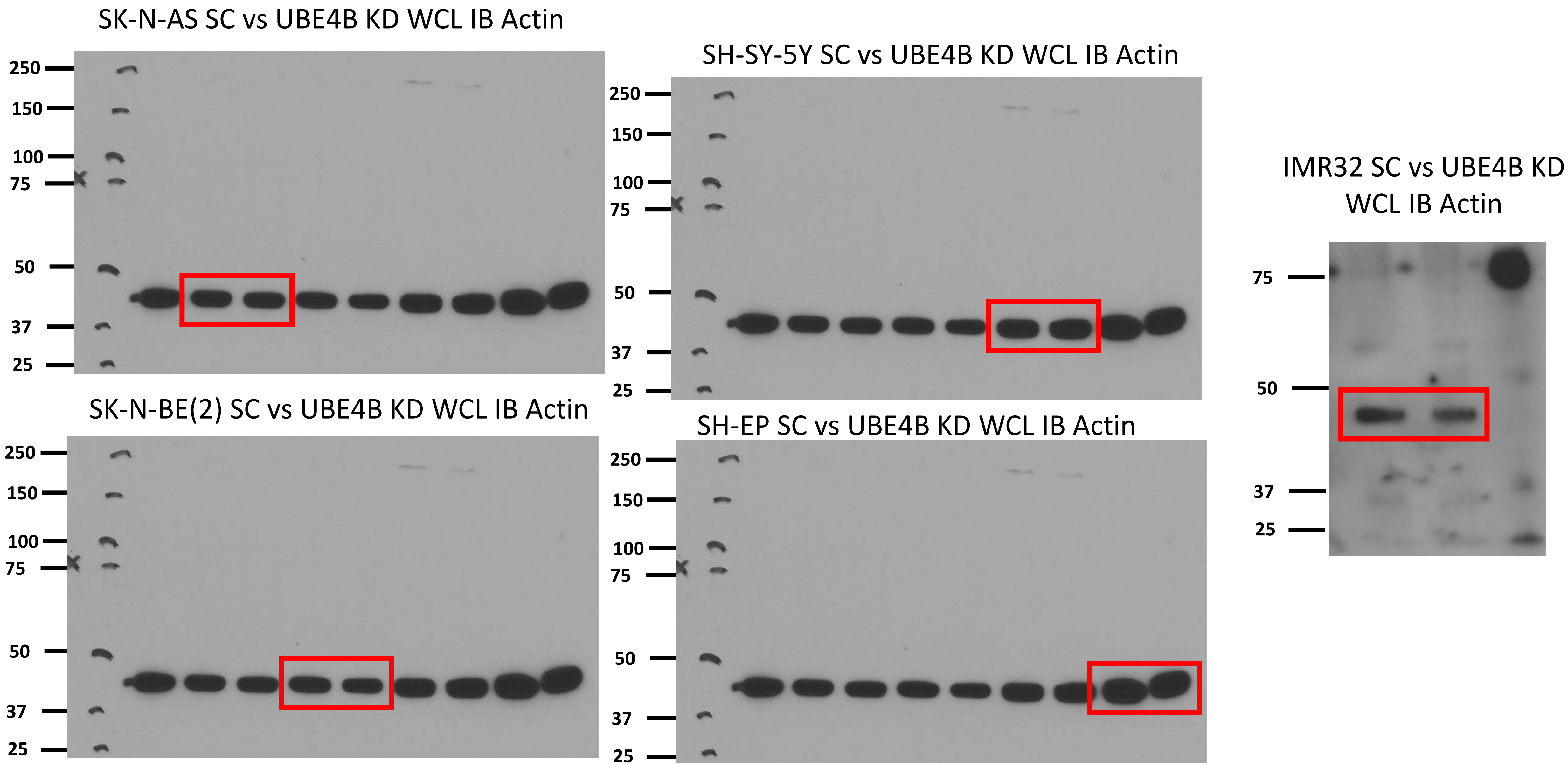

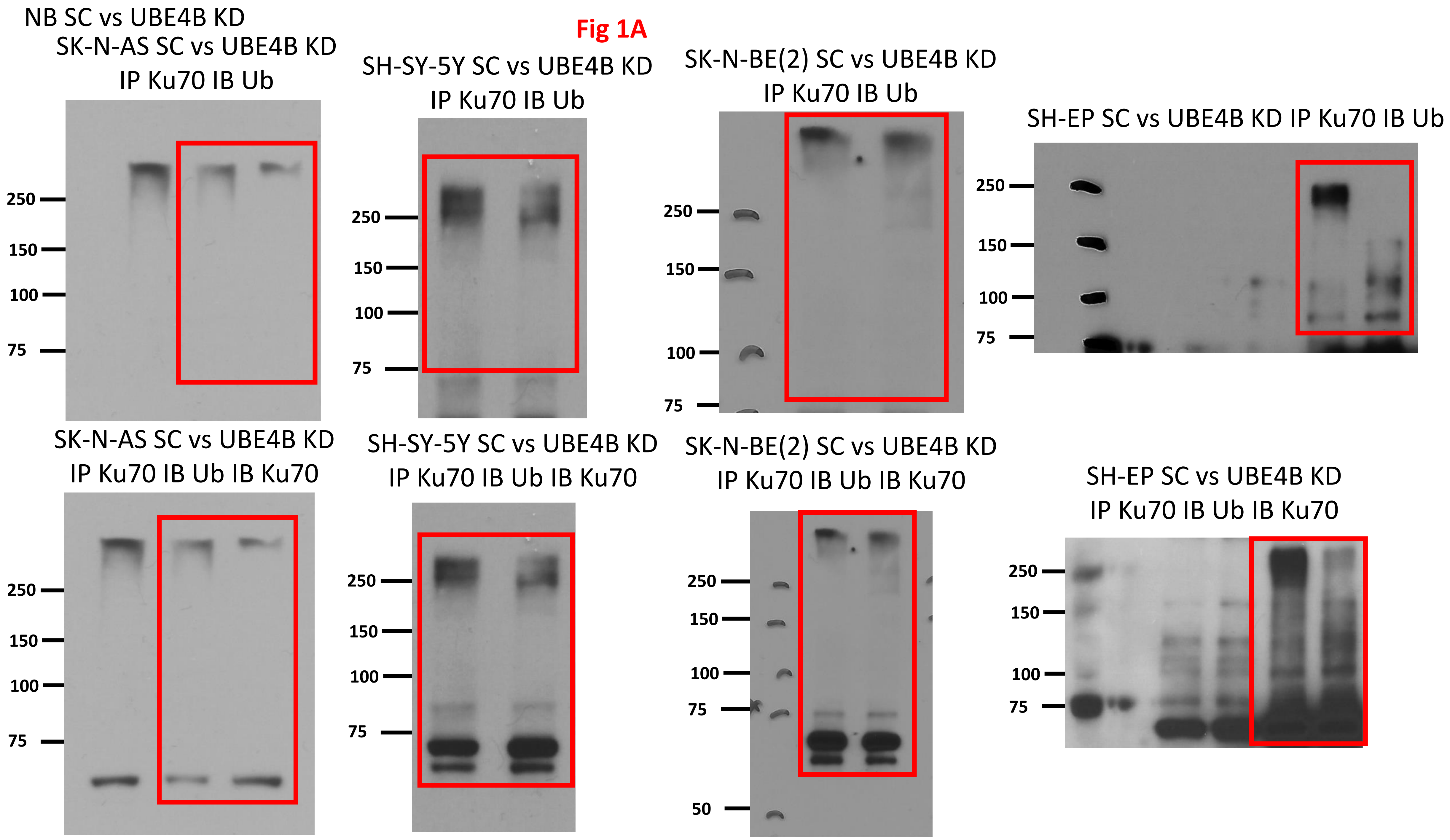
Fig 1A
NB SC vs UBE4B KD
SK-N-AS SC vs UBE4B KD
IP Ku70 IB Ub
250
150
100
75
SH-SY-5Y SC vs UBE4B KD
IP Ku70 IB Ub
250
150
100
75
SK-N-BE(2) SC vs UBE4B KD
IP Ku70 IB Ub
250
150
100
75
SH-EP SC vs UBE4B KD IP Ku70 IB Ub
250
150
100
75
SK-N-AS SC vs UBE4B KD
IP Ku70 IB Ub IB Ku70
250
150
100
75
SH-SY-5Y SC vs UBE4B KD
IP Ku70 IB Ub IB Ku70
250
150
100
75
SK-N-BE(2) SC vs UBE4B KD
IP Ku70 IB Ub IB Ku70
250
150
100
75
50
SH-EP SC vs UBE4B KD
IP Ku70 IB Ub IB Ku70
250
150
100
75

NB SC vs UBE4B KD

Fig 1A

IMR32 SC vs UBE4B KD
IP Ku70 IB Ub

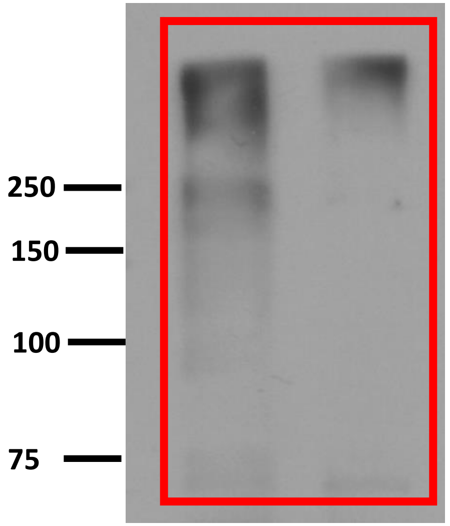

IMR32 SC vs UBE4B KD
IP Ku70 IB Ub IB Ku70

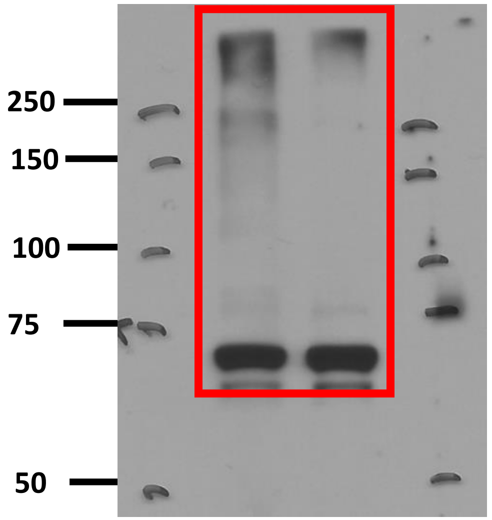

NB SC vs UBE4B KD

Fig 1A

SK-N-AS SC vs UBE4B KD IP c-FLIPL IB Ub

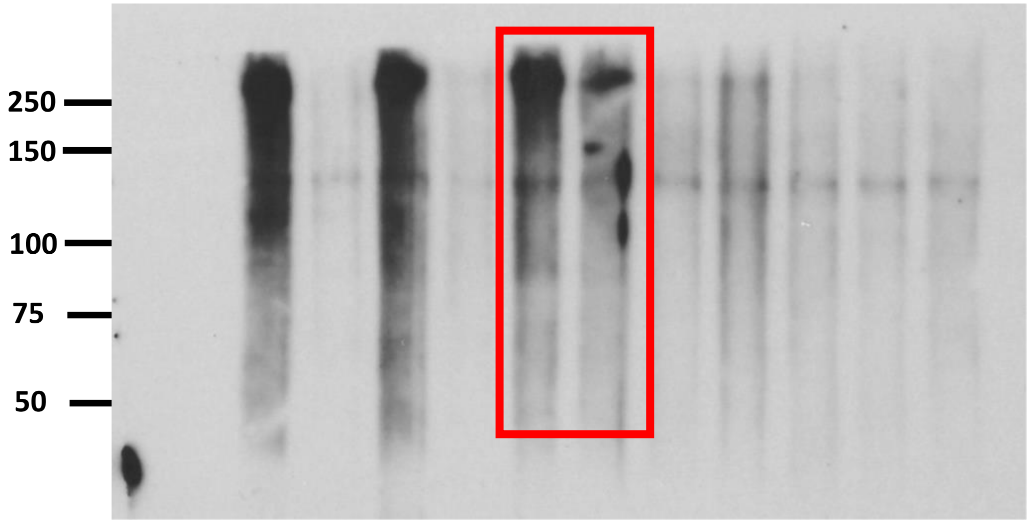

SH-SY-5Y SC vs UBE4B KD IP c-FLIPL IB Ub

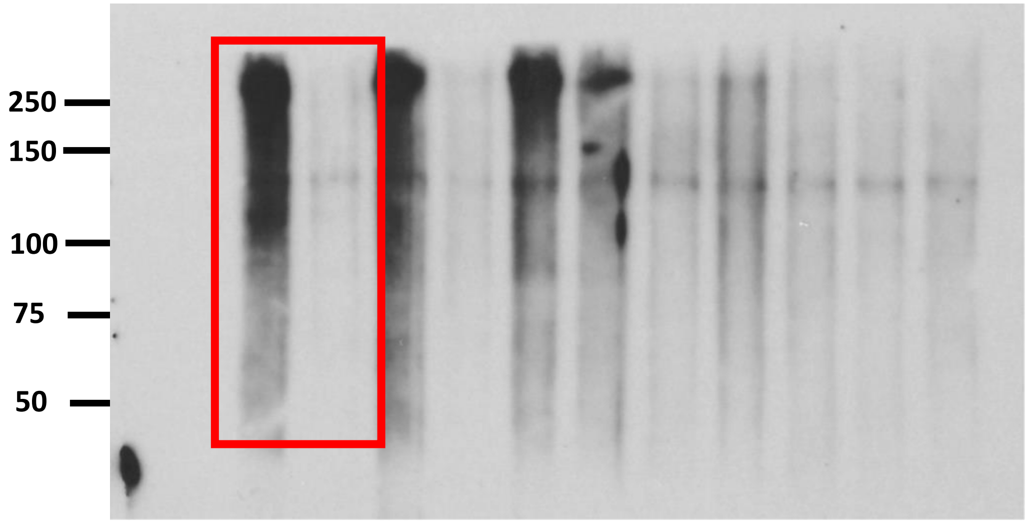

IMR-32 SC vs UBE4B KD IP c-FLIPL IB Ub

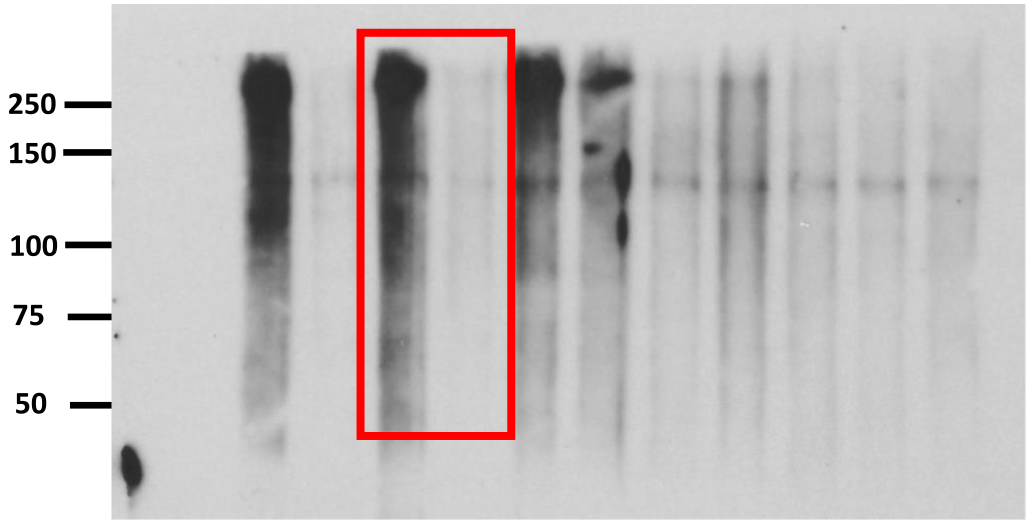

SK-N-BE(2) SC vs UBE4B KD
IP c-FLIPL IB Ub

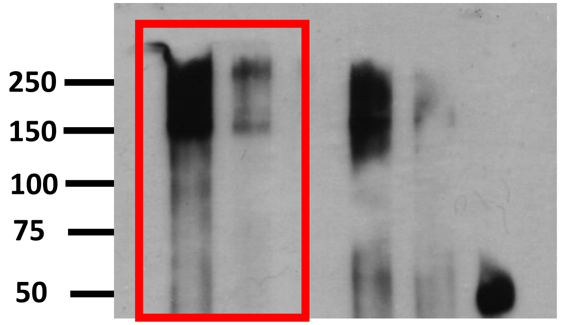

SH-EP SC vs UBE4B KD
IP c-FLIPL IB Ub

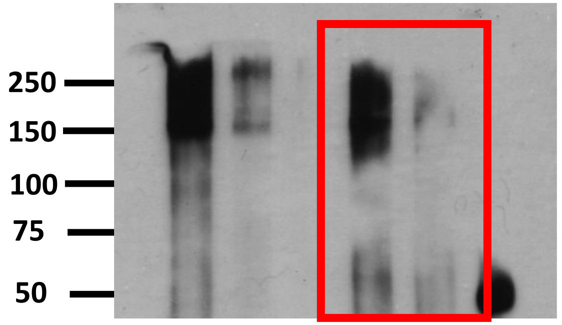

NB SC vs UBE4B KD

Fig 1B

SK-N-AS SC vs UBE4B KD WCL IB UBE4B

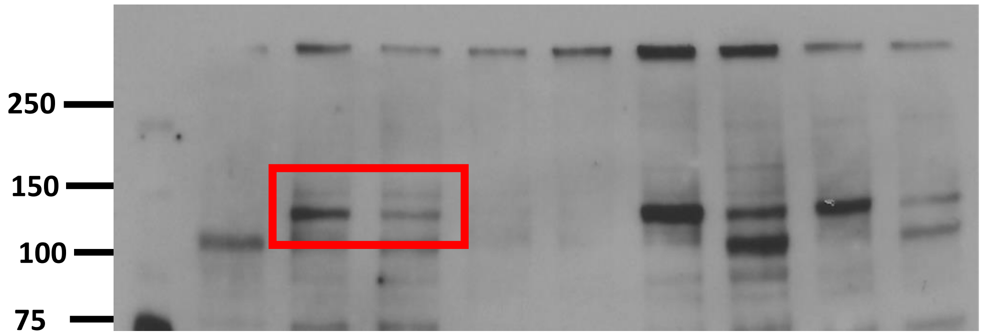

SH-EP SC vs UBE4B KD WCL IB UBE4B

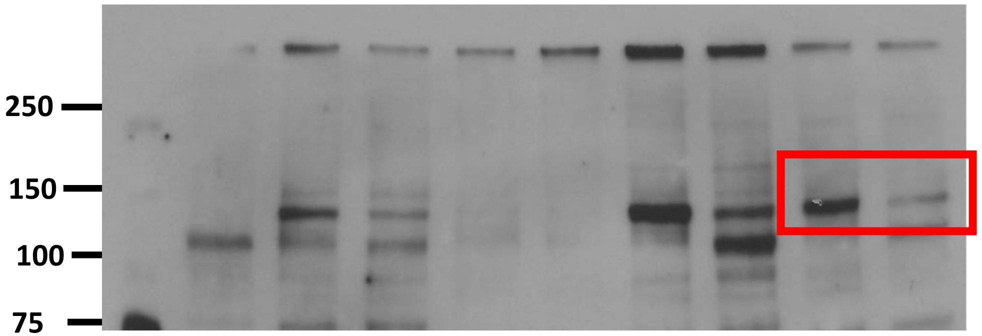

IMR32 SC vs UBE4B KD WCL IB UBE4B

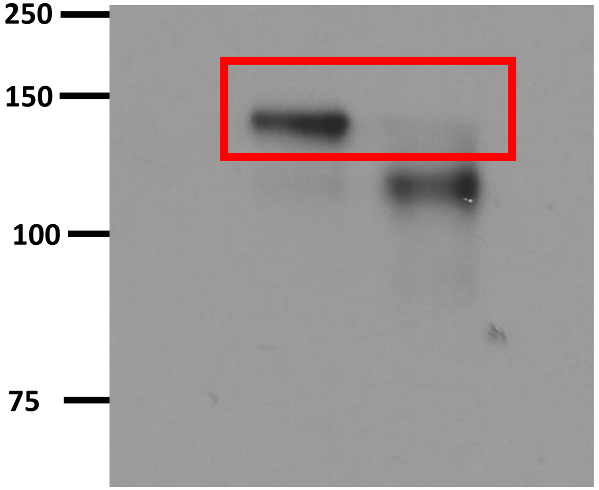

NB SC vs UBE4B KD

Fig 1B

SK-N-AS SC vs UBE4B KD
WCL IB p53 (short Expo)

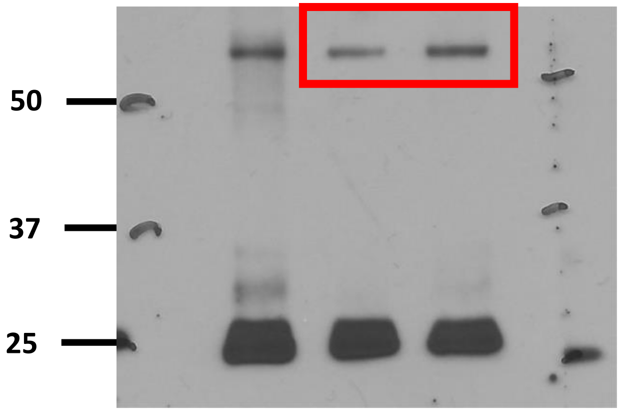

SK-N-AS SC vs UBE4B KD
WCL IB p53 (Long Expo)

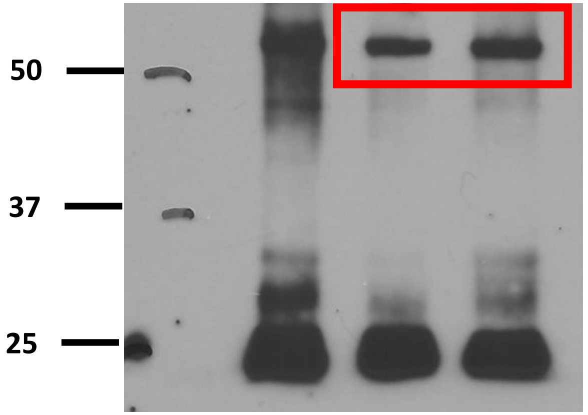

IMR32 SC vs UBE4B KD
WCL IB p53 (short Expo)

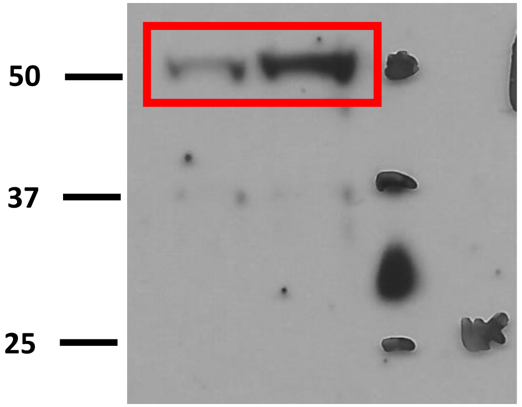

IMR32 SC vs UBE4B KD
WCL IB p53 (Long Expo)

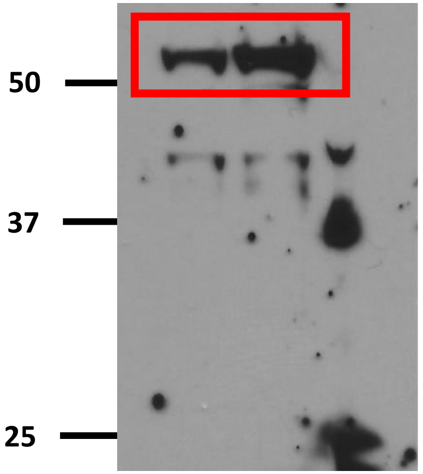

SH-EP SC vs UBE4B KD
WCL IB p53 (Short Expo)

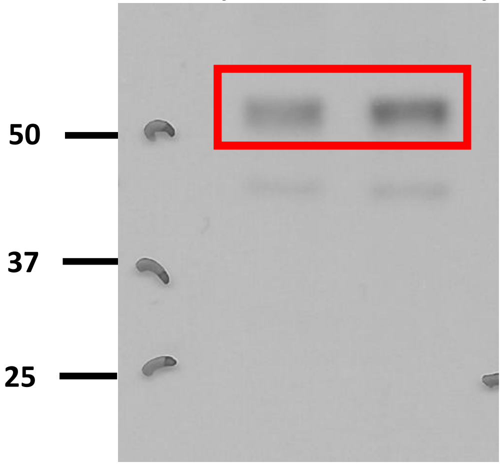

SH-EP SC vs UBE4B KD
WCL IB p53 (Long Expo)

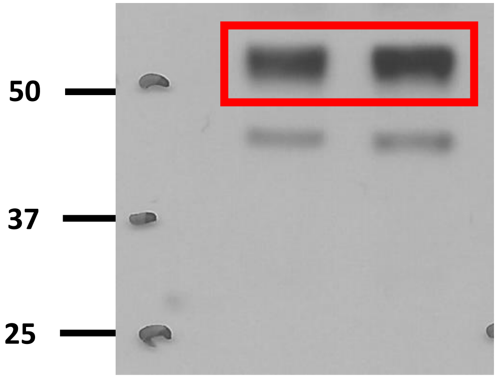

Fig 1B

NB SC vs UBE4B KD

SK-N-AS SC vs UBE4B KD WCL IB Actin

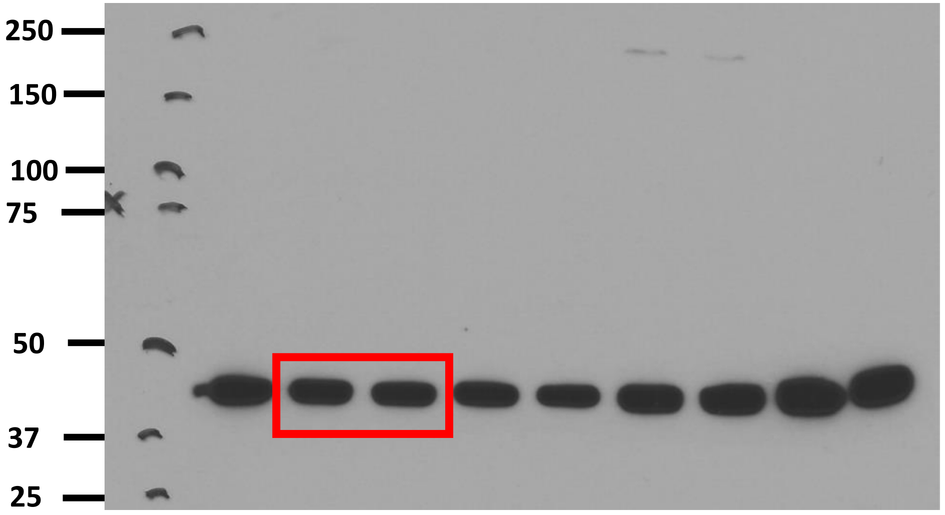

SH-EP SC vs UBE4B KD WCL IB Actin

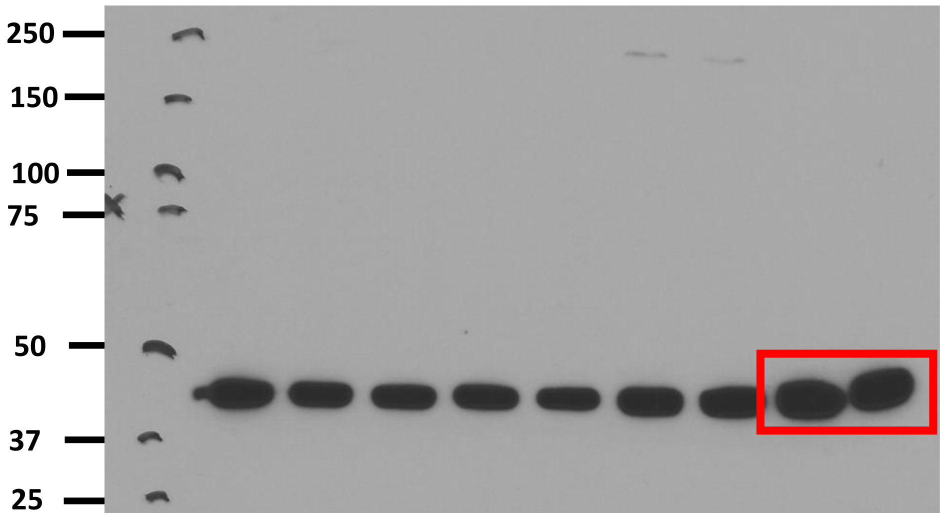

IMR32 SC vs UBE4B KD

WCL IB Actin

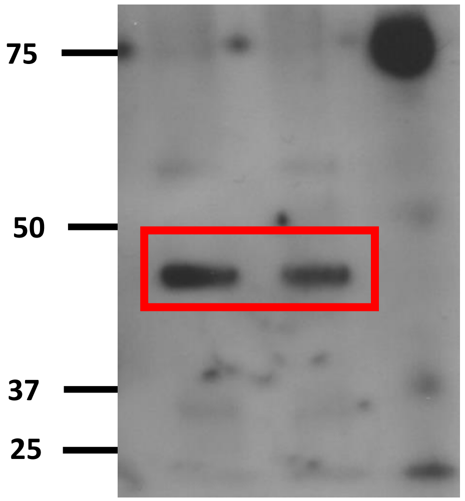

NB SC vs UBE4B KD

Fig 1B

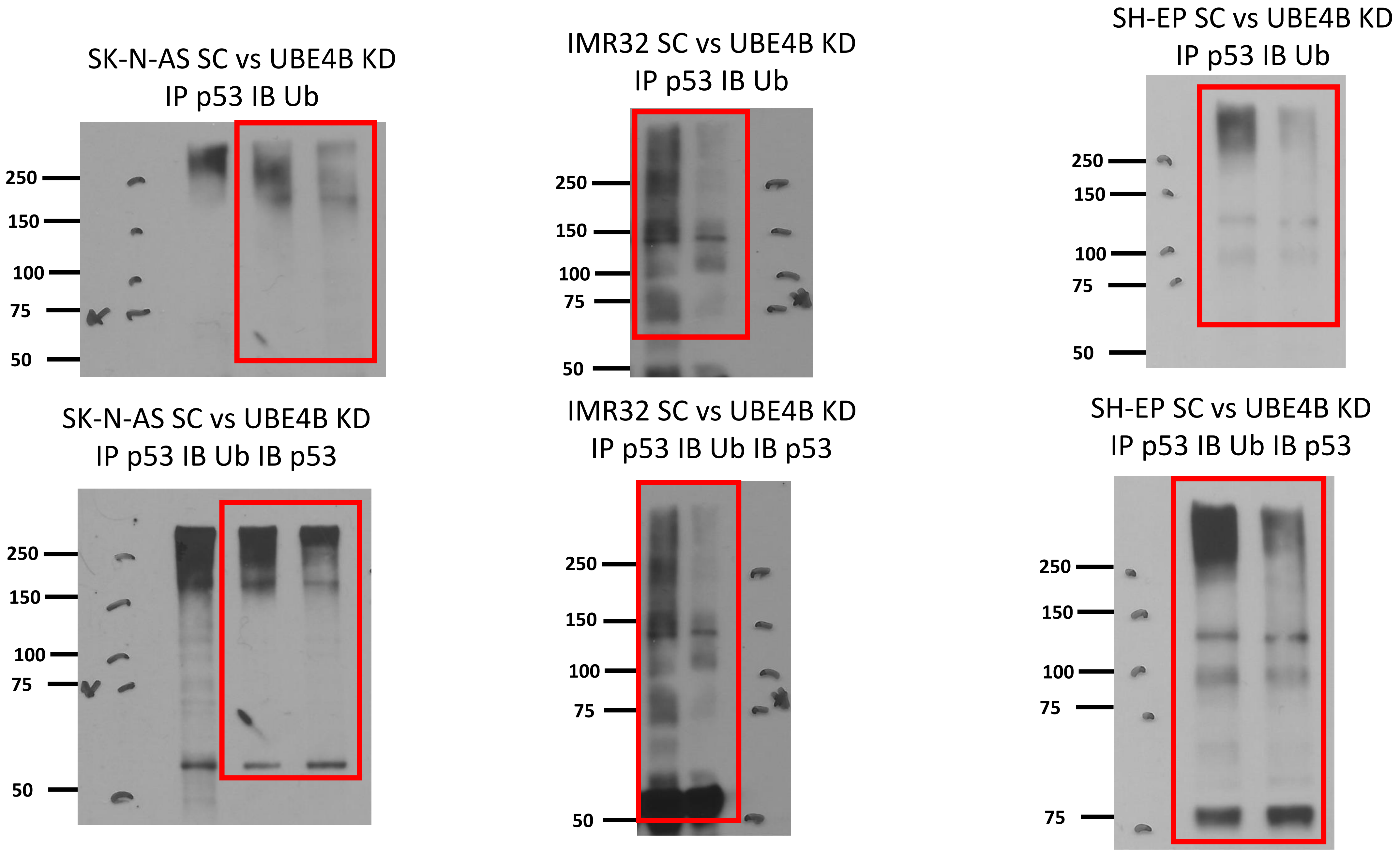

Fig 2F

HEK Myc-ITCHwt Flag-c-FLIPL

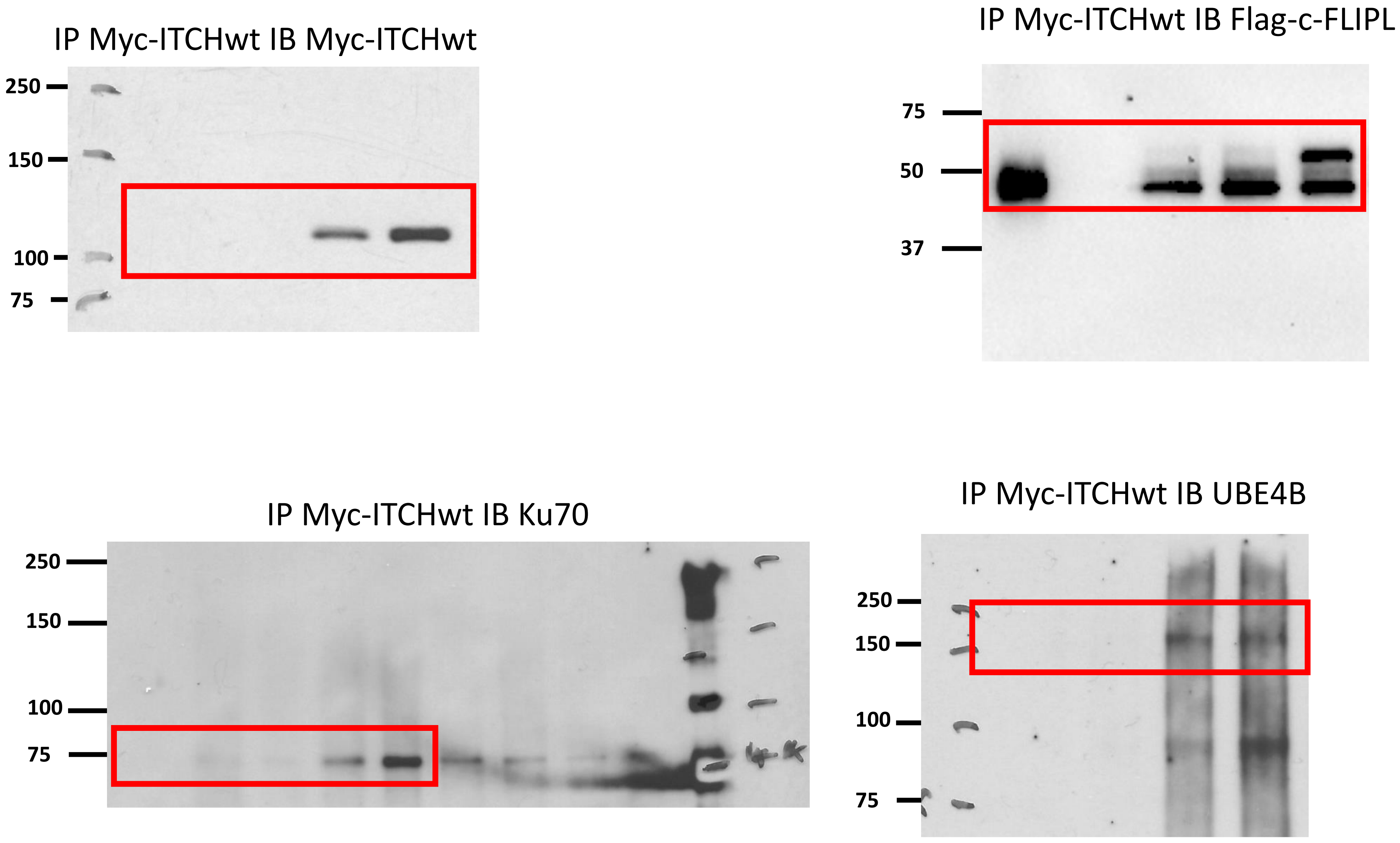

HEK Myc-ITCHwt Flag-c-FLIPL

Fig 2F

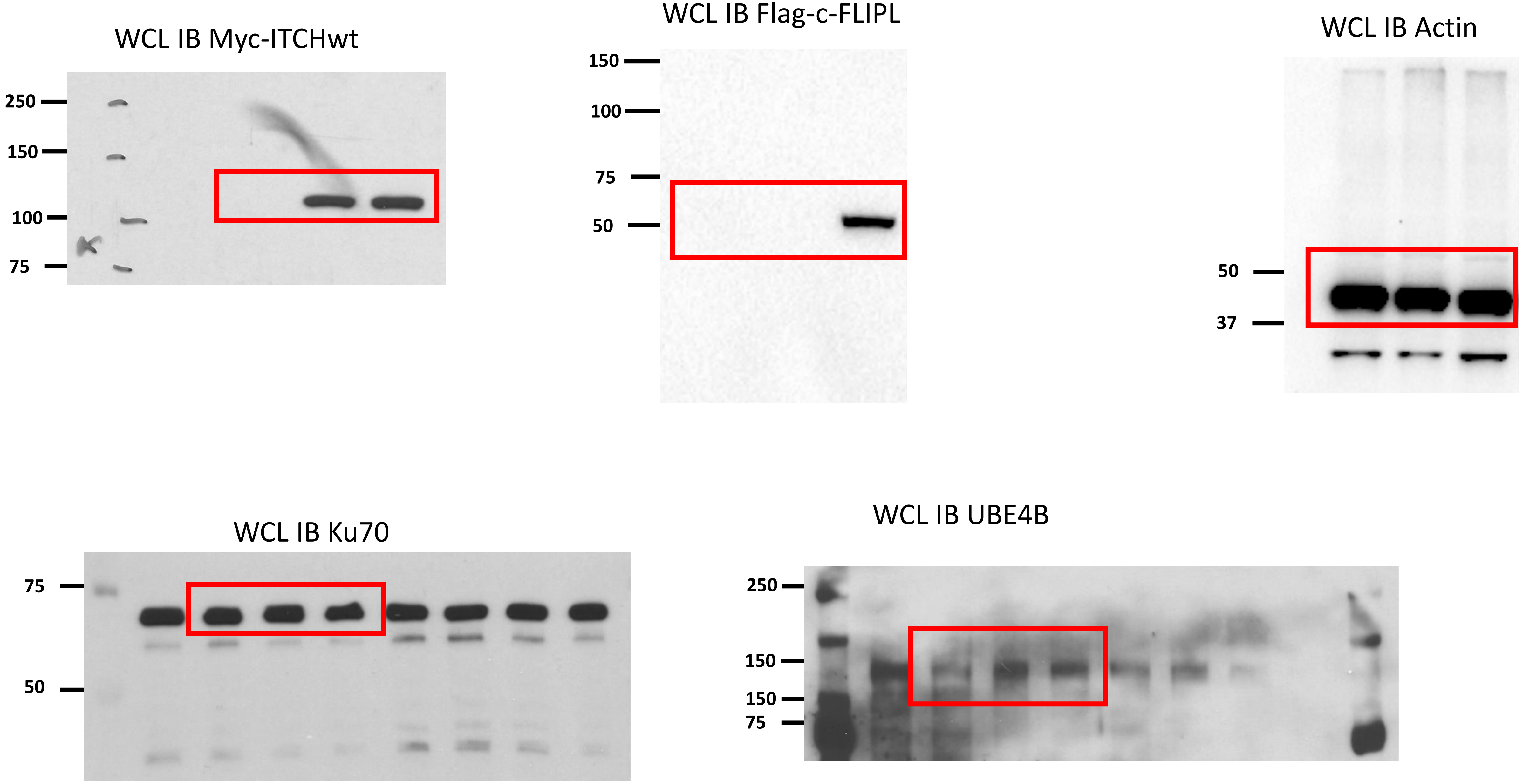

HEK Myc-ITCHwt Myc-Ku70 Flag-c-FLIPL

Fig 2G

IP Flag-c-FLIPL IB ITCH

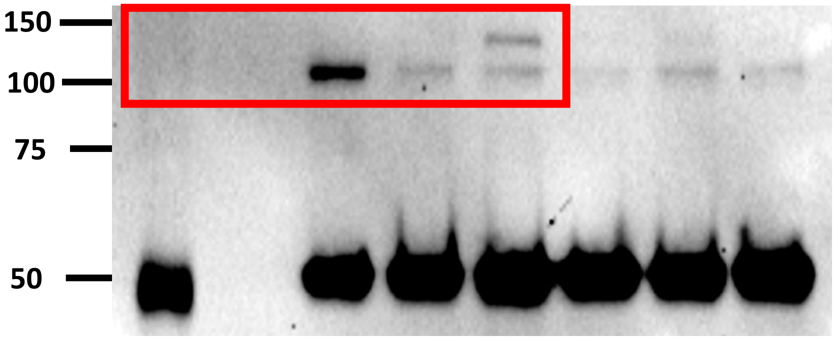

IP Flag-c-FLIPL IB Flag-c-FLIPL

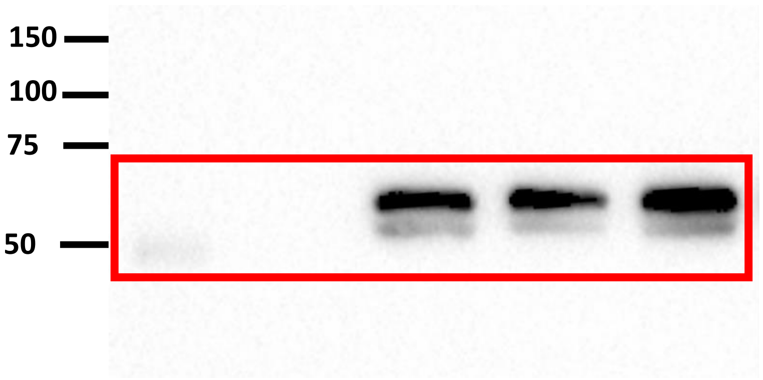

IP Flag-c-FLIPL IB Myc-Ku70

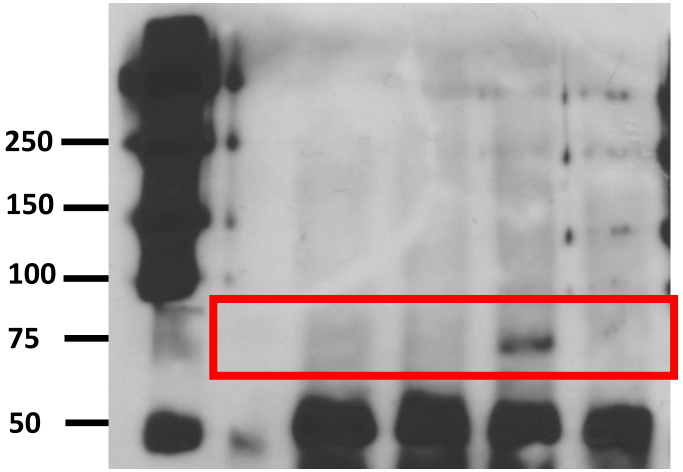

IP Flag-c-FLIPL IB Ku70

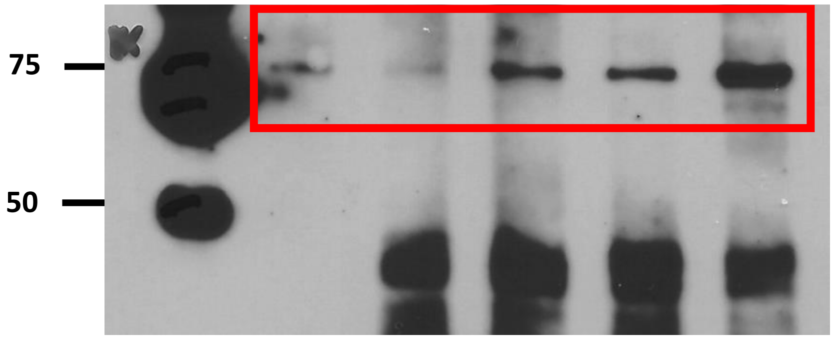

HEK Myc-ITCHwt Myc-Ku70 Flag-c-FLIPL

Fig 2G

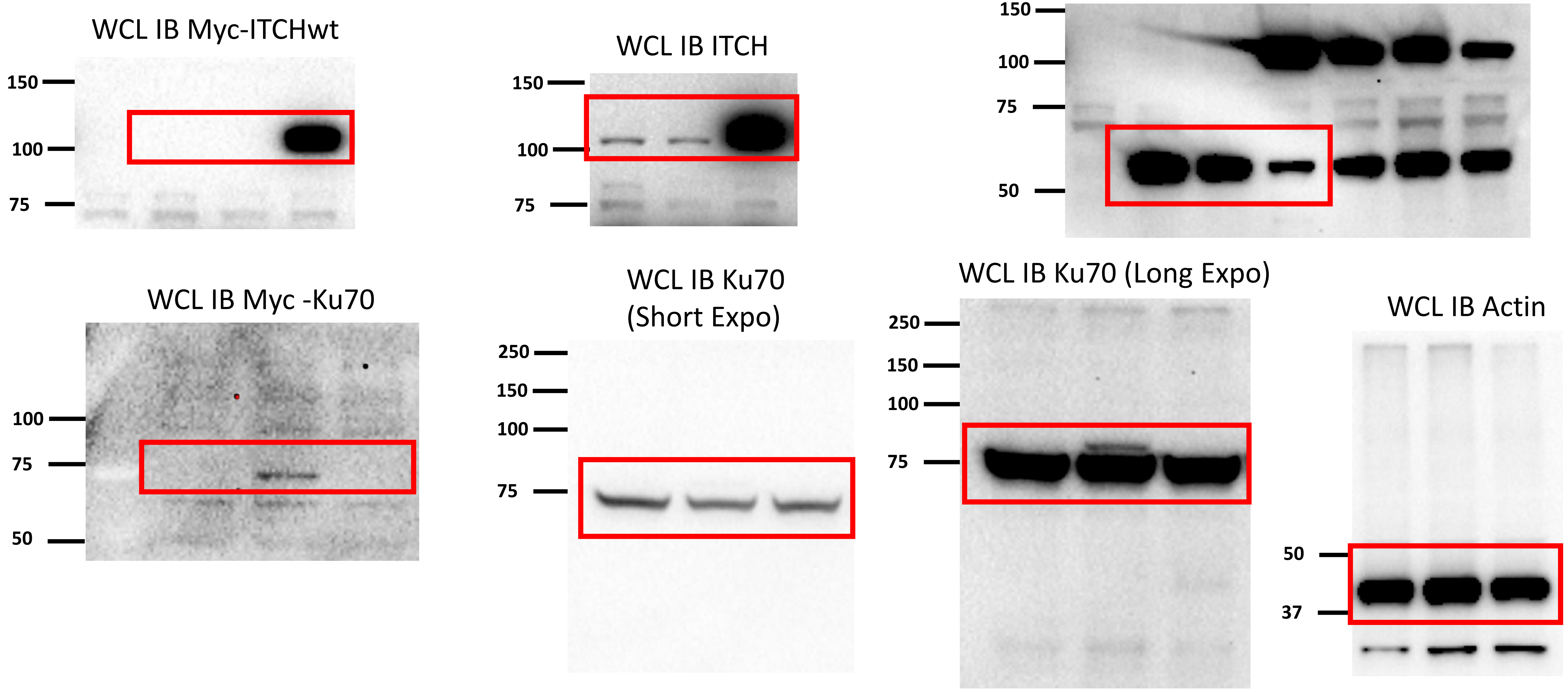

Fig 2H

HEK Myc-ITCHwt

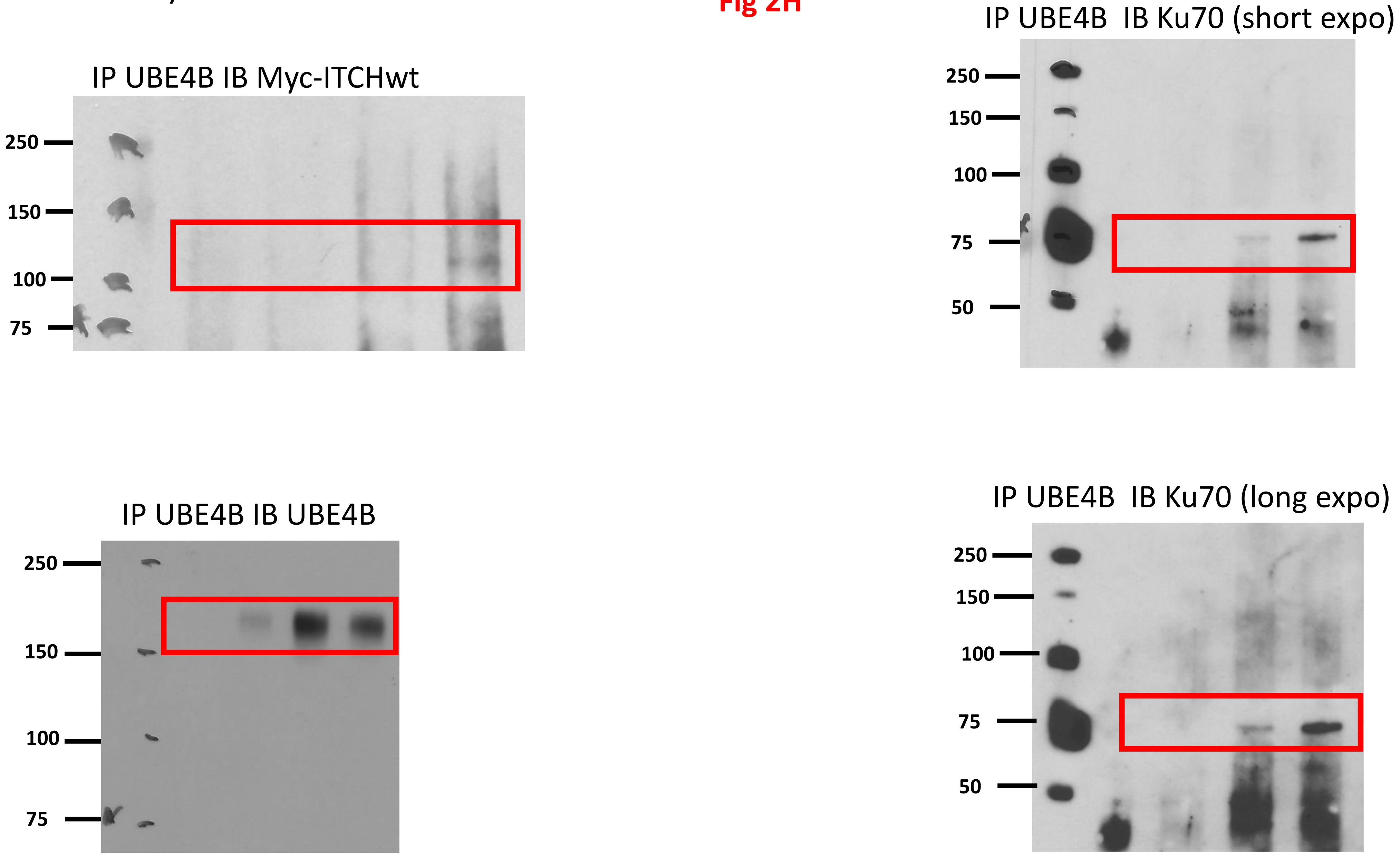

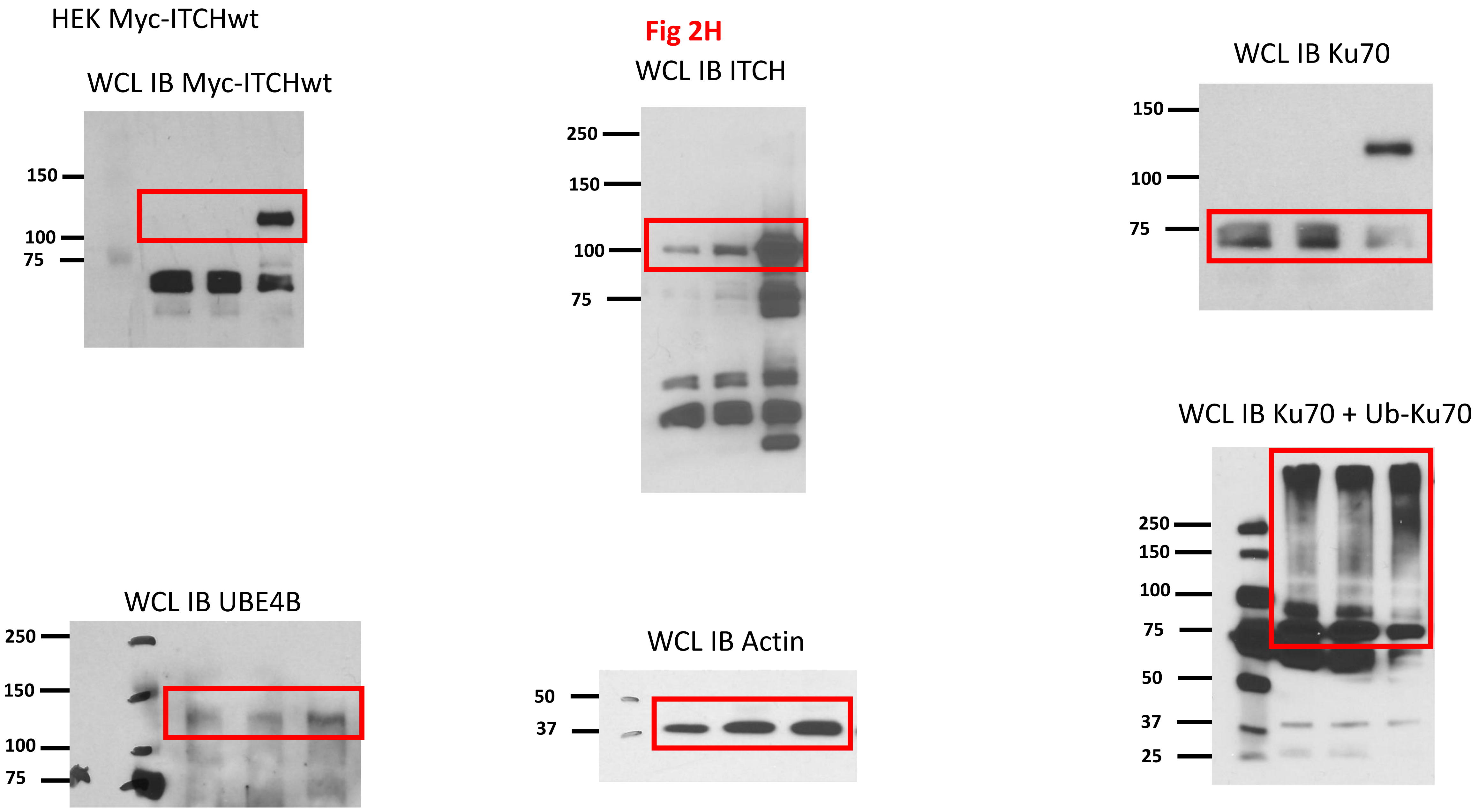
HEK Myc-ITCHwt
Fig 2H
WCL IB Myc-ITCHwt
150
100
75
WCL IB ITCH
250
150
100
75
WCL IB Ku70
150
100
75
WCL IB Ku70 + Ub-Ku70
250
150
100
75
50
37
25
WCL IB UBE4B
250
150
100
75
WCL IB Actin
50
37

SKN-A-S Myc-ITCHwt

Fig 2I

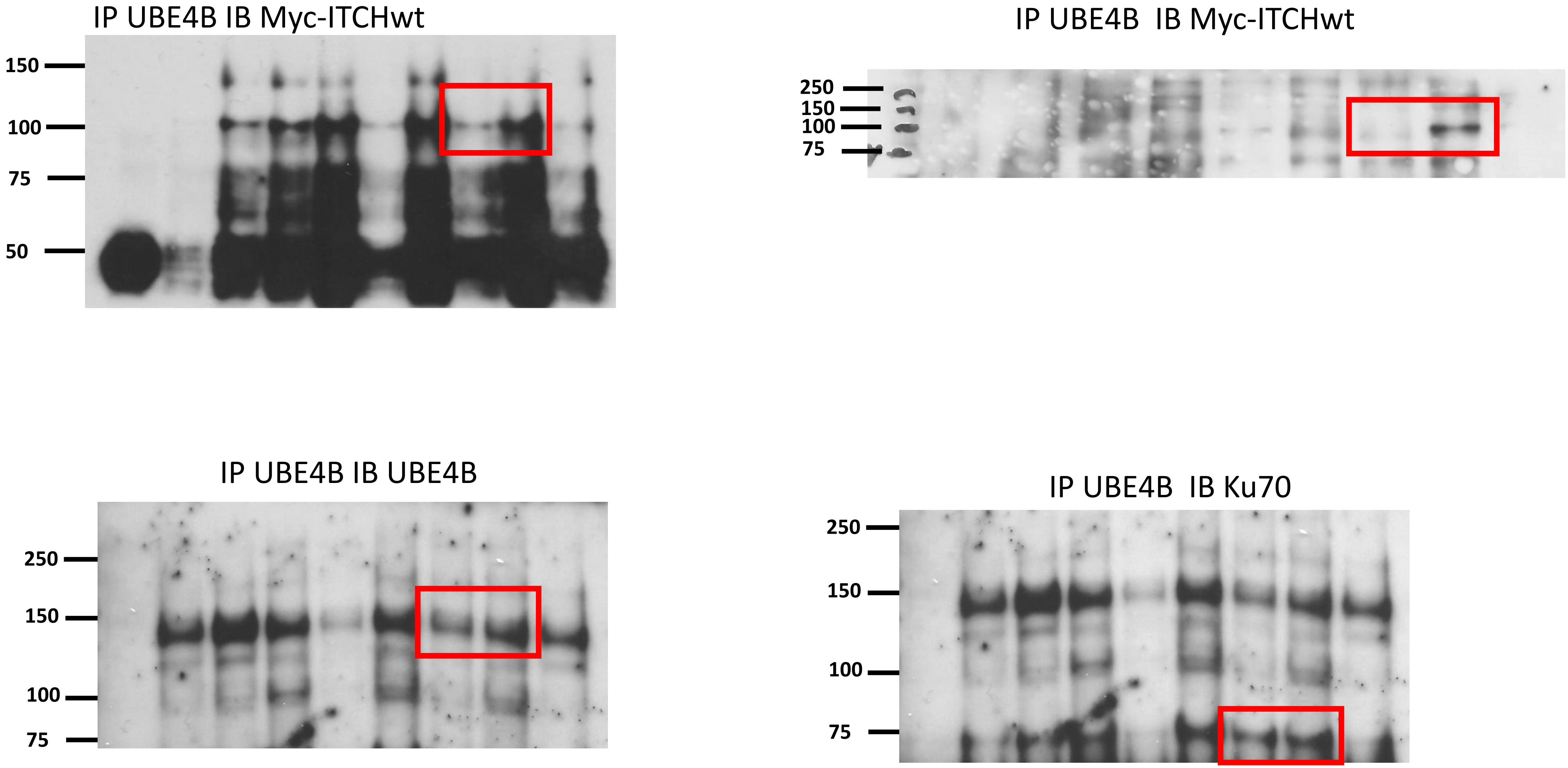

SKN-A-S Myc-ITCHwt

Fig 2I

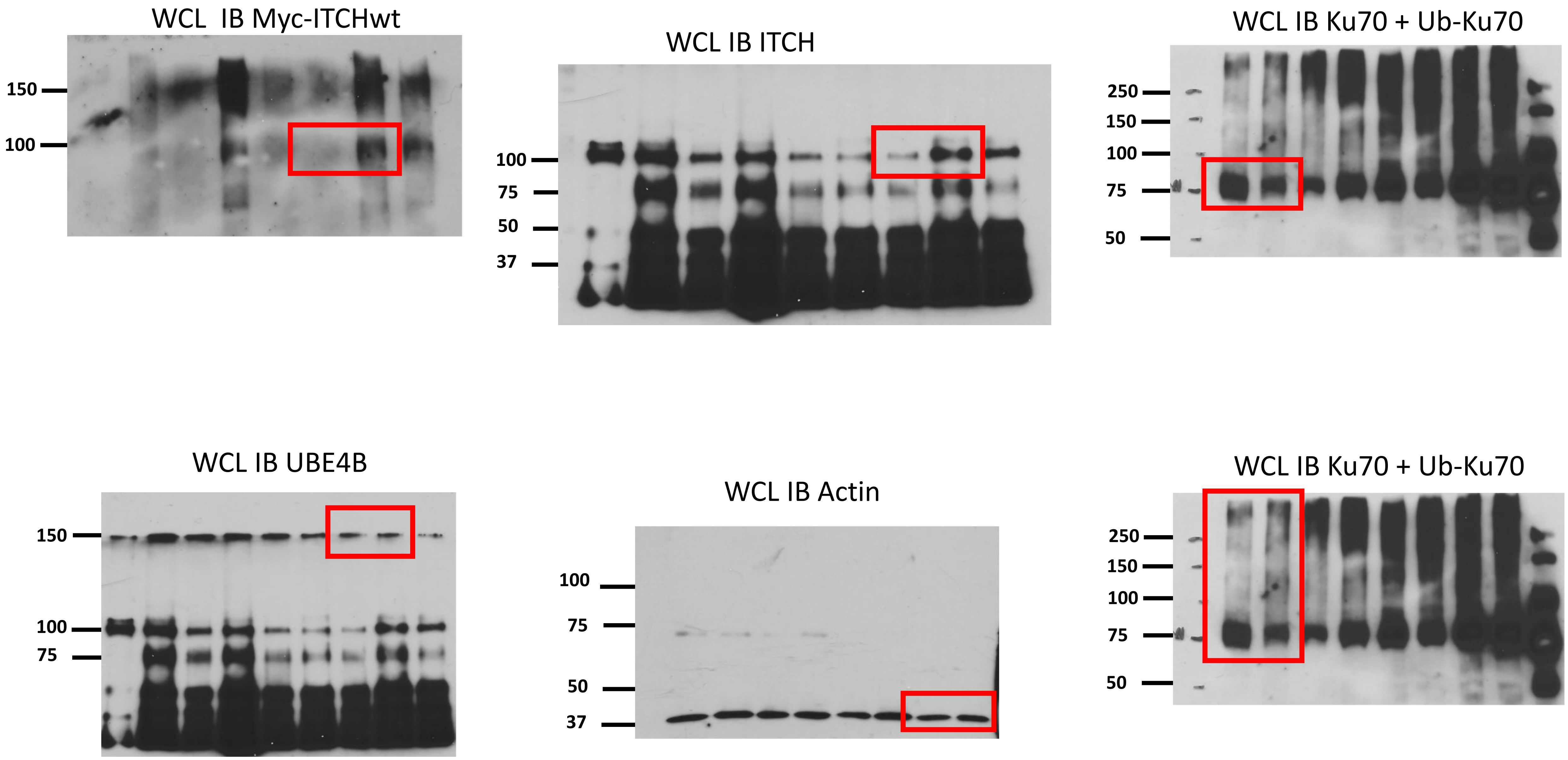

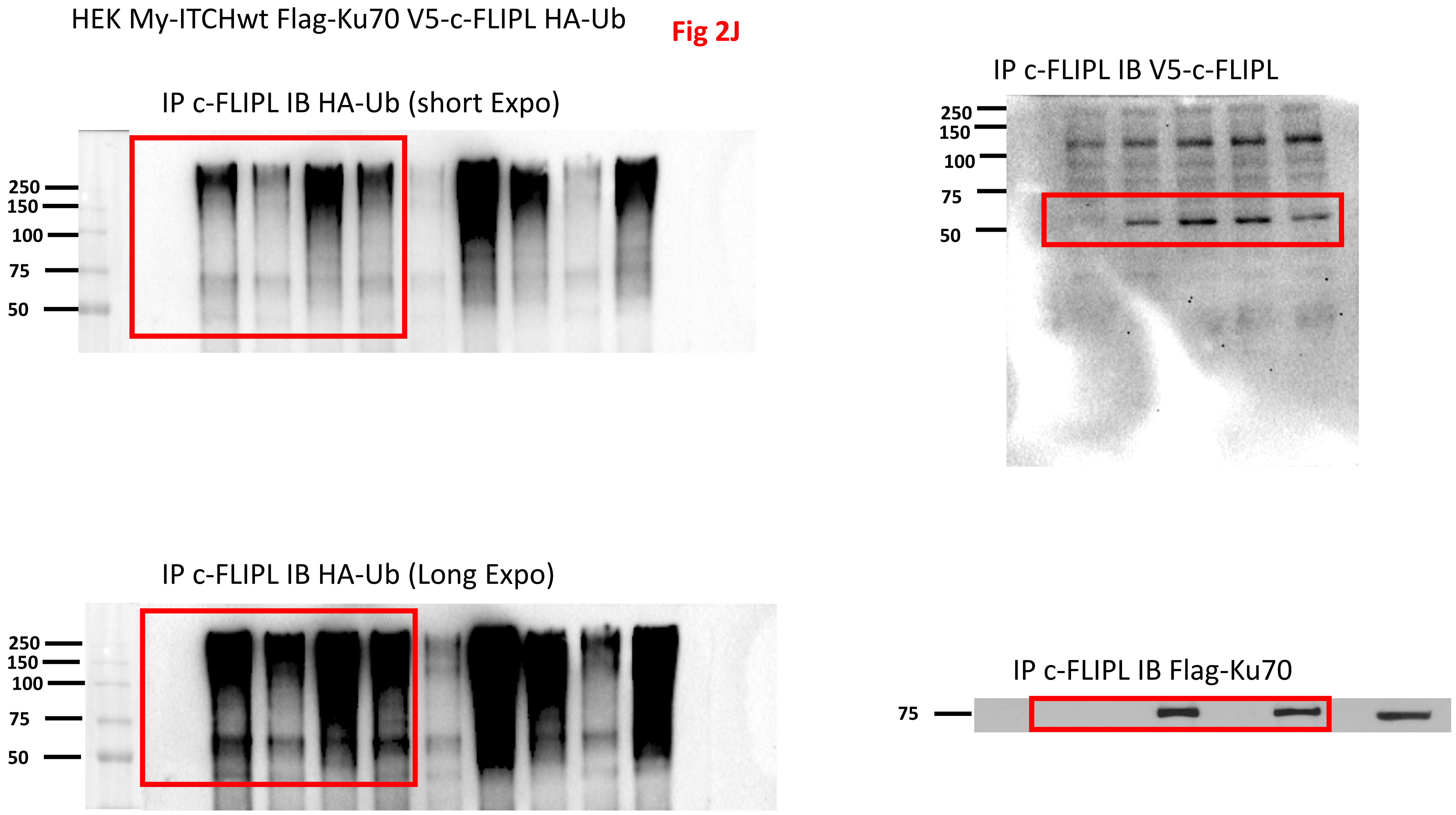

HEK My-ITCHwt Flag-Ku70 V5-c-FLIPL HA-Ub
Fig 2J
IP c-FLIPL IB HA-Ub (short Expo)
250
150
100
75
50
IP c-FLIPL IB V5-c-FLIPL
250
150
100
75
50
IP c-FLIPL IB HA-Ub (Long Expo)
250
150
100
75
50
IP c-FLIPL IB Flag-Ku70
75

HEK My-ITCHwt Flag-Ku70 V5-c-FLIPL HA-Ub

Fig 2J

IP UBE4B IB Myc-ITCHwt

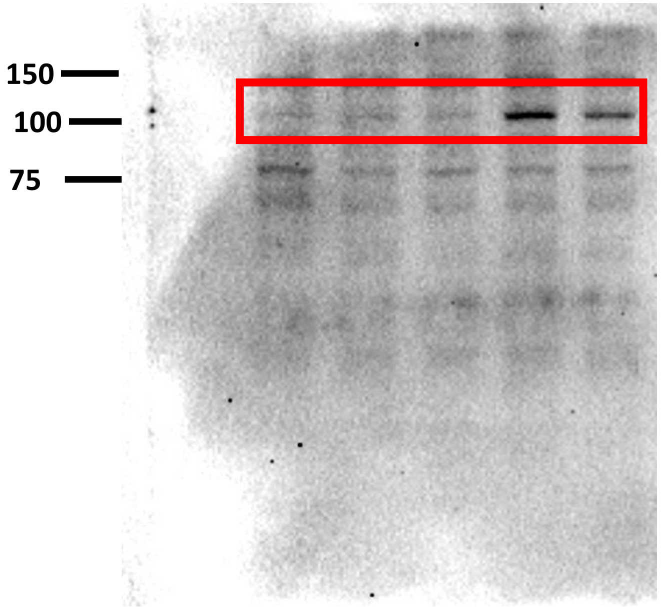

IP UBE4B IB UBE4B

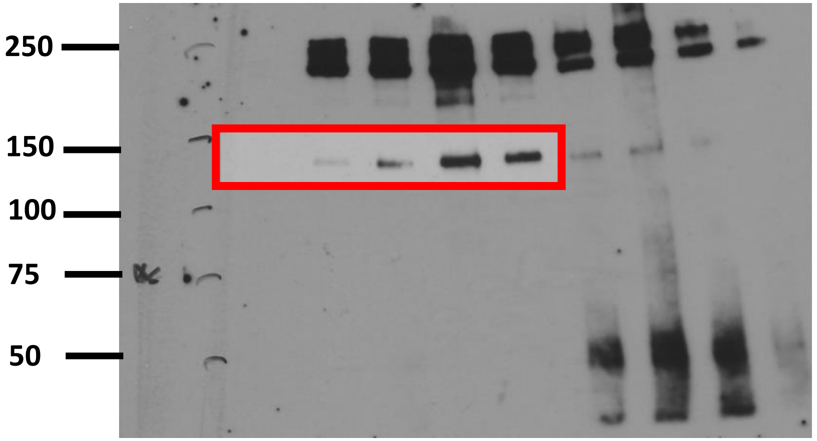

HEK My-ITCHwt Flag-Ku70 V5-c-FLIPL HA-Ub

Fig 2J

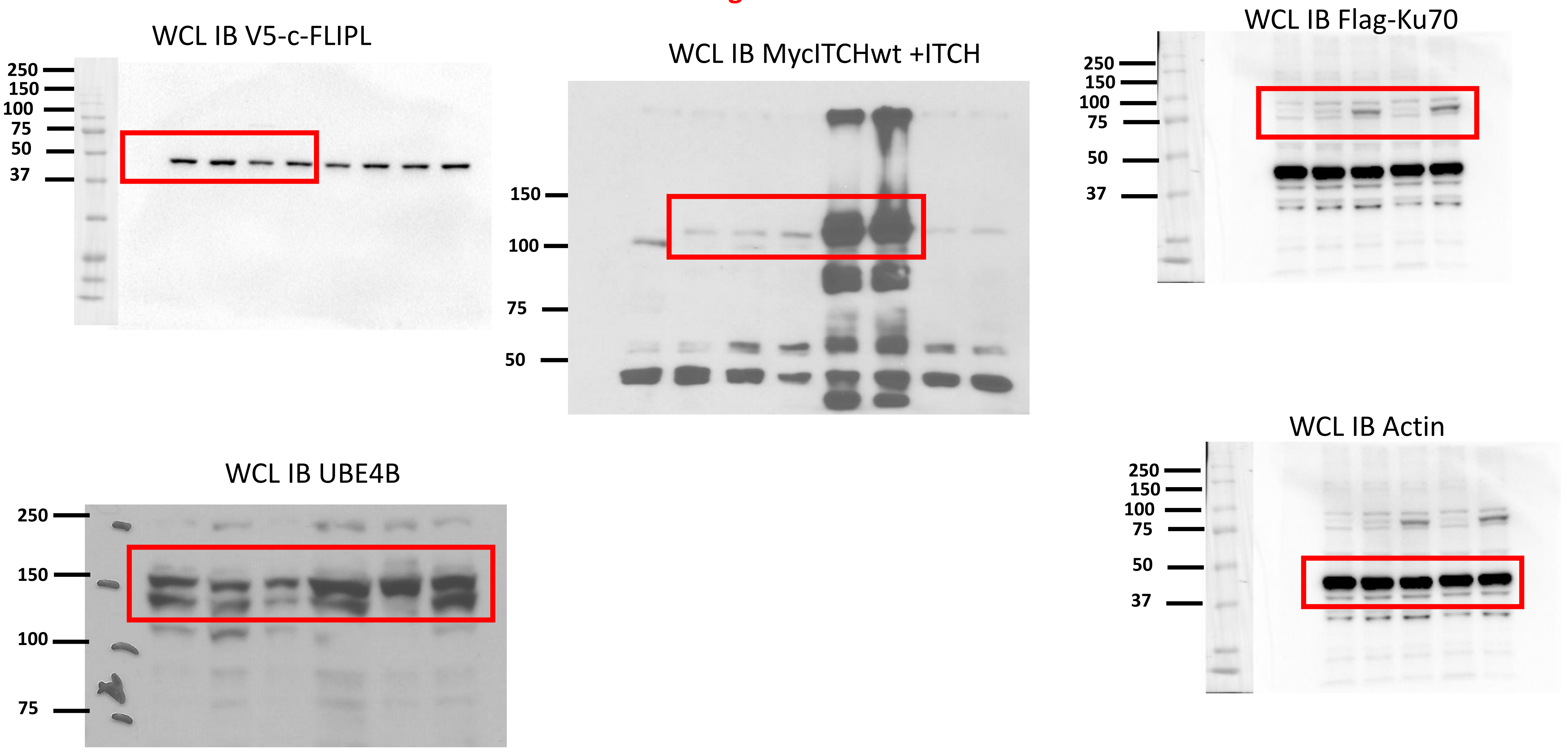

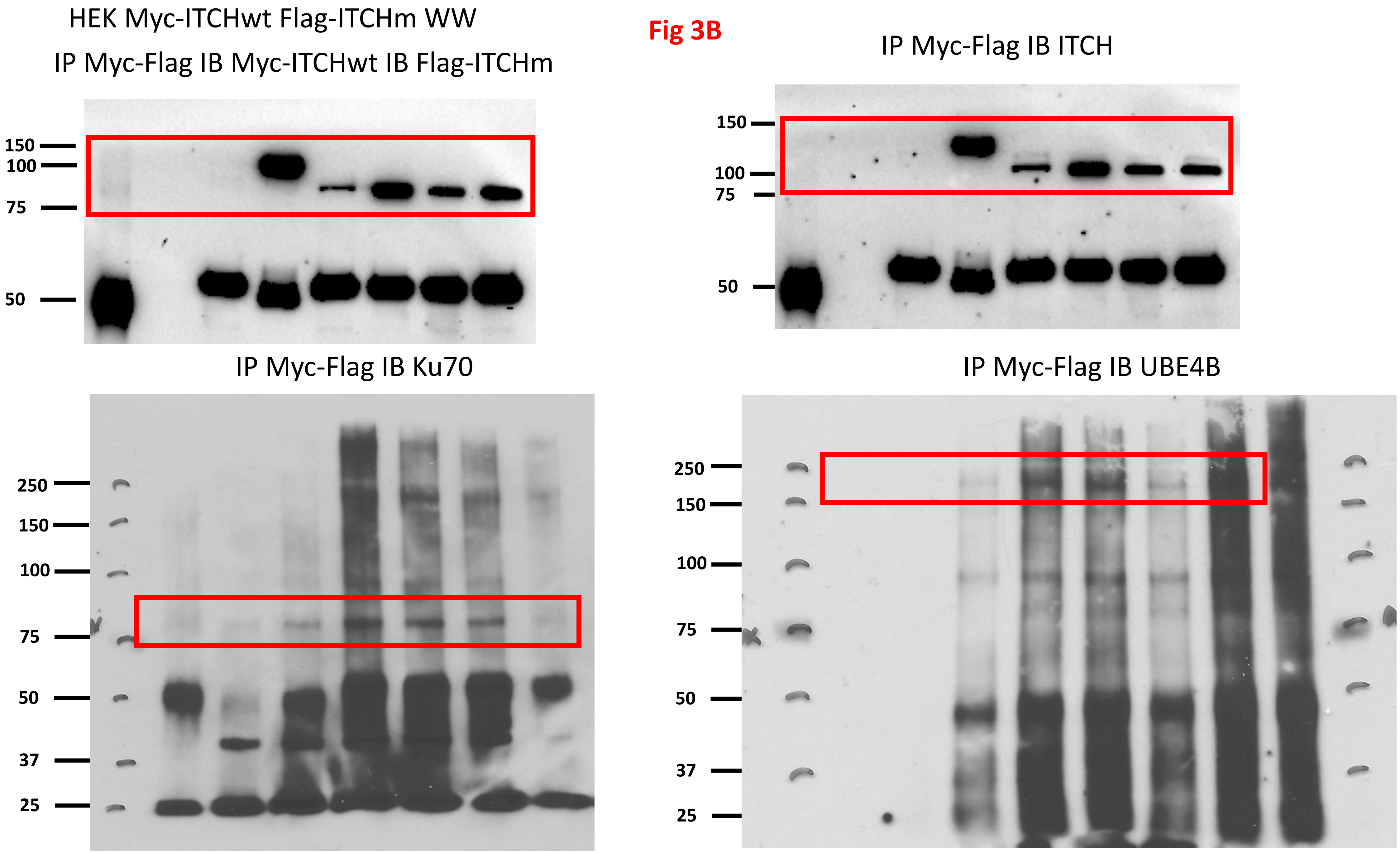
HEK Myc-ITCHwt Flag-ITCHm WW
Fig 3B
IP Myc-Flag IB Myc-ITCHwt IB Flag-ITCHm
150
100
75
50
IP Myc-Flag IB ITCH
150
100
75
50
IP Myc-Flag IB Ku70
250
150
100
75
50
37
25
IP Myc-Flag IB UBE4B
250
150
100
75
50
37
25

HEK Myc-ITCHwt Flag-ITCHm WW

Fig 3B

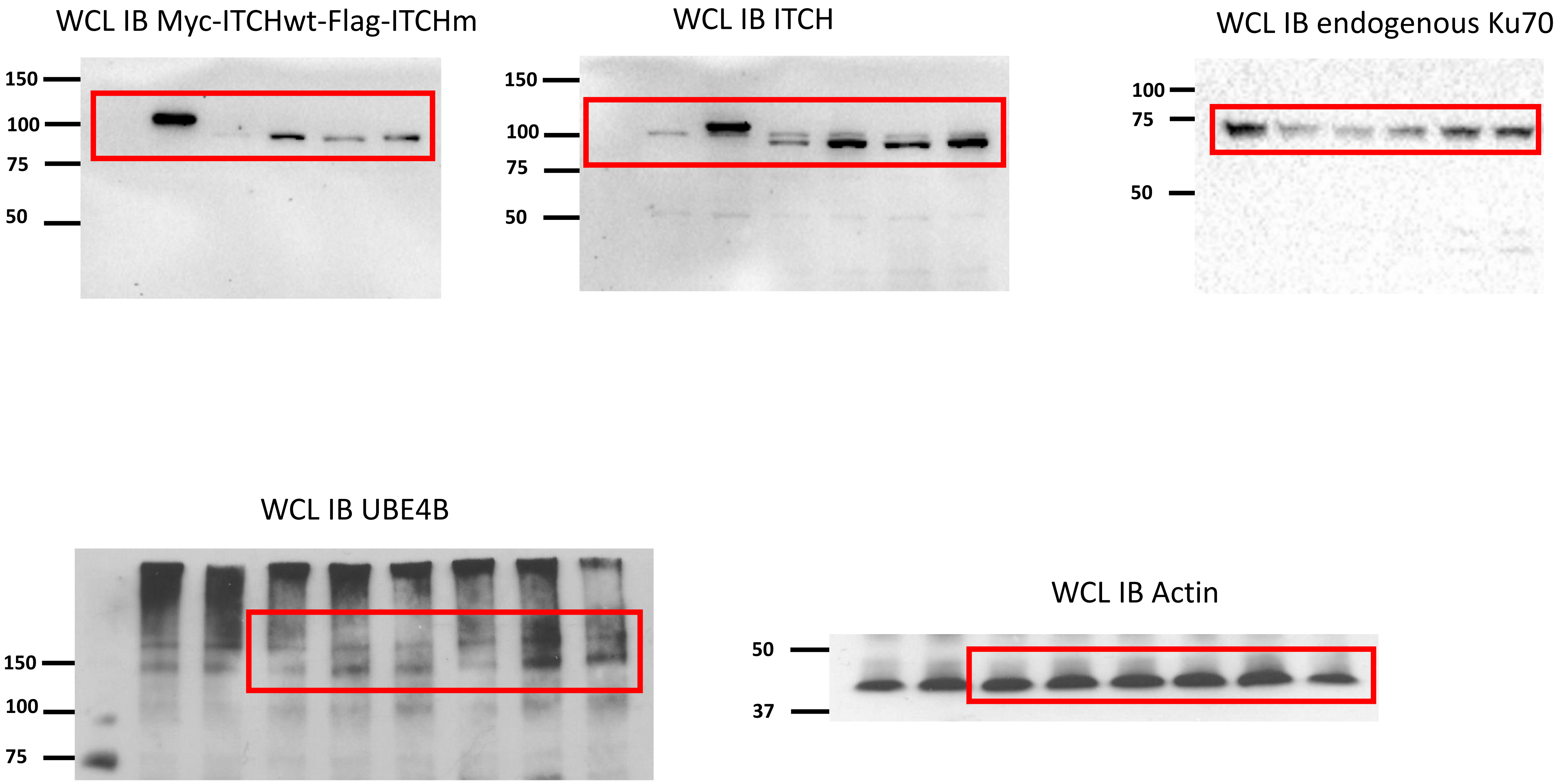

HEK Flag-c-FLIPL Myc-ITCHwt Flag-ITCHm WW + MG

Fig 3C

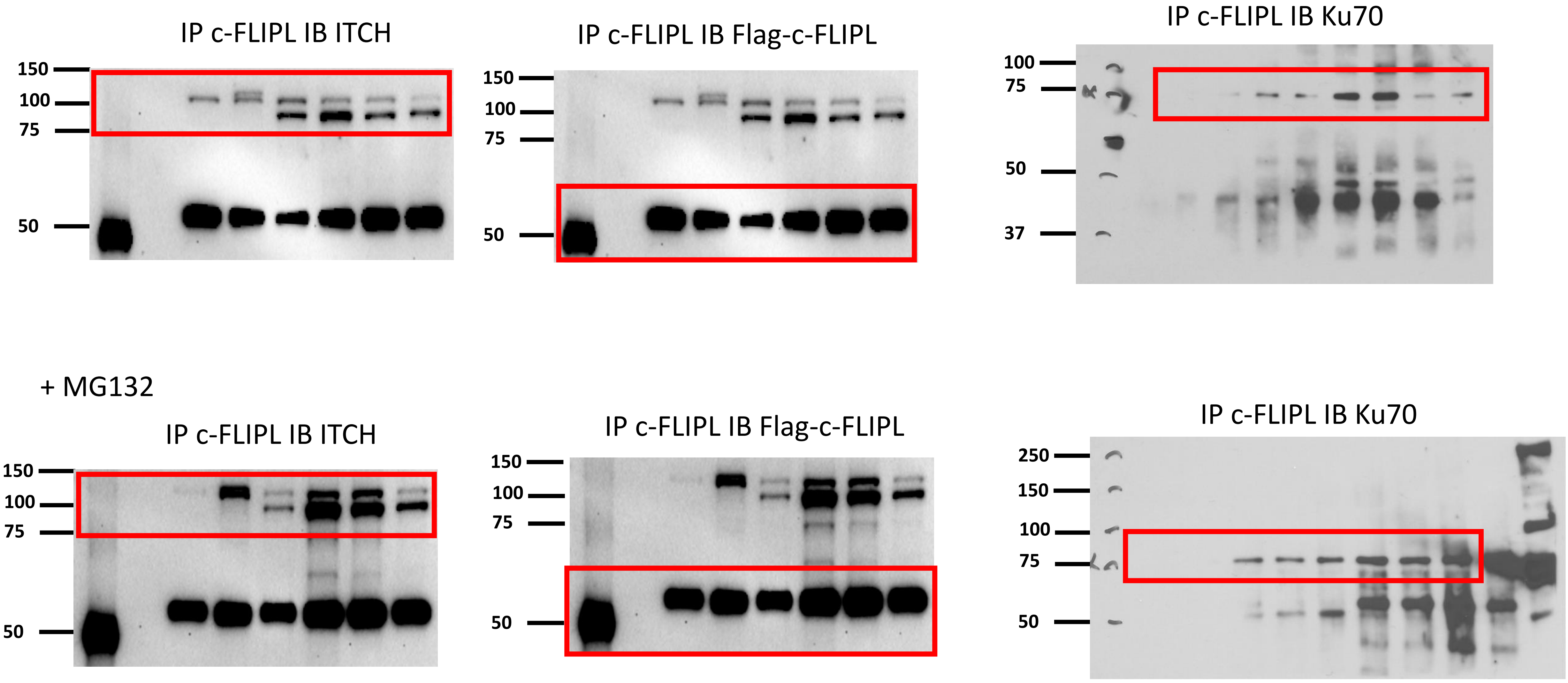

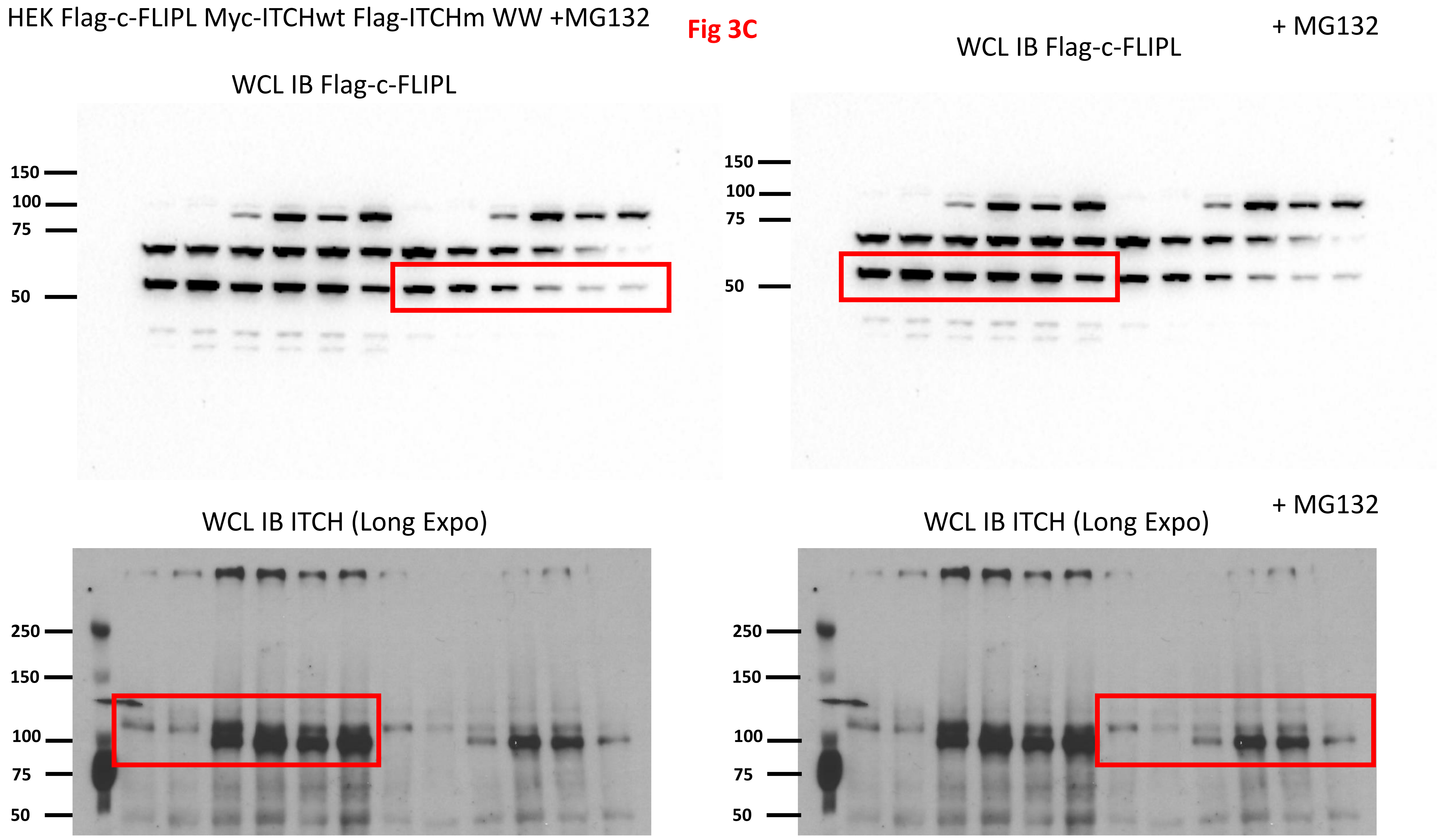
HEK Flag-c-FLIPL Myc-ITCHwt Flag-ITCHm WW +MG132
Fig 3C
WCL IB Flag-c-FLIPL
150
100
75
50
+ MG132
WCL IB Flag-c-FLIPL
150
100
75
50
WCL IB ITCH (Long Expo)
250
150
100
75
50
+ MG132
WCL IB ITCH (Long Expo)
250
150
100
75
50

HEK Flag-c-FLIPL Myc-ITCHwt Flag-ITCHm WW +MG132

Fig 3C

WCL IB endogenous Ku70

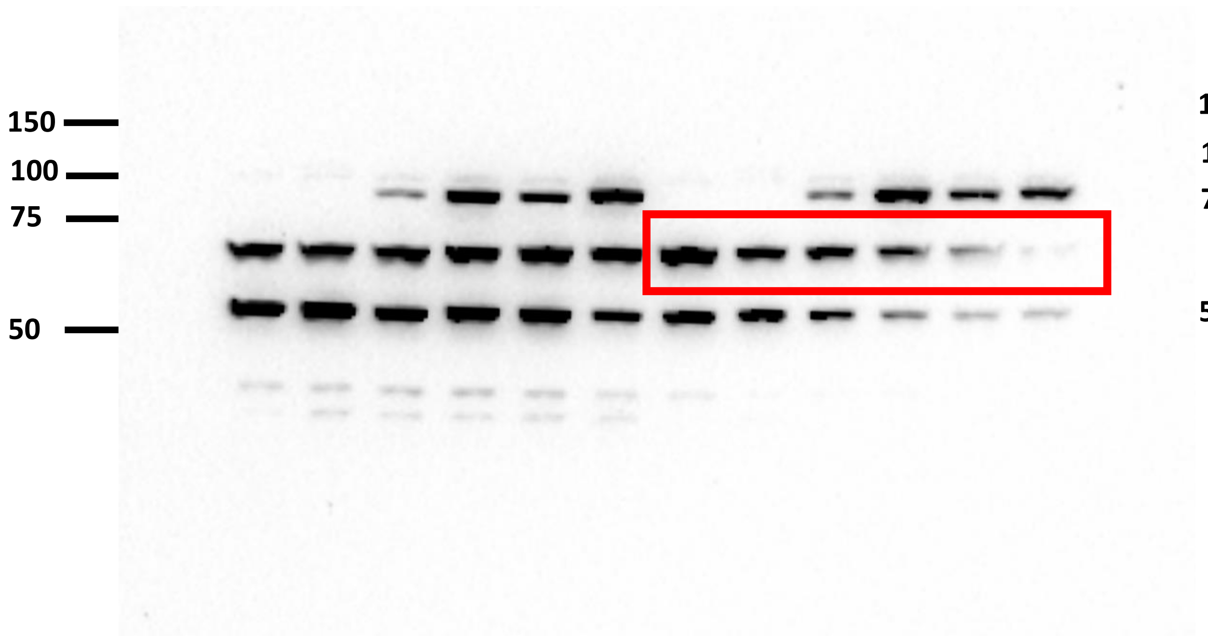

+ MG132

WCL IB endogenous Ku70

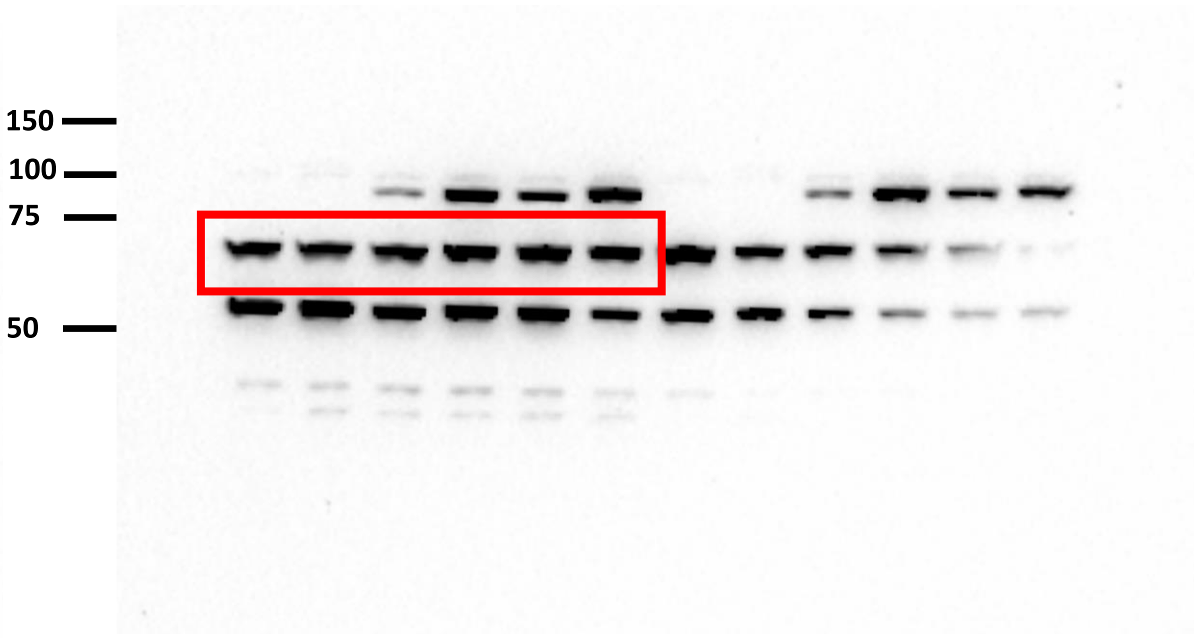

HEK Flag-c-FLIPL Myc-ITCHwt Flag-ITCHm WW +MG132

Fig 3C

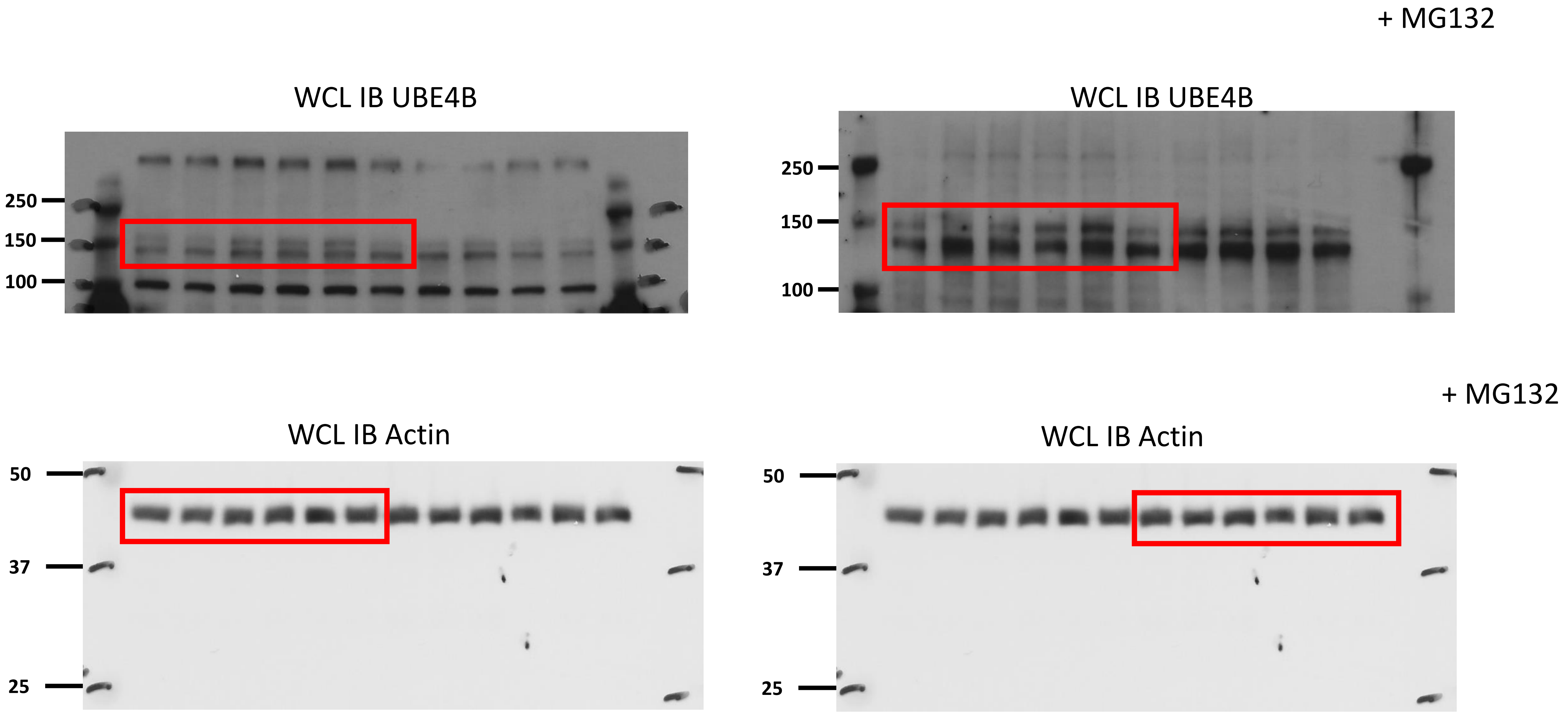

HEK Flag-c-FLIPL Myc-ITCHwt Flag-ITCHm WW

Fig 3D

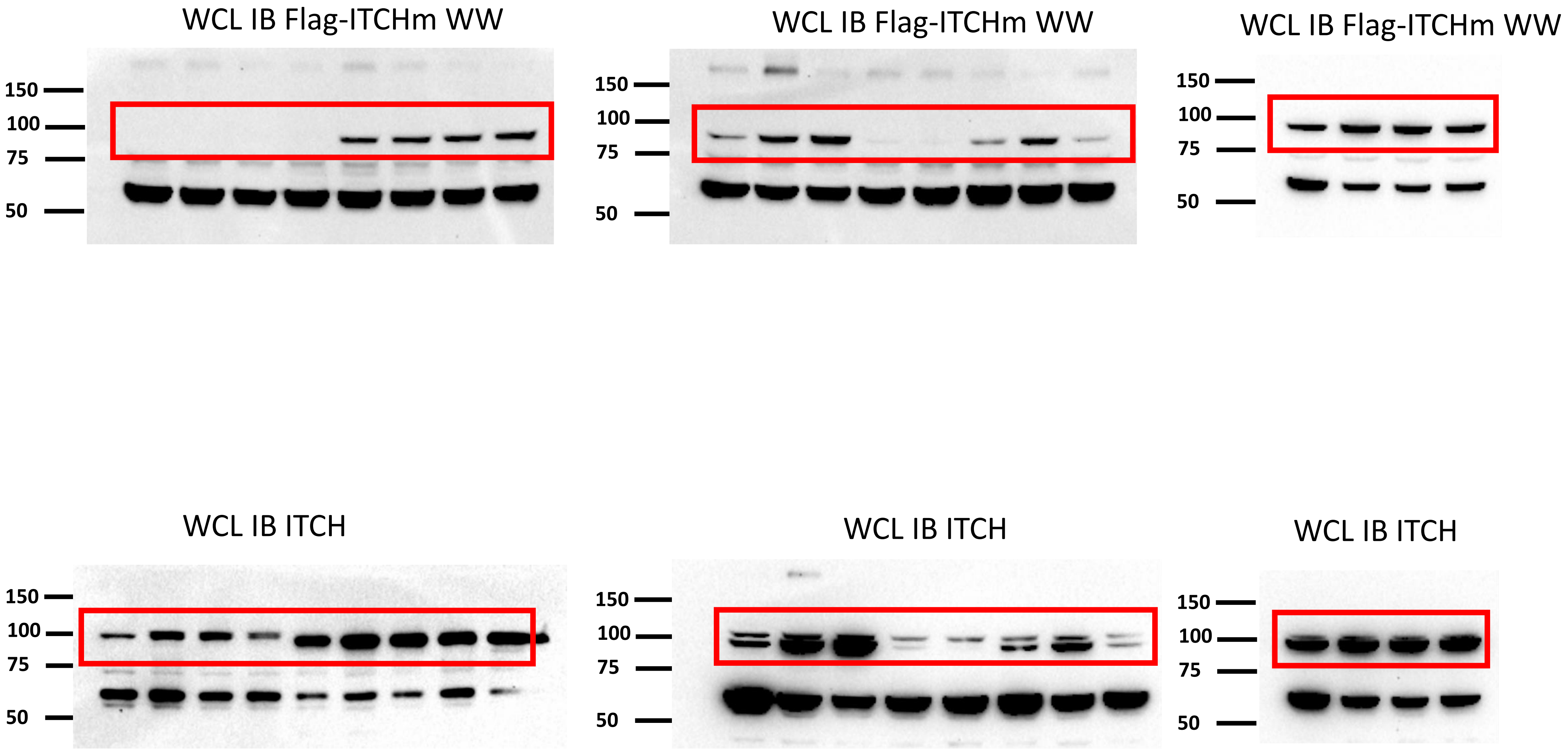

HEK Flag-c-FLIPL Myc-ITCHwt Flag-ITCHm WW

Fig 3D

WCL IB Ku70 (longer Expo)

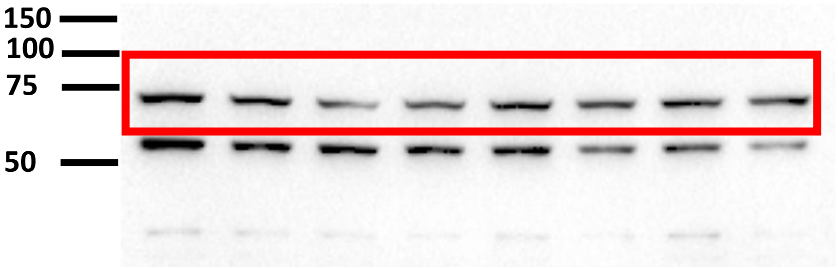

WCL IB Ku70 (longer Expo)

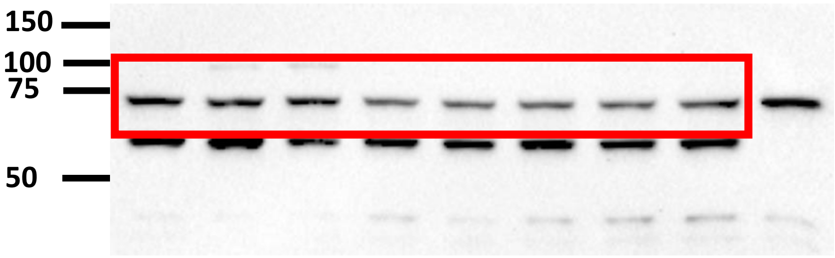

WCL IB Ku70 (Longer Expo)

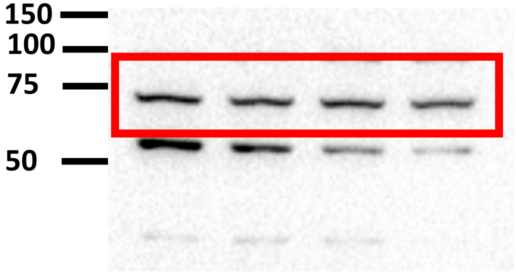

WCL IB Ku70 (short Expo)

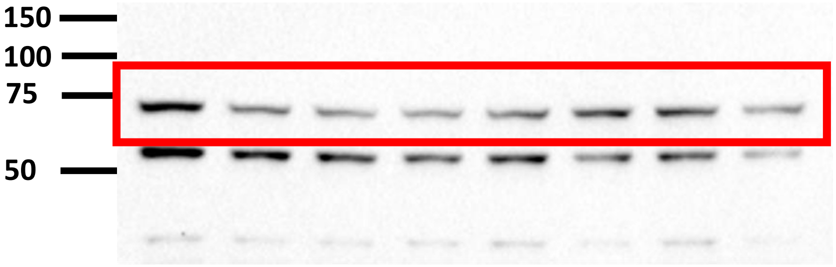

WCL IB Ku70 (short Expo)

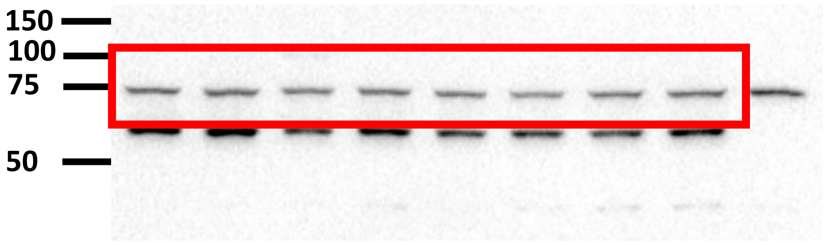

WCL IB Ku70 (short Expo)

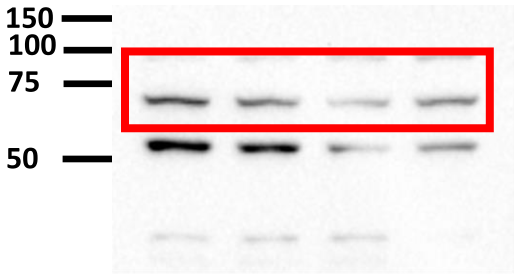

HEK Flag-c-FLIPL Myc-ITCHwt Flag-ITCHm WW

Fig 3D

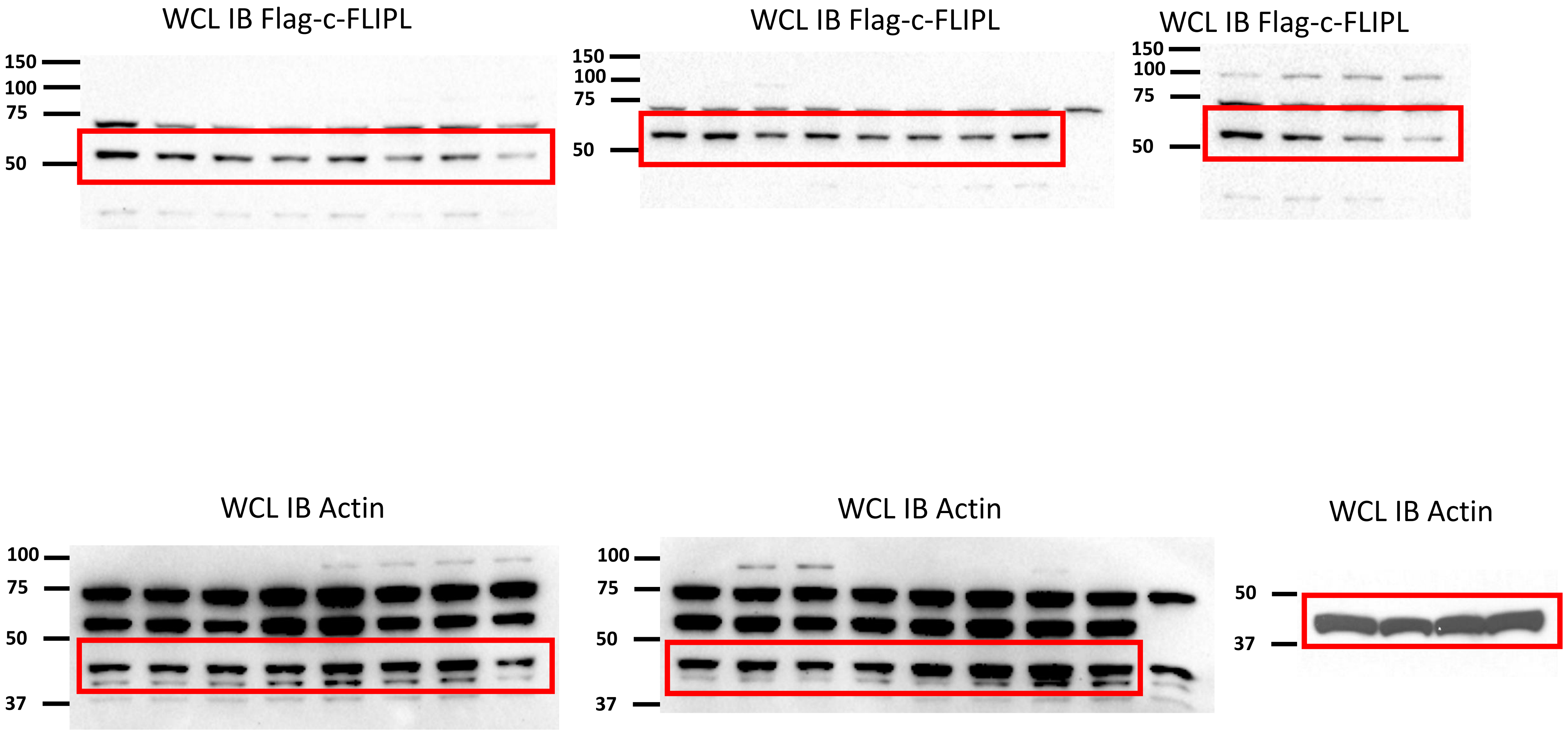

HEK Flag-c-FLIPL Myc-ITCHwt Flag-ITCHm WW +MG132

Fig 3E

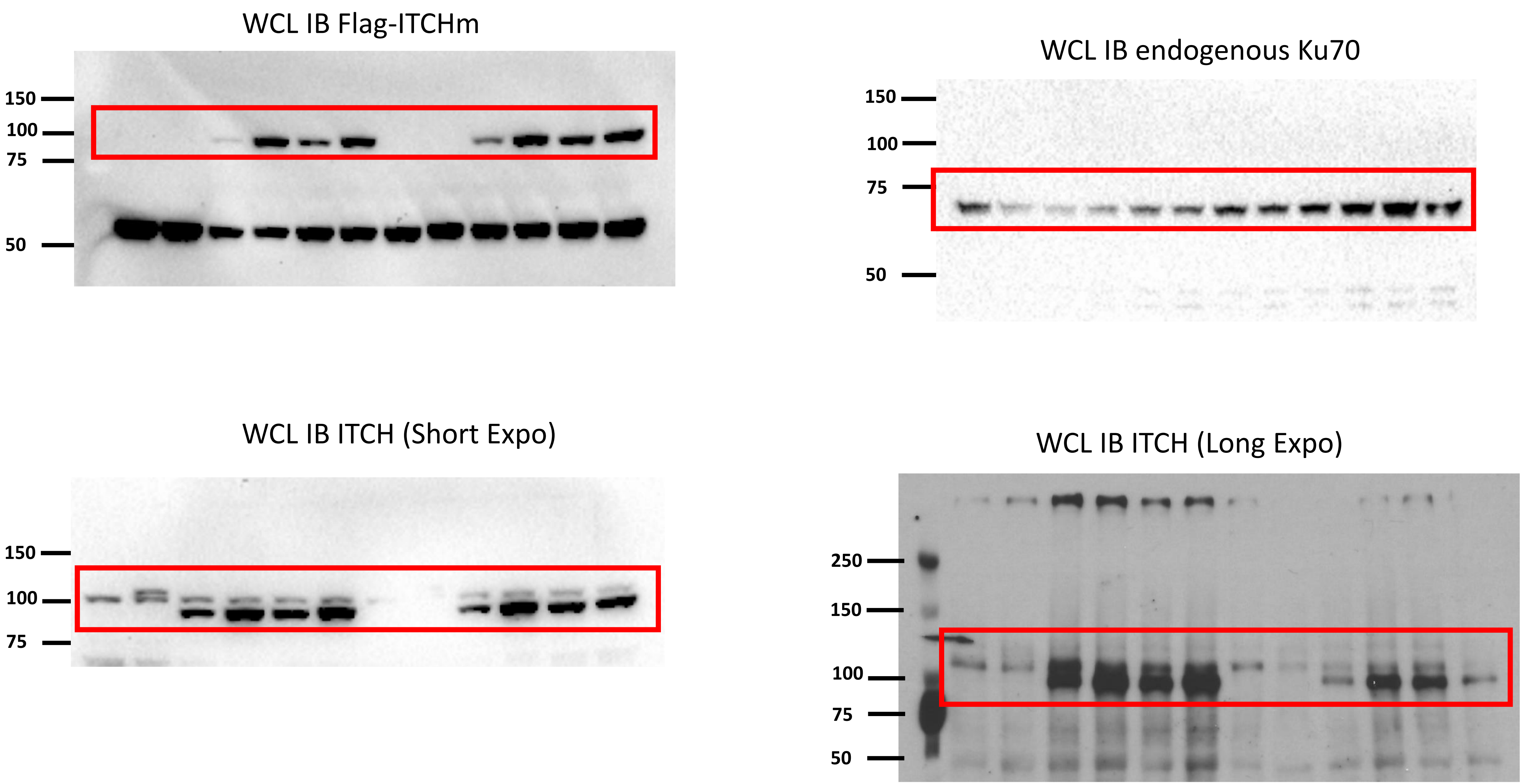

HEK Flag-c-FLIPL Myc-ITCHwt Flag-ITCHm WW +MG132

Fig 3E

WCL IB Flag-c-FLIPL

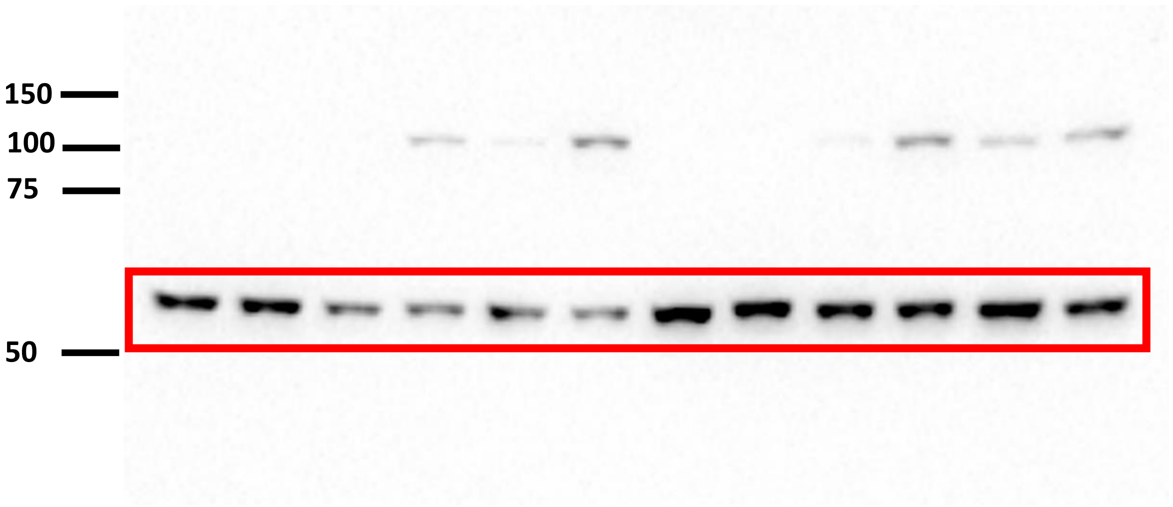

WCL IB Actin

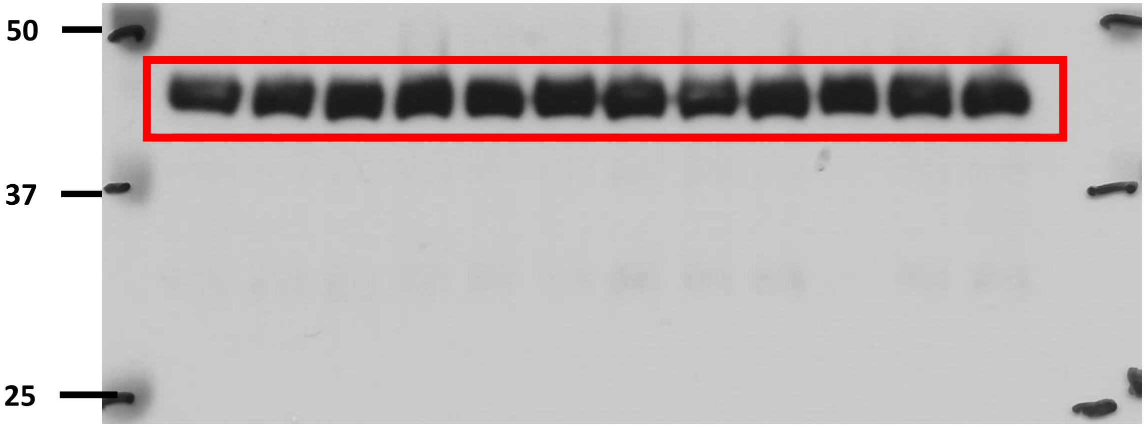

Fig 4A

HEK Myc-ITCHm S199D Flag-Ku70

IP ITCH IB Flag-Ku70

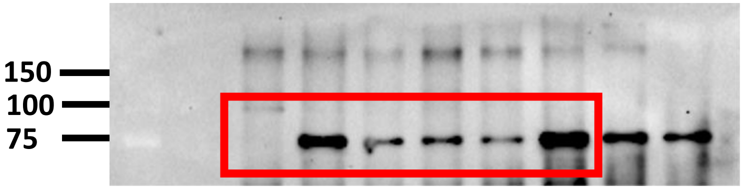

IP ITCH IB Myc-ITCH

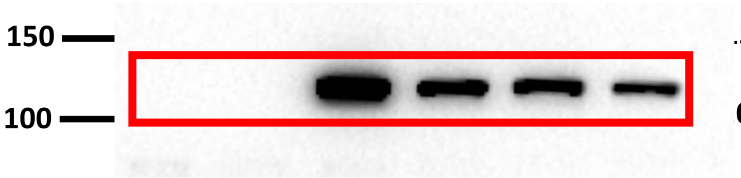

IP ITCH IB ITCH

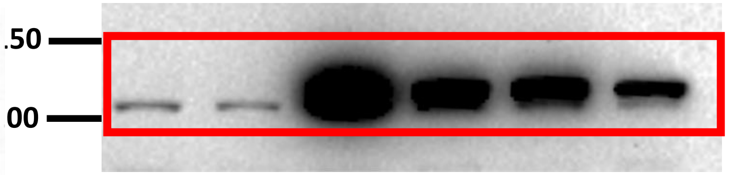

IP ITCH IB UBE4B

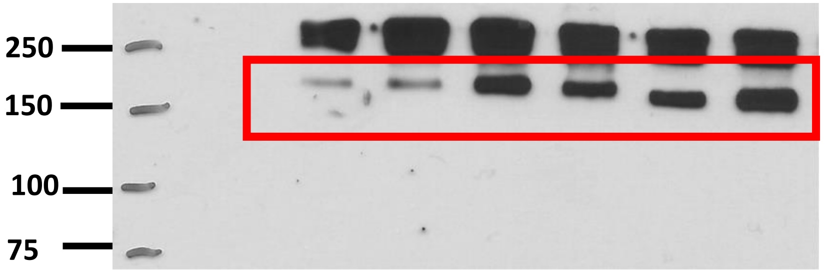

Fig 4A

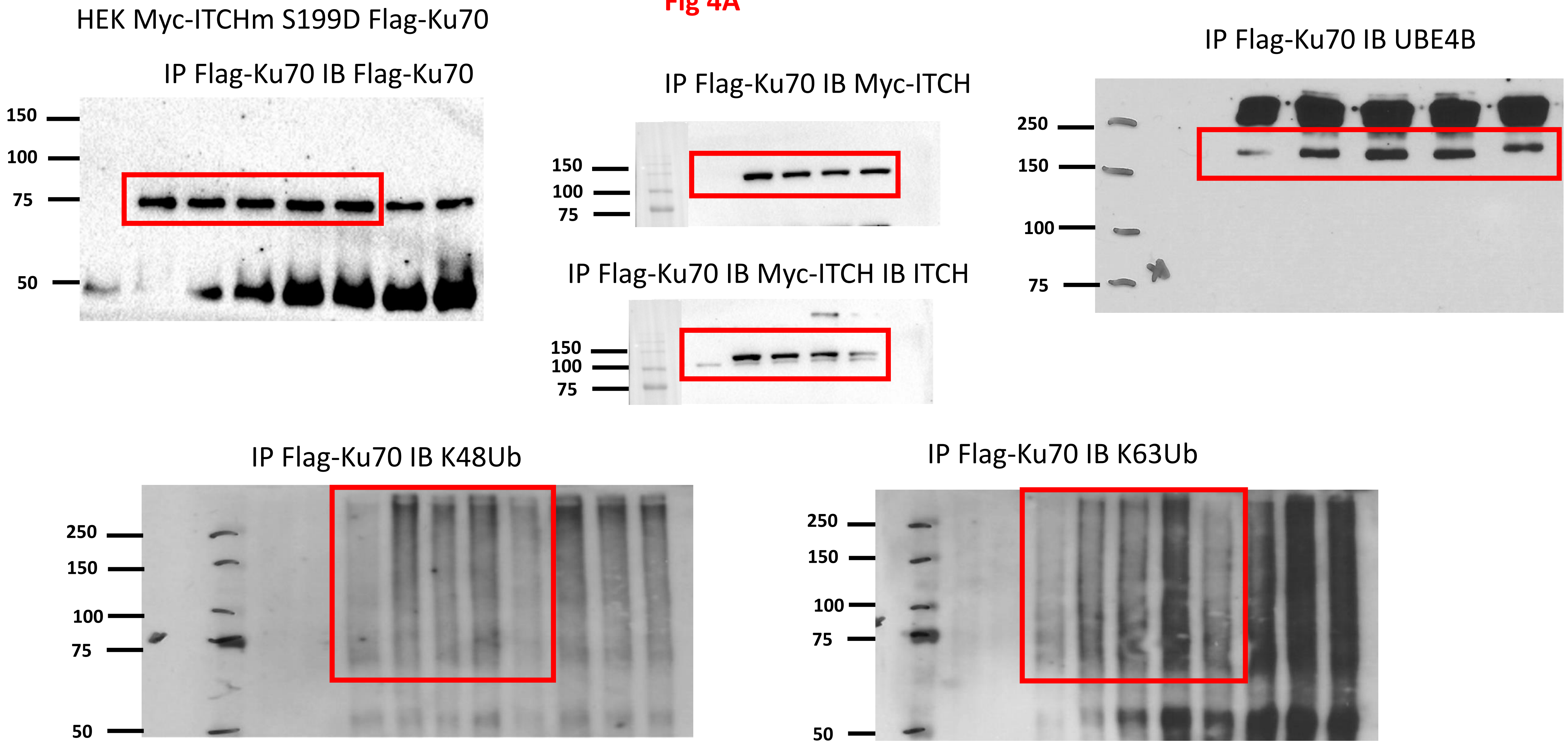

HEK Myc-ITCHm S199D Flag-Ku70
IP Flag-Ku70 IB Flag-Ku70
150
100
75
50
IP Flag-Ku70 IB Myc-ITCH
150
100
75
IP Flag-Ku70 IB Myc-ITCH IB ITCH
150
100
75
IP Flag-Ku70 IB UBE4B
250
150
100
75
IP Flag-Ku70 IB K48Ub
250
150
100
75
50
IP Flag-Ku70 IB K63Ub
250
150
100
75
50

Fig 4A

HEK Myc-ITCHm S199D Flag-Ku70 WCL

WCL IB Flag-Ku70 (short Expo)

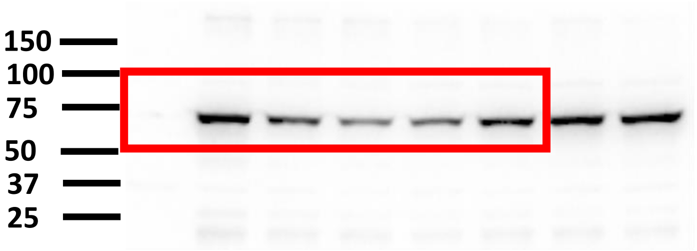

WCL IB Flag-Ku70 (Long Expo)

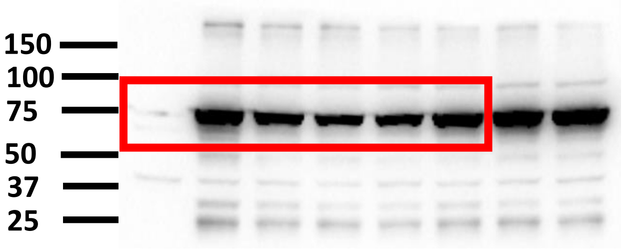

WCL Flag-Ku70 +endogenous Ku70

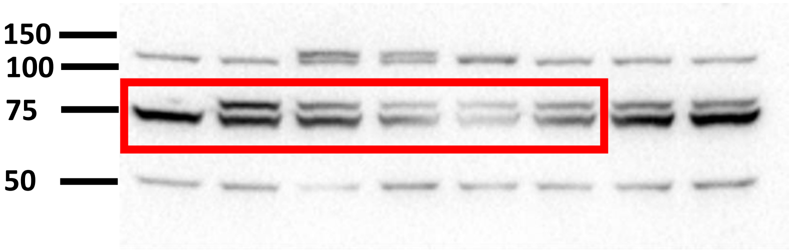

WCL IB UBE4B

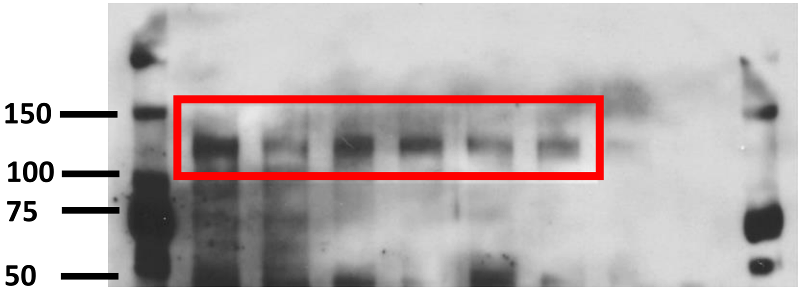

WCL IB ITCH

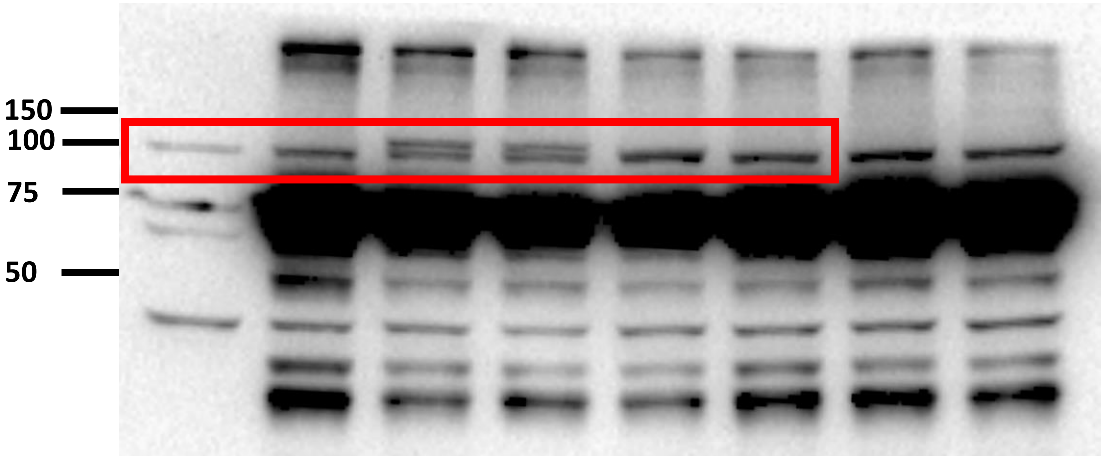

Fig 4B

HEK Myc-ITCHm S199D Flag-Ku70 Flag-c-FLIPL

IP c-FLIP IB Flag-c-FLIPL

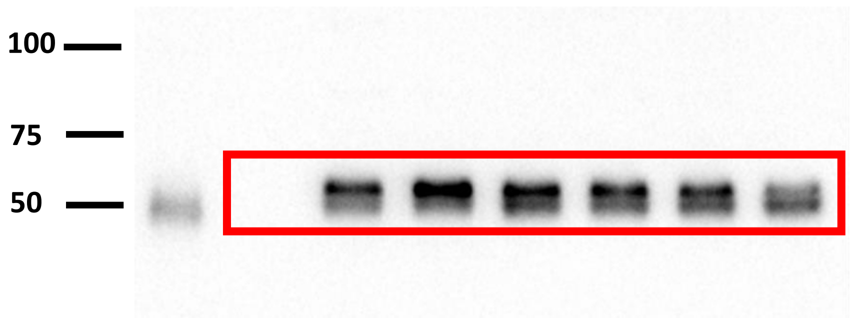

IP c-FLIP IB Myc-ITCH

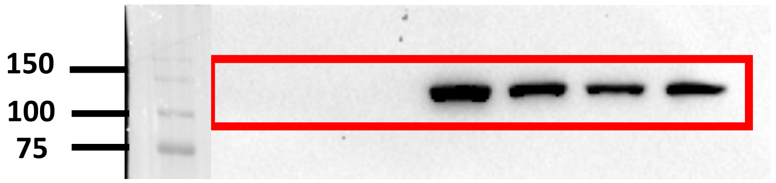

IP c-FLIPL IB Flag-Ku70

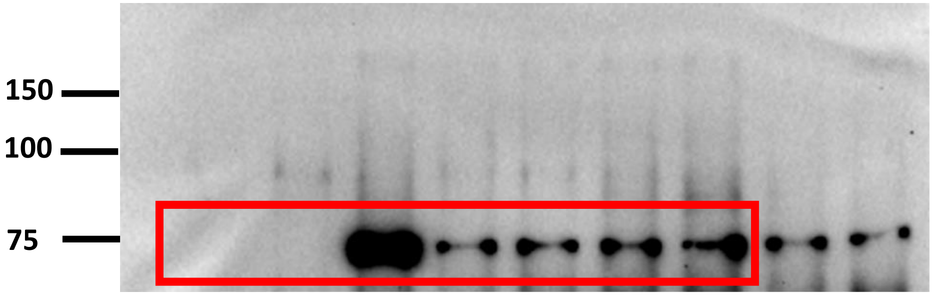

IP c-FLIPL IB UBE4B

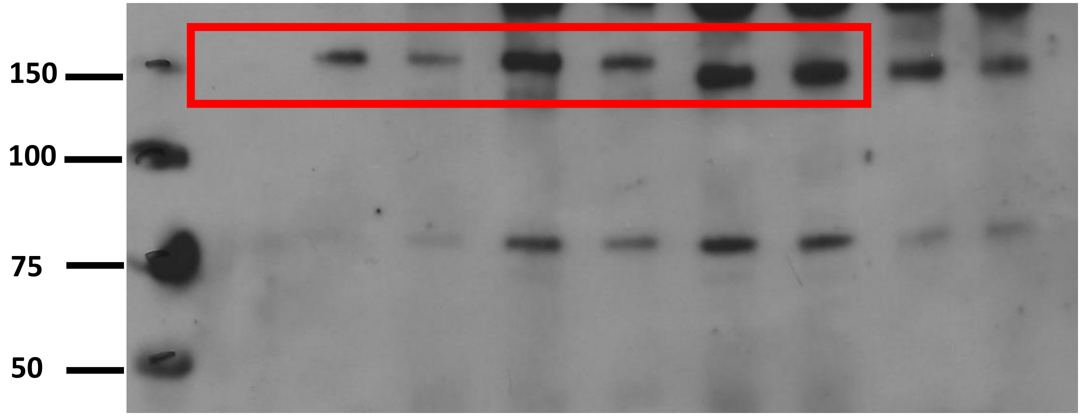

Fig 4B

HEK Myc-ITCHm S199D Flag-Ku70

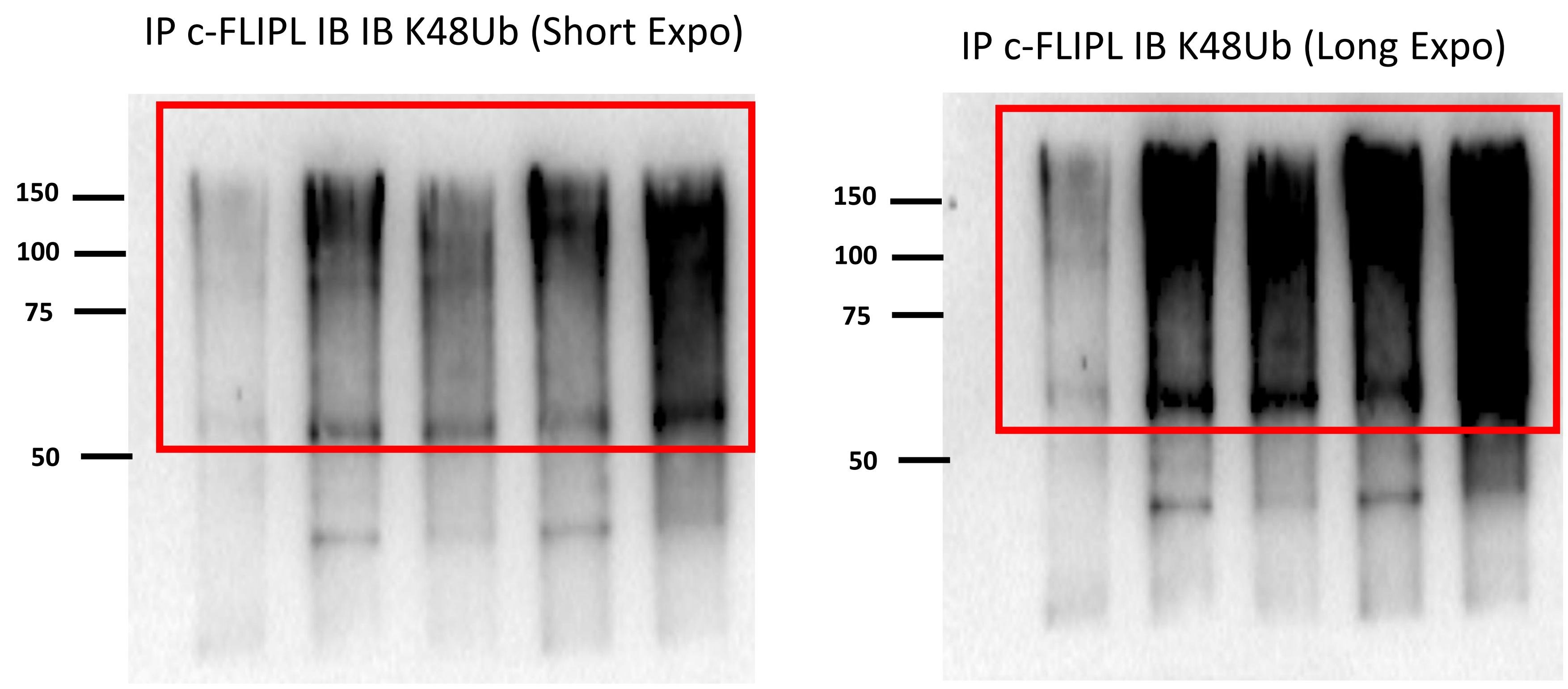

Fig 4B

HEK Myc-ITCHm S199D Flag-Ku70

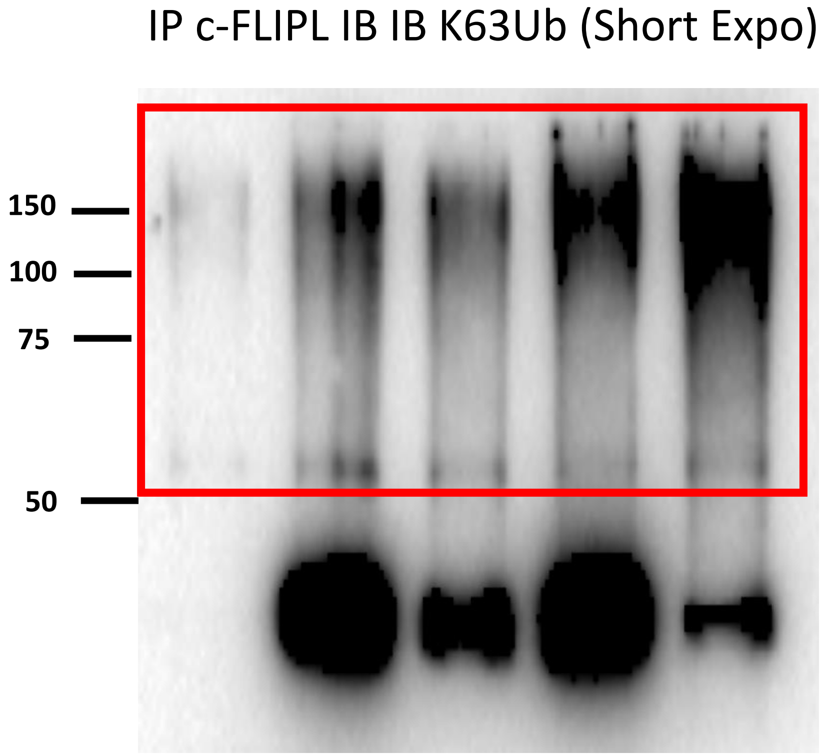

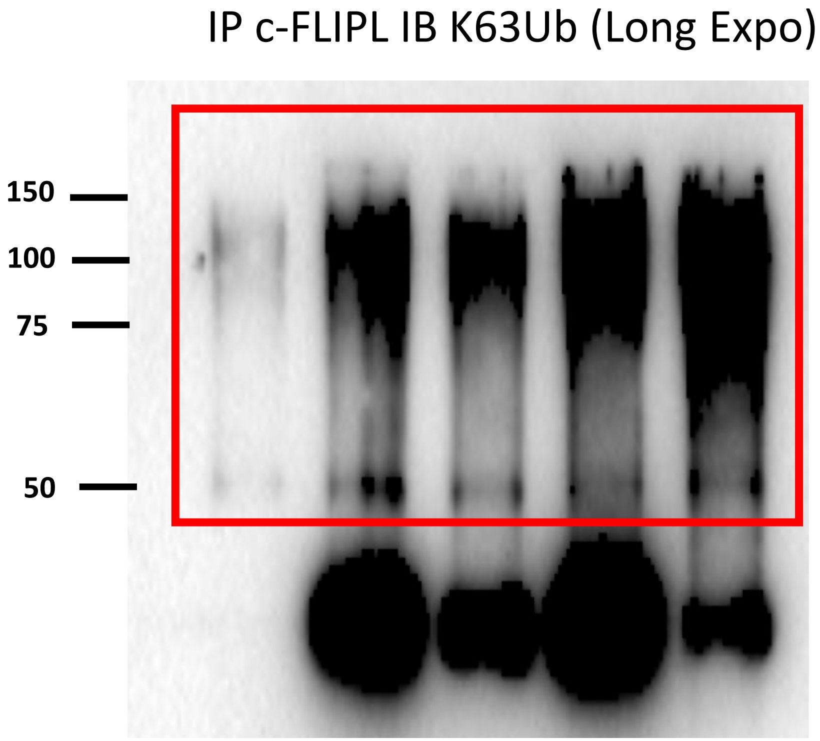

Fig 4A

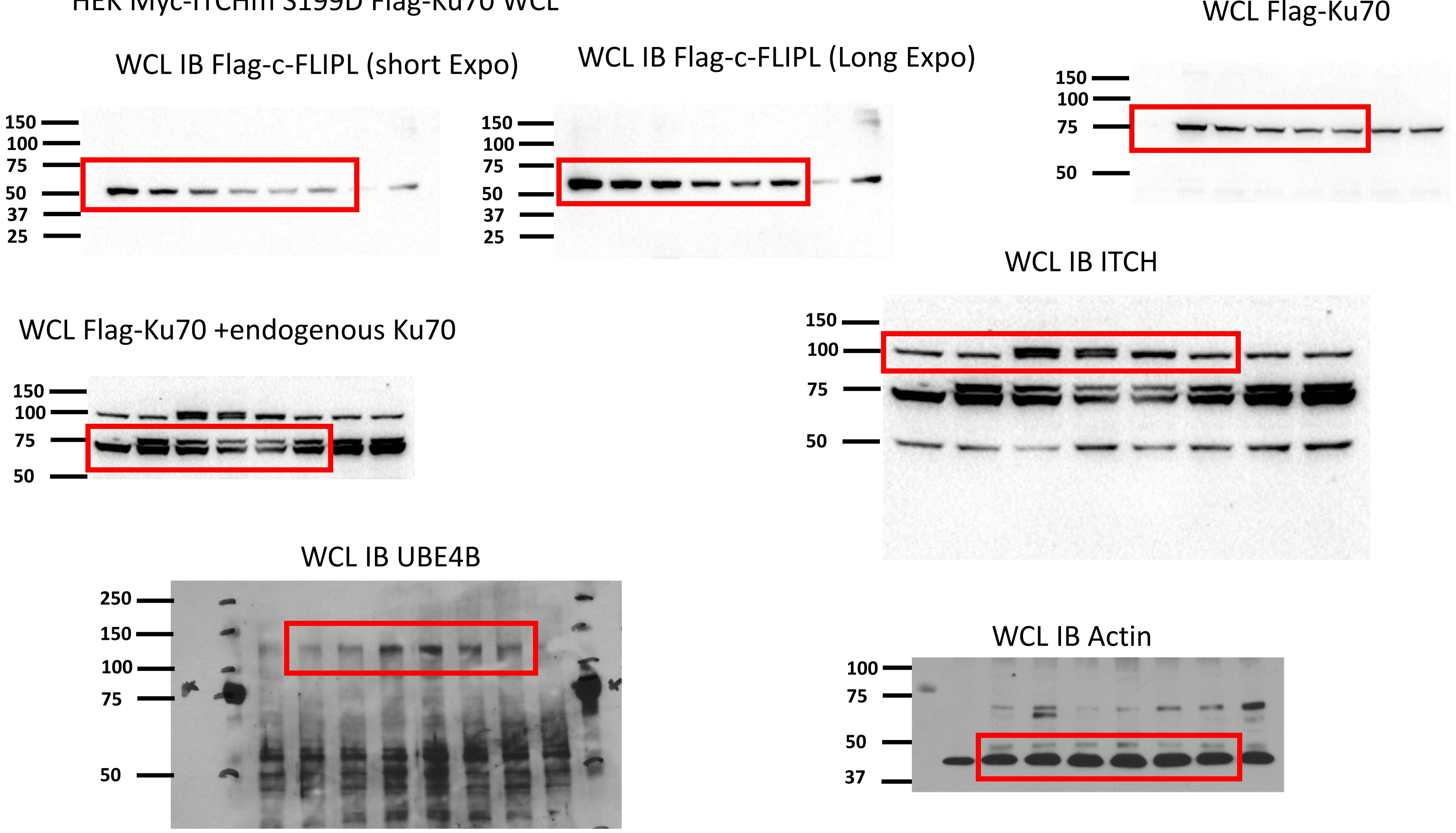

HEK Myc-ITCHm S199D Flag-Ku70 WCL
WCL IB Flag-c-FLIPL (short Expo)
150
100
75
50
37
25
WCL IB Flag-c-FLIPL (Long Expo)
150
100
75
50
37
25
WCL Flag-Ku70
150
100
75
50
WCL Flag-Ku70 +endogenous Ku70
150
100
75
50
WCL IB ITCH
150
100
75
50
WCL IB UBE4B
250
150
100
75
50
WCL IB Actin
100
75
50
37

SKNSH Saha 36h 1uM

Fig 5A

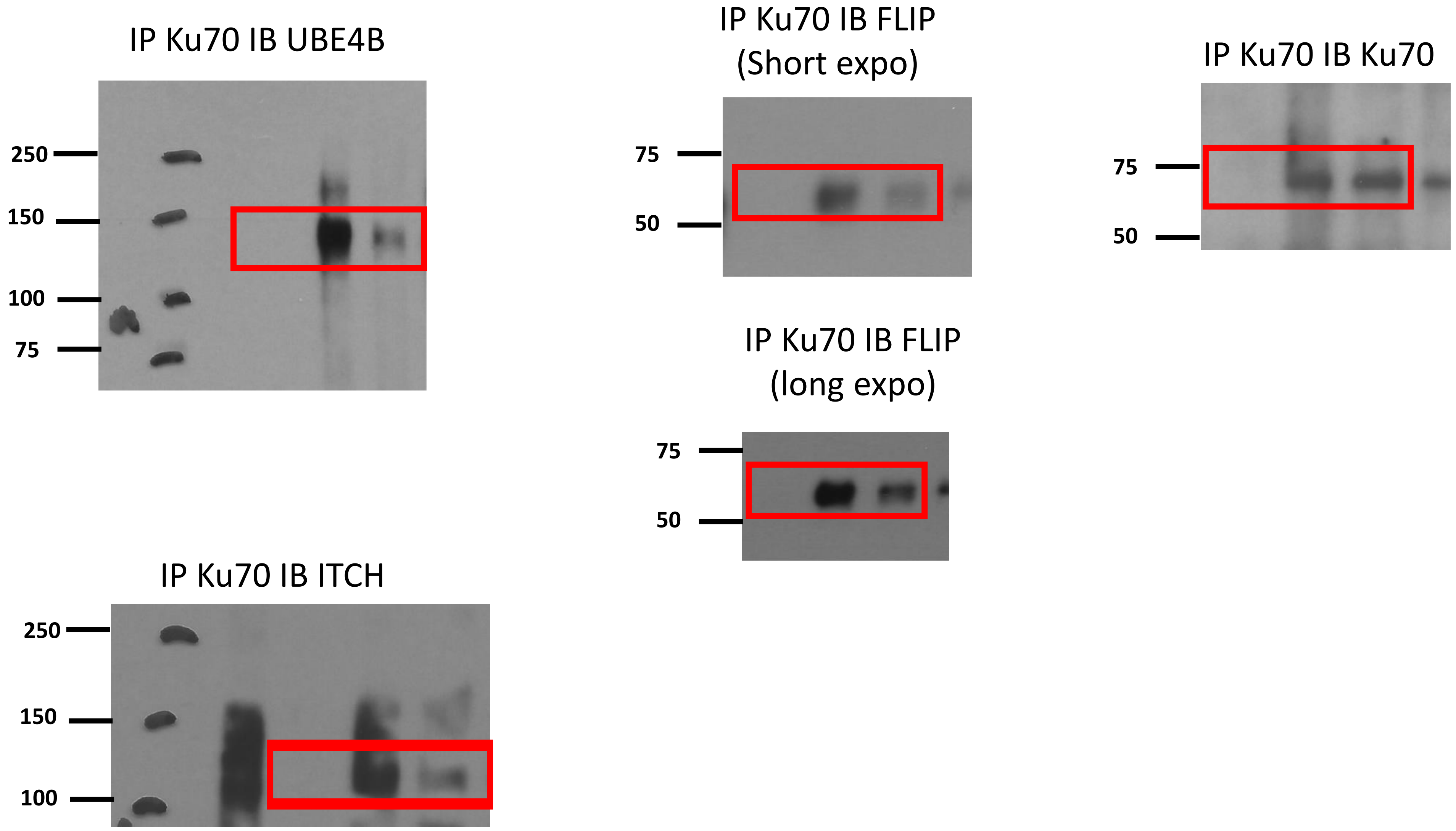

Fig 5A

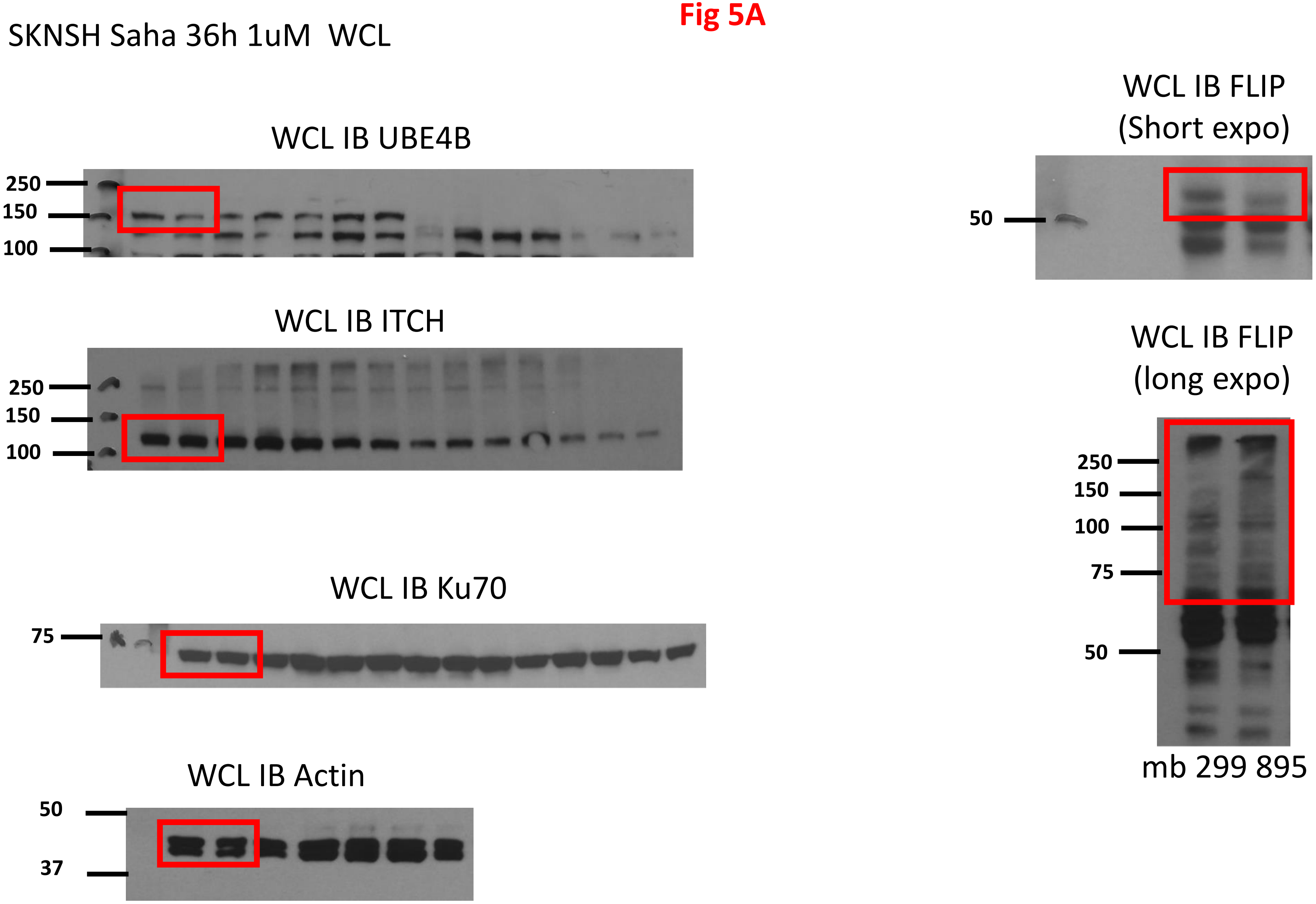
SKNSH Saha 36h 1uM WCL
WCL IB UBE4B
250
150
100
WCL IB ITCH
250
150
100
WCL IB Ku70
75
WCL IB Actin
50
37
WCL IB FLIP
(Short expo)
50
WCL IB FLIP
(long expo)
250
150
100
75
50
mb 299 895

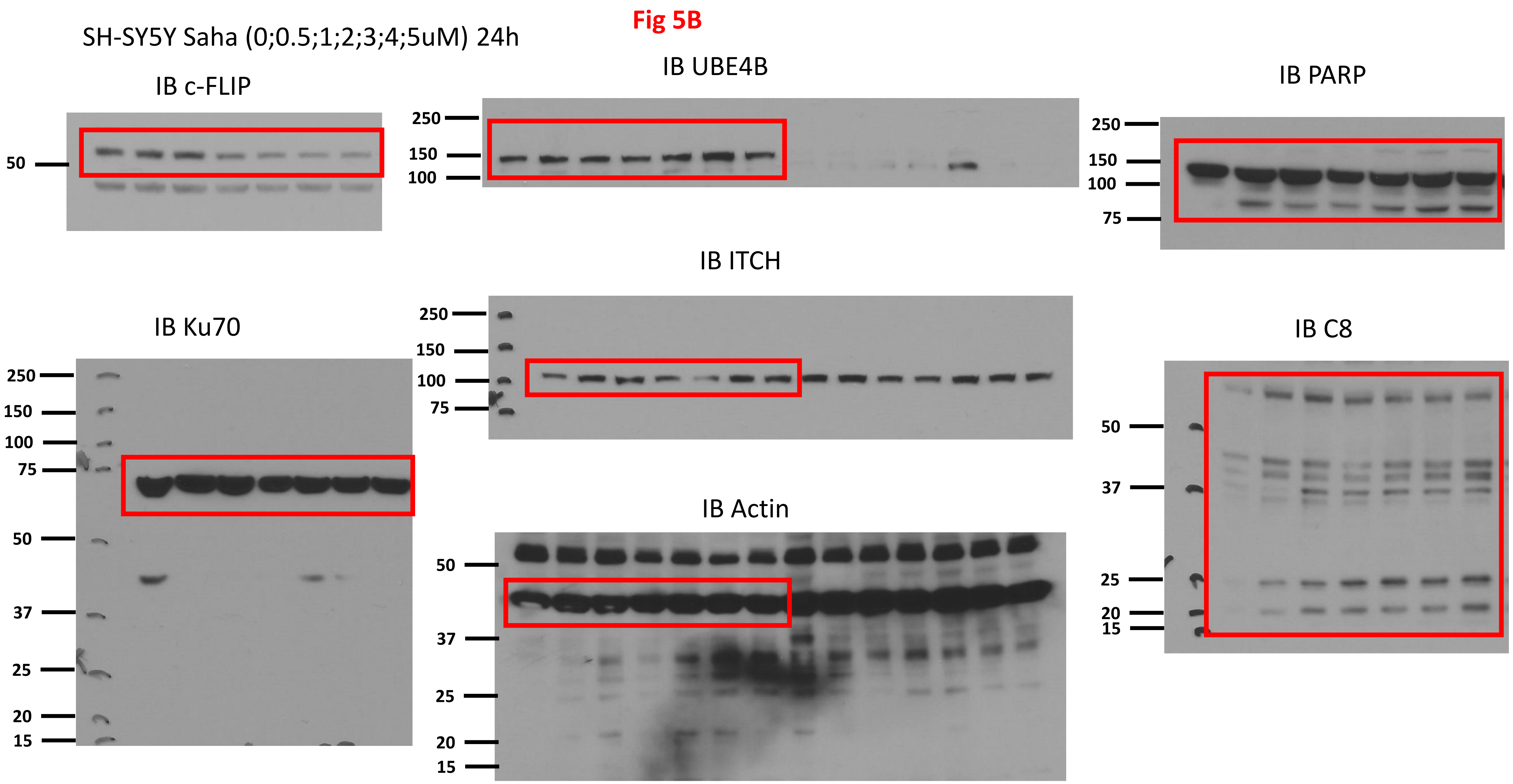
Fig 5B
SH-SY5Y Saha (0;0.5;1;2;3;4;5uM) 24h
IB c-FLIP
50
IB UBE4B
250
150
100
IB PARP
250
150
100
75
IB ITCH
250
150
100
75
IB Ku70
250
150
100
75
50
37
25
20
15
IB Actin
50
37
25
20
15
IB C8
50
37
25
20
15

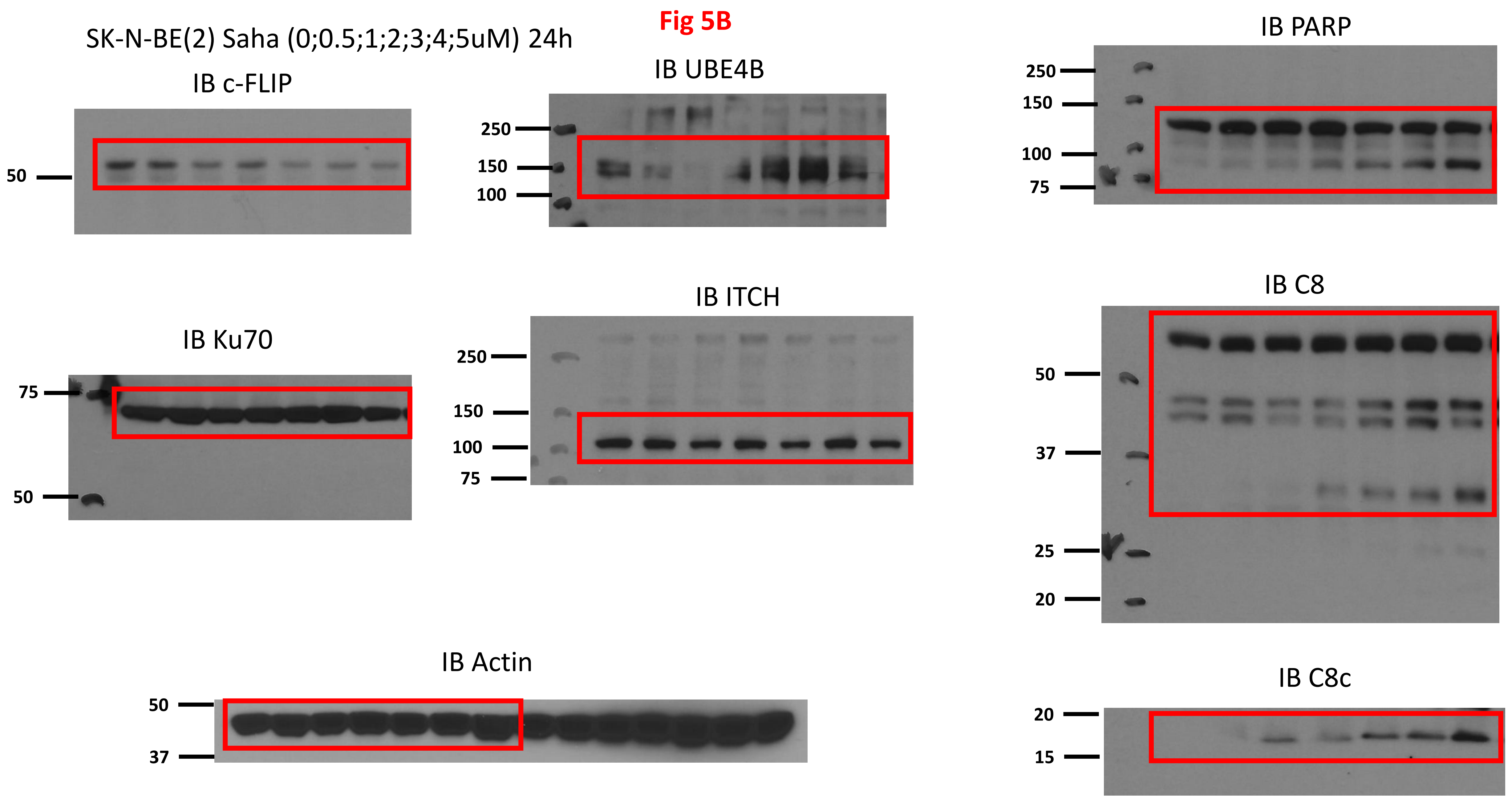
Fig 5B
SK-N-BE(2) Saha (0;0.5;1;2;3;4;5uM) 24h
IB c-FLIP
50
IB UBE4B
250
150
100
IB PARP
250
150
100
75
IB Ku70
75
50
IB ITCH
250
150
100
75
IB C8
50
37
25
20
IB Actin
50
37
IB C8c
20
15

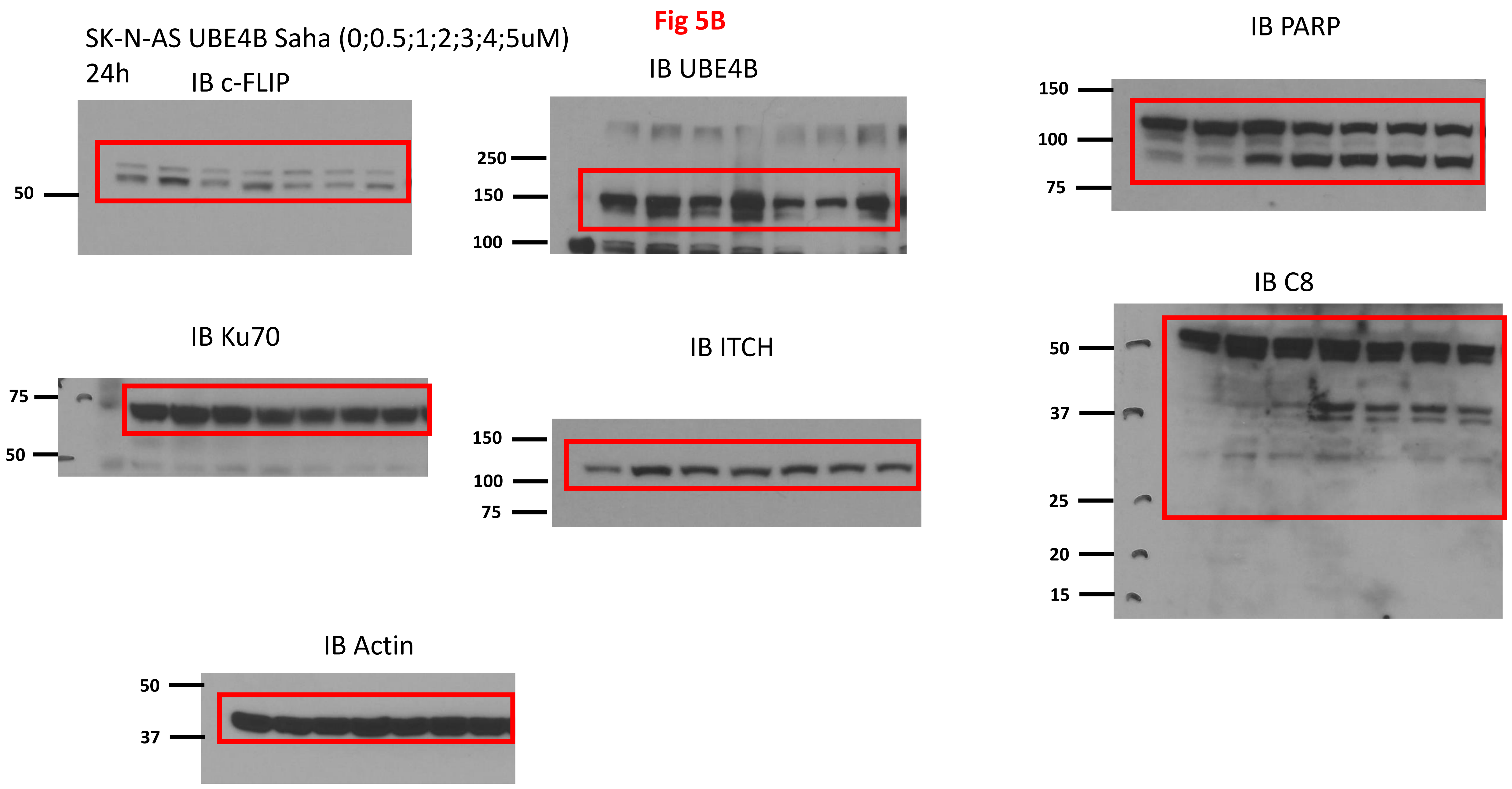
Fig 5B
SK-N-AS UBE4B Saha (0;0.5;1;2;3;4;5uM)
24h
IB c-FLIP
50
IB UBE4B
250
150
100
IB PARP
150
100
75
IB Ku70
75
50
IB ITCH
150
100
75
IB C8
50
37
25
20
15
IB Actin
50
37

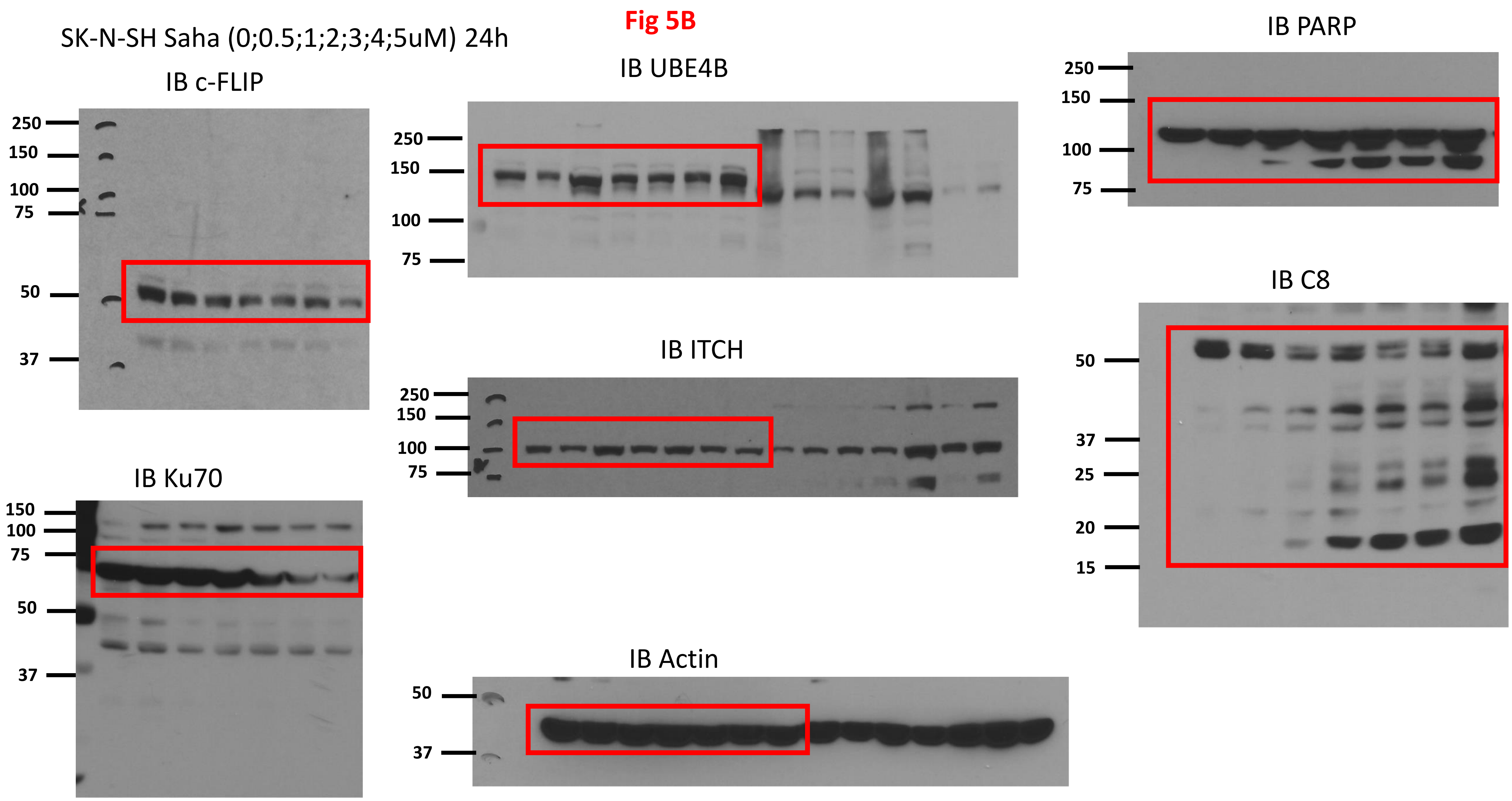
Fig 5B
SK-N-SH Saha (0;0.5;1;2;3;4;5uM) 24h
IB c-FLIP
250
150
100
75
50
37
IB UBE4B
250
150
100
75
IB PARP
250
150
100
75
IB ITCH
250
150
100
75
IB C8
50
37
25
20
15
IB Ku70
150
100
75
50
37
IB Actin
50
37

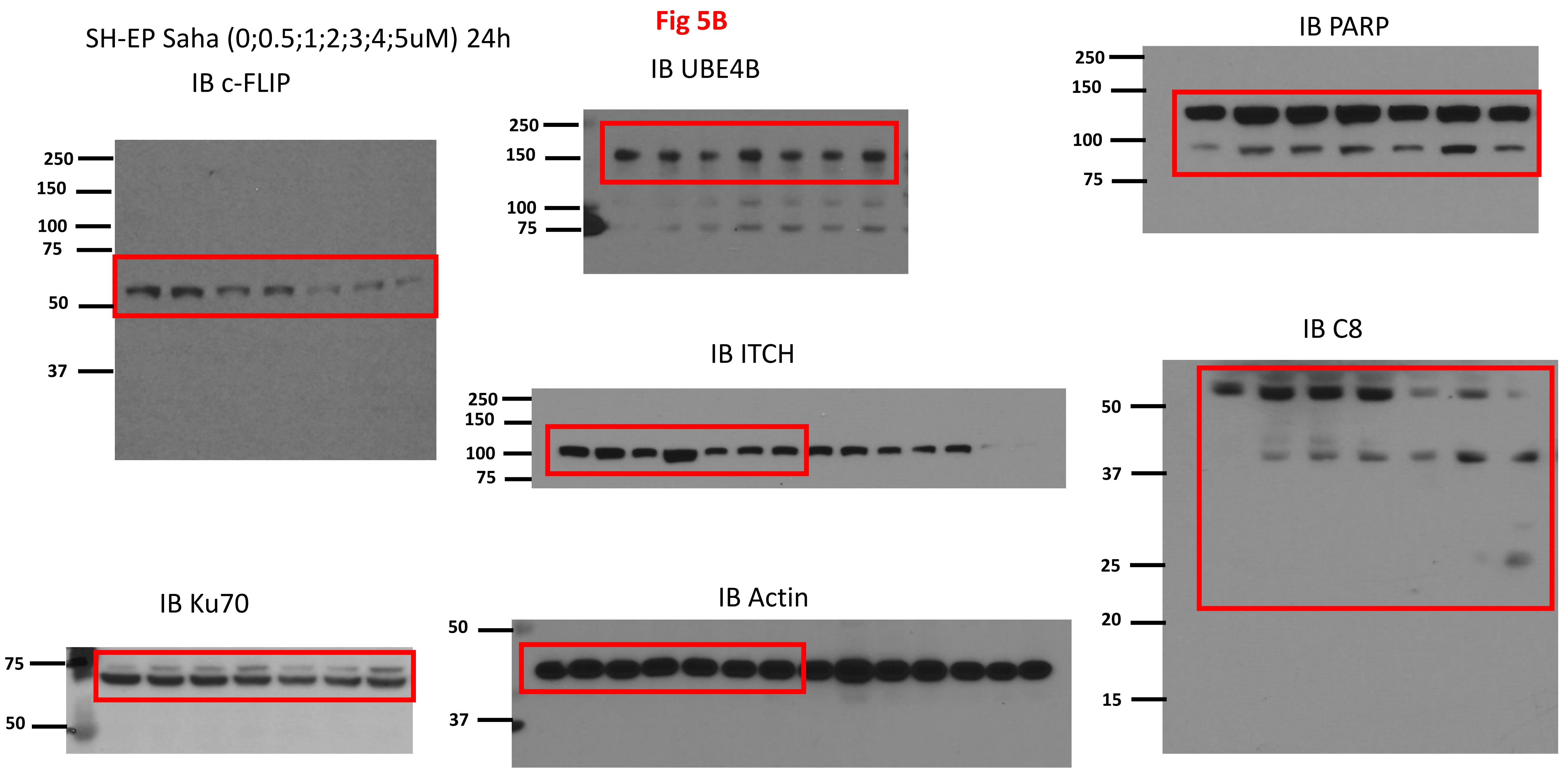
Fig 5B
SH-EP Saha (0;0.5;1;2;3;4;5uM) 24h
IB c-FLIP
250
150
100
75
50
37
IB UBE4B
250
150
100
75
IB PARP
250
150
100
75
IB ITCH
250
150
100
75
IB C8
50
37
25
20
15
IB Ku70
75
50
IB Actin
50
37

Fig 5C

SK-N-AS Saha 0-6h 4uM

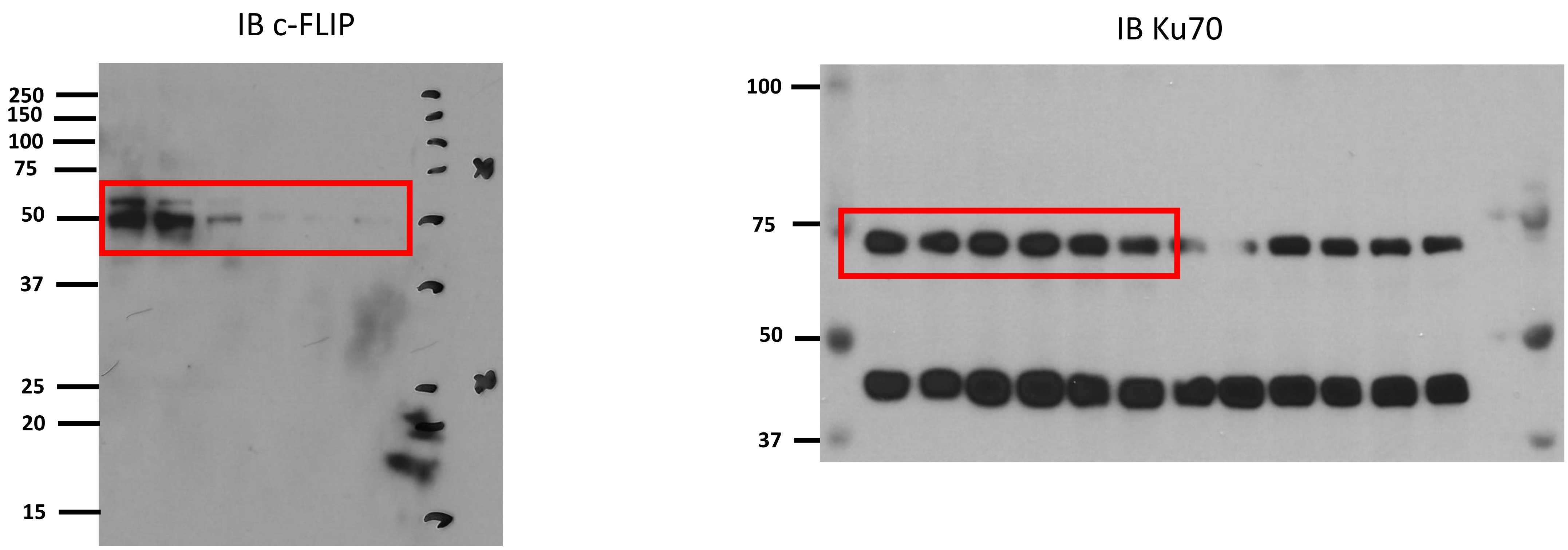

SK-N-AS Saha 0-6h 4uM

Fig 5C

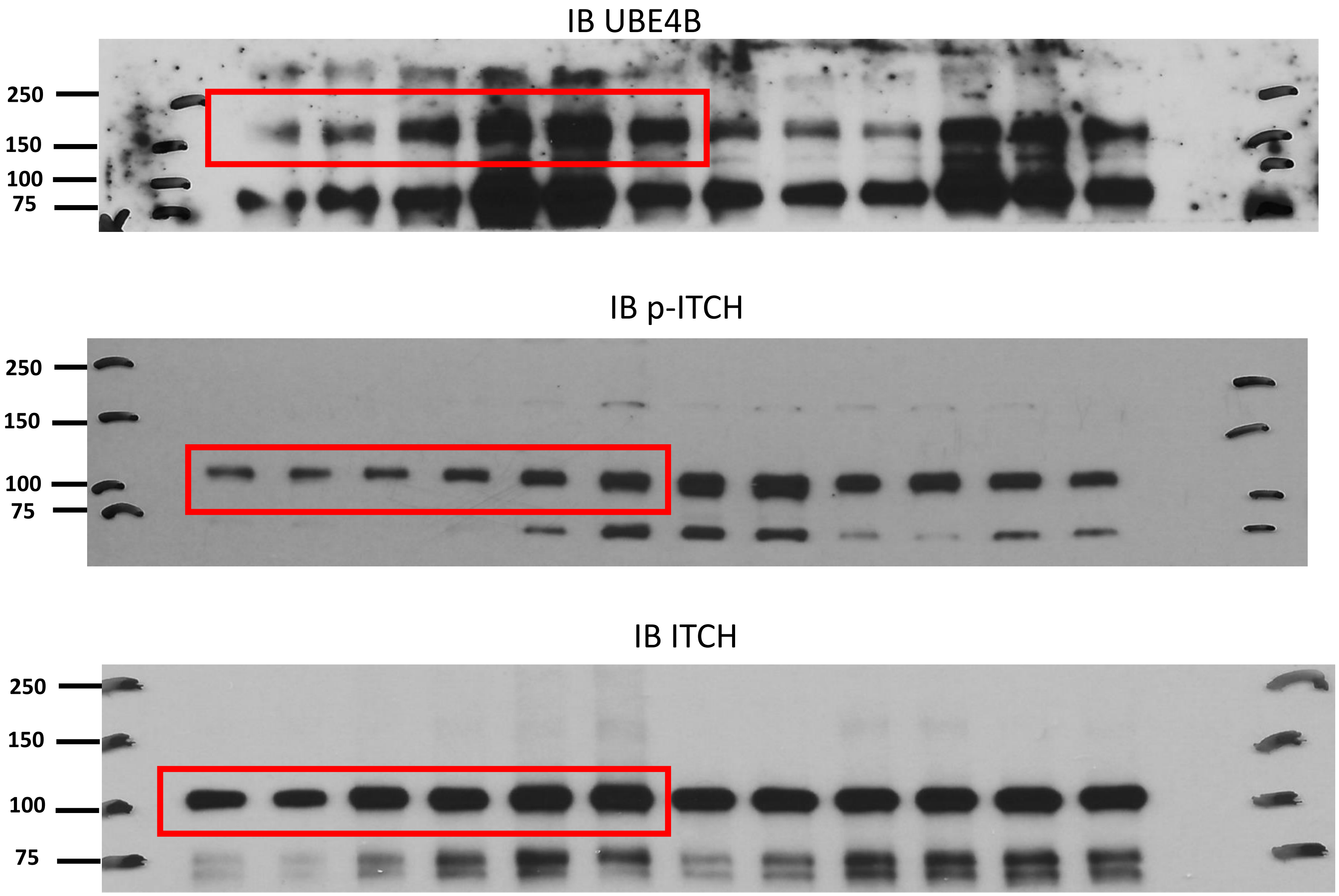

Fig 5C

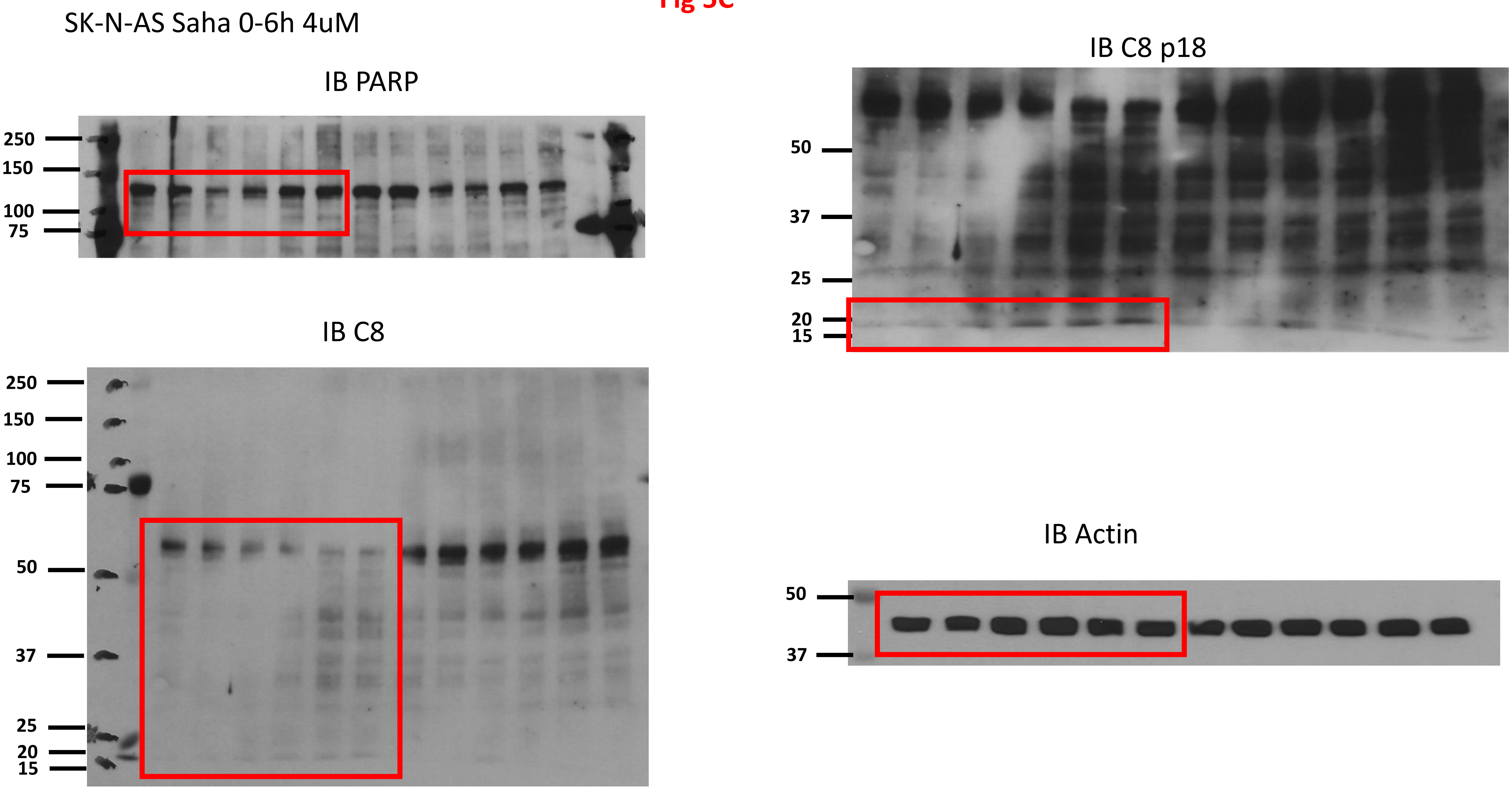
SK-N-AS Saha 0-6h 4uM
IB PARP
250
150
100
75
IB C8
250
150
100
75
50
37
25
20
15
IB C8 p18
50
37
25
20
15
IB Actin
50
37

SK-N-AS Saha 0-6h 4uM -/+ MG

Fig 5D

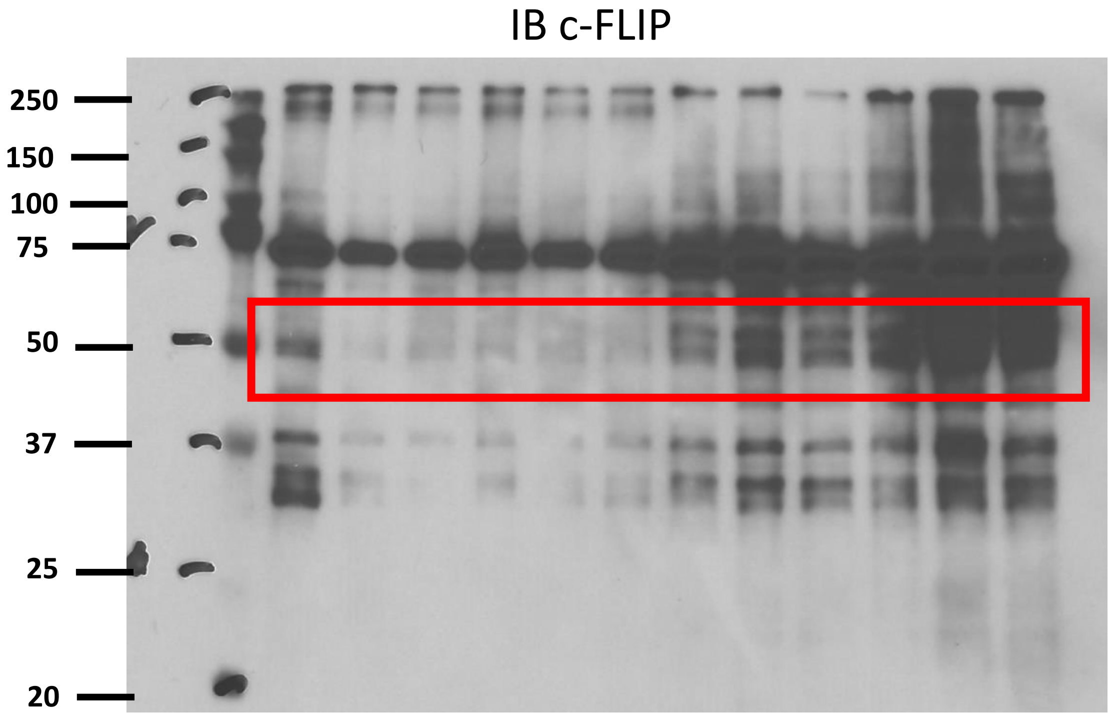

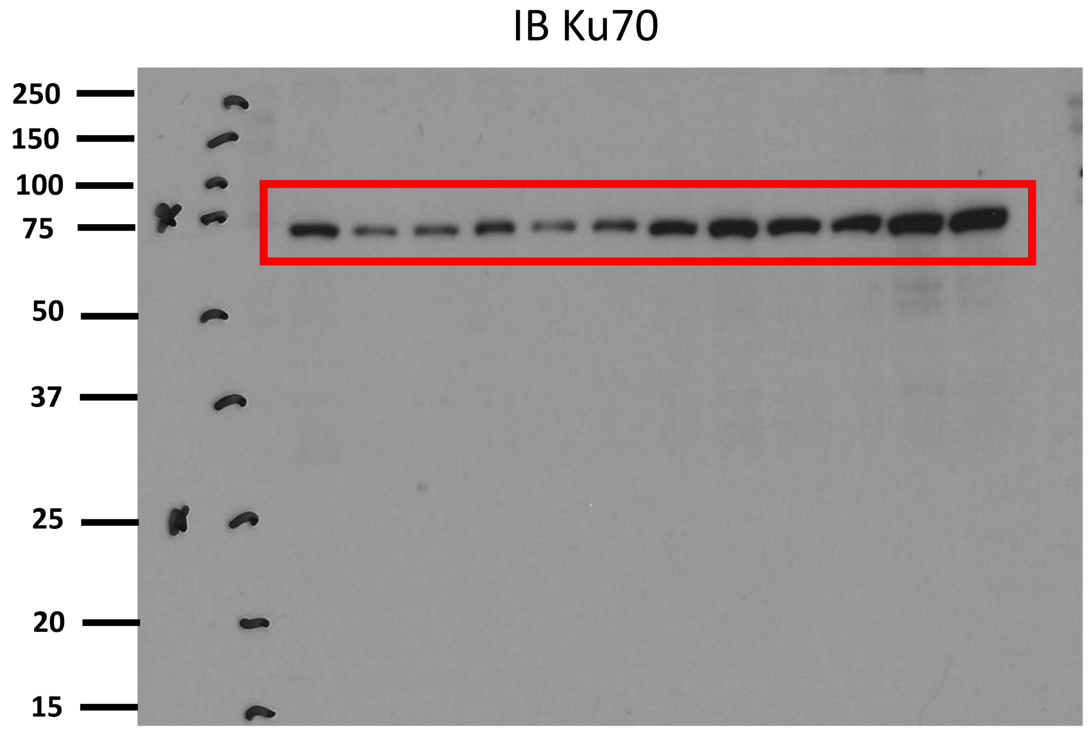

Fig 5D

SK-N-AS Saha 0-6h 4uM

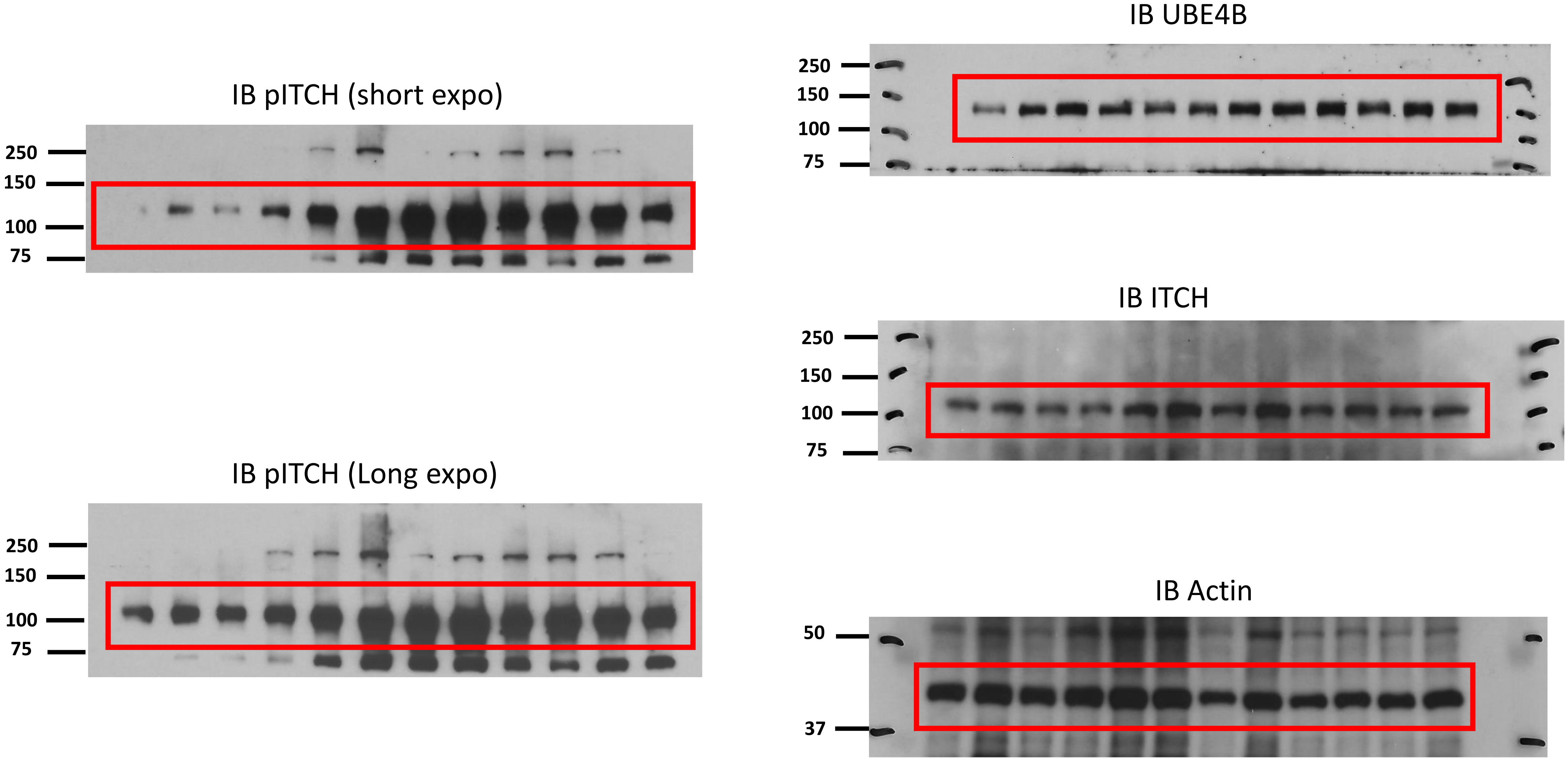

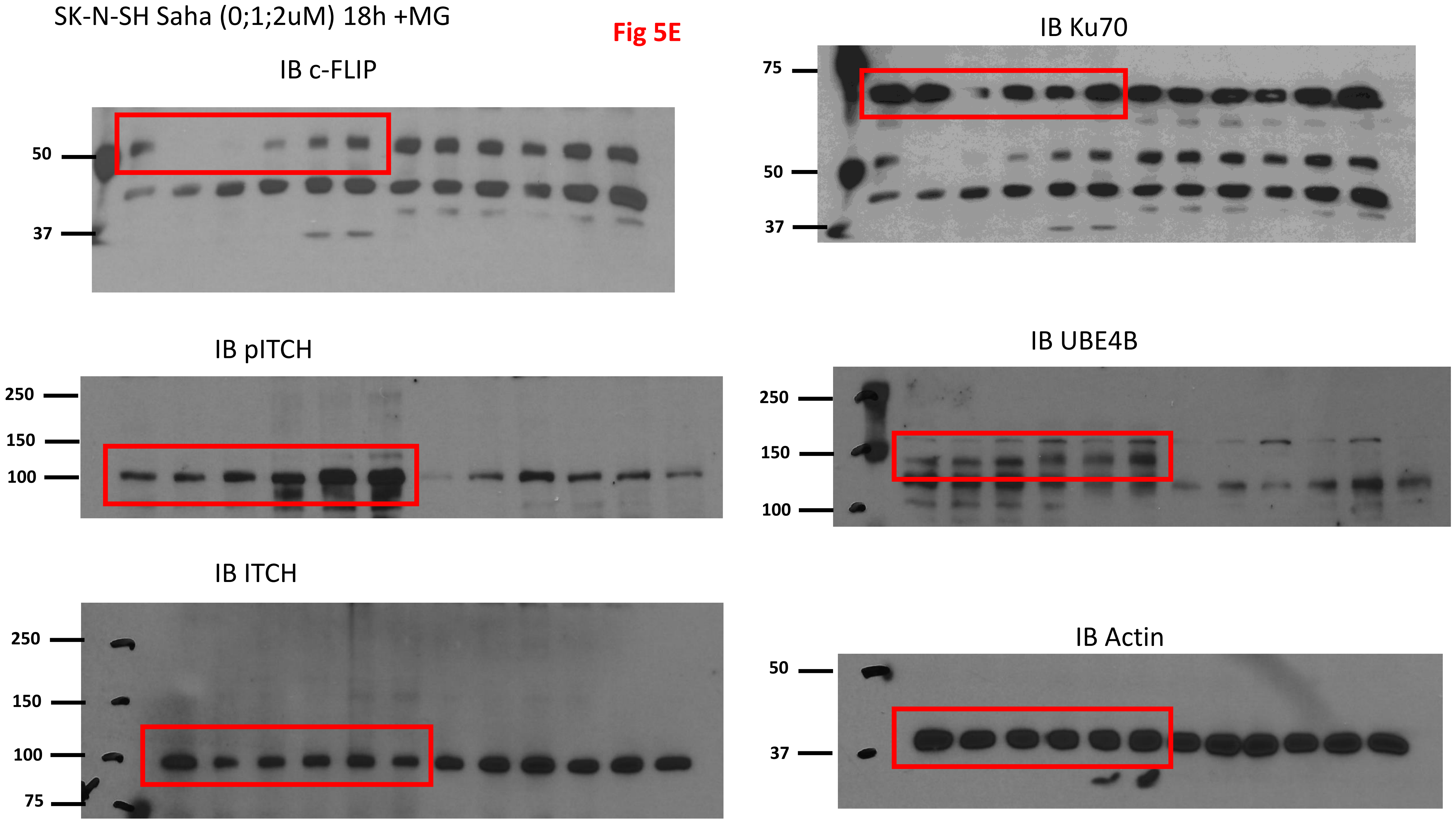
SK-N-SH Saha (0;1;2uM) 18h +MG
Fig 5E
IB c-FLIP
50
37
IB Ku70
75
50
37
IB pITCH
250
150
100
IB UBE4B
250
150
100
IB ITCH
250
150
100
75
IB Actin
50
37

SK-N-SH Saha (0;1;2uM) 18h +MG

Fig 5E

IB USP8

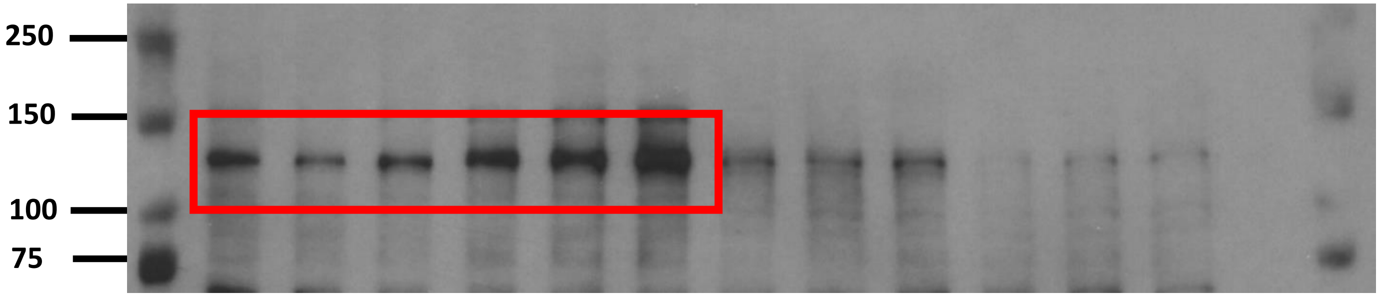

Fig 5F

SKNAS SC vs UBE4B KD Saha 0-6h 4uM

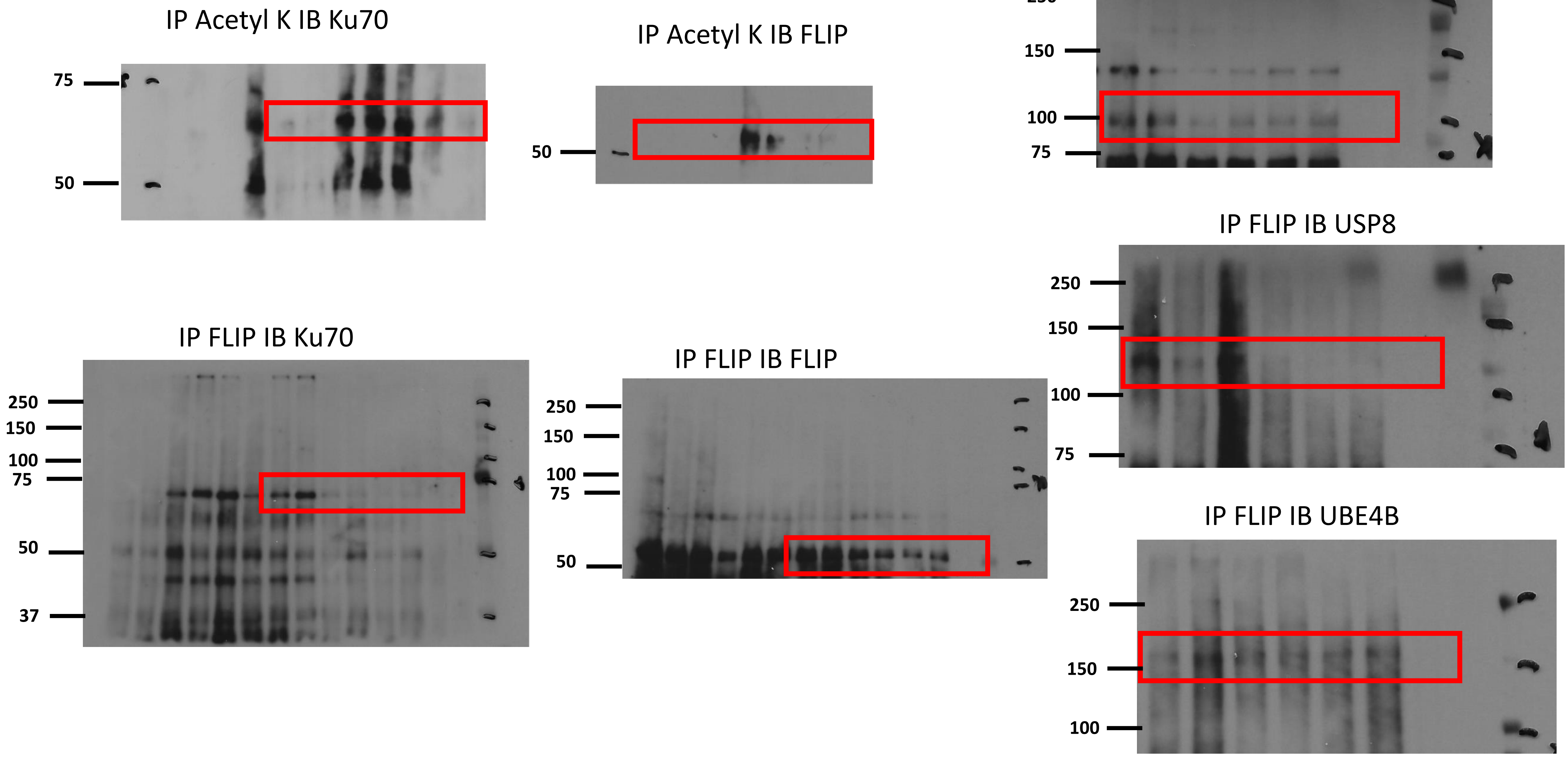

SKNAS SC vs UBE4B KD Saha 0-6h 4uM

Fig 5F

IP Ku70 IB FLIP

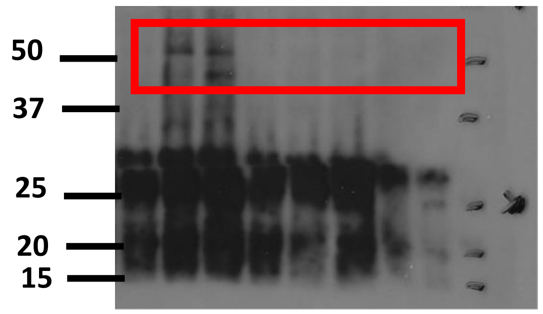

IP Ku70 IB ITCH

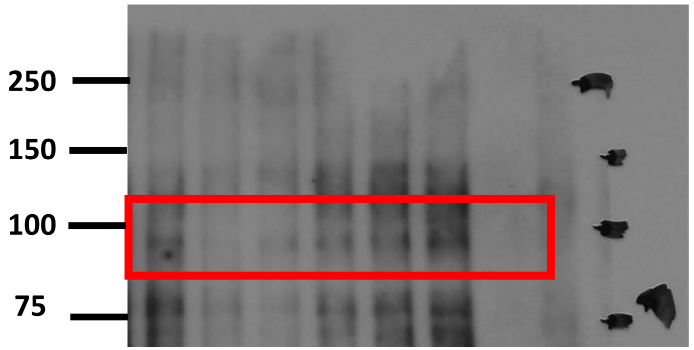

IP Ku70 IB Bax

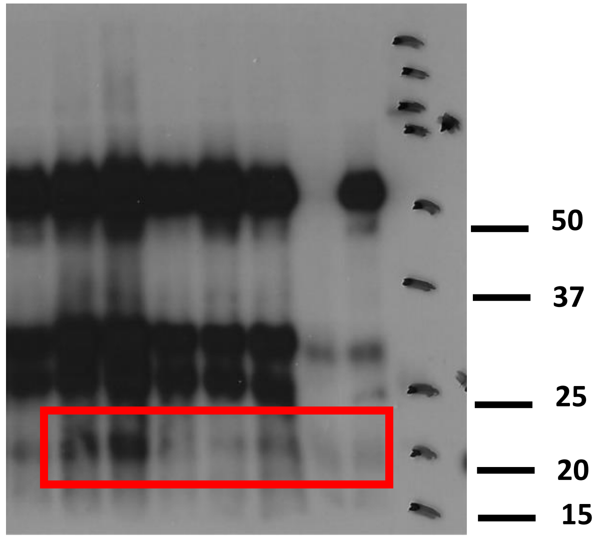

IP Ku70 IB Ku70

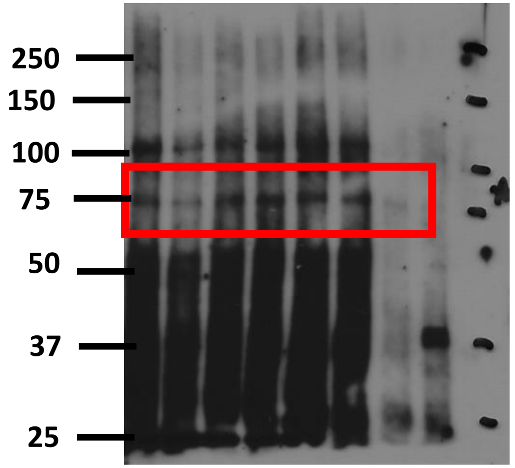

IP Ku70 IB UBE4B

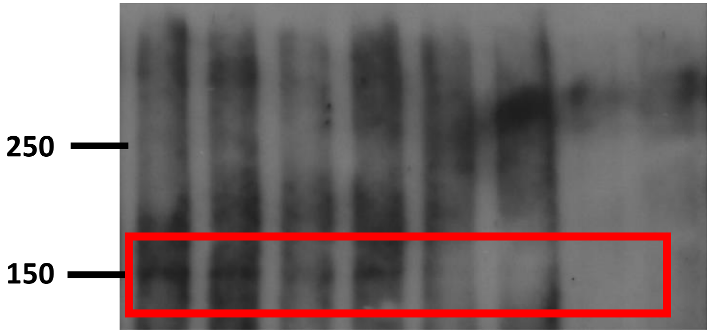

Fig 5F

SKNAS SC vs UBE4B KD Saha 0-6h 4uM

IP Bax IB Ku70

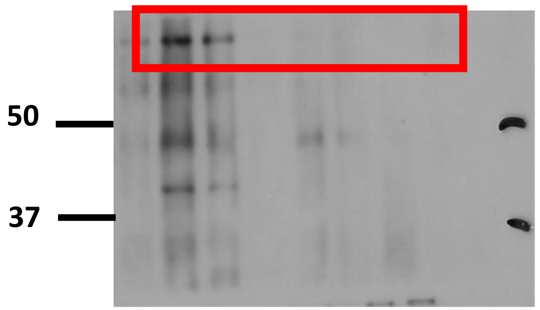

IP Bax IB FLIP

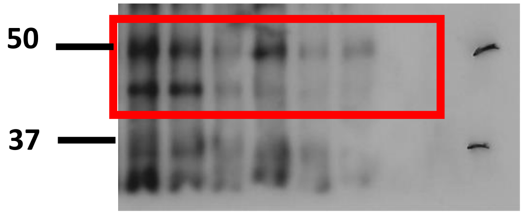

IP Bax IB Bax

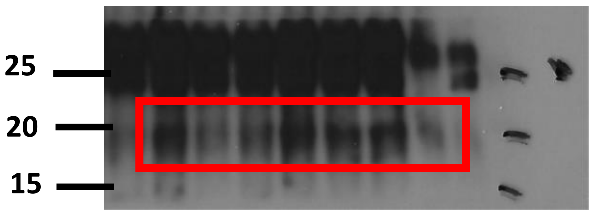

Fig 5F

SKNAS SC vs UBE4B KD Saha 0-6h 4uM

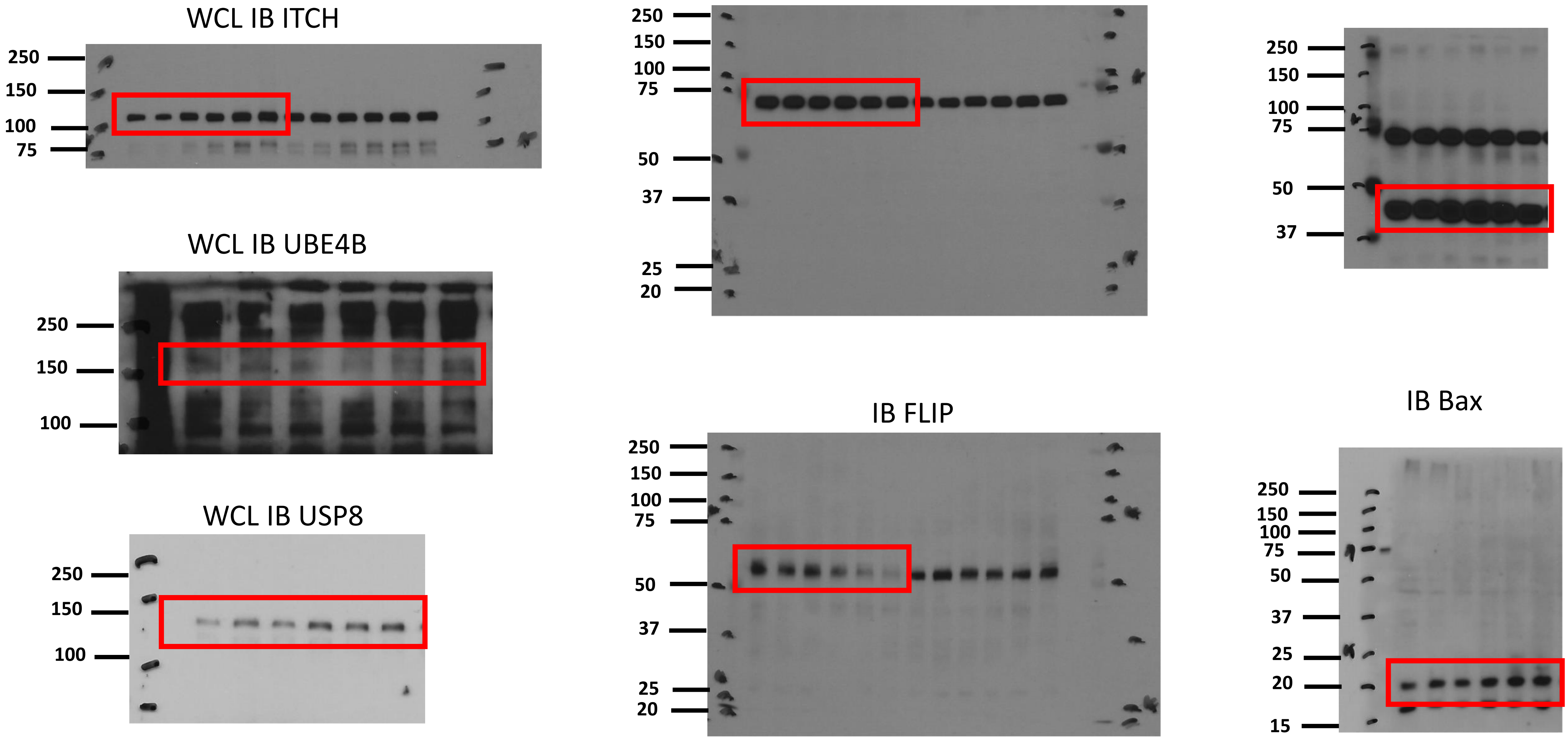

Fig 5G

SKNAS SC vs UBE4B KD Saha 0-6h 4uM

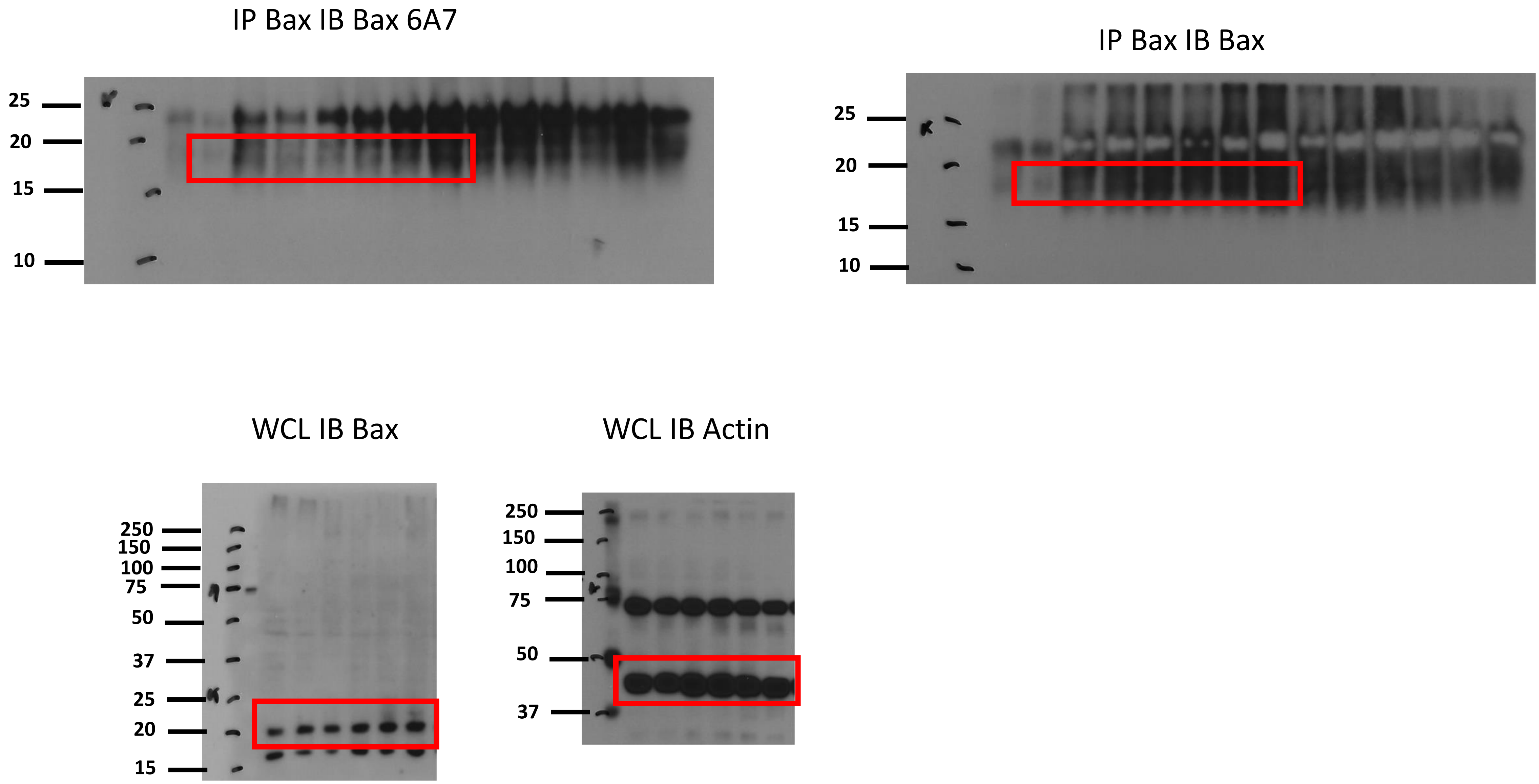

SKNAS SC vs UBE4B KD Saha 0-6h 4uM

Fig 5H

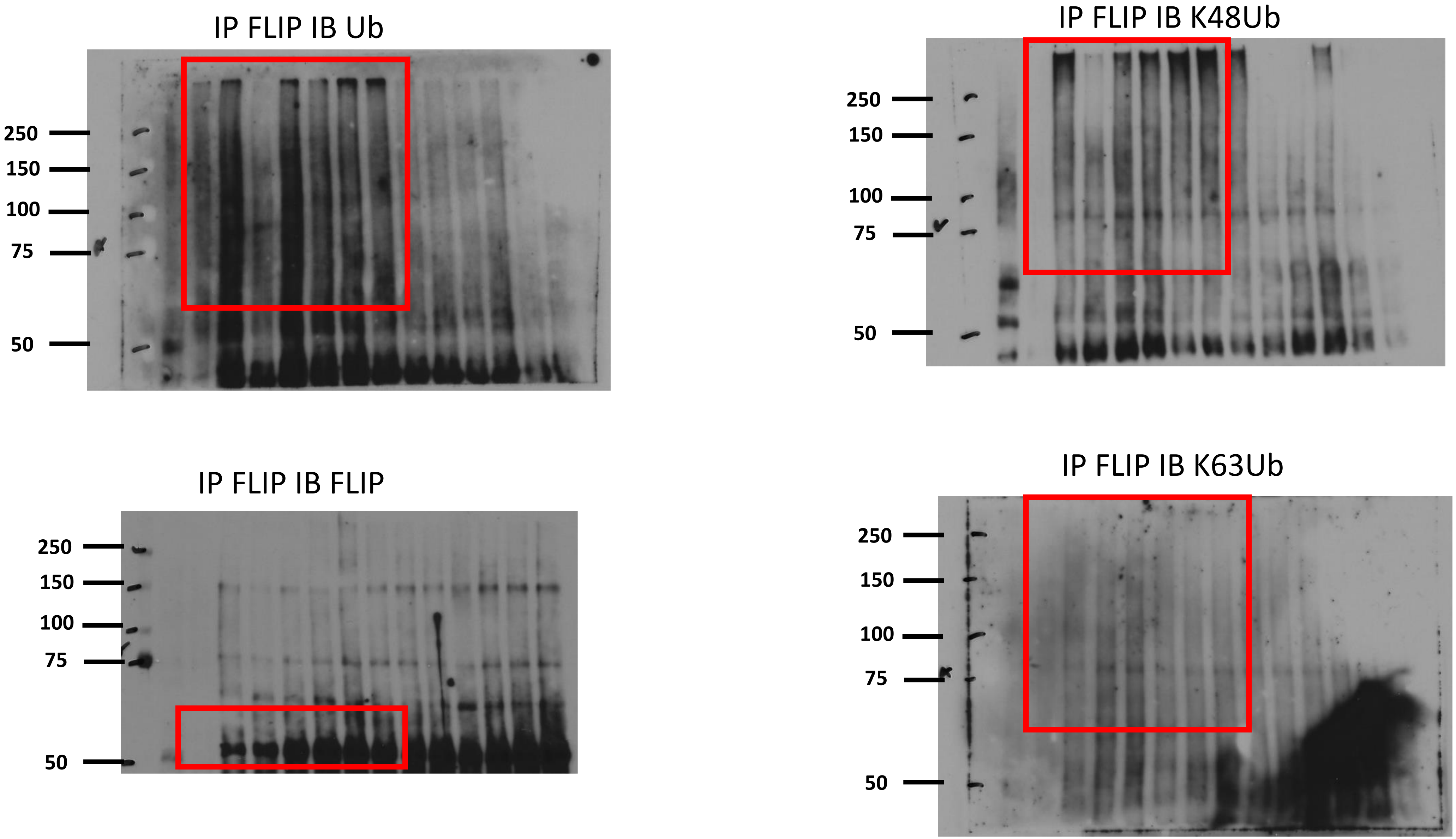

Fig 5H

SKNAS SC vs UBE4B KD Saha 0-6h 4uM

IP Ku70 IB Ub

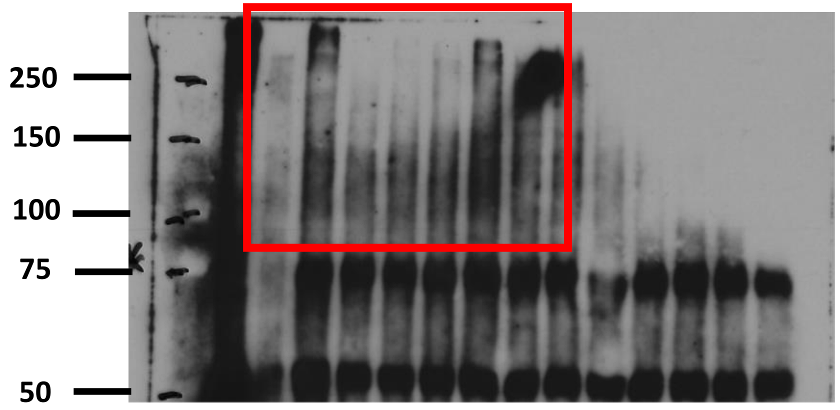

IP Ku70 IB K48Ub

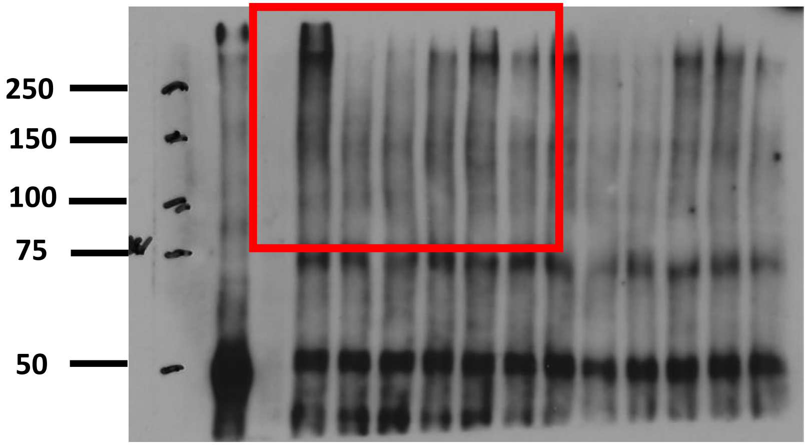

IP Ku70 IB Ku70

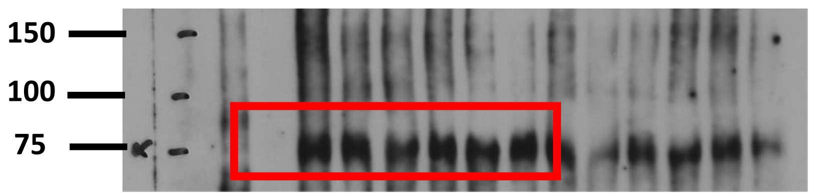

IP Ku70 IB K63Ub

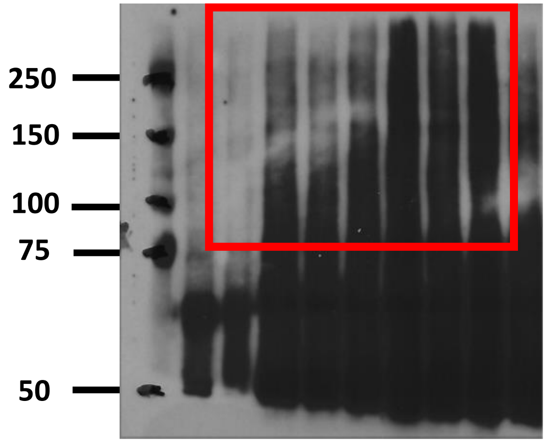

Fig 5H

SKNAS SC vs UBE4B KD Saha 0-6h 4uM WCL

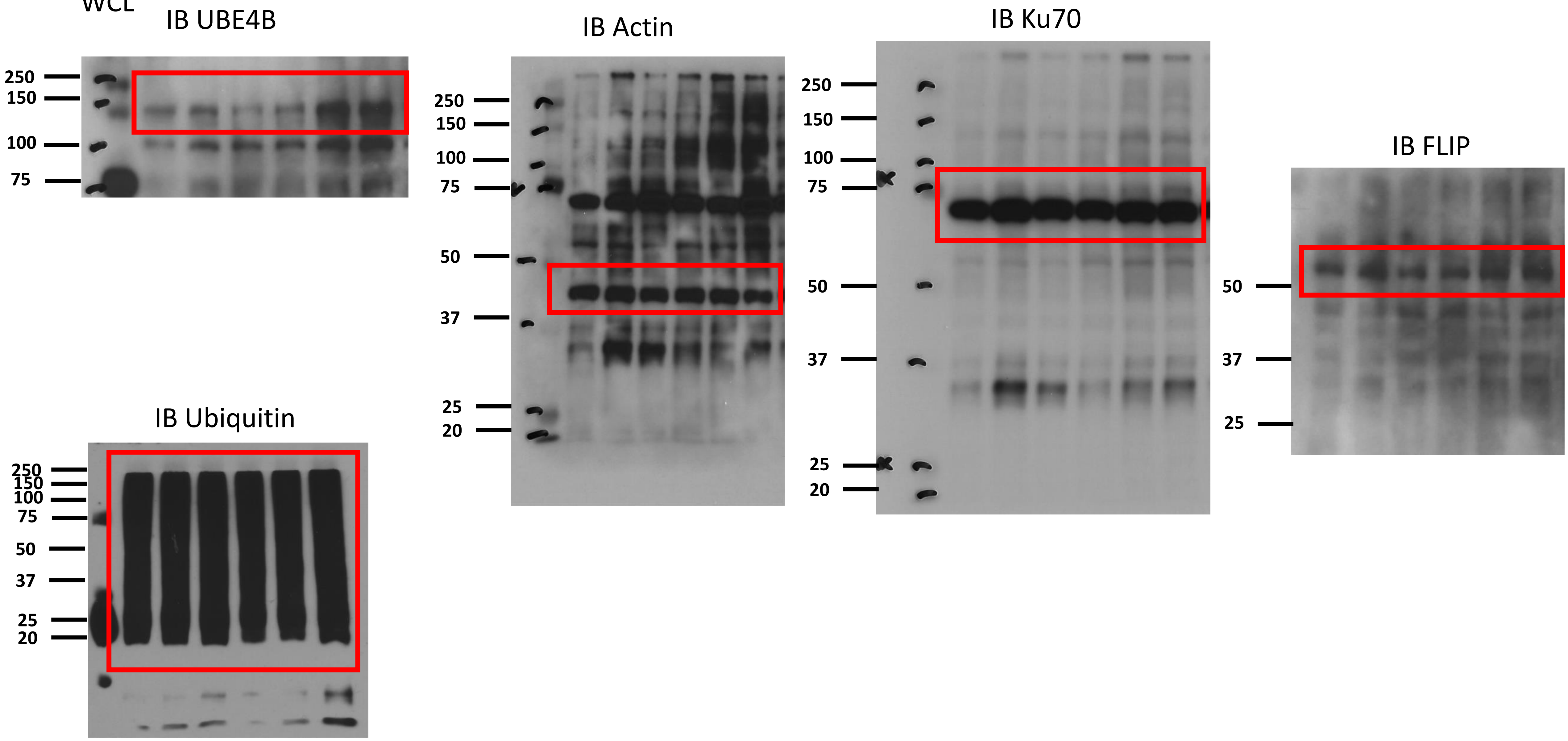

Fig 5I

SKNSH SC vs UBE4B KD Saha (0;1;2uM) 18h

IP FLIP IB Ub

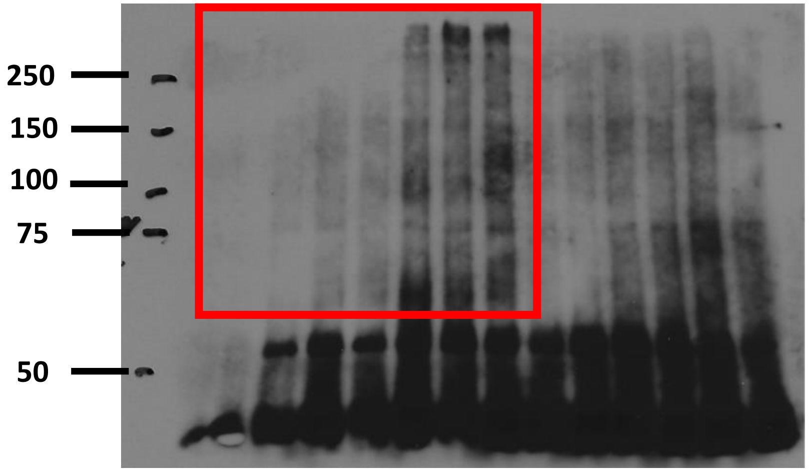

IP FLIP IB K48Ub

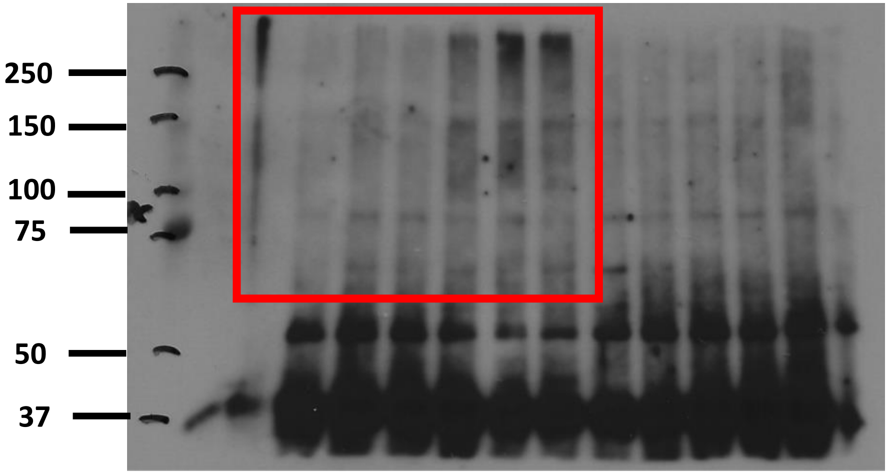

IP FLIP IB FLIP

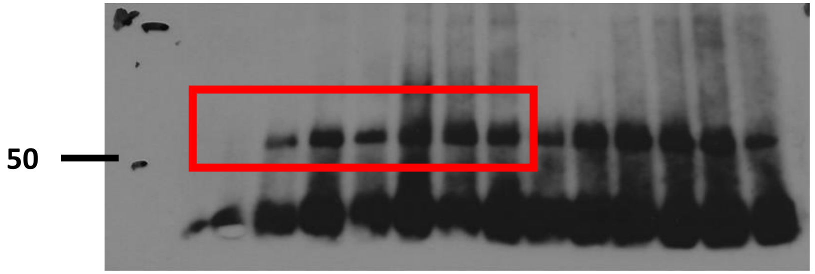

IP FLIP IB K63Ub

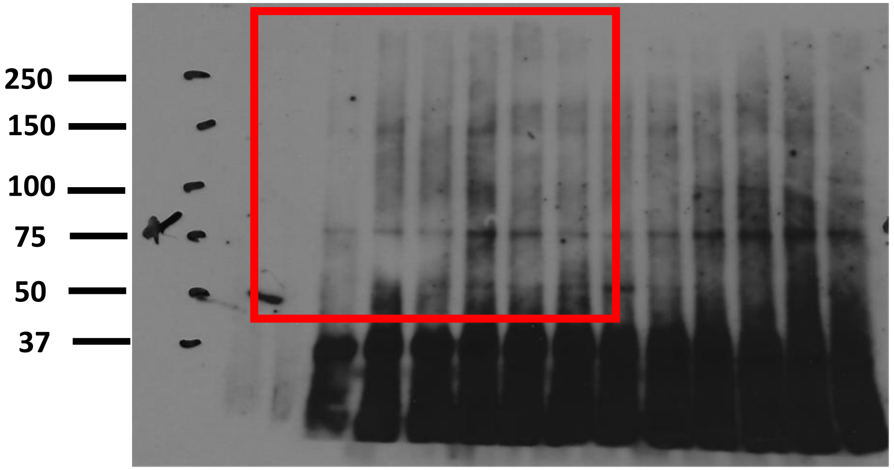

Fig 5I

SKNSH SC vs UBE4B KD Saha (0;1;2uM) 18h

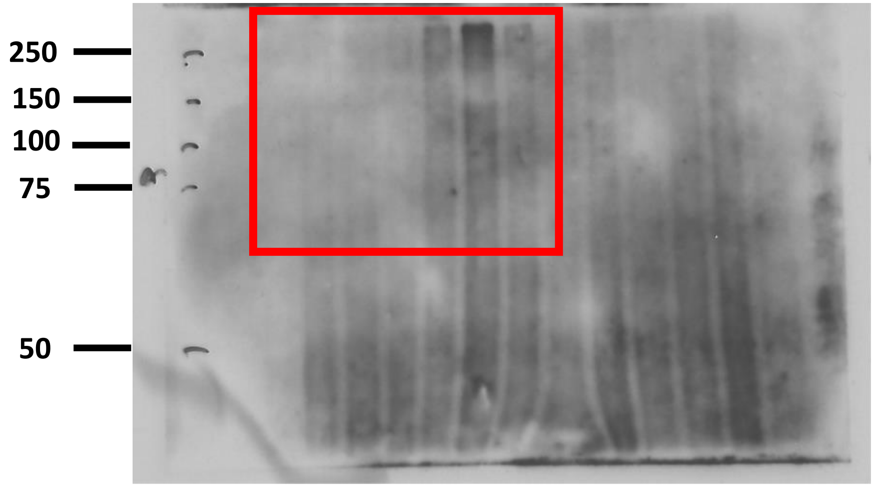

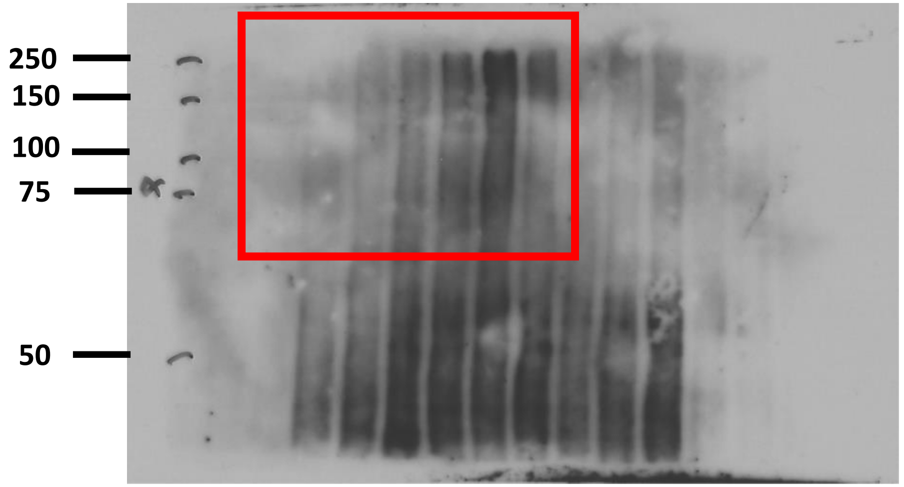

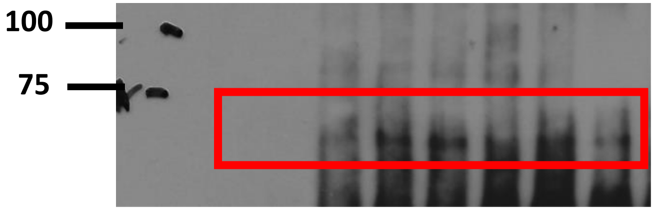

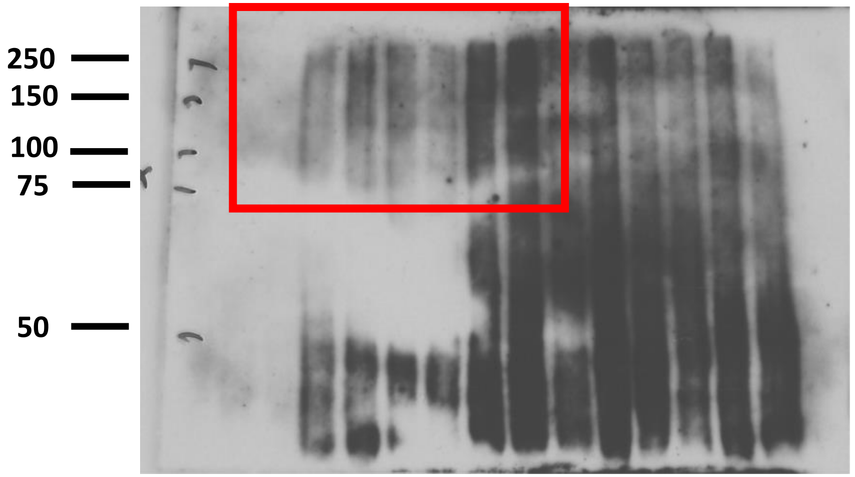

SKNSH SC vs UBE4B KD Saha (0;1;2uM) 18h WCL **Fig 5I**

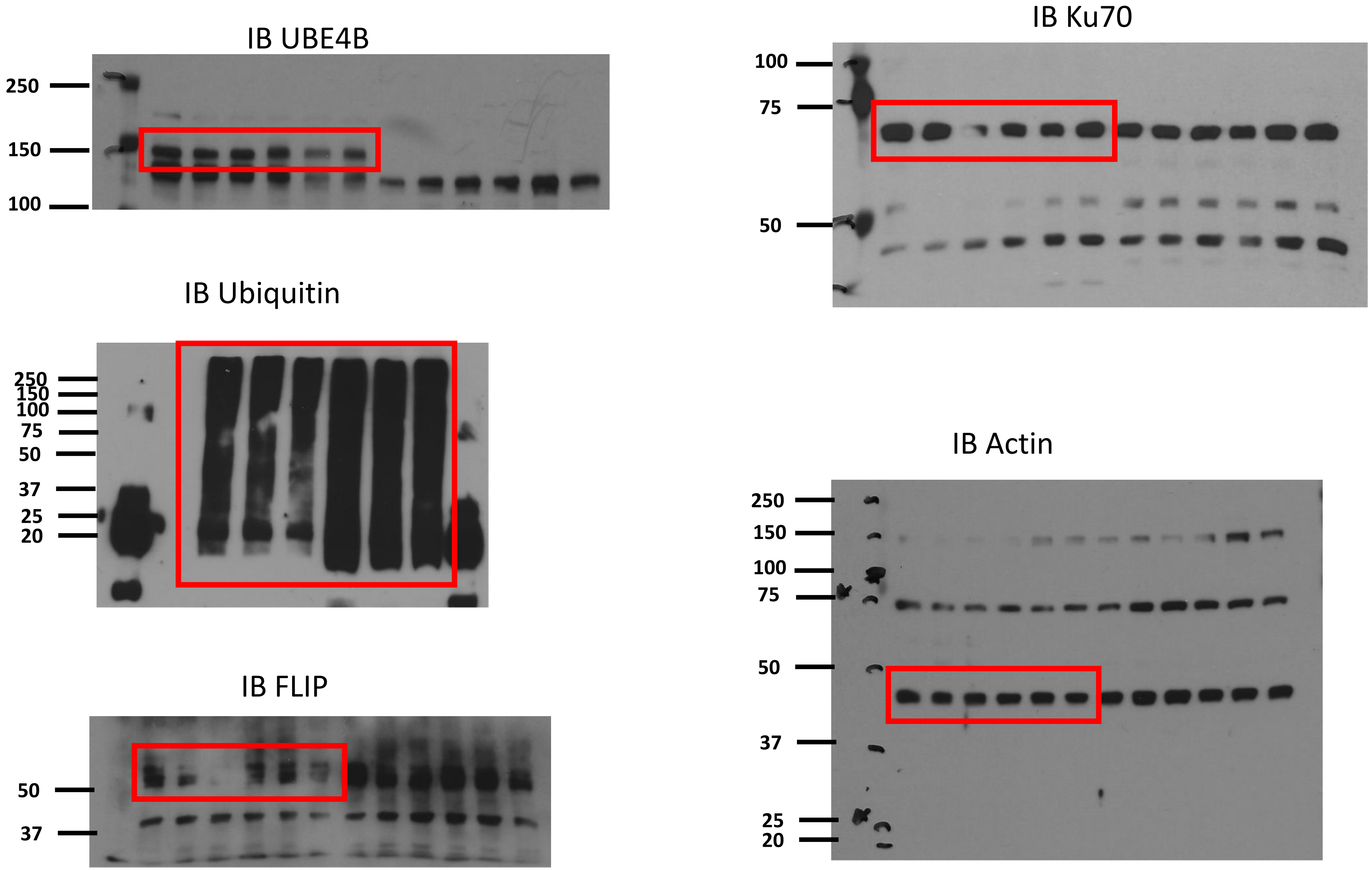

Fig 6A

SKNSH SC vs UBE4B KD Saha 0-5uM 24h

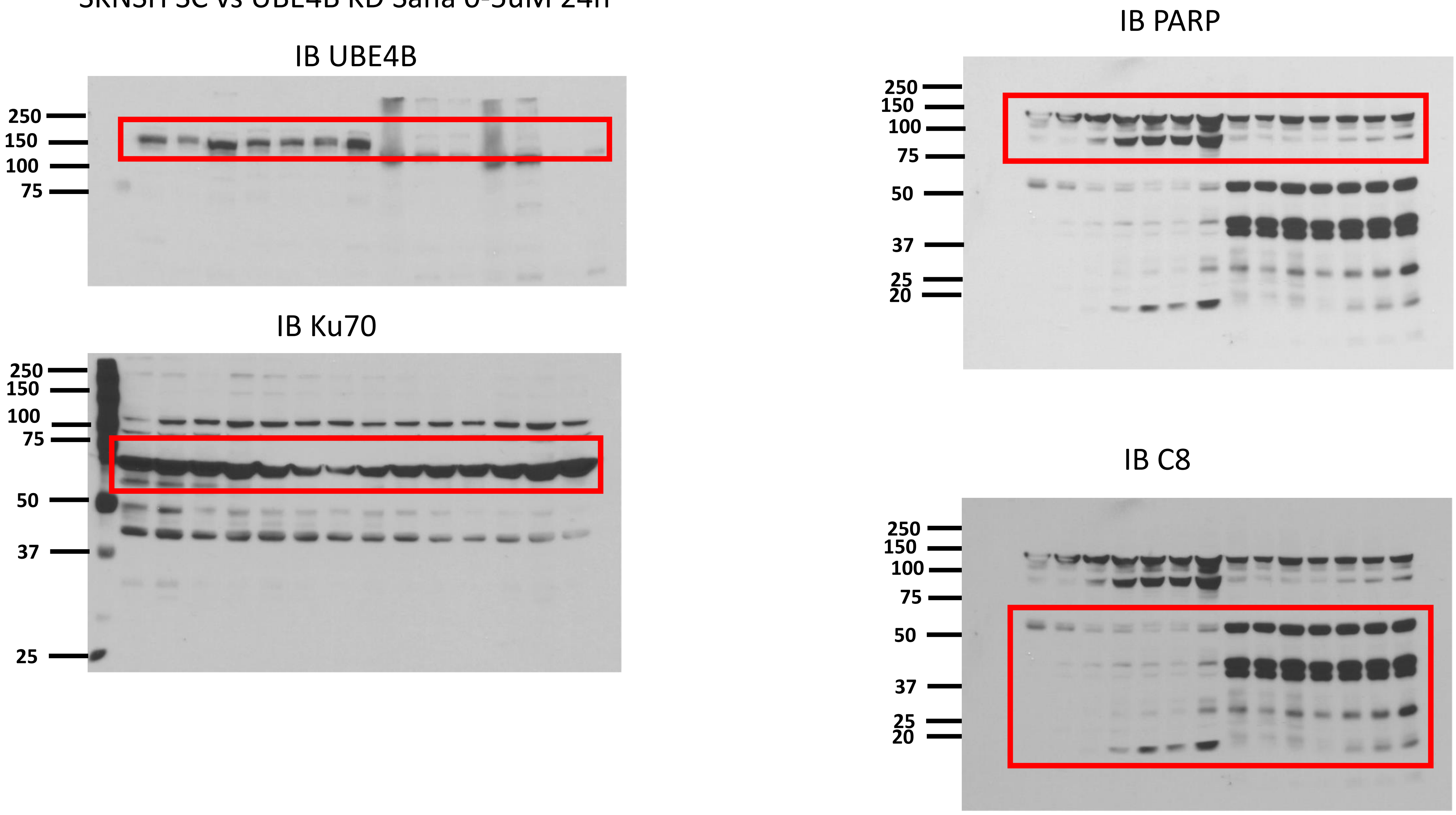

Fig 6A

SKNSH SC vs UBE4B KD Saha 0-5uM 24h

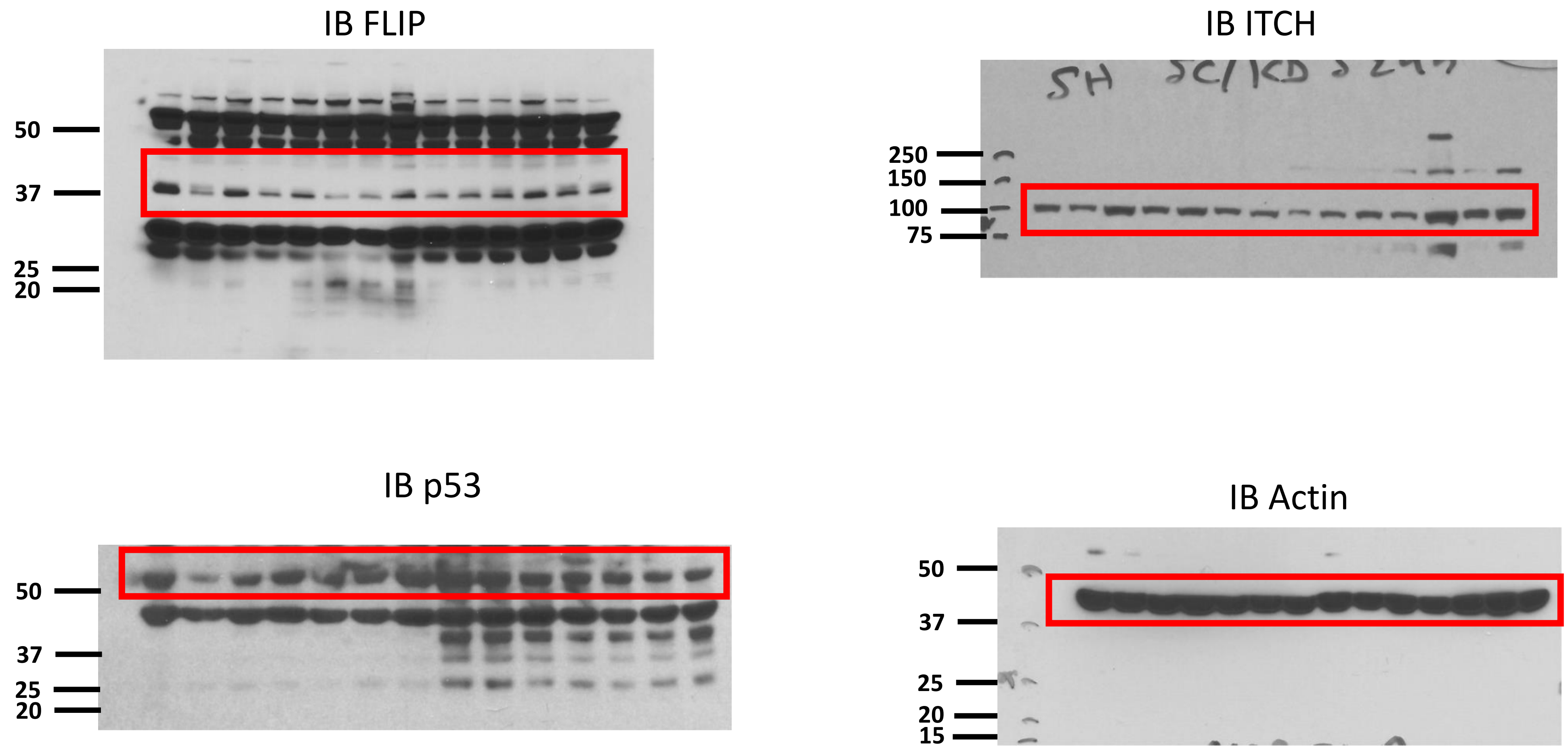

SKNAS SC vs UBE4B KD Saha 0-5uM 24h

Fig 6B

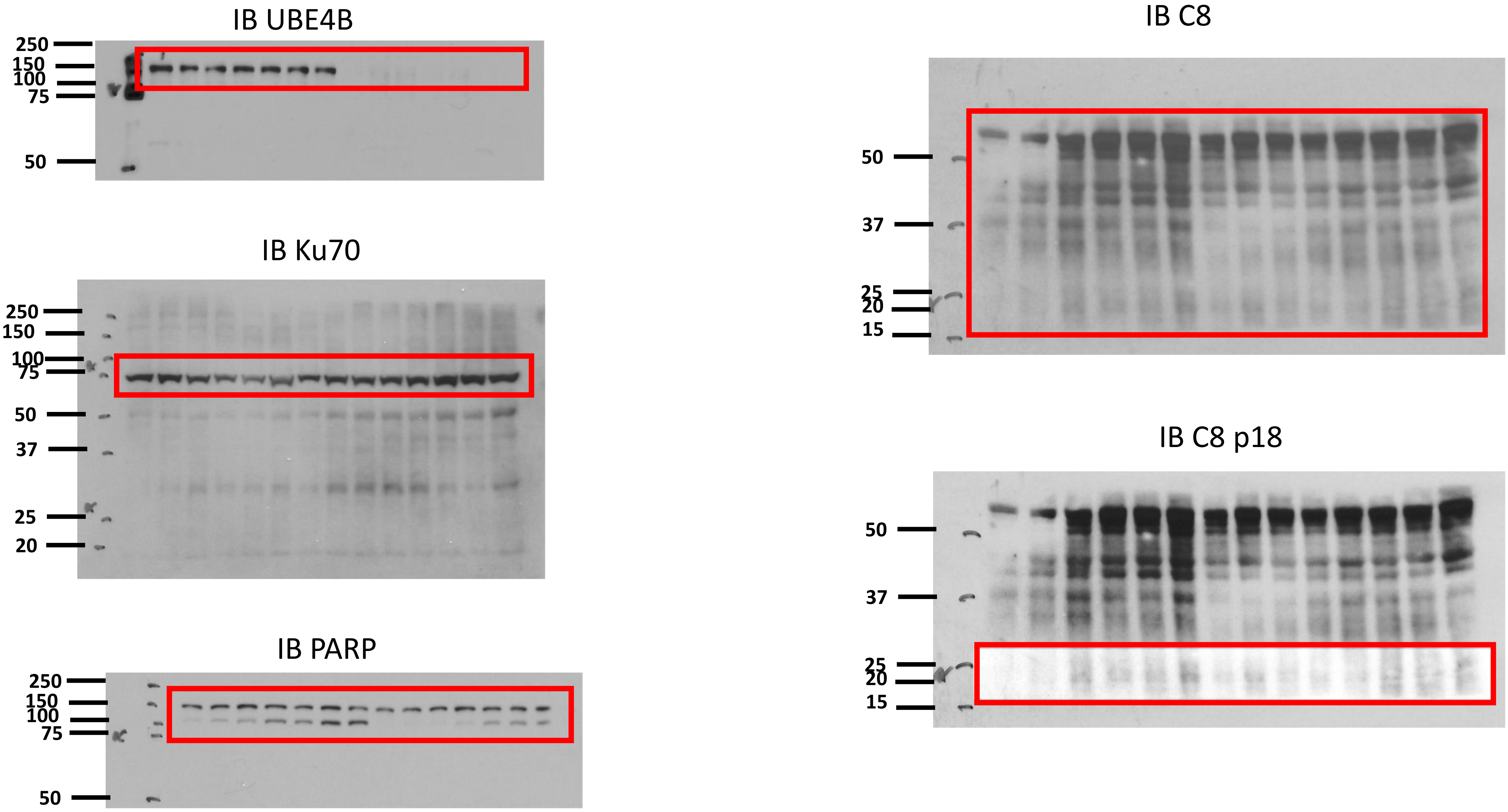

Fig 6B

SKNAS SC vs UBE4B KD Saha 0-5uM 24h

IB c-FLIP

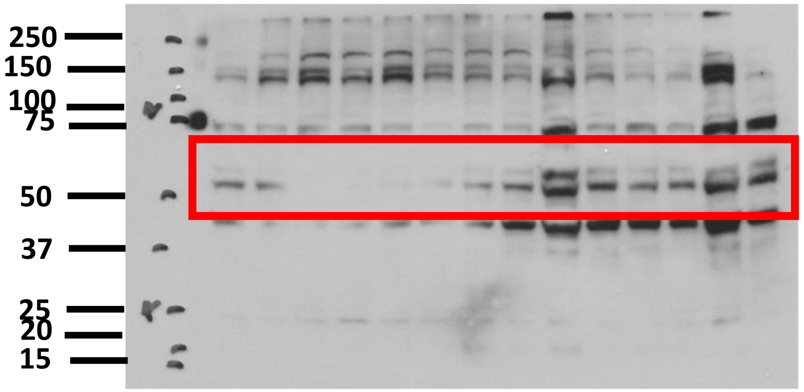

IB ITCH

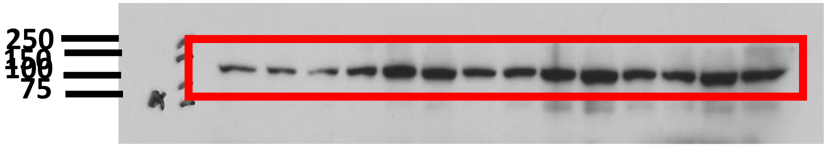

IB p53

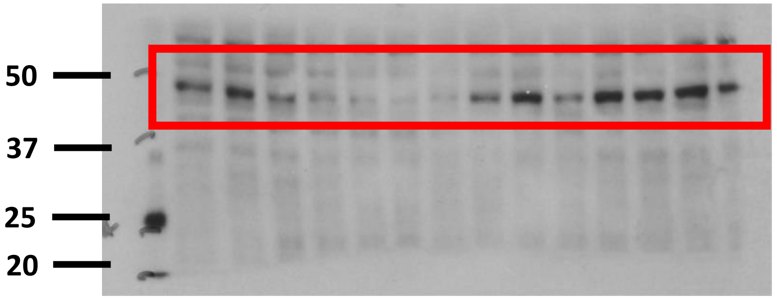

IB Actin

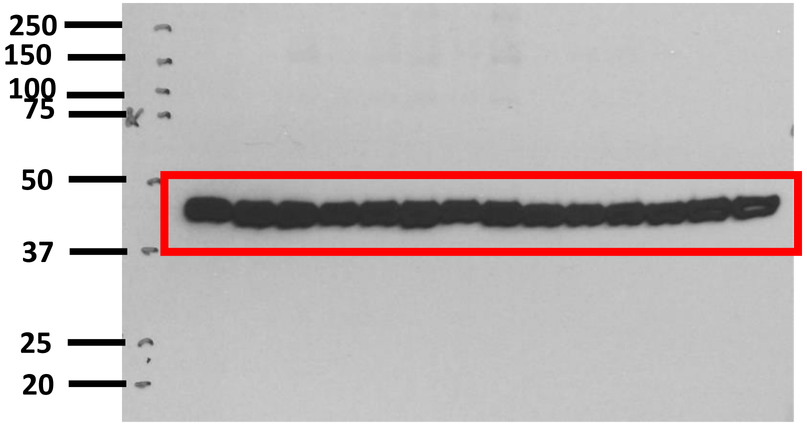

Fig 6C

SKNAS SC vs UBE4B KD Saha 0-6h 4uM

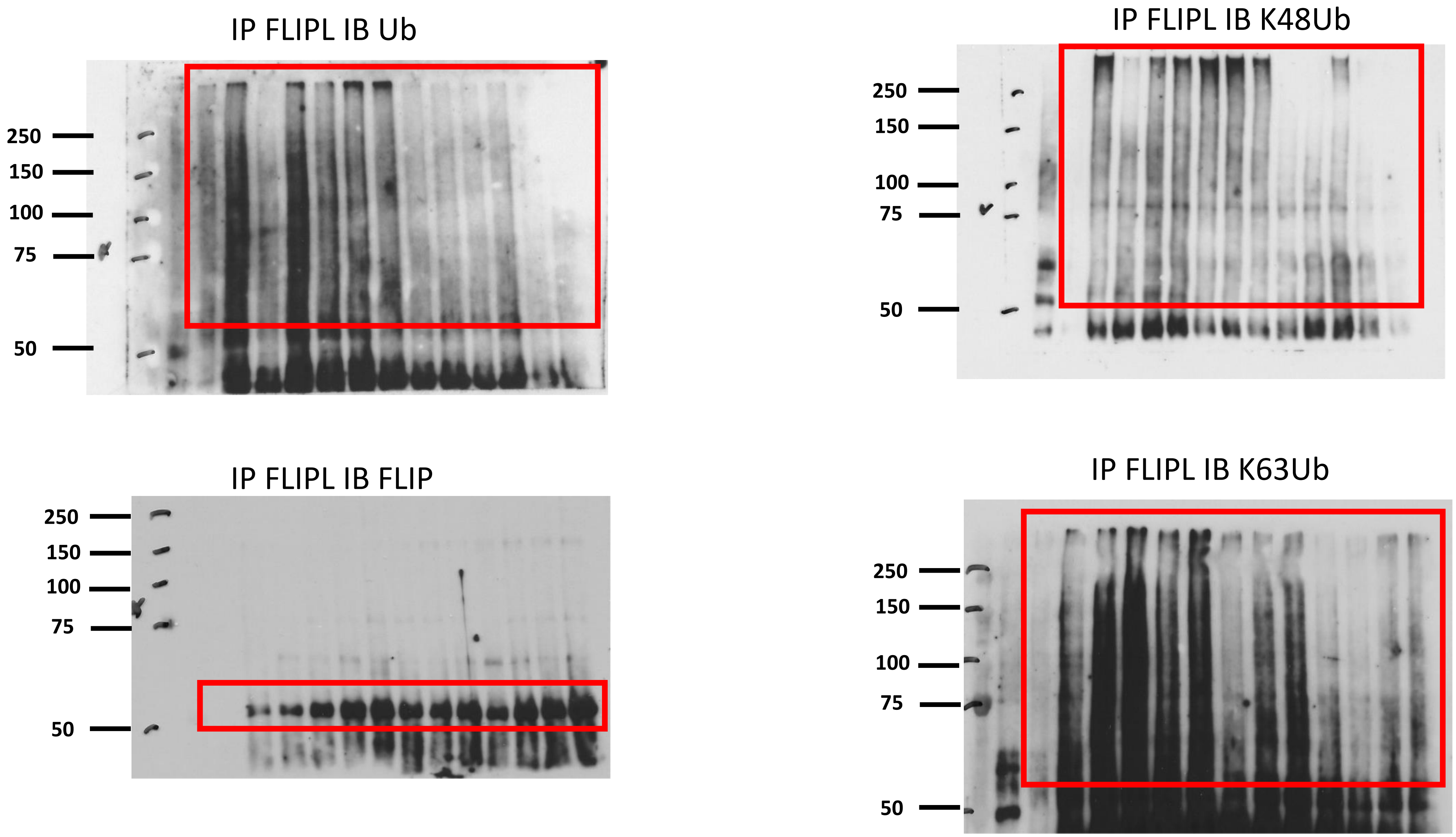

Fig 6C

SKNAS SC vs UBE4B KD Saha 0-6h 4uM

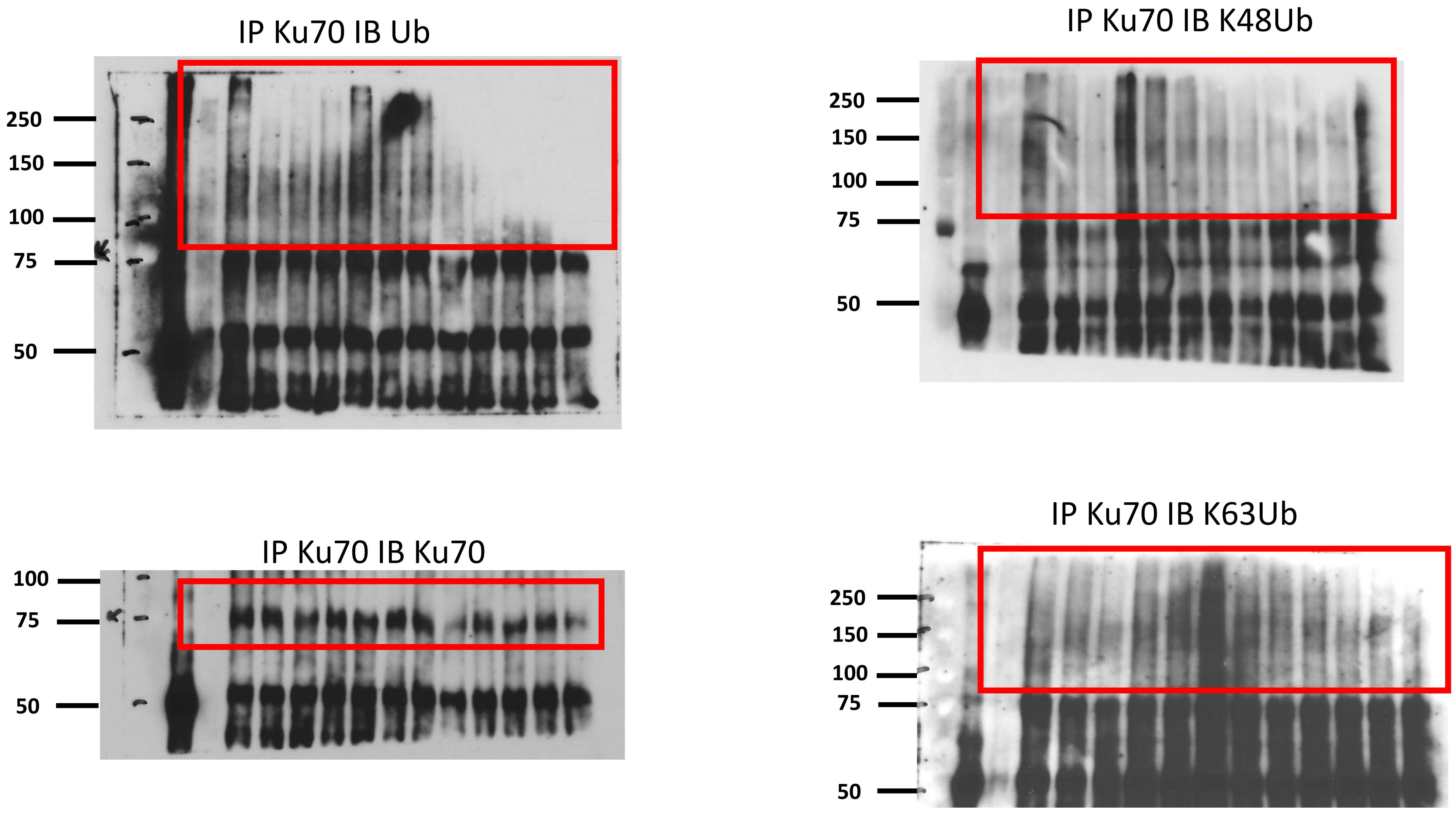

SKNAS SC vs UBE4B KD Saha 0-6h 4uM +MG

Fig 6C

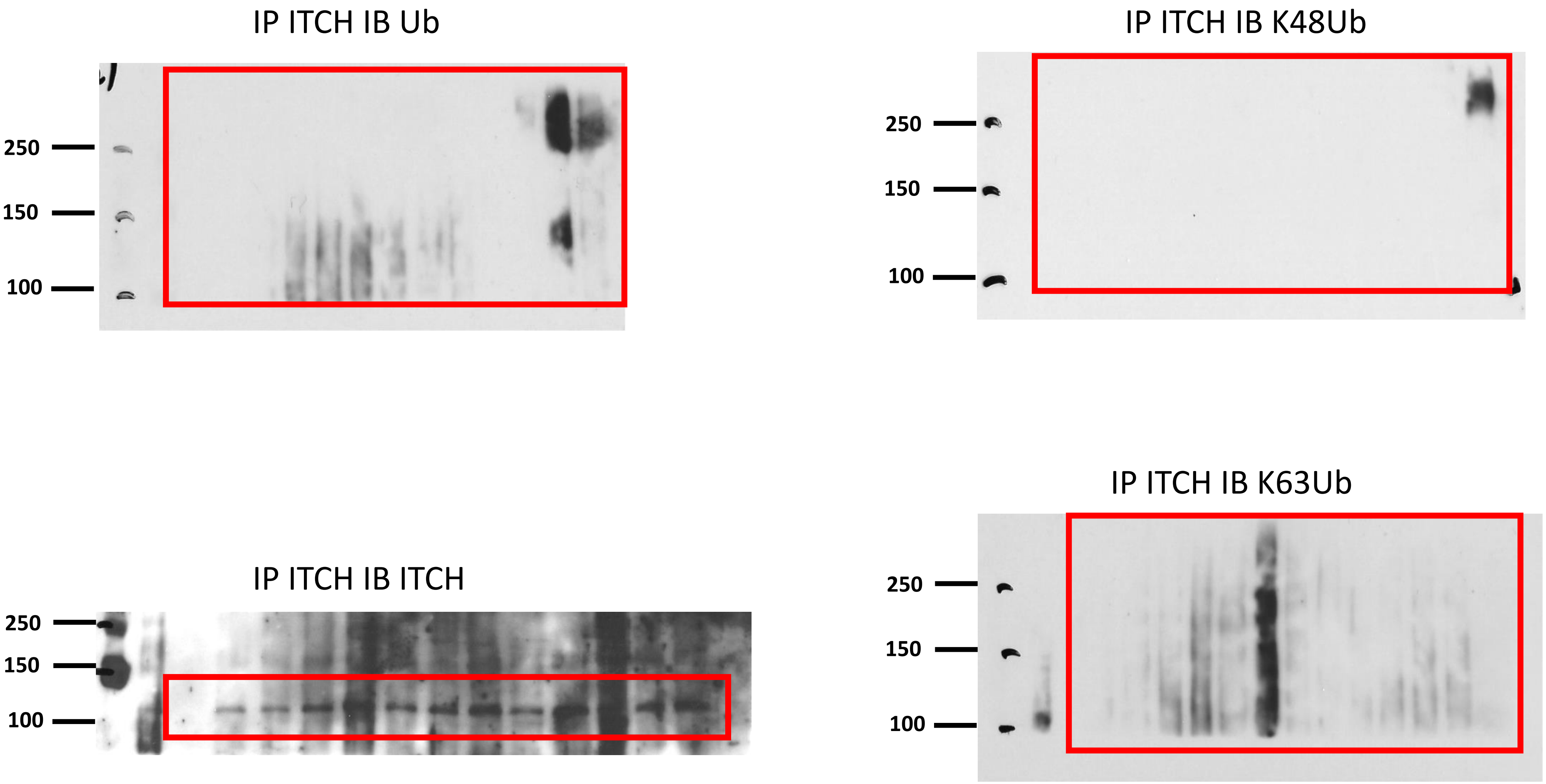

SKNAS SC vs UBE4B KD Saha 0-6h 4uM +MG

Fig 6C

IP ITCH IB UBE4B

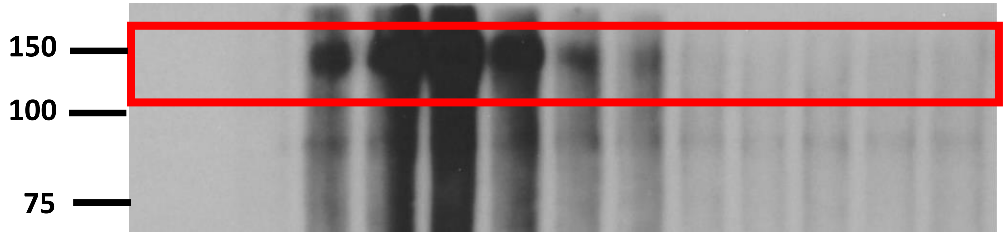

IP ITCH IB Ku70

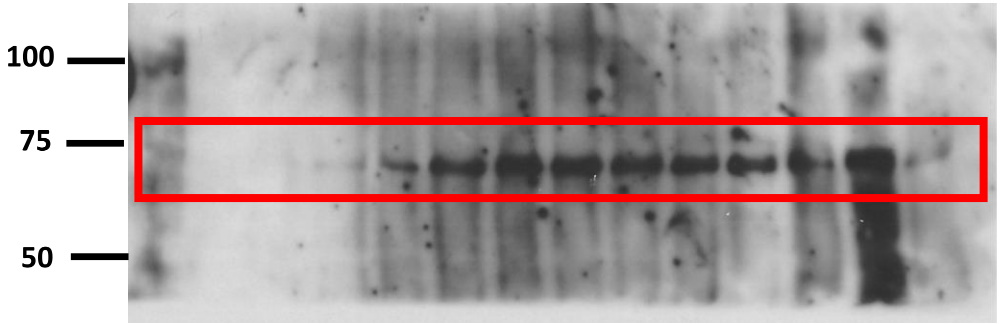

IP ITCH IB c-FLIP

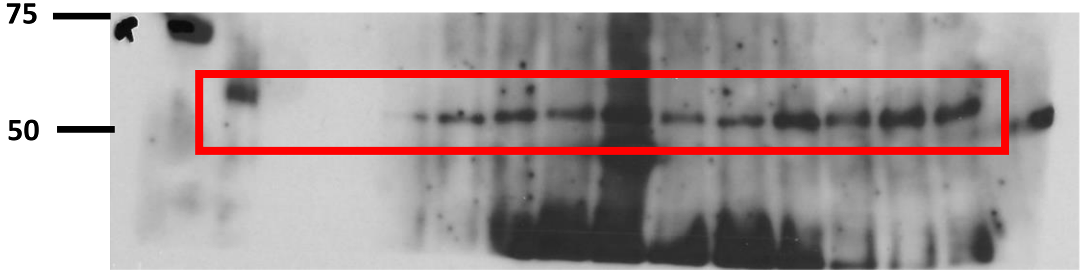

Fig 6C

SKNAS SC vs UBE4B KD Saha 0-6h 4uM
WCL

IB UBE4B

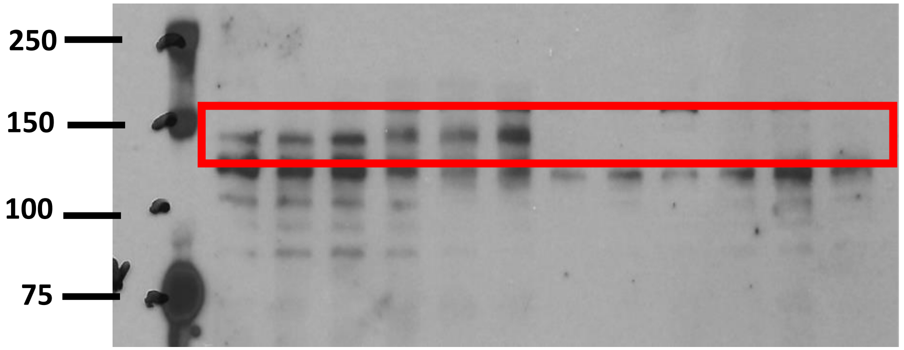

IB FLIP

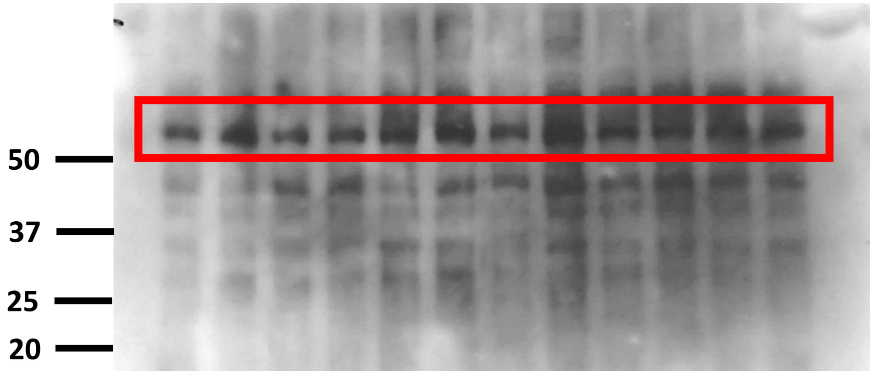

IB Actin

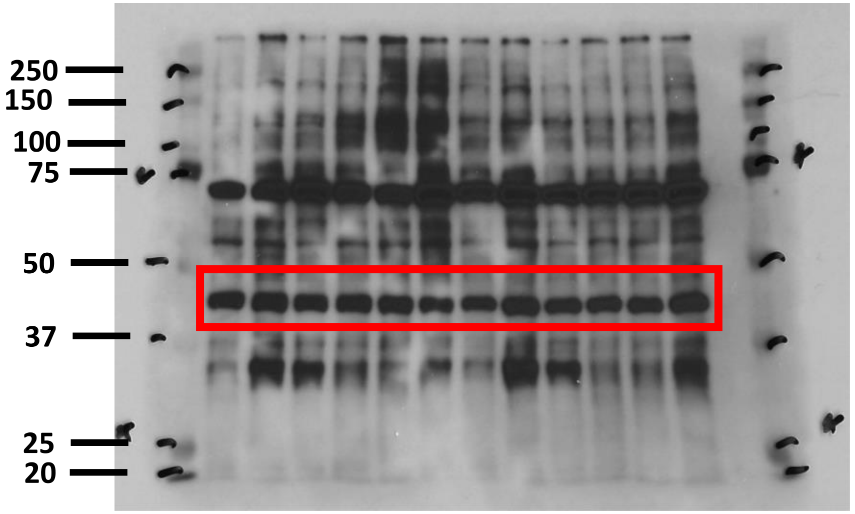

IB FLIP

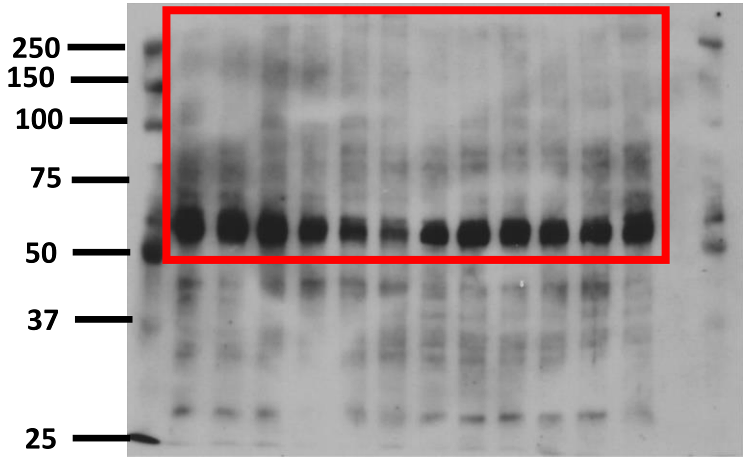

SKNAS SC vs UBE4B KD Saha 0-6h 4uM WCL

Fig 6C

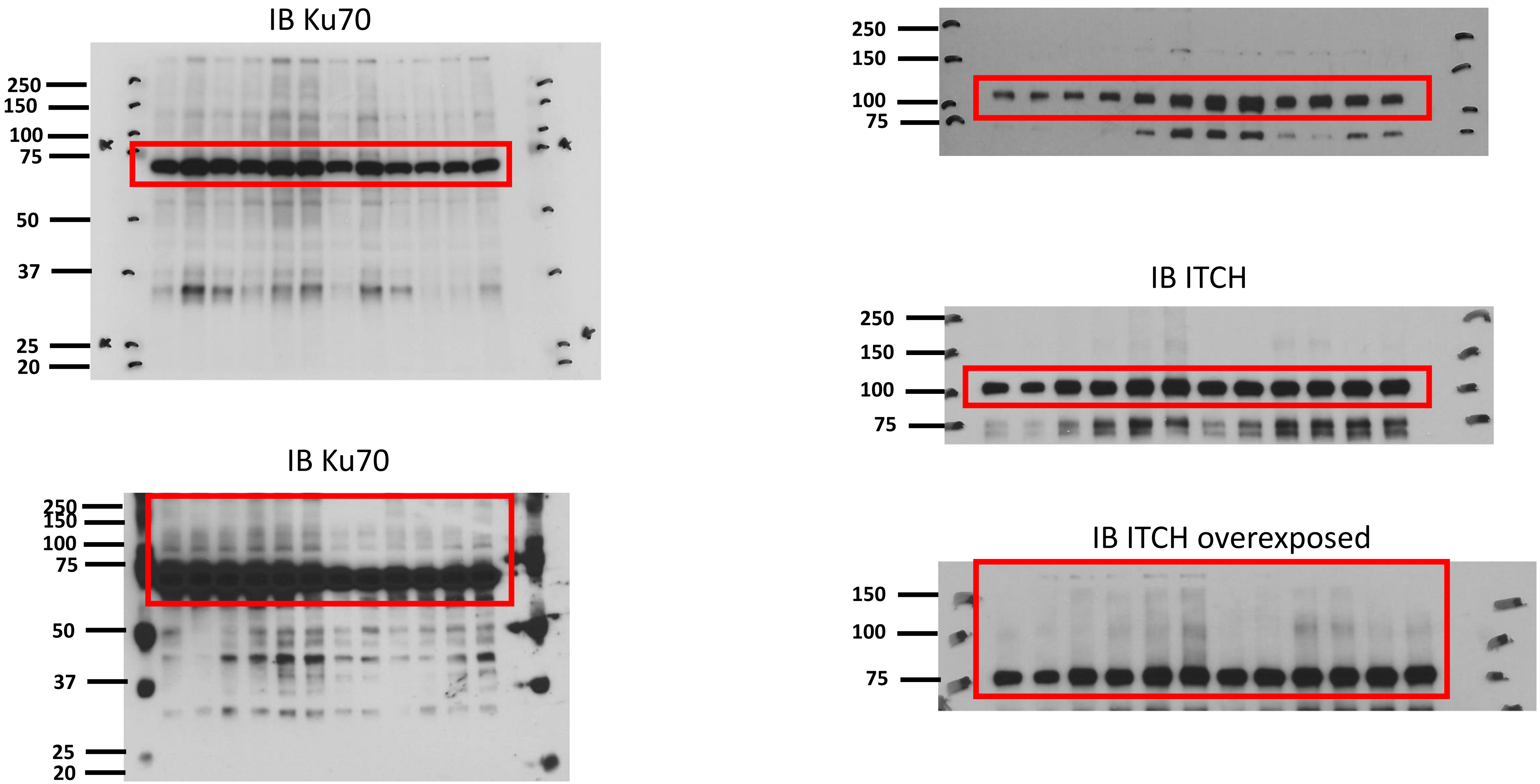

Fig 6D

SKNSH SC vs UBE4B KD Saha 0; 1; 2uM 18h

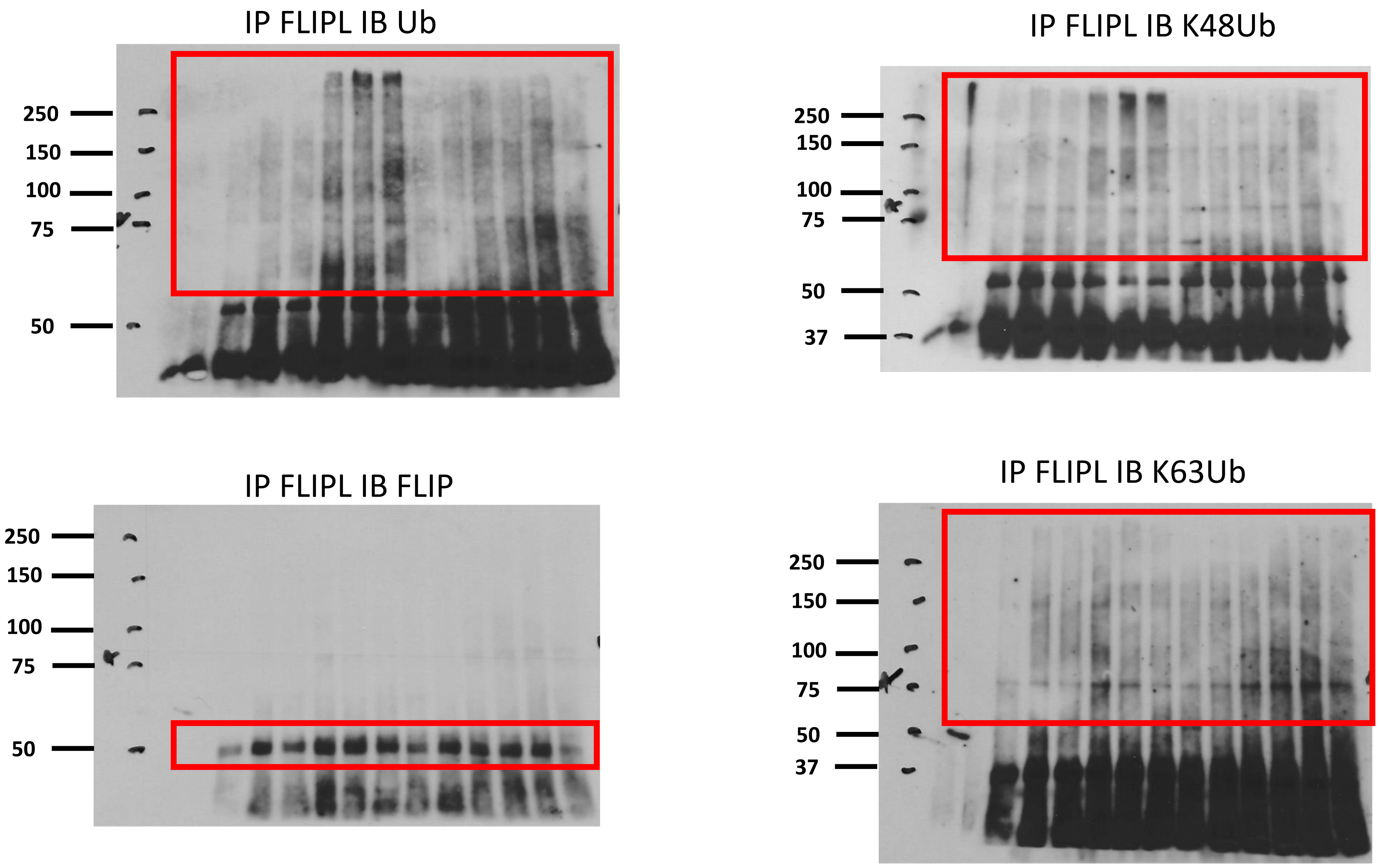

Fig 6D

SKNSH SC vs UBE4B KD Saha 0; 1; 2uM 18h

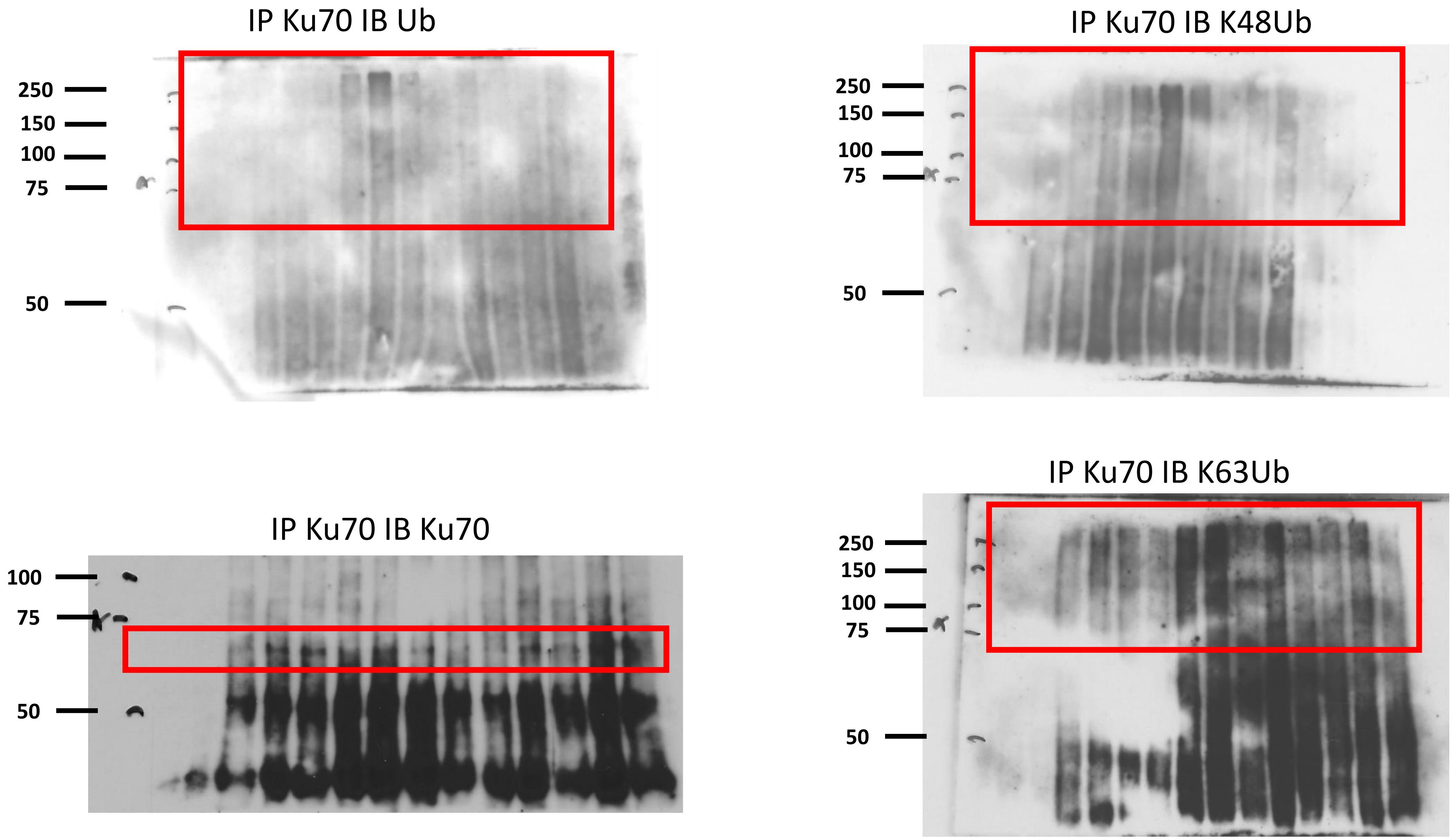

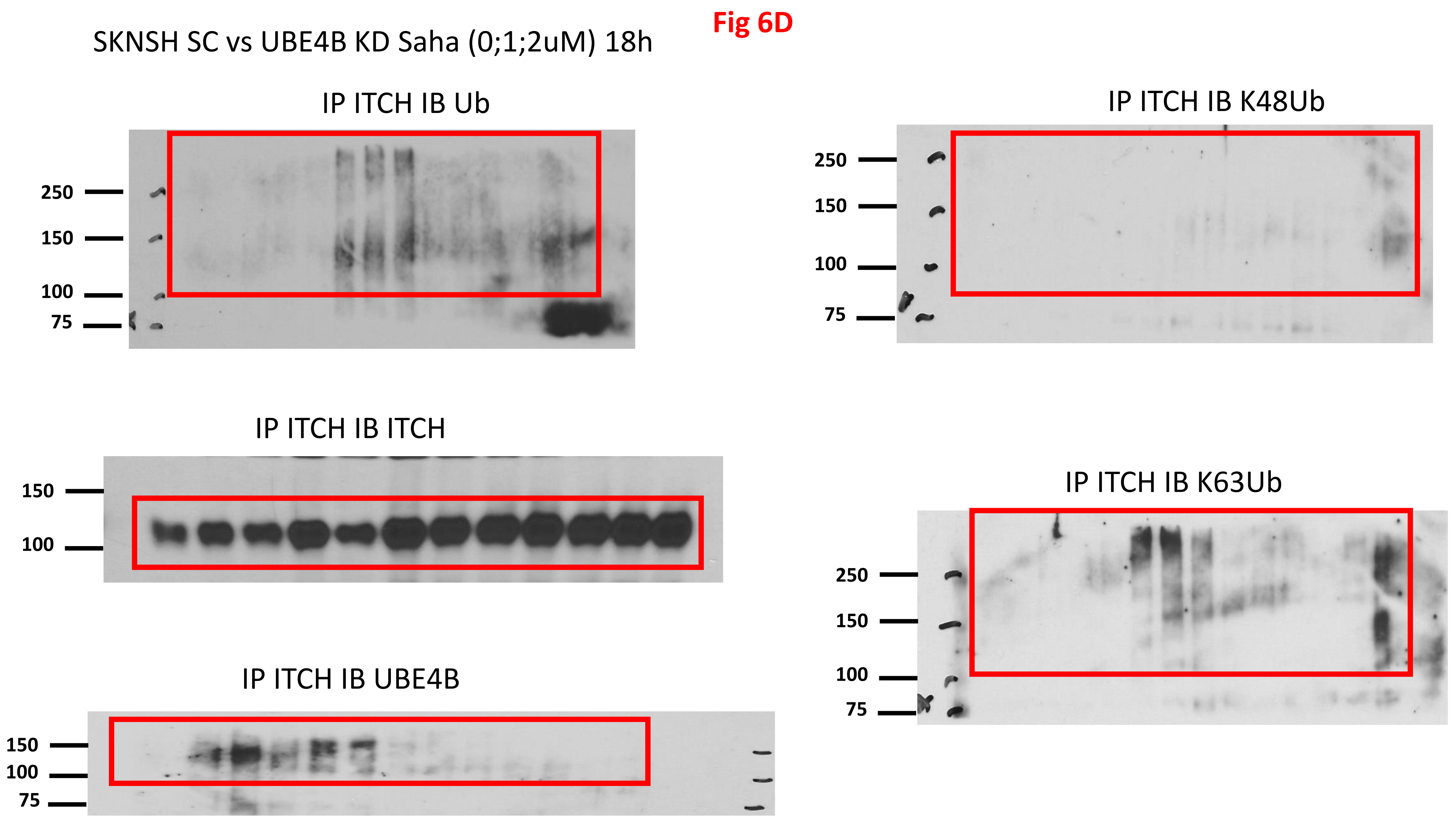
Fig 6D
SKNSH SC vs UBE4B KD Saha (0;1;2uM) 18h
IP ITCH IB Ub
250
150
100
75
IP ITCH IB K48Ub
250
150
100
75
IP ITCH IB ITCH
150
100
IP ITCH IB K63Ub
250
150
100
75
IP ITCH IB UBE4B
150
100
75

SKNSH SC vs UBE4B KD Saha (0;1;2uM) 18h

Fig 6D

IP FLIP IB UBE4B

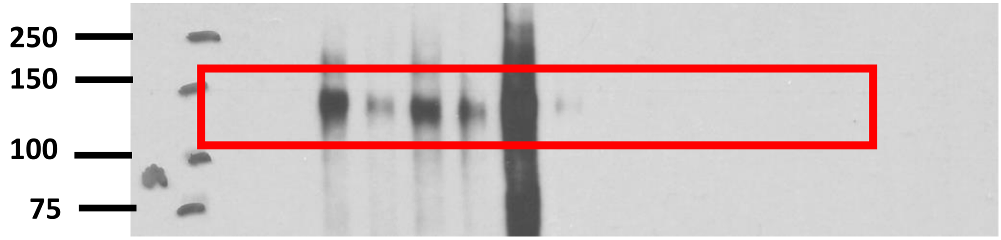

IP FLIP IB ITCH

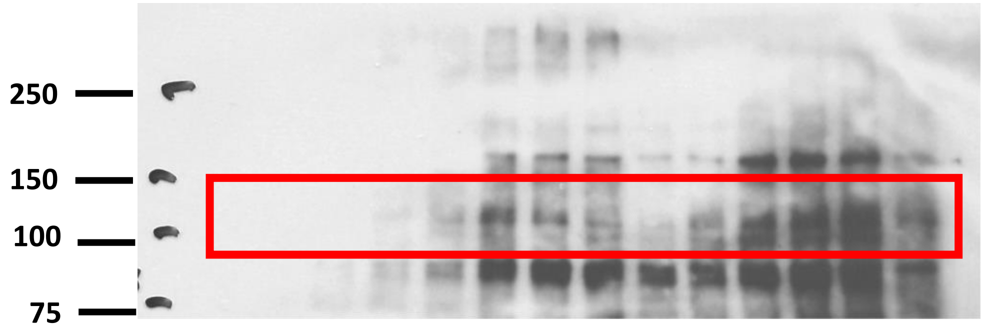

IP FLIP IB USP8

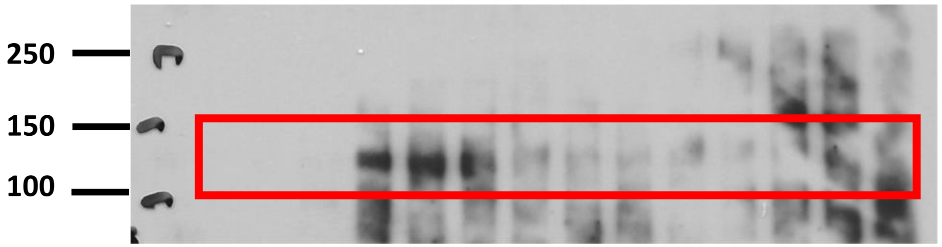

IP FLIP IB Ku70

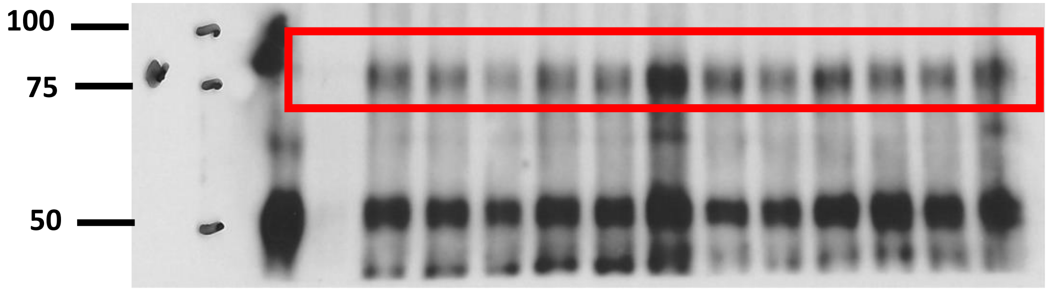

IP FLIP IB FLIP

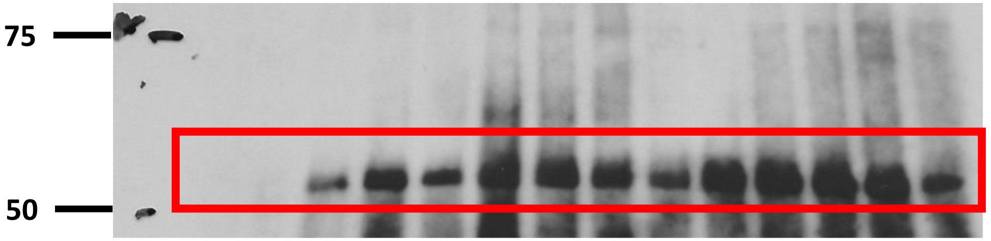

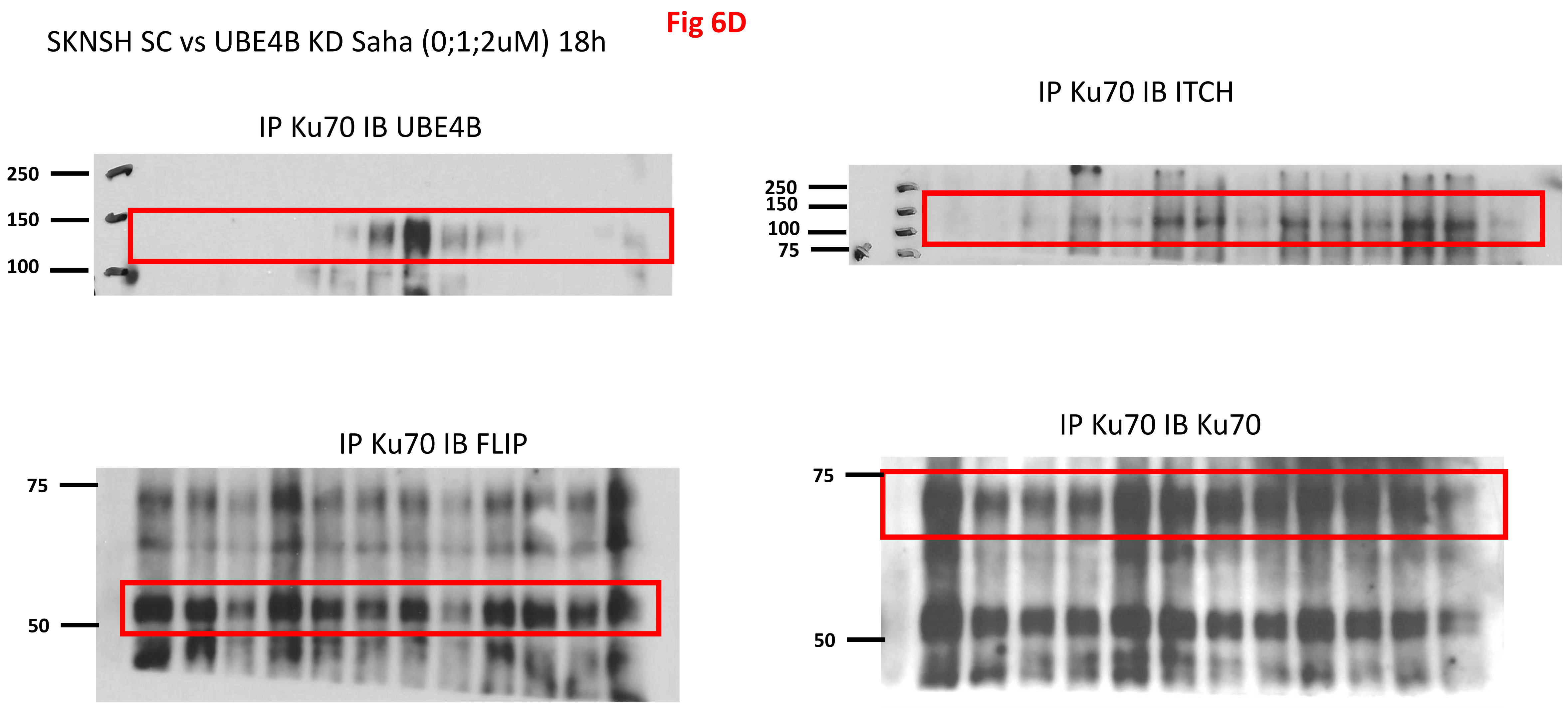
Fig 6D
SKNSH SC vs UBE4B KD Saha (0;1;2uM) 18h
IP Ku70 IB UBE4B
250
150
100
IP Ku70 IB ITCH
250
150
100
75
IP Ku70 IB FLIP
75
50
IP Ku70 IB Ku70
75
50

Fig 6D

SKNSH SC vs UBE4B KD Saha (0;1;2uM) 18h WCL

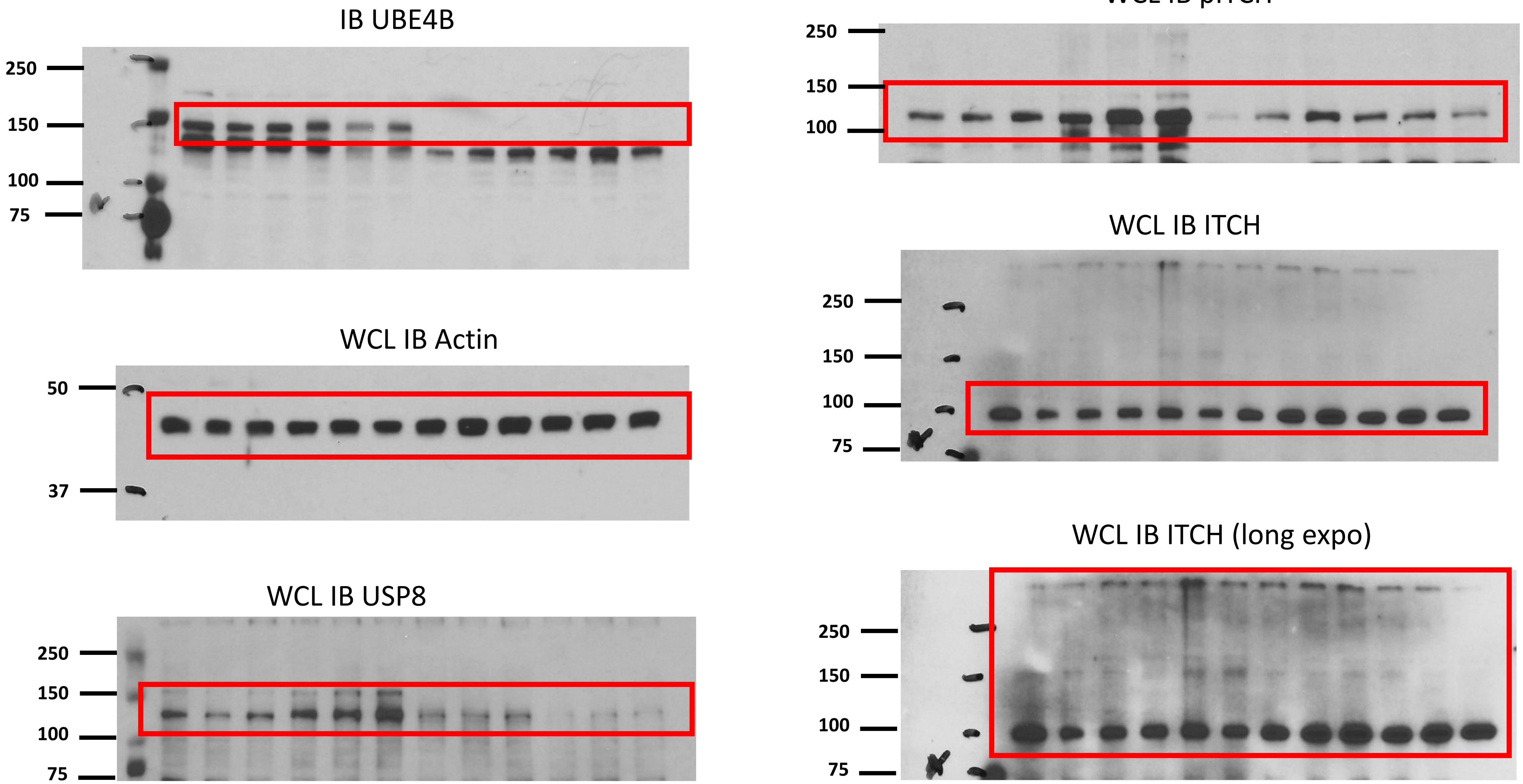

SKNSH SC vs UBE4B KD Saha (0;1;2uM) 18h WCL **Fig 6D**

IB FLIP

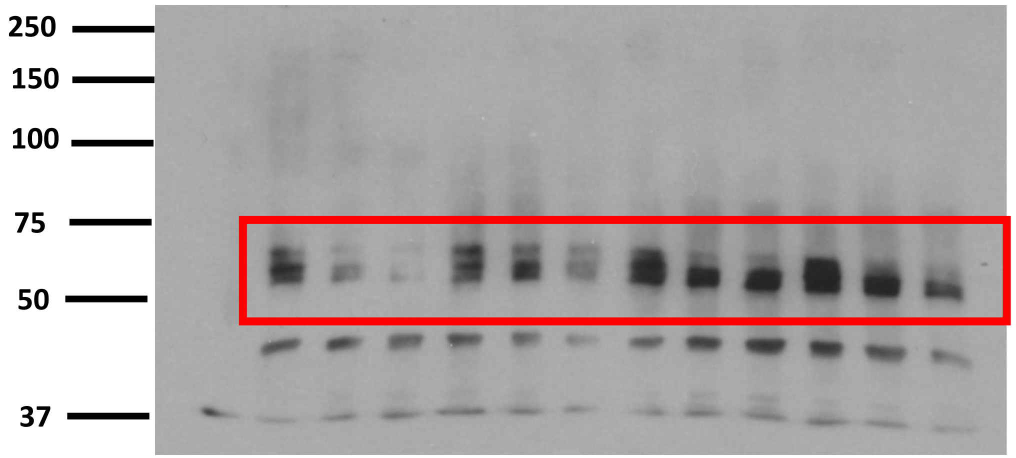

WCL IB Ku70

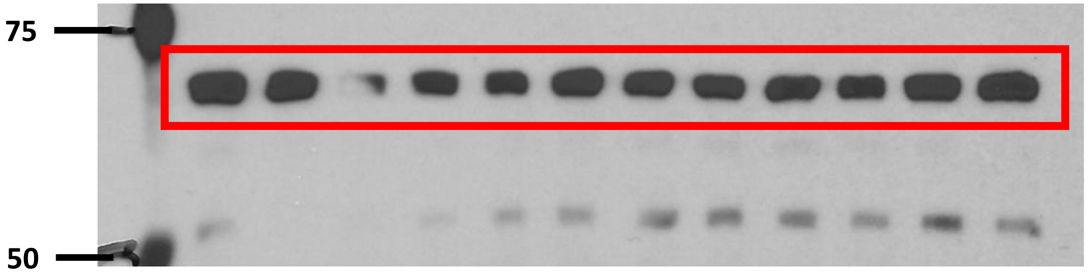

IB FLIP

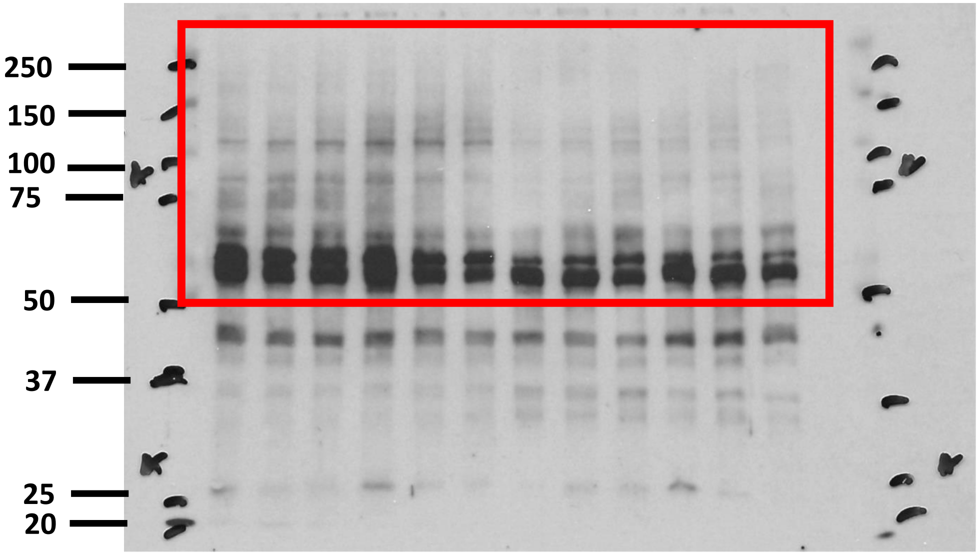

IB Ku70

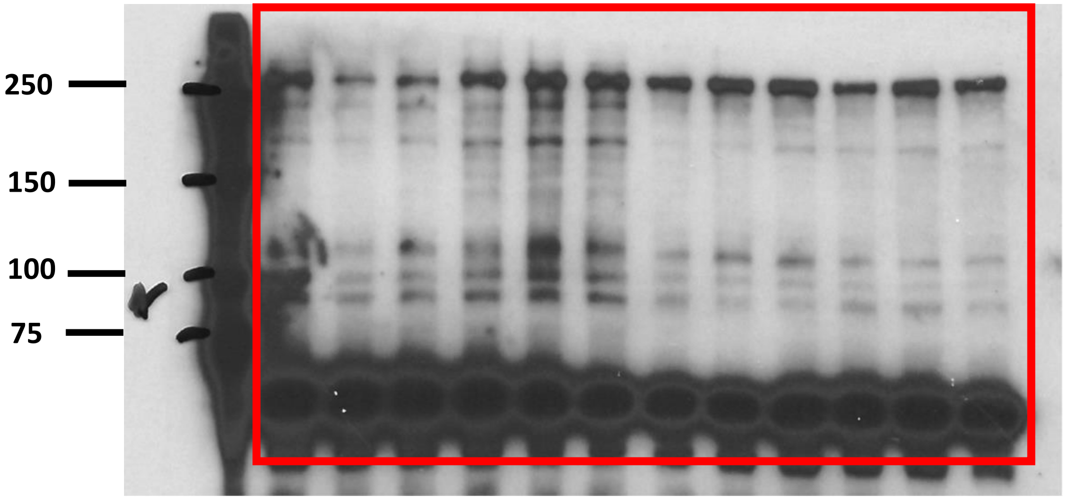

SKNAS SC vs UBE4B KD Saha 0-6h 4uM

Fig 6E

IP Acetyl K IB Ku70

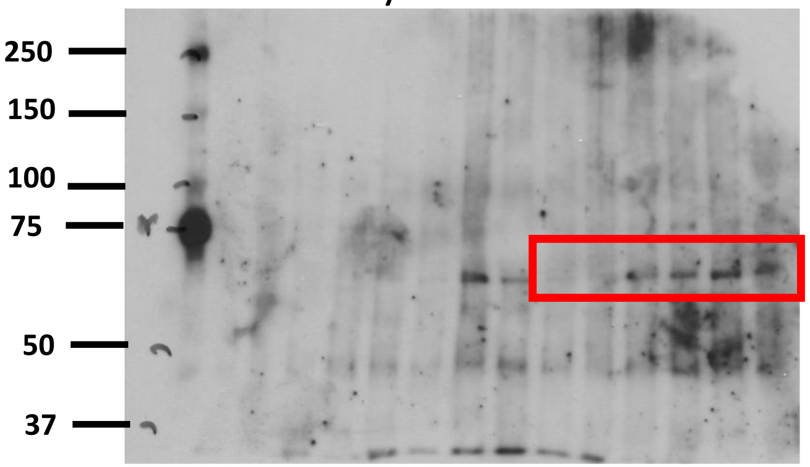

IP Acetyl K IB FLIP

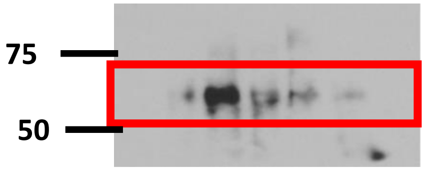

Fig 6E

SKNAS SC vs UBE4B KD Saha 0-6h 4uM

IP FLIP IB Ku70

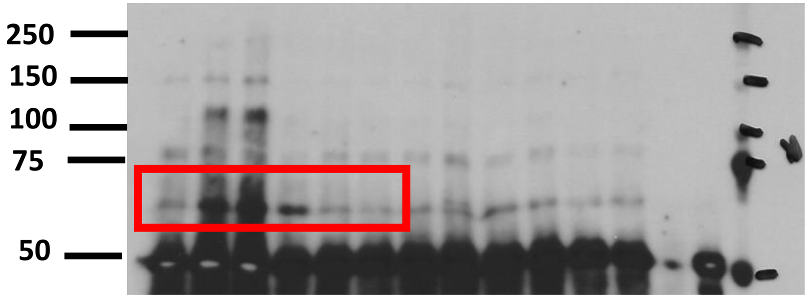

IP FLIP IB USP8

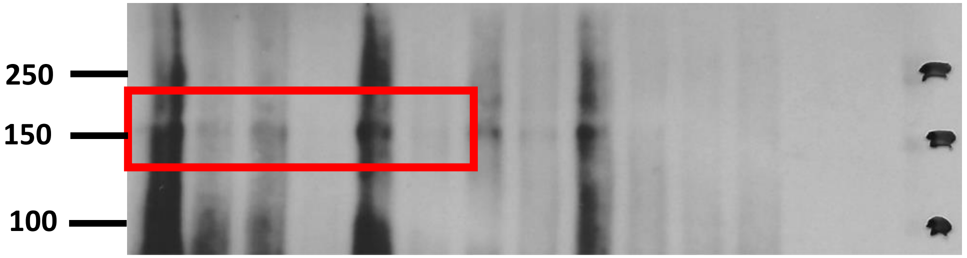

IP FLIP IB ITCH

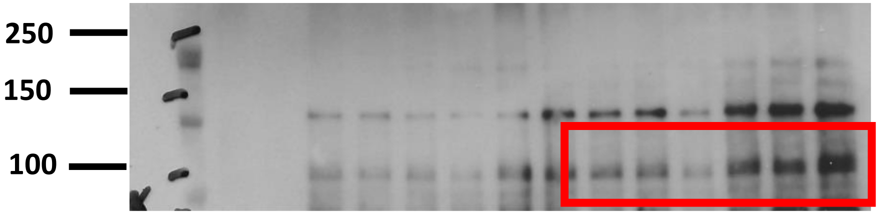

IP FLIP IB FLIP

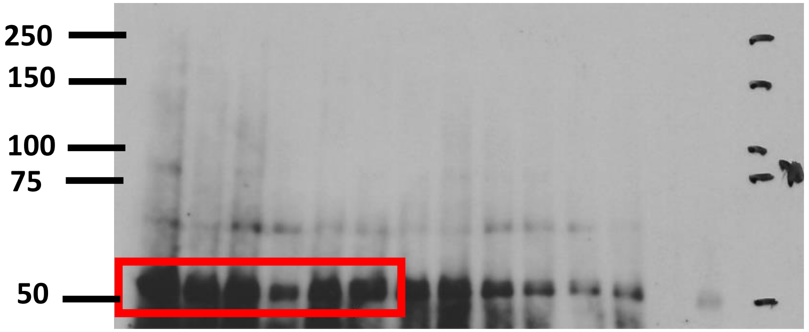

IP FLIP IB UBE4B

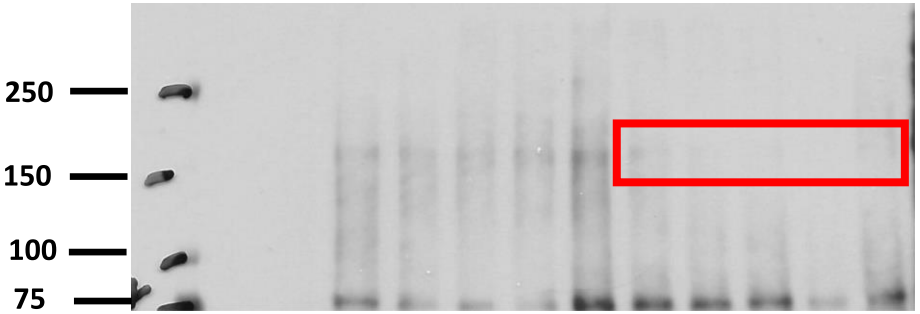

Fig 6E

SKNAS SC vs UBE4B KD Saha 0-6h 4uM

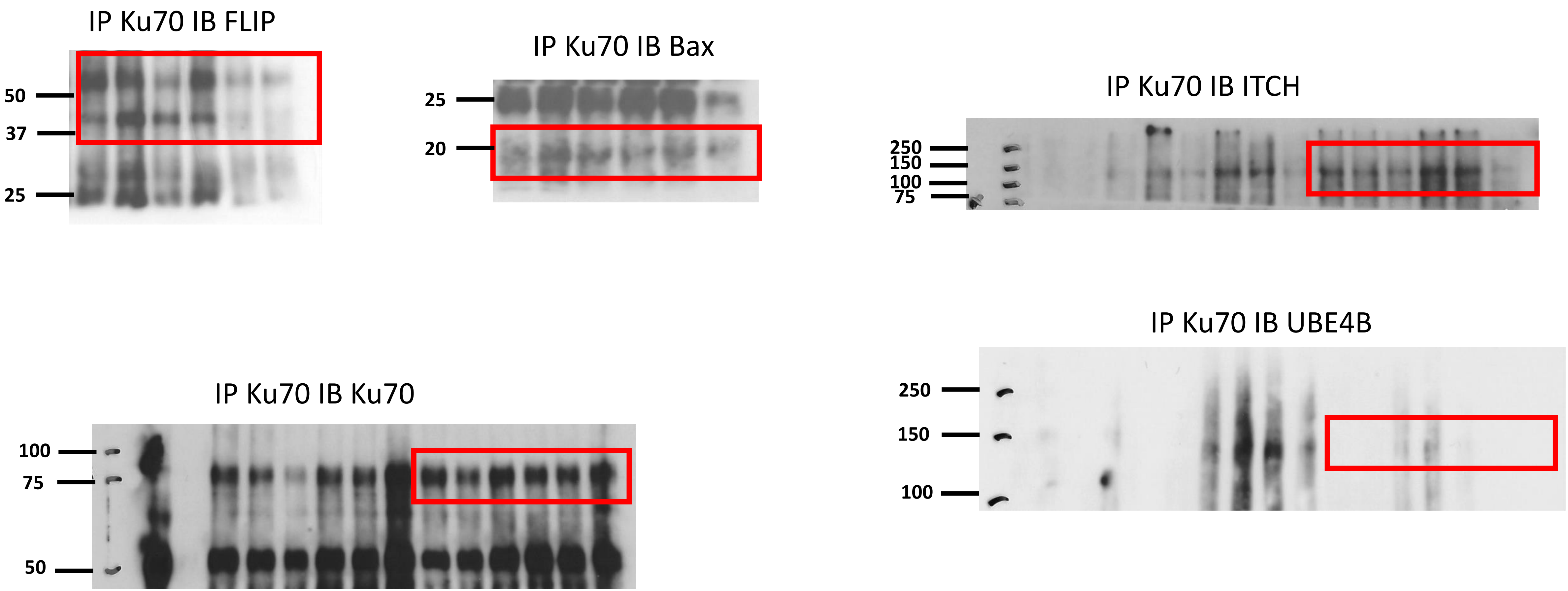

Fig 6E

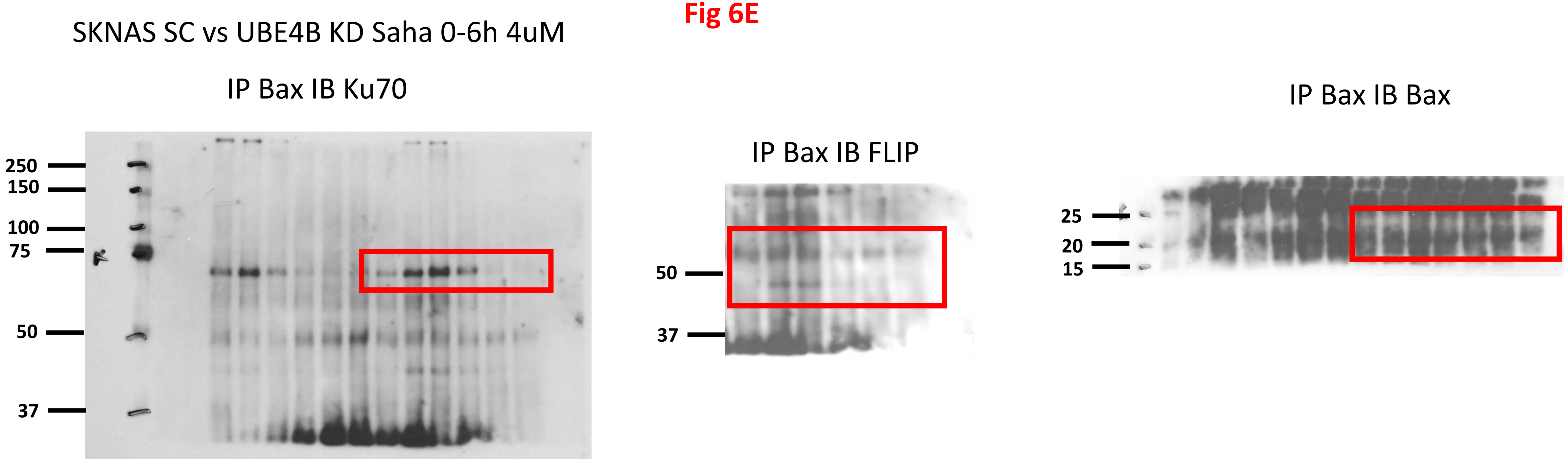
SKNAS SC vs UBE4B KD Saha 0-6h 4uM
IP Bax IB Ku70
250
150
100
75
50
37
IP Bax IB FLIP
50
37
IP Bax IB Bax
25
20
15

Fig 6E

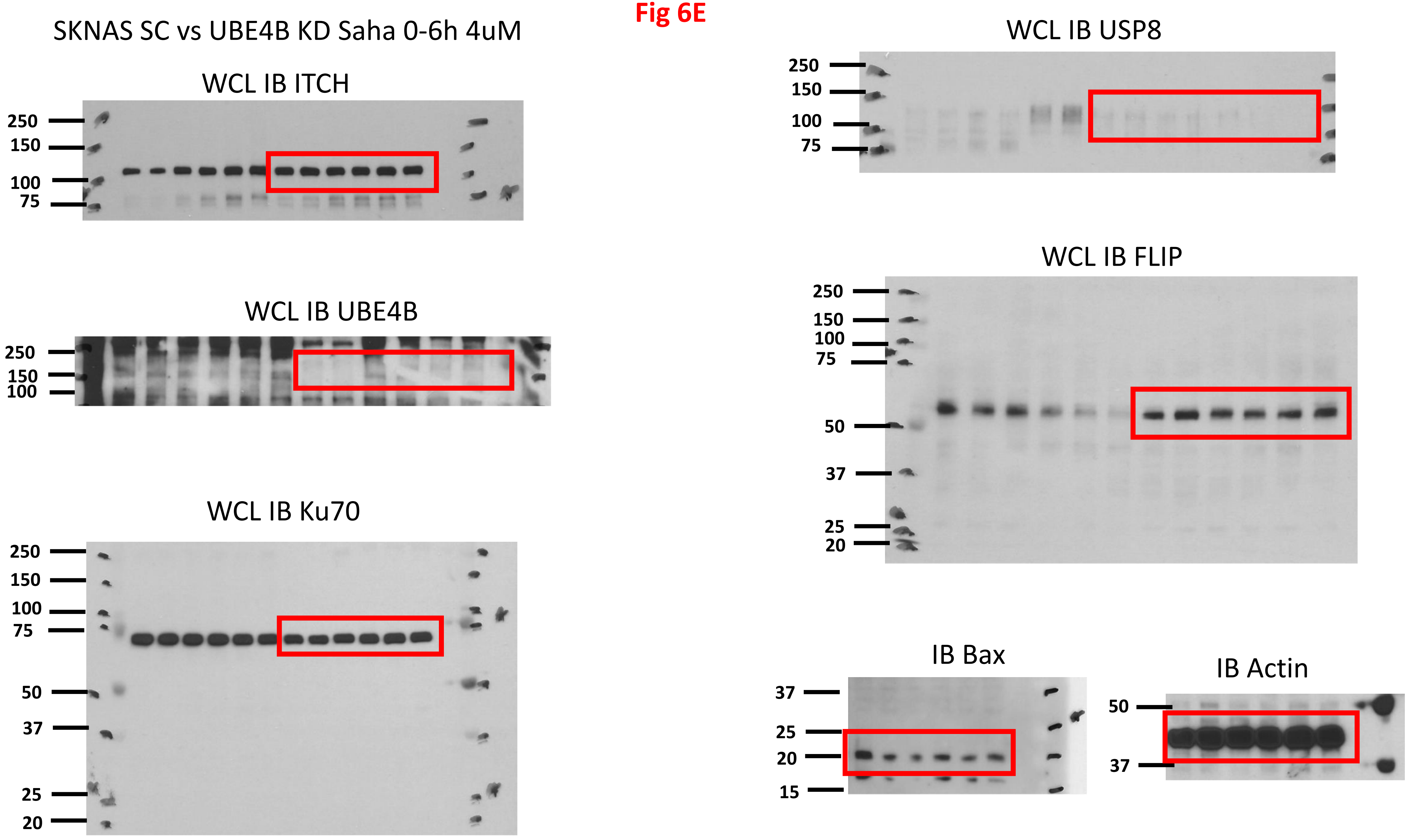

SKNAS SC vs UBE4B KD Saha 0-6h 4uM
WCL IB ITCH
250
150
100
75
WCL IB UBE4B
250
150
100
WCL IB Ku70
250
150
100
75
50
37
25
20
WCL IB USP8
250
150
100
75
WCL IB FLIP
250
150
100
75
50
37
25
20
IB Bax
37
25
20
15
IB Actin
50
37

Fig 6K

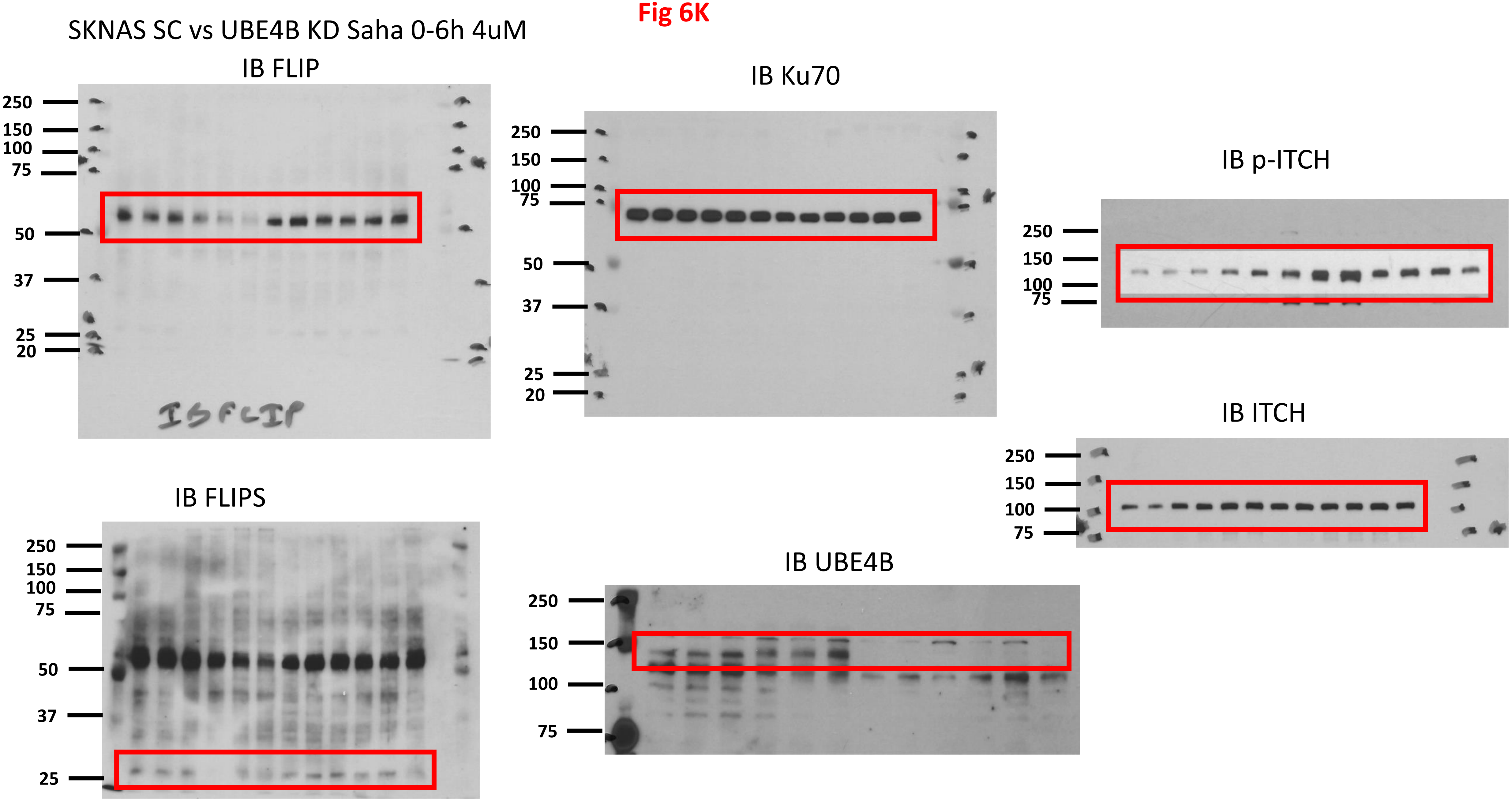
SKNAS SC vs UBE4B KD Saha 0-6h 4uM
IB FLIP
250
150
100
75
50
37
25
20
IB Ku70
250
150
100
75
50
37
25
20
IB p-ITCH
250
150
100
75
IB ITCH
250
150
100
75
IB FLIPS
250
150
100
75
50
37
25
IB UBE4B
250
150
100
75

Fig 6K

SKNAS SC vs UBE4B KD Saha 0-6h 4uM

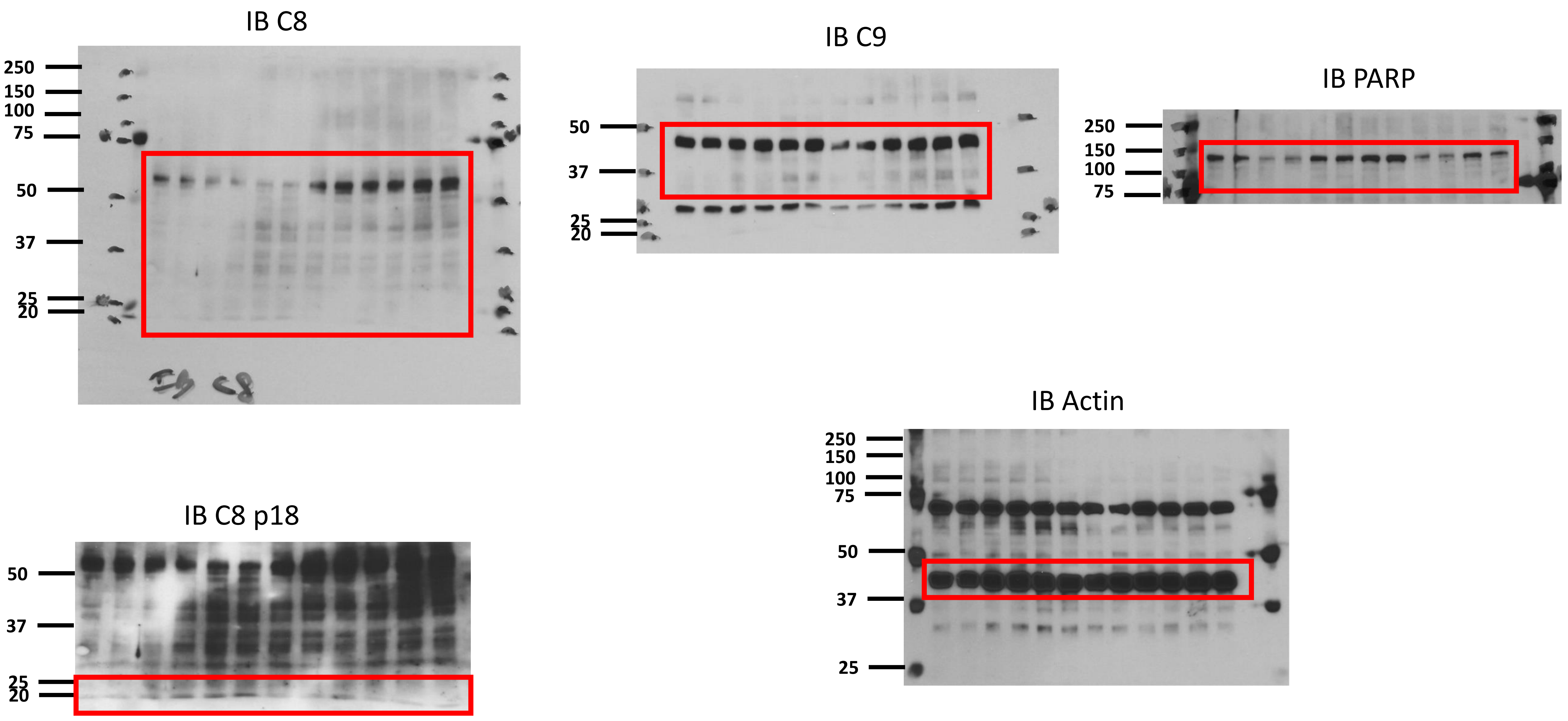

Supplement: Supplementary file 10 — Uncropped Western blots [file 41419_2023_6252_MOESM10_ESM.pdf]
